# Comparative transcriptome profiling of longissimus muscle tissues from Qianhua Mutton Merino and Small Tail Han sheep

**DOI:** 10.1038/srep33586

**Published:** 2016-09-20

**Authors:** Limin Sun, Man Bai, Lujie Xiang, Guishan Zhang, Wei Ma, Huaizhi Jiang

**Affiliations:** 1 College of Animal Science and Technology, Jilin Agricultural University, Changchun 130118, China

## Abstract

The Qianhua Mutton Merino (QHMM) is a new sheep (*Ovis aries*) variety with better meat performance compared with the traditional local variety Small Tail Han (STH) sheep. We aimed to evaluate the transcriptome regulators associated with muscle growth and development between the QHMM and STH. We used RNA-Seq to obtain the transcriptome profiles of the longissimus muscle from the QHMM and STH. The results showed that 960 genes were differentially expressed (405 were up-regulated and 555 were down-regulated). Among these, 463 differently expressed genes (DEGs) were probably associated with muscle growth and development and were involved in biological processes such as skeletal muscle tissue development and muscle cell differentiation; molecular functions such as catalytic activity and oxidoreductase activity; cellular components such as mitochondrion and sarcoplasmic reticulum; and pathways such as metabolic pathways and citrate cycle. From the potential genes, a gene-act-network and co-expression-network closely related to muscle growth and development were identified and established. Finally, the expressions of nine genes were validated by real-time PCR. The results suggested that some DEGs, including *MRFs, GXP1* and *STAC3*, play crucial roles in muscle growth and development processes. This genome-wide transcriptome analysis of QHMM and STH muscle is reported for the first time.

Sheep (*Ovis aries*) form a large part of the global animal husbandry industry. Sheep meat, with its characteristics of high protein, low fat and low cholesterol, is an important source of protein for humans. The Qianhua Mutton Merino (tentative name; abbreviated as QHMM) is a new sheep variety bred for both meat and wool. It was bred through graded crossing and artificial selection of the SA (South Africa) Mutton Merino (male parent) and Chinese Northeast Fine-wool sheep (female parent) in recent years. This new breed has genetic features of strong stress and roughage resistance, and better meat performance compared with the SA Mutton Merino and Northeast Fine-wool sheep. Gene regulation underlies all biological behaviour and phenotype; thus, investigating genetic information, which is controlled by gene regulatory factors, is one of the main challenges in molecular biology^1^. Transcription factors, a major family of gene regulatory proteins, play an essential role in the growth, development and evolution of higher organisms^2^,^3^. Therefore, investigating the transcriptome profile of muscle tissues, especially with reference to transcription regulatory proteins, would provide useful information to improve the production and quality of sheep meat.

In a narrow sense, the transcriptome usually refers to the sum of all mRNAs, because among all types of RNA, mRNA is the direct template for protein synthesis^4^. The growth and development of skeletal muscle is a complex process, which is promoted through transcriptome regulation involving regulatory networks and signalling pathways. To date, muscle regulatory factors (*MRFs*)^5^, growth hormone(*GH*)^6^, insulin-like growth factors (*IGFs*)^7^,^8^, and myostatin(*MSTN*)^9^,^10^ have been reported to be associated with muscle growth and development. To analyse the transcriptome of muscle tissue would identify more candidate genes, regulatory networks and signalling pathways at the transcriptional level.

RNA-sequencing (RNA-seq), a next-generation sequencing method with the advantages of cost-effectiveness and high-throughput, provides a genome-wide sequence readout of a transcriptome^11^,^12^. In this study, we analysed the transcriptome profile of muscle tissues of QHMM and STH sheep, which is a traditional local variety, using RNA-Seq and bioinformatics analysis. The growth rate and meat performance between two sheep breeds are significantly different. The DEGs obtained from this study will aid future investigation of the mechanism of sheep muscle growth and development, and also provide a basis for mutton sheep breeding.

## Results

### Phenotypic difference analysis

**Figure 1:**
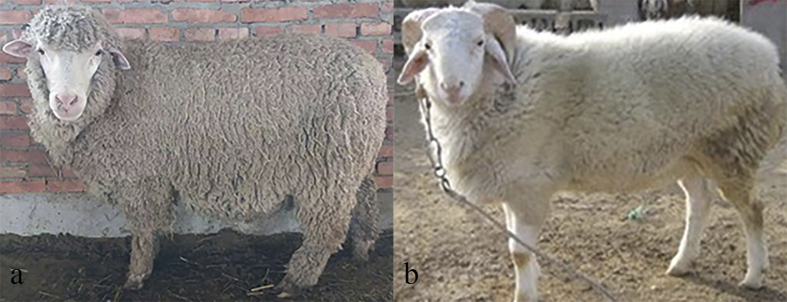
The QHMM sheep and the STH sheep. (**a**) QHMM sheep; (**b**) STH sheep.

As shown in Fig. 1a,b, compared with STH, the new breed QHMM had a good body shape, with the characteristics of no angle, wide and deep chest, straight back and well-developed hindquarters. The analyses of the carcass and meat quality are shown in Table 1. Compared with STH sheep, the cooking percentage, carcass weight, and slaughter percentage were very significantly higher (*P* < 0.01) in the QHMM sheep; live weight, net meat percentage and loin eye muscle area were significantly higher (*P* < 0.05) in the QHMM sheep; the muscle shear force and water loss rate were very significantly lower (*P* < 0.01); and the pH24 (pH of the tissue 24h post-mortem) was significantly lower (*P* < 0.005) in the QHMM sheep. These data indicated that the meat performance was significant different between the QHMM and STH sheep.

### Summary of RNA-seq data

**Figure 2:**
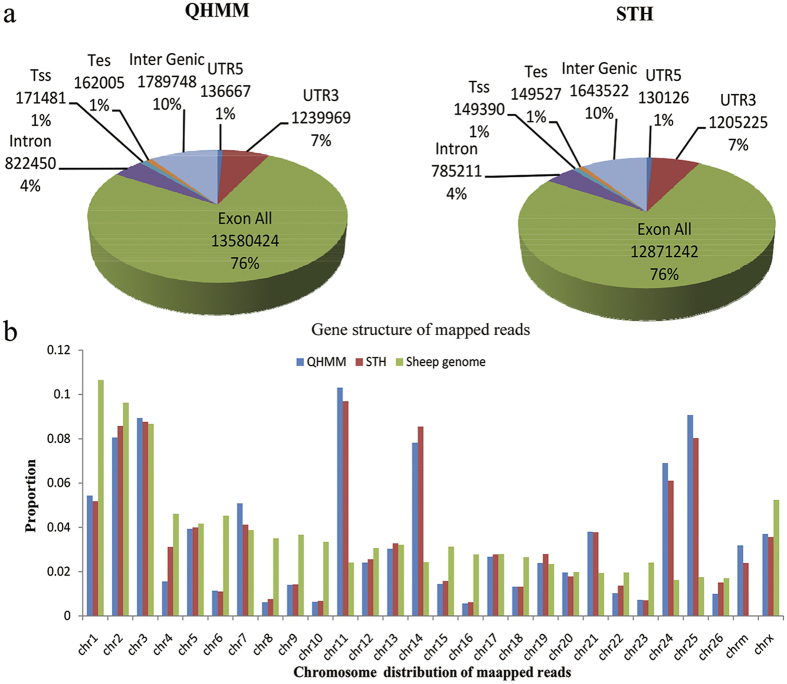
Gene structure and distribution on the chromosome analysis of mapped reads in QHMM and STH sheep. (**a**) Gene structure of mapped reads; (**b**) Chromosome distribution of mapped reads.

In this study, the result of RNA quality are shown in Supplementary Fig. S1–S3 in Supplementary Information; we obtained a total of 18.75, 17.48, 16.49, 17.46, 14.79 and 17.41 million raw reads for QHMM (A1, A2, A3) and STH (B1, B2, B3), respectively (Table 2). The raw reads were filtered and mapped to the Oar 3.1 version of the sheep genome sequence, and the unique mapped rate was 0.853–0.877. The analysis of the genes’ structures and their distribution on the chromosomes of mapped reads are shown in Fig. 2.

### Differentially expressed genes

To investigate the different muscle properties from a genetic perspective, the expression levels of the genes were quantified by the reads per kilobase of transcript per million mapped reads (RPKM) method. Then, using the EBSeq algorithm (|log_2_^FoldChange^| > 0.585 and FDR < 0.05; FDR, false discovery rate), 960 genes were considered as DEGs, including 405 that were up-regulated, such as *CCND3*, *SOCS2* and *HMOX1*, and 555 that were down-regulated, such as *SMAD3*, *NMNAT3*, *SDHC* and *MGST3* in the muscle tissues of STH vs. QHMM, respectively (Supplementary Table S1).

### Gene Ontology (GO) and Kyoto Encyclopedia of Genes and Genomes (KEGG) pathway analyses of DEGs

**Figure 3:**
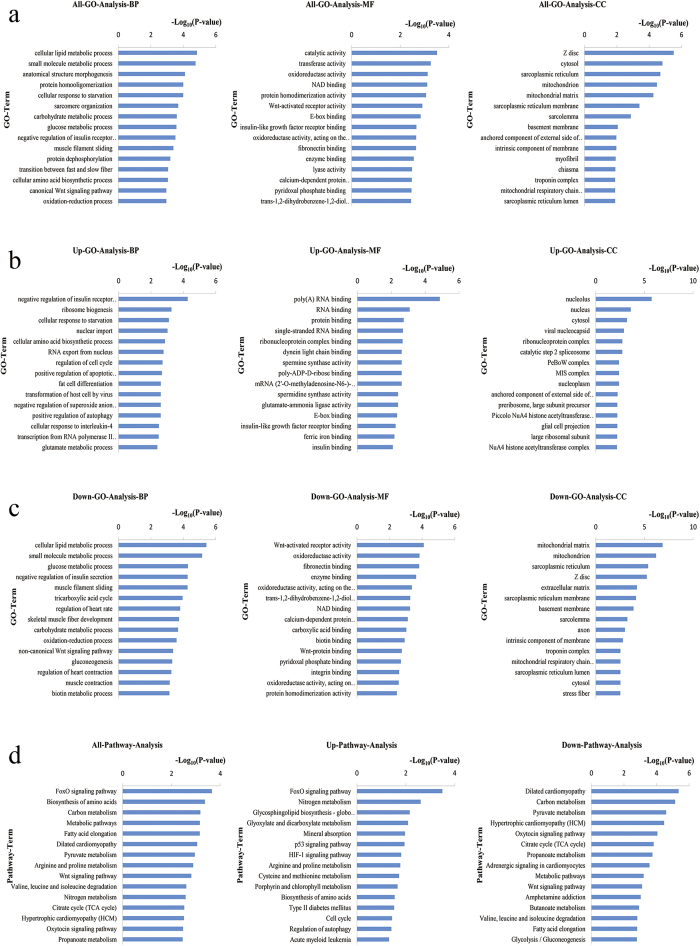
The top 15 significant GO terms and pathways of the DEGs (STH vs. QHMM; *P* < 0.05). (**a**) Significant GO terms for all DEGs; (**b**) Significant GO terms for up-regulated DEGs; (**c**) Significant GO terms for down-regulated DEGs; (**d**) Significant pathways.

**Figure 4:**
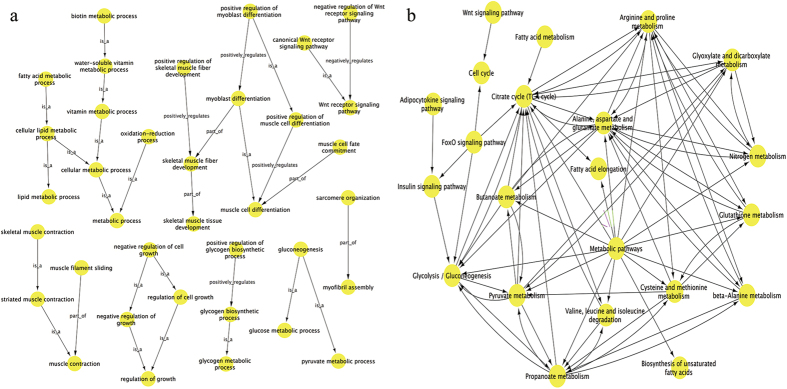
GO-trees and pathway-act-network analysis of DEGs. (**a**) GO-trees analysis of DEGs; (**b**) Pathway-act-network analysis of DEGs. The arrow between two nodes represents an interaction target between GO-terms or pathways.

The 960 DEGs identified in the longissimus dorsi of QHMM and STH sheep were annotated according to three GO categories: biological process, molecular function, and cellular component. Significance analysis revealed the significant enrichment (P < 0.05) of 252 GO terms (including cellular lipid metabolic process, small molecule metabolic process, and fatty acid metabolic process), 81 GO terms (including wnt-activated receptor activity, oxidoreductase activity, and catalytic activity), and 43 GO terms (including mitochondrion, sarcoplasmic reticulum, and Z disc) for all DEGs in the categories of biological process, molecular function, and cellular component, respectively (Supplementary Table S2). GO terms significantly enriched for up- and down-regulated DEGs are shown in Supplementary Tables S3 and S4. The top 15 significant GO terms among the three categories are shown in Fig. 3a–c. After GO annotation and significance analysis of GO terms, to further understand the key GO terms and select DEGs that are probably associated with growth and development of muscle, 82 significantly enriched GO terms (Supplementary Table S5) were selected to create a visual GO-Trees picture, based on the hierarchical and subordinate relationship of the GO terms, as shown in Fig. 4a. The figure indicates that the DEGs mainly influenced biological process such as metabolic process, regulation of growth, skeletal muscle tissue development and glycogen metabolic process. We selected ten GO terms (skeletal muscle tissue development, positive regulation of skeletal muscle fibre development, skeletal muscle fibre development, positive regulation of myoblast differentiation, positive regulation of muscle cell differentiation, muscle cell fate commitment, muscle cell differentiation, sarcomere organization, myofibril assembly and myoblast differentiation) that are directly involved in the biological process of muscle growth and development, and 30 DEGs associated with these GO terms were obtained (Table 3).

The pathway annotation of DEGs was performed using the KEGG database. The results showed that 53 pathways were significantly (P < 0.05) enriched among all DEGs (Supplementary Table S6), pathways significantly enriched for up- and down-regulated DEGs are shown in Supplementary Table S6. The top 15 significant pathways are shown in Fig. 3d. Moreover, pathway analysis showed that these DEGs are mainly involved in carbohydrate metabolism processes, including glycolysis/gluconeogenesis, TCA cycle and pyruvate metabolism; lipid metabolism processes, such as fatty acid elongation; amino acid metabolic processes containing arginine and proline metabolism, valine, leucine and isoleucine degradation, cysteine and methionine metabolism; cell growth and apoptosis; and signal transduction processes, such as the FoxO and Wnt signalling pathways. To further investigate the interactions of pathways and to obtain the key significant pathways that play pivotal roles in muscle growth and development, a pathway-act-network^13^,^14^,^15^ was explored based on the direct or systemic interactions of 23 selected significantly enriched pathways (Supplementary Table S7) in KEGG database. As shown in Fig. 4b, the metabolic pathways; citrate cycle (TCA cycle); and alanine, aspartate and glutamate metabolism, were located in the centers of each pathway clusters and showed the most interactions with surrounding pathways. This indicated that these pathways were probably the most vital pathways. Arginine, proline metabolism, glycolysis/gluconeogenesis, glyoxylate and dicarboxylate metabolism, as well as propanoate metabolism, in the pathway-act-network also played essential roles.

### Gene-act-network and co-expression analysis

**Figure 5:**
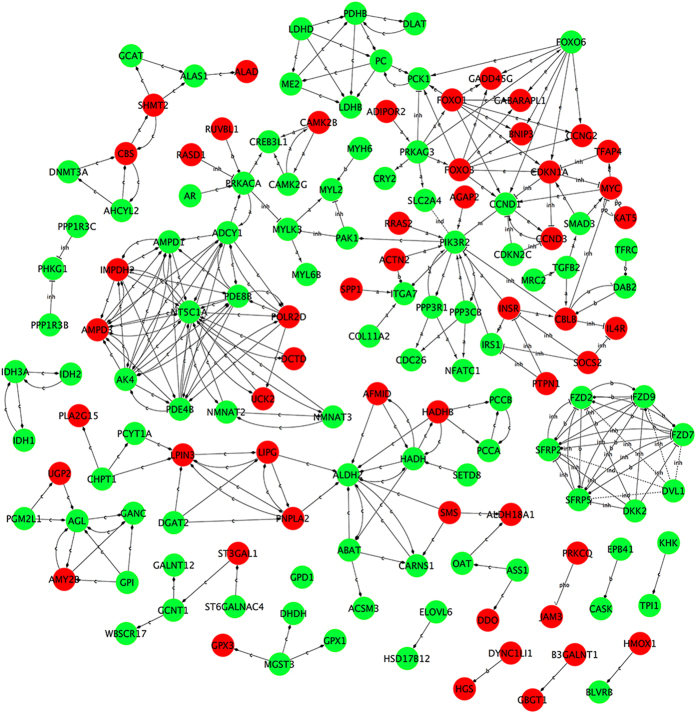
Gene-act-network analysis of DEGs. Red nodes represent the up-regulated mRNAs while green nodes represent the down-regulated mRNAs. (a: activation; b: binding/ association; c: compound; (e: expression; ind: indirect effect; inh: inhibition; m: missing interaction; pho: phosphorylation; u: ubiquitination).

**Figure 6:**
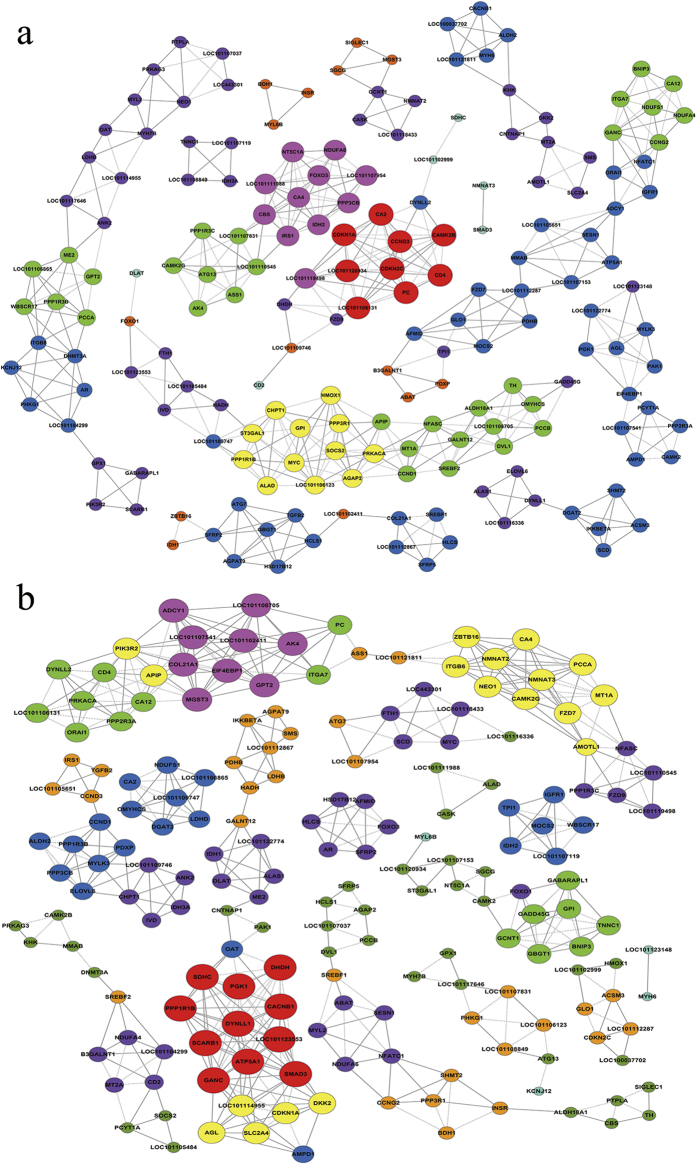
Co-expression-network analysis of DEGs for QHMM and STH. (**a**) Co-expression-network analysis of DEGs for QHMM. (**b**) Co-expression-network analysis of DEGs for STH. The solid lines represent the positive correlation while the dotted lines represent the negative correlation. The same color node represent the same tendency of gene expression, the node size represents the co-expression ability of gene, the greater the node size, the greater the k-core value.

After GO analysis and pathway analysis, 463 DEGs (Supplementary Table S8) that probably regulated muscle growth and development were selected from 82 significantly enriched GO terms and 23 significantly enriched pathways. To further explore the interactions between these DEGs, the gene-act-network^14^,^15^ was established based on the relationships between these DEGs in terms of expression, activation and indirect activation, phosphorylation and dephosphorylation, binding and compounds, inhibition and missing interactions according to KEGG database. As shown in Fig. 5, in the gene-act-network, not only were the processes of signal transduction visible, but also related genes involved in essential regulatory function were more easily discovered. The genes *MYH6, MYL2, MYL6B, MYLK3, ITGA7, GPX1* and *PPP3CB*, which were previously mentioned in Table 3, were involved in the gene-act-network. The results identified gene interactions. Alternatively, we built a co-expression-network based on the DEGs in muscle tissues of QHMM and STH to highlight the synergy of DEGs groups that are functionally related or co-regulated^16^. As shown in Fig. 6, 185 network nodes and 509 connections were found in the QHMM group, while 186 network nodes and 535 connections were observed in the STH group. The “k-core” factor was then applied to identify the key regulatory genes, which probably play pivotal roles in gene interactions and regulations. Nine DEGs with the highest k-core values were identified in the QHMM group, while eleven DEGs with highest k-core values were identified in the STH group (Supplementary Table S9). Moreover, DEGs including *MYH6, MYL2, MYL6B, MYLK3, NEO1, OMYHCS* and *PPP3CB*, previously mentioned in Table 3, were co-expressed with other genes in the co-expression-network of both the QHMM group and STH group. DEG *GPX1*, also listed in Table 3, was only in the co-expression-network of the STH group.

### Validation of RNA-Seq data

**Figure 7:**
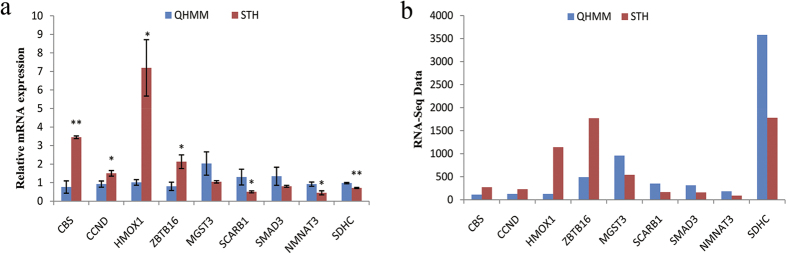
Real time PCR validation of DEGs in QHMM and STH, (**a**) RT-PCR analysis of 9 DEGs, the values were calculated by the 2^−ΔΔCt^ method, *P < 0.05, **P < 0.01. (**b**) RNA-seq data, the values were calculated and normalized by EBSeq algorithm, the fold changes were more than 1.5 and FDR < 0.05.

Nine DEGs were selected randomly to validate the RNA-Seq data by qRT-PCR. The data from the qRT-PCR analysis were calculated by the 2^−ΔΔCt^ method^17^ and the detailed results are shown in Fig. 7. Compared with QHMM sheep, the DEGs *CBS, CCND, HMOX1* and *ZBTB16* were up-regulated, while *MGST3, SCARB1, SMAD3, NMNAT3* and *SDHC* were down-regulated in the STH sheep, and *CCND, HMOX1, ZBTB16, SCARB1* and *NMNAT3* were significantly differently expressed (P < 0.05), *CBS* and *SDHC* were very significantly differently expressed (P < 0.01). The expression levels of these genes determined by qRT-PCR were consistent with the RNA-Seq data, which validated the accuracy of the RNA-Seq data.

## Discussion

Meat performance is an important economic trait in animal husbandry, and is also important reference index in sheep breeding. In the present study, we studied the phenotypic difference between QHMM and STH sheep. The data showed that compared with the local, traditional sheep STH, QHMM, which is a new breeding variety for both mutton and wool, has a significantly different meat production performance and meat quality. Our results highlighted the phenotype difference and genetic difference between these two breeds. To investigate the potential genetic mechanism of the difference in muscle growth and development between the two sheep breeds, a genome-wide transcriptome analysis of the mRNA expressed in the muscle tissues of the two sheep breeds was performed. The present study is the first genome-wide transcriptome analysis of QHMM for the genes related to muscle growth and development.

Muscle growth and development are often regulated by core genes and signal transduction pathways^18^,^19^. We therefore attempted to identify the key genes and signal pathways related to muscle growth and development in the present study. Recently, RNA-seq has emerged as a new technique to determine the expression level of the whole genome, and allows a fast and comprehensive determination of almost all transcripts of a species, thus expanding the frontiers of animal genetics^20^. Previous studies identified 387 DEGs between Dorset and STH sheep^21^ that are involved in stress response, unfolded protein response, myoblast cell fate determination, and the extracellular matrix, and 1300 DEGs between Dorper and STH sheep^22^ using the RPKM algorithm in muscle tissues by RNA-seq. Compared with previous studies, we identified 960 DEGs from the muscle tissues of QHMM and STH sheep by RNA-seq, including 405 that were up-regulated and 555 that were down-regulated. We also found that these DEGs are associated with myoblast cell fate determination and the extracellular matrix, as observed previously^21^.

GO analysis of the DEGs was helpful for understanding the main function of these DEGs. We selected 82 significantly enriched GO terms to create a GO-Trees picture, to identify the core genes in muscle growth and development process. As a result, 10 GO terms that are directly involved in biological process of muscle growth and development and 30 DEGs associated with these ten GO terms were obtained. Among these DEGs, importantly, *MYOG* and *MYOD* were up-regulated in QHMM, while *MYF6* was down-regulated. Previous studies showed that *MYOG*, *MYOD* and *MYF6* are regulatory factors belonging to the muscle regulatory factors (MRFs) family, which has central functions in early muscle differentiation, and muscle growth and development^23^,^24^. *MYOG* and *MYF6* are involved mainly in the fusion and differentiation of myoblast^25^,^26^. *MYOD* is a marker of skeletal muscle satellite cell proliferation^27^. Moreover, *MYL2*, *MYL6B* and *MYLK3* belong to the myosin light chain (MYL) family, and *OMYHCS* and *MYH6* belong to myosin heavy chain (MHC) family, myosin being composed of MHC and MYL. Importantly, myosin is the main component of myofibrillar thick filaments and plays a vital role in muscle growth and contraction^28^. Additionally, previous research indicated that *GXP1* was essential for muscle progenitor cell function and the integrity of muscle differentiation^29^. *STAC3* was identified as a nutrient regulated gene, which is highly expressed in skeletal muscle^30^. *CSRP3* plays a fundamental role in skeletal muscle proliferation and differentiation^31^ and in the maintenance of normal muscle structure and function in terms of myofibre size and sarcomere length^32^. *BTG*, which is expressed in the sarcomere, was identified^33^. There have been few reports of other DEGs with functions related to muscle growth and development. Although these previous studies enhanced the accuracy of our prediction of key genes, deeper and further study of these 30 DEGs are required to identify their functions in muscle growth and development process. From the GO-Trees, we also found metabolic regulation subnetwork, including biotin metabolic process, lipid metabolic process and oxidation-reduction. A regulation of growth subnetwork, including negative regulation of cell growth, regulation of cell growth and negative regulation of growth, and muscle contraction subnetwork were also observed. In addition, a Wnt receptor signalling pathway subnetwork, as well as gluconeogenesis and glycogen metabolic process subnetworks, were identified. The DEGs belonging to these regulatory subnetworks probably also play important roles in regulation gene expression in the longissimus muscle tissue of QHMM and STH sheep. Research into their detailed roles will be carried out in the future.

Similar to the GO analysis, pathway analysis was performed to further investigate the DEGs from another perspective. We found that the DEGs were mainly associated with carbohydrate metabolism, lipid metabolism, amino acid metabolism, signal transduction, and cell growth and apoptosis. We selected 23 significantly enriched pathways to establish a pathway-act-network. We found that the metabolic pathways, citrate cycle (TCA cycle) and alanine, aspartate and glutamate metabolism were the core pathways in the network. Skeletal muscle is the major metabolic tissue^34^; therefore, it was not surprising that metabolic pathways, which included 97 DEGs, comprised the most central pathway in the network in our study. Previously, it was showed that the TCA cycle is involved in skeletal muscle fibre transition^35^,^36^. In this study, seven DEGs from the TCA cycle were identified. In addition, we also obtained other essential pathways, including the glycolysis/gluconeogenesis pathway, the FoxO signalling pathway, the Wnt signalling pathway and the fatty acid elongation pathway. Among these pathways, glycolysis is a vital pathway during the post-mortem period, during which period the glycolysis rate can be influenced by muscle fibre type^37^,^38^. In our study, eight DEGs associated with glycolysis/gluconeogenesis pathway were identified. The Wnt signalling pathway plays an essential role in muscle growth and regeneration^39^, and a total of nineteen DEGs were identified in this research. Genetic studies have confirmed the function of several Wnt regulatory factors in skeletal muscle growth^40^, and the classical Wnt signalling pathway can induce the proliferation of satellite cells in the process of skeletal muscle regeneration process^41^. The FoxO signalling pathway plays a role in the regulation of skeletal muscle type differentiation^42^. We identified a total of nineteen FoxO signalling-related DEGs, among these DEGs, the *FoxO1* gene negatively regulates the II type muscle fibre by greatly increasing the expression level of the MyoD gene^43^,^44^. The DEGs, which were identified from the pathways mentioned above, are shown in Supplementary Table S10. These DEGs might be key genes; therefore, these pathways and DEGs should be investigated in detail for their association with the regulation of muscle growth and development.

After the GO and pathway analyses, we attempted to find the interactions between DEGs using gene-act-network and co-expression analysis. In the gene-act-network, we observed that *MYH6, MYL2, MYL6B, MYLK3, ITGA7, GPX1* and *PPP3CB* (Table 3) were also in the network and these DEGs were up-regulated in QHMM. Moreover, we found that *MYL2* and *MYL6B* were activated by *MYLK3*, and that *MYH6* and *MYL2* had a binding interaction. *PRKACA* and *PAK1* were associated with *MYLK3. ITGA7* was activated by *SPP1* and *COL11A2* and bound with *ACTN2* and *PIK3R2*. *GPX1*was compounded by *MGST3*. *PPP3CB* activated *PIK3R2*, *CDC26* and *NFATC1*. Based on these subnetworks, we are in a good position to investigate the signal transduction processes involving these DEGs and identify more key genes. Further research into the molecular regulation mechanism will be performed using the gene-act-network. Co-expression networks of DEGs are increasingly used to explore the system-level functionality of genes^45^. Based on the k-core value, we identified several core regulatory genes through the gene co-expression-network analysis. Interestingly, we also identified *MYH6, MYL2, MYL6B, MYLK3, NEO1*, *OMYHCS, PPP3CB* and *GPX1*, which are mentioned in Table 3 and are also in the co-expression-network. From the subnetworks, we identified many co-expressed DEGs. For example, in the QHMM co-expression-network, *MYH6* was positively co-expressed with *ALDH2, CACNB1, LOC100037702* and *LOC101121811*; *MYL6B* was positively co-expressed with *BDH1*and *INSR*; and *NEO1* was positively co-expressed with *MYH7B, MYL2, PRKAG3, PTPLA* and negatively co-expressed with *LOC101107037* and *LOC443301*. There are many similar subnetworks in co-expression network. Cells, as ordered units, play important roles in the organization of organs, as well living organisms, through the interaction of many elements (DNA, RNA, protein and small molecules). In a cell, genes that are associated with the same trait tend to have correlated expression patterns, including positive correlation and negative correlation^46^. Therefore, the results of this study will allow us to predict the function of new genes and to explore candidate genes that might play a role in muscle growth and development process in sheep.

Finally, we validated the RNA-seq results using qRT-PCR to measure the expression of nine DEGs (*CBS, CCND, HMOX1, ZBTB16, MGST3, SCARB1, SMAD3, NMNAT3* and *SDHC*), which showed that our findings were reliable. We also found that the DEG *HMOX1* was very highly up-regulated in STH sheep in both qRT-PCR and RNA-seq analyses. Previously, *HMOX1* was shown to be involved in the regulation of disease^47^,^48^,^49^ and cardiorespiratory function^50^, but few reports have described its role in muscle growth and development. The GO analysis of our present study identified *HMOX1* as a negative regulator of smooth muscle cell proliferation and cell proliferation. However, more in-depth study of *HMOX1* is required to clarify its functions in muscle growth and development.

## Conclusion

In summary, we established the transcriptome profiles of the longissimus muscle from two sheep breeds (QHMM and STH, which have different meat performance) using RNA-Seq. Subsequent bioinformatic analyses suggested that some DEGS, such as *MRFs, GXP1* and *STAC*, and pathways such as metabolic pathways, the TCA cycle, and the glycolysis/gluconeogenesis pathway, are indispensable for the process of muscle growth and development. This genome-wide transcriptome analysis of QHMM and STH muscle is reported for the first time, and suggests a role for transcriptome analysis in promoting muscle growth and meat performance in sheep. We also present the gene-act-network and co-expression network closely related to muscle growth and development. However, our study is limited by the fact that functional verification is difficult to perform in sheep because no sheep knockout phenotypes have been established thus far. We will attempt to establish such knockout phenotypes in a further study.

## Materials and Methods

### Ethics statement

All methods were carried out in accordance with relevant guidelines set by the Ministry of Agriculture of the People's Republic of China. All experimental protocols were approved by the Jilin Laboratory Animal Specialized Committee.

### Animal sample preparation

QHMM sheep and STH sheep were obtained from Jilin Qian’an Zhihua Sheep Breeding Co. Ltd (Qian’an, China). All the experimental sheep were raised under the same environment with natural light and free access to food and water. Thirty adult individuals (females, aged 1 year) within each breed were randomly selected for this study; these animals were sacrificed for carcass and meat quality analyses. Three animals within each breed were selected to obtain the longissimus dorsi muscles samples, and all the samples were immediately snap-frozen in liquid nitrogen for total RNA extraction.

### Carcass and meat quality analyses

The animal’s live weight after fasting for 24 h was measured before slaughter. After slaughter, traits including the carcass weight, loin eye muscle area, slaughter percentage and net meat percentage were measured and calculated. The longissimus muscle was removed to determine the meat quality. Muscle shear force was measured using a C-LM3B muscle shear force measuring instrument (Tenovo, China). The water loss rate and cooking percentage were defined by pressure and cooking methods. Muscle pH was measured at 45 min and 24 h post mortem in the longissimus muscle (between the 12th and 13th rib) using a PB-10 portable pH-meter.

### Construction of the mRNA library and sequencing

Total RNA was extracted from each muscle tissue sample using the TRIzol Reagent (Life technologies, USA), according to the manufacturer’s instructions. The concentration and quality of RNA were measured using NanoDrop 2000 (Thermo scientific, USA) and Agilent 2200 (Agilent, USA) instruments. The sequencing library of each RNA sample was prepared using an Ion Total RNA-seq Kit v2 (Life technologies, USA), according to the manufacturer’s instructions. Briefly, poly(A)-containing mRNA was purified using Dynabeads (Life technologies, USA) and fractionated into short fragments using RNase III and an Ion adaptor. The RNA fragments were reverse-transcribed and amplified to form double-stranded cDNA. Emulsion PCR was performed using the cDNA library as the template. RNA-Seq was conducted on an ABI Ion Proton instrument by NovelBio Bio-Pharm Technology Co. Ltd (Shanghai, China).

### MRNA expression data analysis

The raw RNA-seq reads were filtered and the clean reads were mapped to the Oar 3.1version of the sheep genome sequence. Only the unique mapped reads were used for gene expression analysis. The RPKM value was used to calculate gene expression, and the upper-quartile algorithm was used to correct the gene expression, which could produce accurate results for some genes in low abundance. To identify DEGs, the EBSeq algorithm was used and FDR was calculated to correct the *P*-value. If the |log_2_^FoldChange^| was >0.585 and FDR was < 0.05, then these genes were considered as differentially expressed.

### GO and KEGG pathway analyses

GO analysis was used to analyse the main function of the DEGs according to their Gene Ontology (http://geneontology.org/), which is the key functional classification at NCBI^51^ (http://www.ncbi.nlm.nih.gov/). The DEGs were annotated from the three main categories of biological process, molecular function and cellular component. Generally, Fisher’s exact test and the χ2 test were used to classify the GO categories, the *P*-value was computed for the GO terms. The significant GO terms of DEGs were defined as having a *P* -value <0.05.

Pathway analysis was used to identify the significant pathway involving the DEGs by pathway annotations using KEGG^52^ (http://www.genome.jp/kegg/). Fisher’s exact test was used to find the significant enrichment pathway. The significantly enriched pathways for DEGs were defined as having a *P* -value <0.05. To further reveal the interaction among the significant enriched pathways based on the KEGG database (including the metabolism, membrane transport, signal transduction, and cell cycle pathways), a pathway-act-network was constructed using the Cytoscape software to establish a graphical representation of the pathway^53^.

### Gene-act-network and co-expression-network analysis

The gene-act-network, which reflected the relationship between DEGs, was built using Cytoscape software^53^, according to the connections among the genes, proteins and com,pounds from the KEGG database^54^,^55^. The co-expression-network was constructed according to the normalized signal intensity of DEGs that were selected from significant GO terms and pathways. For each pair of genes, Pearson’s correlation coefficient was calculated and significant correlated pairs (FDR<0.05) were chosen to establish the network^56^. Within the co-expression-network, to locate the key regulatory genes, k-cores were introduced to simplify the graph topology analysis. A k-core of a network is a subnetwork in which all nodes are connected to at least k other genes in the subnetwork^57^,^58^. The greater the value of k-core, the stronger the co-expression of the DEGs^59^.

### Validation of RNA-Seq data by qRT-PCR

To validate the reliability of the RNA-Seq data, qRT-PCR was conducted. Nine DEGs (*CBS, CCND, HMOX1, ZBTB16, MGST3, SCARB1, SMAD3, NMNAT3*, and *SDHC*) were randomly selected. First, total RNA was extracted from each muscle sample using the TRIzol Reagent (Life technologies, USA). CDNA was then synthesized using a ReverTra Ace qPCR RT Kit (FSQ-101, TOYOBO, Japan) from 1 μg of the same total RNA samples; β-actin gene was used as reference house-keeping gene, SYBR Green Realtime PCR Master Mix (QPK-201, TOYOBO, Japan) was used to perform the qPCR reactions in a Bio-Rad CFX96 system, with a 20-μL reaction system comprising 10 μL of SYBR Green Realtime PCR Master Mix, 0.8 μL of each of the forward and reverse primers (200 μM), 2 μL of cDNA and 6.4 μL of distilled water. The qRT-PCR program was 95 °C for 60 s; followed by 40 cycles of 95 °C for 15 s, 60 °C for 15 s, and 72 °C for 45 s; and ended with a final stage of melting curve analysis.

The descriptions of genes, which were mentioned above, were shown in Supplementary Table S11.

## Additional Information

Accession codes: The raw sequence and processed data have been submitted to the Gene Expression Omnibus database (GEO dataset). The accession number is GSE84964.

**How to cite this article**: Sun, L. *et al*. Comparative transcriptome profiling of longissimus muscle tissues from Qianhua Mutton Merino and Small Tail Han sheep. *Sci. Rep*. **6**, 33586; doi: 10.1038/srep33586 (2016).

## Supplementary Material

Supplementary Information

## Figures and Tables

**Table 1:** Carcass and meat quality traits in the QHMM and STH.

Traits	Definitions of traits	QHMM (n = 30)	STH (n = 30)
Live weight (Kg)	Live weight before slaughter (fasting 24h)	55.33 ± 1.36^*^	45.15 ± 4.15
Carcass weight (Kg)	Body weight removing the head, tail, limbs, internal organs and other parts of the weight after slaughter	27.38 ± 0.84^**^	17.27 ± 0.84
Slaughter percentage (%)	Carcass weight/Live weight ratio	50.08 ± 1.73^**^	38.33 ± 1.92
Net meat percentage (%)	Net meat weight/ live weight ratio	39.40 ± 1.76^*^	31.35 ± 1.88
Loin eye muscle area (cm^2^)	Area of longissimus dorsiat 12th/13th rib	18.85 ± 1.52^*^	15.39 ± 1.05
Tenderness (N)	Shear force of longissimus dorsiat	32.08 ± 1.34	47.77 ± 1.52^**^
Water loss rate (%)	% of weight loss of longissimus dorsiat determinated by pressure method	6.23 ± 1.06	14.11 ± 0.93^**^
Cooking percentage (%)	longissimus dorsiat determinated by cooking / longissimus dorsiat weight ratio	62.38 ± 3.05^**^	45.03 ± 1.35
pH_1_	pH value in longissimus dorsiat 45 min post mortem	6.13 ± 0.09	6.23 ± 0.07
pH_24_	pH value in longissimus dorsiat 24 h post mortem	5.44 ± 0.14	5.72 ± 0.04^*^

**P < 0.01 and *P < 0.05.

**Table 2:** Reads mapping summary.

Statistics	QHMM A1	QHMM A2	QHMM A3	STH B1	STH B2	STH B3
All reads	18,752,596	17,482,106	16,488,130	17,458,226	14,790,361	17,408,297
Un Mapped reads	1,415,576	1,531,813	1,302,393	1,334,282	1,156,221	1,371,310
Mapped reads	17,337,020	15,950,293	15,185,737	16,123,944	13,634,140	16,036,987
Mapped Rate	0.925	0.912	0.921	0.924	0.922	0.921
Unique Mapped reads	16,437,180	14,905,935	14,372,858	15,292,633	12,779,250	15,237,071
Unique Mapped Rate	0.877	0.853	0.872	0.876	0.864	0.875
Repeat Mapped reads	899,853	1,044,371	812,891	831,323	854,906	799,930
Junction All Mapped reads	7,829,834	6,856,974	6,700,137	6,846,232	5,638,984	7,226,780
Junction Unique Mapped reads	7,826,739	6,854,001	6,697,036	6,843,592	5,636,750	7,223,760

**Table 3:** Differential mRNAs associated with muscle growth and development process by GO analysis.

Gene symbol	Description	QHMM (normalized counts)	STH (normalized counts)	Log2^FoldChange^	FDR	Style (STH vs.QHMM)	Enriched biological process
BCL9L	B-cell CLL/lymphoma 9-like protein	364.4	156.2	−1.2	5.16E-03	down	7
BTG1	Protein BTG1	670.5	3228.5	2.3	9.08E-05	up	4,10
CASQ1	Calsequestrin	45168.0	17353.3	−1.4	5.41E-04	down	1,8
CASQ2	Calsequestrin	620.4	248.1	−1.3	1.90E-02	down	8
CDH15	Cadherin-15	122.5	263.7	1.1	1.66E-03	up	5
CITED2	Cbp/p300-interacting transactivator with Glu/Asp-rich carboxy-terminal domain 2	155.9	289.7	0.9	2.54E-02	up	7
CSRP3	cDNA, FLJ93801, Homo sapiens cysteine and glycine-rich protein 3 (cardiac LIMprotein) (CSRP3), mRNA	4896.3	18425.4	2.0	4.61E-02	up	1,9
FHOD3	FH1/FH2 domain-containing protein 3	502.7	172.0	−1.5	0	down	8,9
FOXP1	cDNA FLJ58267, highly similar to Forkhead box protein P1	233.3	382.1	0.7	2.45E-02	up	1,8
GPX1	Glutathione peroxidase	1529.1	761.9	−1.0	1.22E-06	down	3
HEYL	cDNA FLJ52278, highly similar to Homo sapiens hairy/enhancer-of-split related with YRPW motif-like (HEYL), mRNA	310.0	186.4	−0.7	7.27E-05	down	7
HLF	Hepatic leukemia factor	76.3	31.7	−1.3	1.02E-04	down	7
ITGA7	cDNA FLJ12486 fis, clone NT2RM2000566, highly similar to Integrin alpha-7	4307.8	1157.3	−1.9	0	down	1
KIAA1161	KIAA1161 ortholog	661.6	214.7	−1.6	1.19E-06	down	3
LMOD2	cDNA FLJ50049, highly similar to Mus musculus leiomodin 2 (cardiac) (Lmod2), mRNA	5623.4	12507.3	1.2	1.18E-08	up	9
MAFF	Transcription factor MafF	73.1	239.3	1.7	1.89E-03	up	7
MYF6	Myogenic factor 6	1688.6	4474.1	1.4	0	up	1,2,4,6,7,8,10
MYH6	myosin, heavy chain 6, cardiac muscle, alpha	12787.7	4502.7	−1.5	1.17E-10	down	8,9
MYL2	Myosin light chain 2	63933.5	23309.3	−1.5	7.13E-12	down	9
MYL6B	myosin, light chain 6B, alkali, smooth muscle and non-muscle	12018.3	1654.6	−2.9	3.77E-02	down	1
MYLK3	Myosin light chain kinase 3	265.8	94.1	−1.5	6.03E-06	down	8,9
MYOD1	Myoblast determination protein 1	232.5	37.5	−2.6	3.46E-06	down	1,2,3,4,6,7,8,10
MYOG	Myogenin	501.8	148.6	−1.8	2.41E-04	down	1,2,3,4,6,7,8,10
NEO1	Neogenin homolog 1 (Chicken), isoform CRA_a	390.1	178.1	−1.1	2.90E-06	down	5
OMYHCS	Myosin heavy chain slow	225230.0	88659.7	−1.3	3.66E-14	down	8,9
PPP3CB	Serine/threonine-protein phosphatase	1978.0	1122.0	−0.8	3.85E-02	down	3
RXRG	Retinoic acid receptor RXR-gamma	1104.1	273.7	−2.0	2.89E-06	down	1
SETD3	SET domain containing 3, isoform CRA_a	1297.1	719.0	−0.9	1.02E-04	down	5
STAC3	SH3 and cysteine rich domain 3, isoform CRA_a	5060.1	2569.4	−1.0	4.65E-03	down	3
XIRP1	xin actin-binding repeat containing 1	7518.7	37631.7	2.3	1.11E-06	up	8

Enriched biological process: 1. skeletal muscle tissue development; 2. positive regulation of skeletal muscle fiber development; 3. skeletal muscle fiber development; 4. positive regulation of myoblast differentiation; 5. positive regulation of muscle cell differentiation; 6. muscle cell fate commitment; 7. muscle cell differentiation; 8. sarcomere organization; 9. myofibril assemly; 10. myoblast differentiation.

## References

[b1] Hobert O. Gene regulation by transcription factors and microRNAs. Science 319, 1785–1786, doi: 10.1126/science.1151651 (2008). 18369135

[b2] Chen K. & Rajewsky N. The evolution of gene regulation by transcription factors and microRNAs. Nat Rev Genet 8, 93–103, doi: 10.1038/nrg1990 (2007). 17230196

[b3] Levine M. & Tjian R. Transcription regulation and animal diversity. Nature 424, 147–151, doi: 10.1038/nature01763 (2003). 12853946

[b4] Costa V., Angelini C., De Feis I. & Ciccodicola A. Uncovering the complexity of transcriptomes with RNA-Seq. J Biomed Biotechnol 2010, 853916, doi: 10.1155/2010/853916 (2010). 20625424 PMC2896904

[b5] Bryson-Richardson R. J. & Currie P. D. The genetics of vertebrate myogenesis. Nat Rev Genet 9, 632–646, doi: 10.1038/nrg2369 (2008). 18636072

[b6] Gent J., Van Den Eijnden M., Van Kerkhof P. & Strous G. J. Dimerization and signal transduction of the growth hormone receptor. Mol Endocrinol 17, 967–975, doi: 10.1210/me.2002-0261 (2003). 12576487

[b7] Duan C., Ren H. & Gao S. Insulin-like growth factors (IGFs), IGF receptors, and IGF-binding proteins: roles in skeletal muscle growth and differentiation. Gen Comp Endocrinol 167, 344–351, doi: 10.1016/j.ygcen.2010.04.009 (2010). 20403355

[b8] Mavalli M. D. . Distinct growth hormone receptor signaling modes regulate skeletal muscle development and insulin sensitivity in mice. J Clin Invest 120, 4007–4020, doi: 10.1172/JCI42447 (2010). 20921627 PMC2964973

[b9] Langley B. . Myostatin inhibits myoblast differentiation by down-regulating MyoD expression. J Biol Chem 277, 49831–49840, doi: 10.1074/jbc.M204291200 (2002). 12244043

[b10] Abe S. . Expression of myostatin and follistatin in Mdx mice, an animal model for muscular dystrophy. Zoolog Sci 26, 315–320, doi: 10.2108/zsj.26.315 (2009). 19715499

[b11] Mardis E. R. The impact of next-generation sequencing technology on genetics. Trends Genet 24, 133–141, doi: 10.1016/j.tig.2007.12.007 (2008). 18262675 PMC2680276

[b12] Mutz K. O., Heilkenbrinker A., Lonne M., Walter J. G. & Stahl F. Transcriptome analysis using next-generation sequencing. Curr Opin Biotechnol 24, 22–30, doi: 10.1016/j.copbio.2012.09.004 (2013). 23020966

[b13] Cai Z. . Transcriptomic analysis of hepatic responses to testosterone deficiency in miniature pigs fed a high-cholesterol diet. BMC Genomics 16, 59, doi: 10.1186/s12864-015-1283-0 (2015). 25887406 PMC4328429

[b14] Gao Y. . ACTN4 and the pathways associated with cell motility and adhesion contribute to the process of lung cancer metastasis to the brain. BMC Cancer 15, 277, doi: 10.1186/s12885-015-1295-9 (2015). 25885339 PMC4409712

[b15] Ouyang Y., Pan J., Tai Q., Ju J. & Wang H. Transcriptomic changes associated with DKK4 overexpression in pancreatic cancer cells detected by RNA-Seq. Tumour Biol, doi: 10.1007/s13277-015-4379-x (2016). 26880586

[b16] Xulvi-Brunet R. & Li H. Co-expression networks: graph properties and topological comparisons. Bioinformatics 26, 205–214, doi: 10.1093/bioinformatics/btp632 (2010). 19910304 PMC2804297

[b17] Livak K. J. & Schmittgen T. D. Analysis of relative gene expression data using real-time quantitative PCR and the 2(-Delta Delta C(T)) Method. Methods 25, 402–408, doi: 10.1006/meth.2001.1262 (2001). 11846609

[b18] Ayuso M. . Comparative Analysis of Muscle Transcriptome between Pig Genotypes Identifies Genes and Regulatory Mechanisms Associated to Growth, Fatness and Metabolism. PLoS One 10, e0145162, doi: 10.1371/journal.pone.0145162 (2015). 26695515 PMC4687939

[b19] Myers S. A., Wang S. C. & Muscat G. E. The chicken ovalbumin upstream promoter-transcription factors modulate genes and pathways involved in skeletal muscle cell metabolism. J Biol Chem 281, 24149–24160, doi: 10.1074/jbc.M601941200 (2006). 16803904

[b20] Wickramasinghe S., Cánovas A., Rincón G. & Medrano J. F. RNA-Sequencing: A tool to explore new frontiers in animal genetics. Livestock Science 166, 206–216, doi: 10.1016/j.livsci.2014.06.015 (2014).

[b21] Miao X., Luo Q. & Qin X. Genome-wide analysis reveals the differential regulations of mRNAs and miRNAs in Dorset and Small Tail Han sheep muscles. Gene 562, 188–196, doi: 10.1016/j.gene.2015.02.070 (2015). 25732516

[b22] Wade C. . Characterization and Comparative Analyses of Muscle Transcriptomes in Dorper and Small-Tailed Han Sheep Using RNA-Seq Technique. PLoS ONE 8, e72686, doi: 10.1371/journal.pone.0072686 (2013). 24023632 PMC3758325

[b23] Ropka-Molik K., Eckert R. & Piorkowska K. The expression pattern of myogenic regulatory factors MyoD, Myf6 and Pax7 in postnatal porcine skeletal muscles. Gene Expr Patterns 11, 79–83, doi: 10.1016/j.gep.2010.09.005 (2011). 20888930

[b24] Yin H. . Myogenic regulatory factor (MRF) expression is affected by exercise in postnatal chicken skeletal muscles. Gene 561, 292–299, doi: 10.1016/j.gene.2015.02.044 (2015). 25701607

[b25] Charge S. B. & Rudnicki M. A. Cellular and molecular regulation of muscle regeneration. Physiol Rev 84, 209–238, doi: 10.1152/physrev.00019.2003 (2004). 14715915

[b26] Buckingham M. & Vincent S. D. Distinct and dynamic myogenic populations in the vertebrate embryo. Curr Opin Genet Dev 19, 444–453, doi: 10.1016/j.gde.2009.08.001 (2009). 19762225

[b27] Dedkov E. I., Kostrominova T. Y., Borisov A. B. & Carlson B. M. MyoD and myogenin protein expression in skeletal muscles of senile rats. Cell Tissue Res 311, 401–416, doi: 10.1007/s00441-002-0686-9 (2003). 12658448

[b28] SZ Z. . The possible role of myosin light chain in myoblast proliferation. Biological Research 42, 121–132, doi: /S0716-97602009000100013 (2009). 19621140

[b29] Lee S. . Glutathione-peroxidase-1 null muscle progenitor cells are globally defective. Free Radic Biol Med 41, 1174–1184, doi: 10.1016/j.freeradbiomed.2006.07.005 (2006). 16962942

[b30] Bower N. I. . Stac3 is required for myotube formation and myogenic differentiation in vertebrate skeletal muscle. J Biol Chem 287, 43936–43949, doi: 10.1074/jbc.M112.361311 (2012). 23076145 PMC3527977

[b31] Liu G. . Molecular cloning, characterization and tissue specificity of the expression of the ovine CSRP2 and CSRP3 genes from Small-tail Han sheep (Ovis aries). Gene 580, 47–57, doi: 10.1016/j.gene.2016.01.021 (2016). 26779824

[b32] Xu X. . Porcine CSRP3: polymorphism and association analyses with meat quality traits and comparative analyses with CSRP1 and CSRP2. Mol Biol Rep 37, 451–459, doi: 10.1007/s11033-009-9632-1 (2010). 19634002

[b33] Kamaid A. & Giraldez F. Btg1 and Btg2 gene expression during early chick development. Dev Dyn 237, 2158–2169, doi: 10.1002/dvdy.21616 (2008). 18651656

[b34] Li X. J., Zhou J., Liu L. Q., Qian K. & Wang C. L. Identification of genes in longissimus dorsi muscle differentially expressed between Wannanhua and Yorkshire pigs using RNA-sequencing. Anim Genet 47, 324–333, doi: 10.1111/age.12421 (2016). 27038141

[b35] Khan M. . Niacin supplementation induces type II to type I muscle fiber transition in skeletal muscle of sheep. Acta Veterinaria Scandinavica 55, 506–506, doi: 10.1186/1751-0147-55-85 (2013). PMC4176759 24267720

[b36] Khan M. . Niacin supplementation increases the number of oxidative type I fibers in skeletal muscle of growing pigs. Bmc Veterinary Research 9, 252–252., doi: 10.1186/1746-6148-9-177 (2013). 24010567 PMC3846775

[b37] Choi Y. M. & Kim B. C. Muscle fiber characteristics, myofibrillar protein isoforms, and meat quality. Livestock Science 122, 105–118, doi: 10.1016/j.livsci.2008.08.015 (2009).

[b38] Choe J. H. . The relation between glycogen, lactate content and muscle fiber type composition, and their influence on postmortem glycolytic rate and pork quality. Meat Sci 80, 355–362, doi: 10.1016/j.meatsci.2007.12.019 (2008). 22063340

[b39] Kubota Takuo & Toshimi Michigami & Ozono., K. Wnt signaling in bone and muscle. Bone 80, 60–66, doi: 10.1016/j.bone.2015.02.009 (2015). 26453496 PMC4600531

[b40] van Amerongen R. & Berns A. Knockout mouse models to study Wnt signal transduction. Trends Genet 22, 678–689, doi: 10.1016/j.tig.2006.10.001 (2006). 17045694

[b41] Otto A. . Canonical Wnt signalling induces satellite-cell proliferation during adult skeletal muscle regeneration. J Cell Sci 121, 2939–2950, doi: 10.1242/jcs.026534 (2008). 18697834

[b42] Yuan Y., Shi X. E., Liu Y. G. & Yang G. S. FoxO1 regulates muscle fiber-type specification and inhibits calcineurin signaling during C2C12 myoblast differentiation. Mol Cell Biochem 348, 77–87, doi: 10.1007/s11010-010-0640-1 (2011). 21080037

[b43] Kitamura T. . A Foxo/Notch pathway controls myogenic differentiation and fiber type specification. J Clin Invest 117, 2477–2485, doi: 10.1172/JCI32054 (2007). 17717603 PMC1950461

[b44] Liu C. M. . Effect of RNA oligonucleotide targeting Foxo-1 on muscle growth in normal and cancer cachexia mice. Cancer Gene Ther 14, 945–952, doi: 10.1038/sj.cgt.7701091 (2007). 17885675

[b45] Zhang B. & Horvath S. A general framework for weighted gene co-expression network analysis. Stat Appl Genet Mol Biol 4, Article17, doi: 10.2202/1544-6115.1128 (2005). 16646834

[b46] Arkin A. P. & Schaffer D. V. Network news: innovations in 21st century systems biology. Cell 144, 844–849, doi: 10.1016/j.cell.2011.03.008 (2011). 21414475

[b47] Zagorski J., Marchick M. R. & Kline J. A. Rapid clearance of circulating haptoglobin from plasma during acute pulmonary embolism in rats results in HMOX1 up-regulation in peripheral blood leukocytes. J Thromb Haemost 8, 389–396, doi: 10.1111/j.1538-7836.2009.03704.x (2010). 19943874

[b48] Yim M. S. . HMOX1 is an important prognostic indicator of nonmuscle invasive bladder cancer recurrence and progression. J Urol 185, 701–705, doi: 10.1016/j.juro.2010.09.081 (2011). 21168882

[b49] Vasavda N. . The linear effects of alpha-thalassaemia, the UGT1A1 and HMOX1 polymorphisms on cholelithiasis in sickle cell disease. Br J Haematol 138, 263-270, doi:10.1111/j.1365-2141.2007.06643.x (2007). 10.1111/j.1365-2141.2007.06643.x 17593033

[b50] Llanos A. J. . The heme oxygenase-carbon monoxide system in the regulation of cardiorespiratory function at high altitude. Respir Physiol Neurobiol 184, 186–191, doi: 10.1016/j.resp.2012.05.003 (2012). 22595369

[b51] Ashburner M. . Gene ontology: tool for the unification of biology. The Gene Ontology Consortium. Nat Genet 25, 25–29, doi: 10.1038/75556 (2000). 10802651 PMC3037419

[b52] Draghici S. . A systems biology approach for pathway level analysis. Genome Res 17, 1537–1545, doi: 10.1101/gr.6202607 (2007). 17785539 PMC1987343

[b53] P S. . Cytoscape: A Software Environment for Integrated Models of Biomolecular Interaction Networks. Genome Res 13, 2498–2504, doi: 10.1101/gr.1239303. (2003). 14597658 PMC403769

[b54] Binder H. & Schumacher M. Comment on ‘Network-constrained regularization and variable selection for analysis of genomic data’. Bioinformatics 24, 2566–2568; author reply 2569, doi: 10.1093/bioinformatics/btn412 (2008). 18682424

[b55] Wang M. . LegumeGRN: a gene regulatory network prediction server for functional and comparative studies. PLoS One 8, e67434, doi: 10.1371/journal.pone.0067434 (2013). 23844010 PMC3701055

[b56] Prieto C., Risueno A., Fontanillo C. & De las Rivas J. Human gene coexpression landscape: confident network derived from tissue transcriptomic profiles. PLoS One 3, e3911, doi: 10.1371/journal.pone.0003911 (2008). 19081792 PMC2597745

[b57] Ravasz E., Somera A. L., Mongru D. A., Oltvai Z. N. & Barabási A. L. Hierarchical organization of modularity in metabolic networks. Science 297, 1551–1555 (2002). 12202830 10.1126/science.1073374

[b58] Barabasi A. L. & Oltvai Z. N. Network biology: understanding the cell's functional organization. Nat Rev Genet 5, 101–113, doi: 10.1038/nrg1272 (2004). 14735121

[b59] Chen F. . Genes related to the very early stage of ConA-induced fulminant hepatitis: a gene-chip-based study in a mouse model. BMC Genomics 11, 240, doi: 10.1186/1471-2164-11-240 (2010). 20398290 PMC2867829

