# Supplementary material for: Comparative transcriptome profiling of longissimus muscle tissues from Qianhua Mutton Merino and Small Tail Han sheep
Source: Sci Rep. 2016 Sep 20;6:33586. doi: 10.1038/srep33586 (PMC5028831; doi:10.1038/srep33586)
Supplement: Supplementary Information [file srep33586-s1.pdf]

**Supplementary Information**

# Comparative transcriptome profiling of longissimus muscle tissues from Qianhua Mutton Merino and Small Tail Han sheep

Limin Sun, Man Bai, Lujie Xiang, Guishan Zhang, Wei Ma, Huaizhi Jiang*

College of Animal Science and Technology, Jilin Agricultural University, Changchun 130118, China

*Corresponding author.sheepandgoatjlau@sina.com

## Supplementary Figures

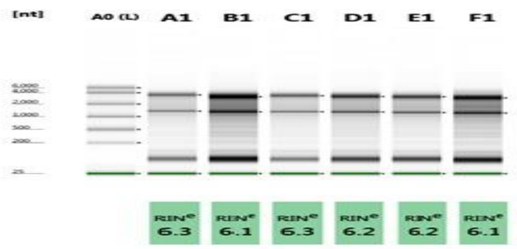


Figure S1. Gel Electrophoresis of extracted RNA samples

| Well | RIN$^e$ | 28S/18S (Height) | Vol [µl] | Conc. [ng/µl] | Sample Description |
|---|---|---|---|---|---|
| A0 | - | - | - | 137 | ladder |
| A1 | 6.3 | 1.2 | 35 | 223 | A-1 |
| B1 | 6.1 | 1.4 | 35 | 639 | A-2 |
| C1 | 6.3 | 1.2 | 35 | 230 | A-3 |
| D1 | 6.2 | 1.3 | 35 | 349 | B-1 |
| E1 | 6.2 | 1.3 | 35 | 319 | B-2 |
| F1 | 6.1 | 1.3 | 35 | 551 | B-3 |

Figure S2. RIN and other quality information of  RNA samples,

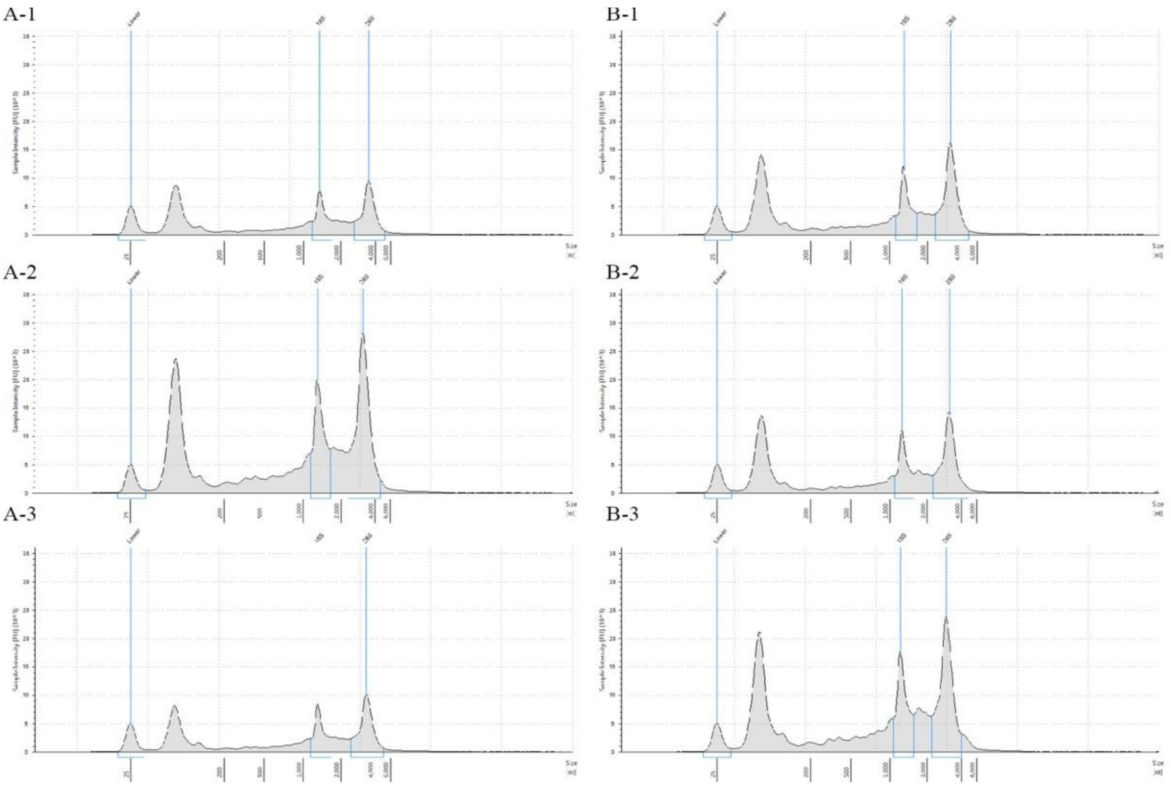


Figure S3. Agilent Bioanalyzer Electropherograms from six RNA samples

## Supplementary Tables

### Table S1: The DEGs between STH and QHMM (STH VS QHMM)

| Gene ID | STH normalized | QHMM normalized | Log2FC | FDR | Style | A1 | A2 | A3 | B1 | B2 | B3 | Symbol | Strand | Description | E-Value | Blast_Symbol |
|---|---|---|---|---|---|---|---|---|---|---|---|---|---|---|---|---|
| UGP2 | 3640.223483 | 1805.096661 | 1.011951 | 0 | up | 1606 | 1519 | 2180 | 4476 | 3404 | 3318 | UGP2 | - | Uncharacterized protein | 0 | UGP2 |
| LOC101121539 | 571.7000366 | 99.05771878 | 2.528917 | 0 | up | 51 | 105 | 127 | 584 | 533 | 614 | LOC101121539 | - | Uncharacterized protein | 0 | CYP2F1 |
| CYLD | 379.1613702 | 174.0174481 | 1.12358 | 0 | up | 193 | 130 | 196 | 489 | 328 | 353 | CYLD | + | Uncharacterized protein | 0 | CYLD |
| ABCC5 | 299.4701362 | 684.2239398 | -1.19206 | 0 | down | 758 | 606 | 664 | 331 | 367 | 220 | ABCC5 | + | Uncharacterized protein | 0 | ABCC5 |
| SLC6A1 | 30.43475776 | 218.3524692 | -2.84287 | 0 | down | 290 | 148 | 222 | 37 | 20 | 36 | SLC6A1 | + | Transporter | 0 | SLC6A1 |
| CEBPD | 2524.4366 | 291.3246856 | 3.115262 | 0 | up | 575 | 267 | 56 | 2840 | 2208 | 2647 | CEBPD | + | CCAAT/enhancer-binding protein delta | 4.00E-33 | CEBPD |
| SEMA6C | 745.7910362 | 3258.449276 | -2.12734 | 0 | down | 3603 | 2613 | 3477 | 1146 | 452 | 734 | SEMA6C | - | Uncharacterized protein | 0 | SEMA6C |
| FOXJ2 | 566.3529635 | 1094.048364 | -0.9499 | 0 | down | 1188 | 1009 | 1037 | 792 | 540 | 433 | FOXJ2 | + | Uncharacterized protein | 0 | FOXJ2 |
| SHISA2 | 128.7031369 | 751.533206 | -2.54579 | 0 | down | 714 | 671 | 825 | 204 | 96 | 105 | SHISA2 | + | shisa homolog 2 (Xenopus laevis) | 4.00E-80 | SHISA2 |
| PCDH12 | 76.06435721 | 317.2170135 | -2.06018 | 0 | down | 362 | 309 | 267 | 106 | 57 | 73 | PCDH12 | - | Uncharacterized protein | 0 | PCDH12 |
| GCAT | 260.287898 | 549.257811 | -1.07738 | 0 | down | 636 | 493 | 502 | 350 | 201 | 254 | GCAT | + | Uncharacterized protein | 0 | GCAT |
| GADL1 | 358.8395672 | 1945.97659 | -2.43908 | 0 | down | 2464 | 1530 | 1843 | 491 | 281 | 340 | GADL1 | - | Uncharacterized protein | 0 | GADL1 |
| CA12 | 123.5386991 | 8.762526325 | 3.817472 | 0 | up | 12 | 10 | 4 | 105 | 129 | 136 | CA12 | + | Uncharacterized protein | 8.00E-167 | CA12 |
| FGF1 | 85.72340353 | 229.1289204 | -1.4184 | 0 | down | 239 | 188 | 252 | 121 | 59 | 86 | FGF1 | - | Acidic fibroblast growth factor | | |
| GPX3 | 3812.124227 | 1709.056044 | 1.157395 | 0 | up | 1880 | 1632 | 1536 | 4582 | 3349 | 3758 | GPX3 | + | Glutathione peroxidase | 1.00E-109 | GPX3 |
| HSPB3 | 78.91417851 | 239.6412036 | -1.60252 | 0 | down | 251 | 214 | 243 | 112 | 38 | 94 | HSPB3 | - | Uncharacterized protein | 2.00E-73 | HSPB3 |
| STRIP2 | 1076.037138 | 204.7932456 | 2.393488 | 0 | up | 252 | 178 | 181 | 1243 | 1207 | 854 | STRIP2 | + | Uncharacterized protein | 0 | STRIP2 |
| JPH2 | 181.1826594 | 850.0819709 | -2.23016 | 0 | down | 982 | 740 | 805 | 212 | 59 | 277 | JPH2 | - | Uncharacterized protein | 6.00E-127 | JPH2 |
| METTL7B | 42.97468707 | 643.6362916 | -3.90469 | 0 | down | 781 | 444 | 709 | 83 | 5 | 49 | METTL7B | - | Uncharacterized protein | 3.00E-115 | METTL7B |
| CD93 | 281.1361818 | 631.4203166 | -1.16733 | 0 | down | 752 | 536 | 594 | 369 | 227 | 272 | CD93 | - | CD93 | 0 | CD93 |
| LOC101108390 | 607.7722565 | 968.3556932 | -0.67201 | 0 | down | 1114 | 850 | 913 | 726 | 509 | 626 | LOC101108390 | + | MORF4 family-associated protein 1-like | 5.00E-56 | MRFAP1 |
| METTL11B | 83.59778013 | 414.695235 | -2.31051 | 0 | down | 503 | 294 | 448 | 149 | 32 | 84 | METTL11B | + | Uncharacterized protein | 2.00E-158 | METTL11B |
| FABP5 | 889.5614609 | 257.4553527 | 1.788772 | 0 | up | 413 | 184 | 190 | 1080 | 731 | 916 | FABP5 | + | Fatty acid binding protein 5 | 3.00E-68 | FABP5 |
| CAP2 | 2291.448951 | 3717.821194 | -0.6982 | 0 | down | 3993 | 3184 | 3839 | 2953 | 1993 | 2125 | CAP2 | - | Adenylyl cyclase-associated protein | 0 | CAP2 |
| TMEM52 | 92.58959162 | 373.2283574 | -2.01114 | 0 | down | 397 | 360 | 343 | 112 | 48 | 122 | TMEM52 | + | transmembrane protein 52 | 7.00E-52 | TMEM52 |
| TRIM7 | 275.296897 | 1455.429455 | -2.40238 | 0 | down | 1697 | 1227 | 1410 | 458 | 152 | 258 | TRIM7 | - | Uncharacterized protein | 0 | TRIM7 |
| KCNMB4 | 200.8698289 | 44.17716088 | 2.184888 | 0 | up | 37 | 41 | 51 | 216 | 176 | 218 | KCNMB4 | - | Potassium large conductance calcium-activated channel subfamily M beta member 4 | 3.00E-122 | KCNMB4 |

| | | | | | | | | | | | | | | | | | | |
|---|---|---|---|---|---|---|---|---|---|---|---|---|---|---|---|---|---|---|
| FRZB | 84.55360789 | 636.2511152 | -2.91166 | 0 | down | 743 | 550 | 600 | 151 | 63 | 56 | FRZB | + | Uncharacterized protein | 1.00E-168 | FRZB |
| LOC101109139 | 68.55844648 | 0 | 20 | 0 | up | 0 | 0 | 0 | 7 | 32 | 152 | LOC101109139 | - | uncharacterized LOC101109139 | | |
| FAM46C | 17.11078796 | 80.40965179 | -2.23246 | 0 | down | 104 | 67 | 70 | 28 | 12 | 14 | FAM46C | + | family with sequence similarity 46, member C | 0 | FAM46C |
| LOC101112867 | 1708.156773 | 2733.784914 | -0.67846 | 0 | down | 3173 | 2423 | 2527 | 1956 | 1514 | 1746 | LOC101112867 | + | enoyl-CoA hydratase, mitochondrial-like | 9.00E-134 | ECHS1 |
| SOCS2 | 191.2094853 | 66.11010074 | 1.532211 | 0 | up | 49 | 70 | 72 | 224 | 201 | 162 | SOCS2 | + | Uncharacterized protein | 9.00E-110 | SOCS2 |
| LOC101110740 | 106.5025077 | 482.0123672 | -2.17818 | 0 | down | 526 | 446 | 453 | 219 | 51 | 75 | LOC101110740 | + | Uncharacterized protein | 0 | TLE1 |
| LOC101105484 | 55594.73484 | 30118.56823 | 0.884295 | 0 | up | 31568 | 29125 | 27998 | 61055 | 52796 | 55527 | LOC101105484 | + | ferritin heavy chain-like | 3.00E-96 | FTH1 |
| ARFGAP1 | 333.3076961 | 186.3014374 | 0.839216 | 0 | up | 214 | 167 | 172 | 355 | 334 | 325 | ARFGAP1 | - | Uncharacterized protein | 0 | ARFGAP1 |
| CHN2 | 17.22045185 | 110.3662506 | -2.6801 | 0 | down | 139 | 109 | 80 | 17 | 16 | 19 | CHN2 | - | Uncharacterized protein | 0 | CHN2 |
| LOC101110855 | 68.71877272 | 347.7506158 | -2.33928 | 0 | down | 457 | 239 | 354 | 115 | 73 | 31 | LOC101110855 | + | uncharacterized LOC101110855 | 2.00E-28 | SECTM1 |
| RELL1 | 284.9291581 | 107.8589271 | 1.401458 | 0 | up | 145 | 60 | 123 | 343 | 302 | 232 | RELL1 | - | Uncharacterized protein | 9.00E-126 | RELL1 |
| SOX6 | 258.4552866 | 448.8171255 | -0.79621 | 0 | down | 496 | 368 | 470 | 308 | 229 | 255 | SOX6 | + | Uncharacterized protein | 0 | SOX6 |
| CHI3L1 | 402.5227716 | 149.7991028 | 1.426041 | 0 | up | 185 | 85 | 183 | 462 | 415 | 356 | CHI3L1 | - | Chitinase-3-like protein 1 | 0 | CHI3L1 |
| ADAM19 | 79.96497322 | 234.6905085 | -1.55332 | 0 | down | 250 | 232 | 209 | 101 | 65 | 80 | ADAM19 | - | Uncharacterized protein | 0 | ADAM19 |
| MYOC | 212.1440262 | 576.9584545 | -1.44342 | 0 | down | 631 | 465 | 619 | 258 | 135 | 255 | MYOC | - | Uncharacterized protein | 0 | MYOC |
| MYF6 | 4474.066182 | 1688.584259 | 1.405772 | 0 | up | 2104 | 1389 | 1559 | 5740 | 3727 | 4323 | MYF6 | + | Myogenic factor 6 | 2.00E-132 | MYF6 |
| FHOD3 | 171.971848 | 502.6496308 | -1.54738 | 0 | down | 581 | 379 | 542 | 201 | 119 | 204 | FHOD3 | - | Uncharacterized protein | 0 | FHOD3 |
| TOE1 | 314.9378903 | 185.2671032 | 0.765461 | 0 | up | 220 | 143 | 191 | 344 | 314 | 302 | TOE1 | + | Uncharacterized protein | 0 | TOE1 |
| ITGA7 | 1157.258948 | 4307.754627 | -1.89622 | 0 | down | 4711 | 4233 | 3760 | 1708 | 696 | 1198 | ITGA7 | + | Uncharacterized protein | 0 | ITGA7 |
| SIMC1 | 182.0048891 | 82.66030818 | 1.138711 | 0 | up | 108 | 53 | 89 | 202 | 189 | 165 | SIMC1 | - | Uncharacterized protein | 0 | SIMC1 |
| LOC101117584 | 92.62031119 | 382.5750231 | -2.04634 | 0 | down | 409 | 330 | 394 | 110 | 38 | 133 | LOC101117584 | + | small EDRK-rich factor 1-like | 2.00E-22 | SERF1A |
| TRAF7 | 1106.694246 | 657.1394645 | 0.751985 | 0 | up | 732 | 622 | 589 | 1290 | 1051 | 1048 | TRAF7 | + | Uncharacterized protein | 0 | TRAF7 |
| ALAD | 987.415589 | 440.0070738 | 1.166131 | 0 | up | 360 | 451 | 467 | 1152 | 922 | 949 | ALAD | + | Delta-aminolevulinic acid dehydratase | 0 | ALAD |
| PPP1R3C | 434.6504844 | 7755.408986 | -4.15728 | 0 | down | 7246 | 7043 | 8484 | 1076 | 76 | 290 | PPP1R3C | - | Protein phosphatase 1 regulatory subunit 3 | 9.00E-170 | PPP1R3C |
| LOC101116336 | 174.6776366 | 1770.450862 | -3.34135 | 0 | down | 2203 | 1566 | 1513 | 168 | 123 | 233 | LOC101116336 | + | Uncharacterized protein | 0 | CES1 |
| CARNS1 | 51.79898779 | 312.9982853 | -2.59516 | 0 | down | 389 | 290 | 253 | 79 | 18 | 64 | CARNS1 | + | Uncharacterized protein | 0 | CARNS1 |
| SFMBT1 | 251.6076728 | 113.4698539 | 1.148867 | 0 | up | 107 | 98 | 129 | 330 | 239 | 210 | SFMBT1 | + | Uncharacterized protein | 0 | SFMBT1 |
| LOC101112291 | 0 | 83.66103559 | -20 | 0 | down | 285 | 0 | 0 | 0 | 0 | 0 | LOC101112291 | - | uncharacterized LOC101112291 | | |
| LOC101113965 | 178.5167279 | 522.925368 | -1.55055 | 0 | down | 589 | 420 | 548 | 293 | 148 | 124 | LOC101113965 | + | ubiquitin D-like | 6.00E-58 | UBD |
| LOC101112411 | 2349.119198 | 634.6033799 | 1.888193 | 0 | up | 392 | 856 | 550 | 2981 | 2120 | 2142 | LOC101112411 | - | interferon regulatory factor 4-like | 2.00E-87 | DUSP15 |
| POLR2D | 215.1294698 | 98.56416711 | 1.12607 | 0 | up | 115 | 87 | 91 | 244 | 233 | 182 | POLR2D | + | Uncharacterized protein | 5.00E-78 | POLR2D |

| | | | | | | | | | | | | | | | | |
|---|---|---|---|---|---|---|---|---|---|---|---|---|---|---|---|---|
| HMCN2 | 302.1854471 | 648.8035588 | -1.10235 | 0 | down | 703 | 573 | 645 | 312 | 270 | 333 | HMCN2 | - | Uncharacterized protein | 0 | HMCN2 |
| PHKG1 | 1553.971458 | 2342.423967 | -0.59204 | 0 | down | 2584 | 2137 | 2212 | 1688 | 1619 | 1432 | PHKG1 | - | Uncharacterized protein | 0 | PHKG1 |
| PDK4 | 37397.81011 | 1055.774358 | 5.14658 | 0 | up | 531 | 2181 | 162 | 41225 | 38416 | 34513 | PDK4 | - | Uncharacterized protein | 0 | PDK4 |
| TFRC | 471.3845222 | 2297.305391 | -2.28497 | 0 | down | 2330 | 1979 | 2477 | 695 | 138 | 625 | TFRC | - | Transferrin receptor | 0 | TFRC |
| DNMT3A | 378.97634 | 650.7281796 | -0.77995 | 0 | down | 761 | 534 | 645 | 430 | 363 | 365 | DNMT3A | - | DNA methyl transferase alpha | 0 | DNMT3A |
| LOC101104299 | 288.4837905 | 502.8181949 | -0.80155 | 0 | down | 551 | 464 | 472 | 328 | 282 | 272 | LOC101104299 | - | NADH dehydrogenase [ubiquinone] 1 alpha subcomplex subunit 4-like | 6.00E-36 | NDUFA4 |
| PBXIP1 | 888.0419325 | 1456.731125 | -0.71403 | 0 | down | 1758 | 1185 | 1409 | 1077 | 793 | 856 | PBXIP1 | - | pre-B-cell leukemia homeobox interacting protein 1 | 0 | PBXIP1 |
| STBD1 | 469.3845426 | 1862.748251 | -1.98859 | 0 | down | 1914 | 1649 | 1937 | 771 | 168 | 533 | STBD1 | + | starch binding domain 1 | 6.00E-123 | STBD1 |
| RASD1 | 5337.657515 | 544.6662402 | 3.292762 | 0 | up | 512 | 992 | 30 | 5550 | 4509 | 6096 | RASD1 | + | RASD1 | 1.00E-134 | RASD1 |
| FZD7 | 172.926849 | 567.105143 | -1.71345 | 0 | down | 585 | 523 | 564 | 241 | 121 | 174 | FZD7 | + | Uncharacterized protein | 1.00E-169 | FZD7 |
| CTSF | 1018.536555 | 555.8489408 | 0.873733 | 0 | up | 622 | 533 | 488 | 1315 | 941 | 891 | CTSF | - | Cathepsin F | 0 | CTSF |
| CTAGE5 | 81.81367856 | 169.2329265 | -1.0486 | 1.11E-16 | down | 200 | 130 | 176 | 93 | 94 | 64 | CTAGE5 | + | CTAGE family, member 5 | 0 | CTAGE5 |
| HR | 342.6766099 | 624.7869016 | -0.86652 | 2.22E-16 | down | 655 | 535 | 659 | 387 | 382 | 281 | HR | + | Uncharacterized protein | 0 | HR |
| HIVEP2 | 835.2565257 | 1528.477293 | -0.8718 | 2.22E-16 | down | 1646 | 1366 | 1510 | 1163 | 686 | 746 | HIVEP2 | - | Uncharacterized protein | 0 | HIVEP2 |
| C4H7orf53 | 46.03228957 | 176.3851396 | -1.93801 | 3.33E-16 | down | 174 | 162 | 183 | 85 | 24 | 38 | C4H7orf53 | - | Uncharacterized protein | 4.00E-50 | LSMEM1 |
| FKBP5 | 5918.499212 | 275.8260633 | 4.423401 | 4.44E-16 | up | 130 | 393 | 250 | 4648 | 7414 | 5652 | FKBP5 | - | Uncharacterized protein | 0 | FKBP5 |
| SH3KBP1 | 140.9560983 | 581.8201163 | -2.04533 | 4.44E-16 | down | 678 | 396 | 671 | 207 | 93 | 139 | SH3KBP1 | - | Uncharacterized protein | 0 | SH3KBP1 |
| SLC29A2 | 471.5254901 | 156.0209931 | 1.595596 | 4.44E-16 | up | 162 | 145 | 153 | 588 | 370 | 490 | SLC29A2 | - | Uncharacterized protein | 0 | SLC29A2 |
| ALS2CL | 676.244731 | 296.4541658 | 1.189736 | 6.66E-16 | up | 290 | 287 | 293 | 711 | 643 | 699 | ALS2CL | + | Uncharacterized protein | 0 | ALS2CL |
| PDE8B | 35.5548054 | 95.97567617 | -1.43262 | 9.99E-16 | down | 121 | 74 | 93 | 40 | 25 | 43 | PDE8B | + | Uncharacterized protein | 0 | PDE8B |
| LOC101112151 | 1724.713854 | 3674.738645 | -1.09128 | 1.11E-15 | down | 4581 | 2793 | 3651 | 1658 | 1897 | 1661 | LOC101112151 | + | uncharacterized LOC101112151 | 1.00E-67 | TNNI1 |
| IDH3A | 1454.473484 | 2965.801513 | -1.02793 | 1.89E-15 | down | 3147 | 2686 | 2930 | 2090 | 1117 | 1322 | IDH3A | + | Isocitrate dehydrogenase [NAD] subunit, mitochondrial | 0 | IDH3A |
| COG1 | 795.2088366 | 523.1967072 | 0.60398 | 2.11E-15 | up | 525 | 486 | 529 | 908 | 759 | 764 | COG1 | - | Uncharacterized protein | 0 | COG1 |
| RNF146 | 607.912583 | 250.6624642 | 1.278118 | 2.44E-15 | up | 236 | 177 | 330 | 679 | 646 | 534 | RNF146 | - | Uncharacterized protein | 0 | RNF146 |
| LOC101102253 | 48.75668093 | 186.1127376 | -1.9325 | 2.66E-15 | down | 175 | 174 | 197 | 82 | 28 | 44 | LOC101102253 | + | uncharacterized LOC101102253 | 2.00E-65 | FAM101B |
| EML1 | 377.1489602 | 1118.402749 | -1.56823 | 4.66E-15 | down | 1388 | 877 | 1086 | 410 | 212 | 517 | EML1 | + | Uncharacterized protein | 0 | EML1 |
| IGFR1 | 823.8780386 | 361.6607092 | 1.187794 | 4.66E-15 | up | 334 | 309 | 421 | 1062 | 839 | 649 | IGFR1 | + | Tyrosine-protein kinase receptor | 0 | IGF1R |
| EPB41 | 805.8569676 | 1381.936698 | -0.7781 | 5.33E-15 | down | 1487 | 1207 | 1398 | 1020 | 871 | 602 | EPB41 | - | Uncharacterized protein | 0 | EPB41 |
| NREP | 300.0748485 | 2455.342205 | -3.03253 | 6.77E-15 | down | 2130 | 2023 | 3061 | 272 | 190 | 433 | NREP | + | Uncharacterized protein | 6.00E-26 | NREP |
| HGS | 788.1457296 | 426.1031154 | 0.88726 | 1.18E-14 | up | 484 | 420 | 355 | 849 | 823 | 730 | HGS | - | Uncharacterized protein | 0 | HGS |
| MCM7 | 747.8541656 | 480.1407237 | 0.6393 | 1.18E-14 | up | 507 | 475 | 431 | 847 | 752 | 688 | MCM7 | + | DNA helicase | 0 | MCM7 |

| | | | | | | | | | | | | | | | | | |
|---|---|---|---|---|---|---|---|---|---|---|---|---|---|---|---|---|---|
| ALAS1 | 1180.381108 | 2440.355139 | -1.04784 | 1.24E-14 | down | 3027 | 2168 | 2083 | 1599 | 1019 | 1042 | ALAS1 | - | 5-aminolevulinate synthase | 0 | ALAS1 |
| ZBTB16 | 1768.331257 | 489.8326424 | 1.852028 | 1.29E-14 | up | 356 | 641 | 401 | 2041 | 1463 | 1893 | ZBTB16 | + | zinc finger and BTB domain containing 16 | 0 | ZBTB16 |
| DCHS1 | 147.1764912 | 258.6036134 | -0.8132 | 1.39E-14 | down | 277 | 215 | 275 | 186 | 123 | 144 | DCHS1 | + | Uncharacterized protein | 0 | DCHS1 |
| ITGB1BP2 | 373.4335882 | 1183.376565 | -1.66399 | 1.42E-14 | down | 1234 | 1075 | 1184 | 690 | 249 | 257 | ITGB1BP2 | + | Uncharacterized protein | 9.00E-174 | ITGB1BP2 |
| HOXC6 | 253.7384708 | 390.0675553 | -0.62038 | 1.44E-14 | down | 417 | 353 | 383 | 290 | 230 | 255 | HOXC6 | - | Homeobox protein Hox-C6 | 2.00E-87 | HOXC6 |
| LOC101110574 | 169.7865531 | 67.72780896 | 1.325902 | 1.72E-14 | up | 83 | 59 | 60 | 225 | 174 | 128 | LOC101110574 | + | 60S ribosomal protein L10a-like | | |
| FTH1 | 36433.11543 | 20327.73081 | 0.841801 | 2.25E-14 | up | 20407 | 19811 | 19494 | 39656 | 35198 | 36100 | FTH1 | - | Ferritin heavy chain | 3.00E-86 | FTH1 |
| OMYHCS | 88659.65715 | 225229.999 | -1.34505 | 3.66E-14 | down | 291283 | 197248 | 185247 | 113972 | 82107 | 77753 | OMYHCS | + | Myosin heavy chain slow | 0 | MYH7 |
| RGMA | 101.3522251 | 252.9777908 | -1.31963 | 4.60E-14 | down | 259 | 215 | 274 | 153 | 73 | 91 | RGMA | + | Uncharacterized protein | 1.00E-122 | RGMA |
| TNNT1 | 17000.23345 | 44366.56216 | -1.38392 | 4.84E-14 | down | 45862 | 34973 | 50771 | 16458 | 17941 | 16994 | TNNT1 | + | Uncharacterized protein | 3.00E-104 | TNNT1 |
| CLC4 | 246.233173 | 642.8738382 | -1.38451 | 5.31E-14 | down | 686 | 522 | 700 | 291 | 154 | 305 | CLC4 | - | Chloride channel ClC4 | 0 | CLCN4 |
| C17H22orf25 | 321.8665724 | 112.9114554 | 1.511271 | 7.85E-14 | up | 83 | 128 | 114 | 429 | 320 | 250 | C17H22orf25 | + | Uncharacterized protein | 7.00E-130 | TANGO2 |
| C1H1orf51 | 147.5109995 | 21.25750626 | 2.794778 | 2.09E-13 | up | 11 | 13 | 38 | 168 | 111 | 170 | C1H1orf51 | + | Uncharacterized protein | 1.00E-155 | CIART |
| CAMK2B | 979.0561731 | 622.7675129 | 0.652698 | 2.26E-13 | up | 595 | 600 | 631 | 1176 | 975 | 858 | CAMK2B | + | Uncharacterized protein | 0 | CAMK2B |
| TMEM182 | 556.0645151 | 1069.859845 | -0.9441 | 2.31E-13 | down | 1238 | 868 | 1083 | 805 | 429 | 499 | TMEM182 | - | Uncharacterized protein | 8.00E-122 | TMEM182 |
| VNN1 | 13.23537483 | 60.37234176 | -2.18949 | 2.57E-13 | down | 57 | 50 | 71 | 16 | 18 | 7 | VNN1 | - | Uncharacterized protein | 0 | VNN1 |
| NMT2 | 95.43314071 | 34.03722573 | 1.487377 | 2.94E-13 | up | 28 | 34 | 37 | 95 | 99 | 95 | NMT2 | - | Glycylpeptide N-tetradecanoyltransferase | 0 | NMT2 |
| MASP1 | 61.87822941 | 209.6887026 | -1.76075 | 4.50E-13 | down | 280 | 165 | 186 | 105 | 41 | 50 | MASP1 | + | Uncharacterized protein | 0 | MASP1 |
| KCNJ12 | 343.5465939 | 1060.515947 | -1.62619 | 4.84E-13 | down | 1177 | 959 | 1005 | 355 | 157 | 519 | KCNJ12 | + | Uncharacterized protein | 0 | KCNJ12 |
| SREBF1 | 210.2108314 | 730.9851994 | -1.79801 | 6.85E-13 | down | 723 | 748 | 670 | 243 | 115 | 280 | SREBF1 | + | Sterol regulatory element-binding transcription factor 1 | 0 | SREBF1 |
| LOC101107541 | 1066.297181 | 3365.518702 | -1.65822 | 8.53E-13 | down | 3641 | 2473 | 3916 | 1480 | 687 | 1133 | LOC101107541 | + | Uncharacterized protein | 2.00E-145 | AKR1C1 |
| CST6 | 28.88019055 | 105.6451629 | -1.87107 | 8.57E-13 | down | 134 | 90 | 92 | 27 | 13 | 46 | CST6 | + | Cystatin-B | 2.00E-57 | CST6 |
| LOC101110796 | 9.078282456 | 90.8186535 | -3.3225 | 9.00E-13 | down | 133 | 56 | 88 | 17 | 4 | 8 | LOC101110796 | - | leucine-rich repeat neuronal protein 1-like | 0 | LRRN1 |
| QPCT | 21.22855073 | 66.06535444 | -1.63789 | 1.02E-12 | down | 84 | 46 | 69 | 28 | 24 | 14 | QPCT | - | Glutaminyl-peptide cyclotransferase | 0 | QPCT |
| SH2D4A | 283.7292416 | 30.30642067 | 3.226819 | 1.03E-12 | up | 7 | 50 | 26 | 378 | 337 | 169 | SH2D4A | - | Uncharacterized protein | 3.00E-166 | SH2D4A |
| PDLIM3 | 94737.26335 | 28300.0577 | 1.743127 | 1.04E-12 | up | 22997 | 20815 | 39447 | 94769 | 92903 | 98994 | PDLIM3 | - | Uncharacterized protein | 0 | PDLIM3 |
| SESN1 | 4907.210295 | 623.2063446 | 2.977121 | 1.19E-12 | up | 557 | 501 | 777 | 4658 | 6284 | 3940 | SESN1 | + | Uncharacterized protein | 0 | SESN1 |
| MYC | 1252.153492 | 233.7906604 | 2.421122 | 1.48E-12 | up | 564 | 140 | 41 | 1460 | 1406 | 982 | MYC | - | Myc proto-oncogene protein | 0 | MYC |
| CA2 | 262.1757619 | 933.4641547 | -1.83206 | 1.56E-12 | down | 1176 | 901 | 700 | 239 | 226 | 321 | CA2 | - | Carbonic anhydrase 2 | 8.00E-119 | CA2 |
| RNF149 | 107.8589586 | 231.5654859 | -1.10227 | 1.68E-12 | down | 254 | 205 | 227 | 155 | 69 | 111 | RNF149 | + | Uncharacterized protein | 6.00E-106 | RNF149 |
| KLHDC8B | 79.57281756 | 169.4114407 | -1.09018 | 1.77E-12 | down | 215 | 150 | 141 | 83 | 85 | 74 | KLHDC8B | - | Uncharacterized protein | 0 | KLHDC8B |

| | | | | | | | | | | | | | | | | |
|---|---|---|---|---|---|---|---|---|---|---|---|---|---|---|---|---|
| LARP6 | 186.1049186 | 97.67413968 | 0.930068 | 1.84E-12 | up | 107 | 89 | 93 | 248 | 159 | 169 | LARP6 | - | Uncharacterized protein | 0 | LARP6 |
| PKHD1 | 28.98379606 | 2.729382903 | 3.4086 | 2.01E-12 | up | 2 | 2 | 4 | 32 | 22 | 34 | PKHD1 | - | Uncharacterized protein | 0 | PKHD1 |
| EIF2C1 | 493.6139323 | 885.0824667 | -0.84243 | 3.42E-12 | down | 928 | 768 | 922 | 584 | 395 | 530 | EIF2C1 | + | Protein argonaute | 0 | AGO1 |
| SFRP2 | 8.685556591 | 51.06010716 | -2.55551 | 3.58E-12 | down | 71 | 33 | 51 | 13 | 4 | 10 | SFRP2 | + | Secreted frizzled-related protein 2 | 6.00E-167 | SFRP2 |
| MIF4GD | 1317.849444 | 511.8386129 | 1.364425 | 3.71E-12 | up | 489 | 529 | 478 | 1427 | 1520 | 1080 | MIF4GD | + | MIF4G domain containing | 6.00E-87 | MIF4GD |
| AGXT2L1 | 419.726203 | 1471.602546 | -1.80987 | 3.94E-12 | down | 1359 | 1321 | 1641 | 419 | 269 | 573 | AGXT2L1 | + | Uncharacterized protein | 0 | ETNPPL |
| LOC101120819 | 210.6109413 | 114.0366835 | 0.885082 | 4.17E-12 | up | 125 | 92 | 122 | 225 | 229 | 188 | LOC101120819 | - | Uncharacterized protein | 0 | HNRNPA1 |
| HRC | 3870.385658 | 7034.373774 | -0.86194 | 6.12E-12 | down | 7497 | 6930 | 6288 | 4715 | 3767 | 3423 | HRC | - | Uncharacterized protein | 3.00E-166 | HRC |
| MYL2 | 23309.2945 | 63933.45192 | -1.45567 | 7.13E-12 | down | 76275 | 46219 | 69149 | 21705 | 31231 | 17742 | MYL2 | - | Myosin light chain 2 | 4.00E-89 | MYL2 |
| CDNF | 55.22660791 | 134.2818872 | -1.28183 | 7.22E-12 | down | 138 | 115 | 144 | 85 | 38 | 50 | CDNF | - | cerebral dopamine neurotrophic factor | 9.00E-94 | CDNF |
| MXRA5 | 221.7872352 | 680.508949 | -1.61744 | 7.93E-12 | down | 858 | 578 | 599 | 299 | 89 | 293 | MXRA5 | - | Uncharacterized protein | 0 | MXRA5 |
| LEPREL1 | 58.51571198 | 124.3665078 | -1.0877 | 9.45E-12 | down | 163 | 104 | 106 | 67 | 57 | 55 | LEPREL1 | + | Uncharacterized protein | 0 | LEPREL1 |
| CAMK2G | 486.8823402 | 1199.344128 | -1.3006 | 9.94E-12 | down | 1154 | 1108 | 1262 | 669 | 342 | 496 | CAMK2G | - | Uncharacterized protein | 0 | CAMK2G |
| WDR16 | 13.48292755 | 167.3336581 | -3.63352 | 1.96E-11 | down | 147 | 122 | 225 | 9 | 1 | 29 | WDR16 | + | WD repeat domain 16 | 0 | CFAP52 |
| GPR116 | 1982.390659 | 1148.083004 | 0.788014 | 1.96E-11 | up | 1566 | 945 | 943 | 2309 | 2008 | 1761 | GPR116 | - | Uncharacterized protein | 0 | GPR116 |
| USP54 | 492.6393592 | 244.2037364 | 1.012447 | 1.98E-11 | up | 241 | 221 | 257 | 644 | 504 | 379 | USP54 | - | Uncharacterized protein | 0 | USP54 |
| FZD9 | 17.0094205 | 62.77971617 | -1.88396 | 2.17E-11 | down | 59 | 60 | 65 | 30 | 9 | 15 | FZD9 | - | Uncharacterized protein | 0 | FZD9 |
| MUSTN1 | 25343.86189 | 3310.238866 | 2.936629 | 2.43E-11 | up | 2754 | 2131 | 4903 | 31713 | 16854 | 29098 | MUSTN1 | + | musculoskeletal, embryonic nuclear protein 1 | 1.00E-39 | MUSTN1 |
| FAM107A | 173.3808572 | 63.37389992 | 1.451984 | 3.99E-11 | up | 73 | 73 | 40 | 236 | 168 | 135 | FAM107A | + | Uncharacterized protein | 3.00E-67 | FAM107A |
| CNTNAP1 | 48.11416431 | 116.8498833 | -1.28012 | 4.15E-11 | down | 145 | 100 | 104 | 78 | 39 | 35 | CNTNAP1 | + | Uncharacterized protein | 0 | CNTNAP1 |
| PPM1K | 118.3936054 | 879.010029 | -2.89229 | 4.55E-11 | down | 981 | 562 | 1092 | 301 | 43 | 52 | PPM1K | + | Uncharacterized protein | 0 | PPM1K |
| EIF3B | 2559.508773 | 1612.373035 | 0.666681 | 5.74E-11 | up | 1704 | 1612 | 1427 | 2986 | 2342 | 2505 | EIF3B | - | Eukaryotic translation initiation factor 3 subunit B | 0 | EIF3B |
| LOC101113195 | 134.8927246 | 227.1463768 | -0.75181 | 5.85E-11 | down | 244 | 210 | 217 | 134 | 133 | 141 | LOC101113195 | + | uncharacterized LOC101113195 | 2.00E-37 | COA6 |
| LOC101118156 | 30.29822316 | 90.09717831 | -1.57225 | 7.25E-11 | down | 113 | 64 | 94 | 44 | 16 | 34 | LOC101118156 | - | ornithine aminotransferase pseudogene | | |
| OAT | 70.8432272 | 167.1316639 | -1.23828 | 8.43E-11 | down | 203 | 117 | 182 | 98 | 59 | 63 | OAT | - | Uncharacterized protein | 0 | OAT |
| MYH7B | 380.7357804 | 1533.55853 | -2.01002 | 9.47E-11 | down | 1883 | 1029 | 1703 | 472 | 596 | 119 | MYH7B | + | Uncharacterized protein | 0 | MYH7B |
| MLH1 | 161.2759484 | 274.9046428 | -0.7694 | 1.13E-10 | down | 311 | 264 | 238 | 191 | 144 | 159 | MLH1 | - | Uncharacterized protein | 0 | MLH1 |
| MYH6 | 4502.737351 | 12787.65091 | -1.50588 | 1.17E-10 | down | 17653 | 9576 | 11398 | 5828 | 4311 | 3786 | MYH6 | + | myosin, heavy chain 6, cardiac muscle, alpha | 0 | MYH6 |
| CREB3L1 | 86.70298015 | 189.2762538 | -1.12634 | 1.21E-10 | down | 202 | 167 | 191 | 124 | 53 | 92 | CREB3L1 | + | Uncharacterized protein | 0 | CREB3L1 |
| RAPSN | 269.0737244 | 139.8930024 | 0.943678 | 1.45E-10 | up | 151 | 132 | 130 | 326 | 242 | 258 | RAPSN | - | Uncharacterized protein | 0 | RAPSN |
| ZNF496 | 260.481524 | 167.6688903 | 0.635566 | 1.46E-10 | up | 160 | 166 | 165 | 313 | 250 | 237 | ZNF496 | - | Zinc finger protein 496 | 0 | ZNF496 |

| | | | | | | | | | | | | | | | | | |
|---|---|---|---|---|---|---|---|---|---|---|---|---|---|---|---|---|---|
| MMP15 | 87.47815401 | 420.8002669 | -2.26614 | 1.67E-10 | down | 459 | 465 | 310 | 110 | 55 | 103 | MMP15 | + | Uncharacterized protein | 3.00E-169 | MMP15 |
| DYRK1B | 651.4066945 | 1404.550559 | -1.10848 | 2.31E-10 | down | 1474 | 1315 | 1353 | 558 | 600 | 789 | DYRK1B | - | Uncharacterized protein | 0 | DYRK1B |
| IPO4 | 672.9529612 | 259.4374489 | 1.375119 | 2.36E-10 | up | 301 | 244 | 224 | 644 | 748 | 643 | IPO4 | + | Uncharacterized protein | 0 | IPO4 |
| LMAN2L | 86.38532124 | 173.8262989 | -1.00879 | 2.67E-10 | down | 169 | 153 | 190 | 100 | 76 | 88 | LMAN2L | + | Uncharacterized protein | 0 | LMAN2L |
| LOC101119529 | 457.0289165 | 174.3175073 | 1.390568 | 2.90E-10 | up | 184 | 127 | 208 | 439 | 485 | 457 | LOC101119529 | + | E3 ubiquitin-protein ligase RNF146-like | | |
| NSUN2 | 679.7356434 | 417.9196415 | 0.701748 | 3.58E-10 | up | 451 | 423 | 356 | 756 | 708 | 613 | NSUN2 | + | Uncharacterized protein | 0 | NSUN2 |
| UBE2Q1 | 1140.966399 | 541.718672 | 1.074641 | 3.64E-10 | up | 456 | 557 | 562 | 1237 | 1254 | 992 | UBE2Q1 | - | Uncharacterized protein | 0 | UBE2Q1 |
| CPSF3L | 595.2549357 | 394.2795362 | 0.594289 | 3.88E-10 | up | 421 | 337 | 410 | 709 | 592 | 527 | CPSF3L | + | Uncharacterized protein | 0 | CPSF3L |
| NOP16 | 282.4007591 | 175.7831017 | 0.683948 | 3.92E-10 | up | 188 | 141 | 193 | 304 | 285 | 271 | NOP16 | - | Uncharacterized protein | 9.00E-96 | NOP16 |
| ALDH18A1 | 603.3092514 | 342.2219516 | 0.817965 | 5.40E-10 | up | 403 | 316 | 297 | 627 | 640 | 567 | ALDH18A1 | - | Uncharacterized protein | 0 | ALDH18A1 |
| LOC101109564 | 439.4083119 | 196.5840308 | 1.160416 | 5.46E-10 | up | 130 | 191 | 247 | 503 | 426 | 415 | LOC101109564 | + | uncharacterized LOC101109564 | | |
| CACNB1 | 2314.979104 | 3907.082246 | -0.75509 | 5.72E-10 | down | 4720 | 3391 | 3534 | 3190 | 1909 | 2086 | CACNB1 | - | Uncharacterized protein | 0 | CACNB1 |
| ORMDL3 | 244.9115899 | 970.8346381 | -1.98696 | 5.84E-10 | down | 983 | 689 | 1215 | 260 | 199 | 283 | ORMDL3 | - | Uncharacterized protein | 2.00E-85 | ORMDL3 |
| AATF | 181.3141193 | 82.13125385 | 1.142488 | 6.89E-10 | up | 89 | 89 | 63 | 188 | 175 | 187 | AATF | + | Uncharacterized protein | 0 | AATF |
| CRYL1 | 157.6592212 | 77.37599406 | 1.026852 | 8.07E-10 | up | 114 | 56 | 65 | 173 | 161 | 147 | CRYL1 | + | Uncharacterized protein | 1.00E-148 | CRYL1 |
| CGREF1 | 172.1998714 | 69.2266922 | 1.314684 | 8.37E-10 | up | 51 | 73 | 76 | 231 | 142 | 160 | CGREF1 | - | Uncharacterized protein | 3.00E-88 | CGREF1 |
| NSMAF | 1063.913855 | 472.3584239 | 1.171427 | 1.24E-09 | up | 596 | 368 | 453 | 1304 | 1191 | 789 | NSMAF | - | neutral sphingomyelinase (N-SMase) activation associated factor | 0 | NSMAF |
| SLC9A2 | 84.72272947 | 193.9361215 | -1.19476 | 1.29E-09 | down | 225 | 151 | 203 | 105 | 50 | 104 | SLC9A2 | - | Sodium/hydrogen exchanger | 0 | SLC9A2 |
| GPD1 | 6108.79247 | 18995.4779 | -1.6367 | 1.32E-09 | down | 17677 | 19997 | 17731 | 7243 | 4768 | 6660 | GPD1 | - | Glycerol-3-phosphate dehydrogenase [NAD(+)] | 0 | GPD1 |
| KIAA1456 | 5.415568511 | 31.10979336 | -2.52218 | 1.38E-09 | down | 41 | 21 | 32 | 13 | 3 | 2 | KIAA1456 | - | Uncharacterized protein | 0 | KIAA1456 |
| CCDC80 | 553.2934101 | 2517.523592 | -2.18589 | 1.48E-09 | down | 3313 | 1504 | 2815 | 654 | 309 | 720 | CCDC80 | - | Uncharacterized protein | 0 | CCDC80 |
| ST5 | 241.0939096 | 383.8021904 | -0.67077 | 1.71E-09 | down | 452 | 320 | 372 | 335 | 200 | 214 | ST5 | + | Uncharacterized protein | 0 | ST5 |
| HPDL | 12.27405889 | 43.69494886 | -1.83185 | 1.72E-09 | down | 56 | 28 | 48 | 16 | 12 | 10 | HPDL | + | Uncharacterized protein | 2.00E-174 | HPDL |
| EAPP | 99.26982295 | 41.11703218 | 1.271619 | 2.13E-09 | up | 57 | 25 | 43 | 111 | 112 | 81 | EAPP | - | Uncharacterized protein | 7.00E-136 | EAPP |
| C21H11orf48 | 288.8419194 | 169.8611999 | 0.765924 | 2.28E-09 | up | 197 | 118 | 194 | 323 | 301 | 259 | C21H11orf48 | - | chromosome 21 open reading frame, human C11orf48 | 1.00E-95 | LBHD1 |
| LRP4 | 1142.316748 | 410.207617 | 1.477537 | 2.33E-09 | up | 466 | 450 | 290 | 1252 | 1348 | 896 | LRP4 | - | Uncharacterized protein | 0 | LRP4 |
| DST | 4046.588753 | 6234.153063 | -0.62349 | 2.79E-09 | down | 7549 | 5020 | 6066 | 5413 | 3891 | 3252 | DST | + | Uncharacterized protein | 0 | DST |
| LOC101114133 | 83.21282325 | 31.41740061 | 1.405242 | 2.79E-09 | up | 20 | 28 | 43 | 87 | 87 | 79 | LOC101114133 | - | uncharacterized LOC101114133 | | |
| LOC101102254 | 25.97637123 | 68.98658121 | -1.40912 | 2.88E-09 | down | 65 | 60 | 78 | 37 | 23 | 21 | LOC101102254 | - | intercellular adhesion molecule 2-like | | |
| ASB15 | 257.4571927 | 554.5725291 | -1.10704 | 3.30E-09 | down | 597 | 410 | 645 | 326 | 238 | 230 | ASB15 | + | Uncharacterized protein | 0 | ASB15 |
| TMEM38A | 2004.372417 | 3767.071831 | -0.91029 | 3.82E-09 | down | 4377 | 3512 | 3282 | 2588 | 1481 | 2101 | TMEM38A | - | Uncharacterized protein | 1.00E-151 | TMEM38A |

| | | | | | | | | | | | | | | | | |
|---|---|---|---|---|---|---|---|---|---|---|---|---|---|---|---|---|
| GPATCH4 | 157.9179071 | 69.06522872 | 1.193143 | 3.99E-09 | up | 75 | 80 | 47 | 167 | 172 | 142 | GPATCH4 | - | Uncharacterized protein | 6.00E-130 | GPATCH4 |
| FNTB | 748.8333289 | 278.8117012 | 1.425354 | 4.27E-09 | up | 300 | 263 | 260 | 692 | 804 | 761 | FNTB | + | farnesyltransferase, CAAX box, beta | 0 | CHURC1-FNTB |
| MRC2 | 228.0121041 | 764.6913378 | -1.74577 | 5.30E-09 | down | 1069 | 533 | 715 | 292 | 131 | 276 | MRC2 | + | Uncharacterized protein | 0 | MRC2 |
| MANEAL | 53.9889156 | 12.30083988 | 2.133906 | 5.33E-09 | up | 27 | 6 | 6 | 74 | 53 | 41 | MANEAL | + | mannosidase, endo-alpha-like | 4.00E-156 | MANEAL |
| ABCB9 | 21.08561562 | 57.88460065 | -1.45692 | 6.37E-09 | down | 74 | 51 | 48 | 24 | 13 | 27 | ABCB9 | + | Uncharacterized protein | 0 | ABCB9 |
| PTGES3L | 90.55625524 | 299.0431866 | -1.72347 | 6.56E-09 | down | 265 | 257 | 356 | 122 | 68 | 90 | PTGES3L | - | prostaglandin E synthase 3 (cytosolic)-like | 1.00E-67 | PTGES3L-AARSD1 |
| HMOX1 | 1141.803853 | 126.9970956 | 3.168447 | 6.82E-09 | up | 217 | 99 | 73 | 924 | 1483 | 1020 | HMOX1 | + | Uncharacterized protein | 2.00E-139 | HMOX1 |
| GAL-1 | 1542.340159 | 3771.58081 | -1.29005 | 7.17E-09 | down | 3967 | 2999 | 4228 | 1555 | 1097 | 1992 | GAL-1 | + | Galectin | 2.00E-65 | LGALS1 |
| ARRDC2 | 3535.801724 | 303.4303963 | 3.5426 | 7.71E-09 | up | 108 | 579 | 139 | 3667 | 2386 | 4609 | ARRDC2 | - | Uncharacterized protein | 0 | ARRDC2 |
| INO80B | 304.6922498 | 151.5179141 | 1.007864 | 8.15E-09 | up | 142 | 145 | 157 | 306 | 327 | 291 | INO80B | + | Uncharacterized protein | 6.00E-149 | INO80B |
| FLVCR2 | 29.62656927 | 146.3802736 | -2.30476 | 8.28E-09 | down | 132 | 119 | 180 | 12 | 20 | 53 | FLVCR2 | + | Uncharacterized protein | 0 | FLVCR2 |
| CHPT1 | 163.2434266 | 347.7041268 | -1.09084 | 1.05E-08 | down | 331 | 345 | 342 | 205 | 134 | 163 | CHPT1 | + | Uncharacterized protein | 1.00E-142 | CHPT1 |
| LMOD2 | 12507.28684 | 5623.44244 | 1.153243 | 1.18E-08 | up | 7213 | 3331 | 6481 | 14120 | 11001 | 13019 | LMOD2 | + | Uncharacterized protein | 0 | LMOD2 |
| LOC101106528 | 21.65423421 | 65.27305058 | -1.59184 | 1.21E-08 | down | 80 | 62 | 52 | 37 | 20 | 12 | LOC101106528 | - | T-cell receptor delta chain C region-like | | |
| SCNM1 | 222.347315 | 399.8816079 | -0.84676 | 1.24E-08 | down | 472 | 334 | 386 | 265 | 159 | 255 | SCNM1 | + | Uncharacterized protein | 6.00E-119 | SCNM1 |
| LOC101107954 | 1360.910075 | 478.8783953 | 1.50684 | 1.39E-08 | up | 763 | 517 | 160 | 1515 | 1422 | 1222 | LOC101107954 | + | Cytochrome c | 1.00E-51 | CYCS |
| KLHL31 | 619.0857407 | 1744.899266 | -1.49493 | 1.69E-08 | down | 2159 | 1196 | 1895 | 1007 | 396 | 547 | KLHL31 | + | kelch-like 31 (Drosophila) | 0 | KLHL31 |
| G3BP2 | 438.0171215 | 898.9814677 | -1.0373 | 1.69E-08 | down | 1037 | 693 | 954 | 473 | 567 | 302 | G3BP2 | - | Uncharacterized protein | 0 | G3BP2 |
| NFIX | 2638.054759 | 5652.90902 | -1.09952 | 1.86E-08 | down | 5980 | 5467 | 5207 | 2943 | 1863 | 3202 | NFIX | - | Nuclear factor 1 | 0 | NFIX |
| GADD45G | 1155.761914 | 49.02994405 | 4.559037 | 2.23E-08 | up | 96 | 30 | 27 | 1666 | 532 | 1380 | GADD45G | + | Growth arrest and DNA-damage-inducible gamma | 3.00E-87 | GADD45G |
| EDEM1 | 308.2149794 | 134.3680003 | 1.197747 | 2.89E-08 | up | 149 | 106 | 145 | 384 | 348 | 221 | EDEM1 | - | alpha-1,2-Mannosidase | 0 | EDEM1 |
| PROB1 | 403.3342815 | 720.7510144 | -0.83752 | 2.99E-08 | down | 824 | 645 | 670 | 432 | 314 | 476 | PROB1 | - | proline-rich basic protein 1 | 0 | PROB1 |
| LOC101106024 | 5.268784546 | 29.29372951 | -2.47505 | 3.06E-08 | down | 38 | 23 | 27 | 15 | 2 | 1 | LOC101106024 | + | secreted and transmembrane protein 1A-like | | |
| KIAA0232 | 491.4194144 | 895.0849954 | -0.86507 | 3.27E-08 | down | 912 | 783 | 948 | 684 | 489 | 359 | KIAA0232 | + | KIAA0232 ortholog | 0 | KIAA0232 |
| KLF15 | 124.2020566 | 45.6742425 | 1.443236 | 3.81E-08 | up | 47 | 47 | 40 | 146 | 124 | 111 | KLF15 | + | Uncharacterized protein | 5.00E-119 | KLF15 |
| AHDC1 | 173.4928874 | 394.6164169 | -1.18557 | 3.98E-08 | down | 400 | 390 | 369 | 220 | 110 | 202 | AHDC1 | + | Uncharacterized protein | 0 | AHDC1 |
| ECHDC3 | 949.3372455 | 246.5108556 | 1.94527 | 4.33E-08 | up | 267 | 194 | 272 | 1228 | 1123 | 598 | ECHDC3 | + | Uncharacterized protein | 4.00E-122 | ECHDC3 |
| RGCC | 935.938779 | 306.8053741 | 1.60909 | 4.67E-08 | up | 297 | 357 | 238 | 1225 | 677 | 982 | RGCC | + | Uncharacterized protein | 1.00E-18 | RGCC |
| DHTKD1 | 396.5721409 | 611.702189 | -0.62525 | 5.33E-08 | down | 618 | 569 | 614 | 444 | 422 | 347 | DHTKD1 | + | Uncharacterized protein | 0 | DHTKD1 |
| TLR7 | 11.29224423 | 39.05385101 | -1.79013 | 5.54E-08 | down | 43 | 40 | 32 | 6 | 12 | 15 | TLR7 | + | Toll-like receptor 7 | 0 | TLR7 |
| IGFBP5 | 195.9773056 | 1216.778217 | -2.63431 | 6.57E-08 | down | 1803 | 714 | 1202 | 388 | 104 | 140 | IGFBP5 | - | Uncharacterized protein | 2.00E-147 | IGFBP5 |
| LOC101120590 | 254.5113638 | 525.3541617 | -1.04556 | 6.58E-08 | down | 619 | 468 | 475 | 332 | 152 | 298 | LOC101120590 | + | Uncharacterized protein | 3.00E-100 | ARF1 |

| | | | | | | | | | | | | | | | | | |
|---|---|---|---|---|---|---|---|---|---|---|---|---|---|---|---|---|---|
| PDXP | 60.5698892 | 185.6329748 | -1.61578 | 6.97E-08 | down | 179 | 145 | 225 | 94 | 43 | 53 | PDXP | + | Uncharacterized protein | 7.00E-158 | PDXP |
| APBB2 | 1310.031519 | 589.0851724 | 1.153053 | 7.29E-08 | up | 596 | 363 | 800 | 1484 | 1426 | 1103 | APBB2 | - | Uncharacterized protein | 0 | APBB2 |
| LOC101109676 | 43.59813047 | 13.14696883 | 1.729536 | 8.95E-08 | up | 17 | 14 | 8 | 42 | 50 | 40 | LOC101109676 | + | 60S ribosomal protein L17-like | | |
| NCEH1 | 239.9666937 | 70.45838117 | 1.767991 | 9.50E-08 | up | 75 | 73 | 59 | 355 | 175 | 219 | NCEH1 | + | Uncharacterized protein | 0 | NCEH1 |
| YIPF7 | 219.4728041 | 562.0457144 | -1.35665 | 1.02E-07 | down | 573 | 421 | 675 | 228 | 182 | 254 | YIPF7 | - | Protein YIPF | 1.00E-129 | YIPF7 |
| TUB | 20.71146405 | 58.16589001 | -1.48974 | 1.04E-07 | down | 55 | 52 | 64 | 33 | 12 | 20 | TUB | - | Tubby-like protein | 0 | TUB |
| SLC22A16 | 229.8883502 | 94.92522149 | 1.27607 | 1.07E-07 | up | 135 | 96 | 53 | 245 | 256 | 200 | SLC22A16 | + | Uncharacterized protein | 0 | SLC22A16 |
| LDHB | 827.3723722 | 1608.181673 | -0.95882 | 1.25E-07 | down | 1935 | 1177 | 1709 | 1200 | 734 | 651 | LDHB | + | L-lactate dehydrogenase | 0 | LDHB |
| PGK1 | 6945.387884 | 20207.27227 | -1.54075 | 1.25E-07 | down | 21220 | 21420 | 16622 | 5658 | 6451 | 8586 | PGK1 | + | Phosphoglycerate kinase 1 | 0 | PGK1 |
| DDX27 | 491.4054445 | 217.4641727 | 1.176136 | 1.40E-07 | up | 235 | 198 | 210 | 496 | 579 | 419 | DDX27 | + | Uncharacterized protein | 0 | DDX27 |
| OLA1 | 260.3596863 | 168.8805377 | 0.624503 | 1.47E-07 | up | 197 | 119 | 190 | 322 | 254 | 226 | OLA1 | + | Uncharacterized protein | 0 | OLA1 |
| ST3GAL1 | 328.8051902 | 163.2516182 | 1.010136 | 1.50E-07 | up | 167 | 159 | 154 | 332 | 312 | 351 | ST3GAL1 | + | Uncharacterized protein | 0 | ST3GAL1 |
| ADAM11 | 0.646964188 | 15.53120469 | -4.58534 | 1.53E-07 | down | 20 | 17 | 9 | 1 | 0 | 1 | ADAM11 | + | Uncharacterized protein | 0 | ADAM11 |
| TMEM120A | 541.9802395 | 279.5817496 | 0.95497 | 1.58E-07 | up | 311 | 254 | 263 | 737 | 433 | 509 | TMEM120A | - | Uncharacterized protein | 2.00E-162 | TMEM120A |
| SLC4A4 | 16.12483935 | 71.13660553 | -2.14131 | 1.68E-07 | down | 104 | 51 | 61 | 31 | 12 | 9 | SLC4A4 | + | Putative sodium bicarbonate cotransporter | 0 | SLC4A4 |
| AMPD1 | 2881.739989 | 8247.763365 | -1.51706 | 1.80E-07 | down | 8842 | 5998 | 9737 | 4728 | 2112 | 2263 | AMPD1 | - | Uncharacterized protein | 0 | AMPD1 |
| SFRP5 | 54.48017218 | 128.5185404 | -1.23817 | 1.82E-07 | down | 162 | 104 | 119 | 66 | 29 | 71 | SFRP5 | - | Uncharacterized protein | 6.00E-108 | SFRP5 |
| SCARB1 | 165.6742785 | 351.3544356 | -1.08458 | 1.87E-07 | down | 456 | 242 | 362 | 223 | 137 | 153 | SCARB1 | + | Uncharacterized protein | 0 | SCARB1 |
| GALNT12 | 42.19011915 | 104.3099686 | -1.3059 | 1.95E-07 | down | 130 | 94 | 87 | 71 | 36 | 27 | GALNT12 | - | Polypeptide N-acetylgalactosaminyltransferase | 0 | GALNT12 |
| LRRC2 | 2044.502828 | 795.8309228 | 1.361216 | 2.11E-07 | up | 431 | 735 | 1125 | 2166 | 2062 | 1990 | LRRC2 | + | leucine rich repeat containing 2 | 0 | LRRC2 |
| CTSH | 289.0294442 | 161.65938 | 0.838259 | 2.21E-07 | up | 184 | 142 | 154 | 365 | 281 | 246 | CTSH | - | Uncharacterized protein | 4.00E-172 | CTSH |
| HSPBAP1 | 139.4783087 | 50.3868991 | 1.46892 | 2.27E-07 | up | 58 | 42 | 50 | 135 | 169 | 119 | HSPBAP1 | - | HSPB (heat shock 27kDa) associated protein 1 | 0 | HSPBAP1 |
| DECR1 | 115.8826402 | 192.9569547 | -0.73561 | 2.68E-07 | down | 199 | 162 | 210 | 126 | 130 | 98 | DECR1 | - | Uncharacterized protein | 1.00E-161 | DECR1 |
| TACC2 | 8554.806975 | 3411.676122 | 1.326255 | 2.82E-07 | up | 2645 | 4128 | 3032 | 8509 | 9408 | 8021 | TACC2 | + | Uncharacterized protein | 0 | TACC2 |
| IRAK2 | 25.4432334 | 96.19521564 | -1.91868 | 3.25E-07 | down | 139 | 83 | 68 | 29 | 28 | 21 | IRAK2 | - | Uncharacterized protein | 0 | IRAK2 |
| CTTN | 609.3744498 | 394.7775271 | 0.626289 | 3.29E-07 | up | 464 | 326 | 387 | 726 | 519 | 621 | CTTN | + | Uncharacterized protein | 0 | CTTN |
| SNAI3 | 3.993401953 | 34.27296808 | -3.10138 | 3.36E-07 | down | 25 | 36 | 38 | 9 | 0 | 4 | SNAI3 | - | Snail-like protein 3 | | |
| PLEKHB2 | 559.3215882 | 1011.012528 | -0.85405 | 3.78E-07 | down | 965 | 938 | 1066 | 715 | 557 | 457 | PLEKHB2 | + | pleckstrin homology domain containing, family B (evectins) member 2 | 1.00E-117 | PLEKHB2 |
| PSMA7 | 1560.053121 | 909.5383913 | 0.778389 | 3.82E-07 | up | 950 | 803 | 935 | 1604 | 1713 | 1425 | PSMA7 | + | Uncharacterized protein | 8.00E-114 | PSMA7 |
| FOSL2 | 1233.941517 | 209.535894 | 2.558005 | 3.89E-07 | up | 408 | 155 | 87 | 1043 | 1557 | 1111 | FOSL2 | + | Uncharacterized protein | 7.00E-158 | FOSL2 |
| TRMT12 | 142.2927062 | 267.1445395 | -0.90876 | 3.96E-07 | down | 257 | 269 | 256 | 170 | 140 | 127 | TRMT12 | - | tRNA methyltransferase 12 | 0 | TRMT12 |

| | | | | | | | | | | | | | | | | |
|---|---|---|---|---|---|---|---|---|---|---|---|---|---|---|---|---|
| | | | | | | | | | | | | | | homolog (S. cerevisiae) | | |
| LOC101121418 | 94.90302494 | 176.828964 | -0.89783 | 4.00E-07 | down | 188 | 149 | 187 | 119 | 65 | 107 | LOC101121418 | + | uncharacterized LOC101121418 | 3.00E-103 | KDELR3 |
| SLC7A6OS | 322.8097417 | 165.641671 | 0.962618 | 4.35E-07 | up | 205 | 124 | 168 | 317 | 342 | 318 | SLC7A6OS | - | Uncharacterized protein | 2.00E-117 | SLC7A6OS |
| TGFB2 | 55.01778609 | 126.0595664 | -1.19614 | 4.73E-07 | down | 162 | 95 | 122 | 88 | 36 | 49 | TGFB2 | + | Transforming growth factor-beta 2 | 0 | TGFB2 |
| RNF25 | 397.8462975 | 238.2204733 | 0.739914 | 5.17E-07 | up | 258 | 243 | 200 | 427 | 381 | 402 | RNF25 | - | Uncharacterized protein | 0 | RNF25 |
| LOC101104304 | 97.23531765 | 177.5600596 | -0.86875 | 5.35E-07 | down | 192 | 177 | 154 | 132 | 91 | 79 | LOC101104304 | + | Uncharacterized protein | 0 | ENOX2 |
| LIMCH1 | 1057.483528 | 2454.907765 | -1.21503 | 5.40E-07 | down | 3025 | 1777 | 2570 | 1727 | 830 | 785 | LIMCH1 | + | Uncharacterized protein | 0 | LIMCH1 |
| RPL35 | 2338.332608 | 1557.559608 | 0.586193 | 6.20E-07 | up | 1902 | 1406 | 1330 | 2525 | 2299 | 2295 | RPL35 | + | Uncharacterized protein | 4.00E-44 | RPL35 |
| LOC101121401 | 1662.01817 | 672.8735819 | 1.304529 | 6.46E-07 | up | 535 | 638 | 786 | 1667 | 1531 | 1826 | LOC101121401 | + | ferritin, heavy polypeptide 1 pseudogene | | |
| DMPK | 332.1328684 | 593.0966781 | -0.83651 | 6.67E-07 | down | 707 | 510 | 550 | 482 | 234 | 318 | DMPK | - | Uncharacterized protein | 0 | DMPK |
| SLC37A4 | 206.2106155 | 601.112271 | -1.54352 | 6.98E-07 | down | 517 | 635 | 595 | 242 | 166 | 222 | SLC37A4 | - | Uncharacterized protein | 0 | SLC37A4 |
| LOC101117181 | 756.351114 | 1264.263201 | -0.74117 | 7.09E-07 | down | 1401 | 1031 | 1327 | 703 | 883 | 699 | LOC101117181 | + | cytochrome c oxidase copper chaperone-like | | |
| C2H9orf41 | 49.29730265 | 336.8750633 | -2.77263 | 7.10E-07 | down | 350 | 194 | 465 | 39 | 68 | 41 | C2H9orf41 | + | chromosome 2 open reading frame, human C9orf41 | 9.00E-166 | C9orf41 |
| P2RY1 | 99.97576016 | 416.4010463 | -2.05832 | 7.22E-07 | down | 444 | 261 | 541 | 142 | 78 | 91 | P2RY1 | - | Uncharacterized protein | 0 | P2RY1 |
| TPI1 | 8725.494231 | 19951.72263 | -1.1932 | 7.99E-07 | down | 22201 | 20767 | 15772 | 9762 | 8042 | 8806 | TPI1 | - | Triosephosphate isomerase | 9.00E-120 | TPI1 |
| CCND3 | 227.7056069 | 125.6076995 | 0.858245 | 8.35E-07 | up | 125 | 121 | 123 | 286 | 183 | 231 | CCND3 | - | Ccnd3 | 3.00E-167 | CCND3 |
| EXTL3 | 88.08196825 | 166.6563522 | -0.91996 | 8.61E-07 | down | 183 | 138 | 174 | 135 | 64 | 77 | EXTL3 | + | Uncharacterized protein | 0 | EXTL3 |
| NT5C1A | 348.2609579 | 623.9887807 | -0.84135 | 8.75E-07 | down | 616 | 589 | 629 | 327 | 360 | 363 | NT5C1A | - | Uncharacterized protein | 0 | NT5C1A |
| ADHFE1 | 233.0735066 | 522.9754838 | -1.16596 | 9.22E-07 | down | 482 | 488 | 563 | 195 | 254 | 249 | ADHFE1 | + | Uncharacterized protein | 0 | ADHFE1 |
| CCDC107 | 166.0135481 | 103.1309155 | 0.686824 | 9.85E-07 | up | 102 | 106 | 94 | 212 | 147 | 153 | CCDC107 | - | coiled-coil domain containing 107 | 1.00E-80 | CCDC107 |
| SIK1 | 1015.407948 | 275.4098403 | 1.882407 | 9.87E-07 | up | 525 | 285 | 32 | 1189 | 824 | 1089 | SIK1 | - | Uncharacterized protein | 0 | SIK1 |
| PDP1 | 263.4641878 | 725.01642 | -1.46041 | 9.88E-07 | down | 861 | 537 | 773 | 500 | 178 | 169 | PDP1 | - | Uncharacterized protein | 0 | np_001155250 |
| XIRP1 | 37631.66032 | 7518.661738 | 2.323399 | 1.11E-06 | up | 12165 | 8405 | 2027 | 47714 | 25168 | 42592 | XIRP1 | - | xin actin-binding repeat containing 1 | 0 | XIRP1 |
| SCARB2 | 252.6875763 | 391.0163417 | -0.62987 | 1.14E-06 | down | 440 | 323 | 400 | 330 | 272 | 182 | SCARB2 | - | Uncharacterized protein | 0 | SCARB2 |
| PLXDC1 | 106.322652 | 550.2644261 | -2.37168 | 1.16E-06 | down | 853 | 362 | 467 | 148 | 50 | 130 | PLXDC1 | - | Uncharacterized protein | 0 | PLXDC1 |
| KIAA1161 | 214.7233015 | 661.5827602 | -1.62344 | 1.19E-06 | down | 721 | 716 | 505 | 278 | 124 | 257 | KIAA1161 | + | KIAA1161 ortholog | 0 | KIAA1161 |
| AMPD3 | 1828.12717 | 115.0175341 | 3.990441 | 1.21E-06 | up | 176 | 70 | 106 | 1093 | 2271 | 2027 | AMPD3 | - | Uncharacterized protein | 0 | AMPD3 |
| GPX1 | 761.868891 | 1529.089222 | -1.00506 | 1.22E-06 | down | 1654 | 1441 | 1420 | 773 | 556 | 967 | GPX1 | + | Glutathione peroxidase | 1.00E-98 | GPX1 |
| PTPLA | 241.1555198 | 459.766792 | -0.93094 | 1.43E-06 | down | 537 | 344 | 494 | 256 | 186 | 288 | PTPLA | - | Very-long-chain (3R)-3-hydroxyacyl-CoA dehydratase 1 | 3.00E-149 | PTPLA |
| LOC101120261 | 328.3663066 | 551.7438329 | -0.74869 | 1.48E-06 | down | 535 | 523 | 562 | 417 | 288 | 307 | LOC101120261 | - | up-regulated during skeletal muscle growth protein 5-like | 4.00E-27 | USMG5 |
| GLT25D2 | 159.4510632 | 68.46755948 | 1.219621 | 1.51E-06 | up | 78 | 70 | 54 | 154 | 153 | 174 | GLT25D2 | - | glycosyltransferase 25 domain containing 2 | 0 | COLGALT2 |

| | | | | | | | | | | | | | | | | |
|---|---|---|---|---|---|---|---|---|---|---|---|---|---|---|---|---|
| MLF1 | 335.5333612 | 1449.724032 | -2.11125 | 1.59E-06 | down | 1432 | 976 | 1905 | 508 | 245 | 297 | MLF1 | - | Uncharacterized protein | 3.00E-124 | MLF1 |
| GOSR2 | 435.3593011 | 287.9412885 | 0.596432 | 1.70E-06 | up | 261 | 287 | 293 | 523 | 433 | 382 | GOSR2 | - | Golgi SNAP receptor complex member 2 | 3.00E-105 | GOSR2 |
| SERF2 | 2123.67984 | 3686.378994 | -0.79564 | 1.86E-06 | down | 4026 | 3575 | 3276 | 2356 | 1749 | 2354 | SERF2 | + | small EDRK-rich factor 2 | 3.00E-70 | SERF2 |
| PLEKHH3 | 33.83272743 | 211.0478479 | -2.64108 | 1.95E-06 | down | 284 | 222 | 122 | 63 | 15 | 30 | PLEKHH3 | - | Uncharacterized protein | 0 | PLEKHH3 |
| ARHGAP23 | 105.6823735 | 266.8226841 | -1.33615 | 1.96E-06 | down | 249 | 276 | 254 | 151 | 67 | 110 | ARHGAP23 | + | Uncharacterized protein | 0 | ARHGAP23 |
| SUFU | 95.49851424 | 172.5445412 | -0.85342 | 2.08E-06 | down | 180 | 144 | 187 | 136 | 72 | 89 | SUFU | + | Uncharacterized protein | 0 | SUFU |
| BOLA3 | 172.8298721 | 317.8942369 | -0.87919 | 2.21E-06 | down | 339 | 254 | 351 | 206 | 127 | 195 | BOLA3 | - | bolA homolog 3 (E. coli) | 7.00E-45 | BOLA3 |
| HNRNPR | 436.2458922 | 286.3046455 | 0.60759 | 2.21E-06 | up | 356 | 224 | 278 | 570 | 428 | 353 | HNRNPR | + | Uncharacterized protein | 0 | HNRNPR |
| FAM78A | 501.2747381 | 1620.160269 | -1.69246 | 2.26E-06 | down | 1531 | 1410 | 1827 | 473 | 198 | 821 | FAM78A | + | family with sequence similarity 78, member A | 2.00E-152 | FAM78A |
| CDCA7 | 117.9655556 | 28.30310534 | 2.059333 | 2.37E-06 | up | 11 | 10 | 62 | 120 | 109 | 128 | CDCA7 | - | Uncharacterized protein | 0 | CDCA7 |
| KERA | 14.107212 | 51.96737369 | -1.88117 | 2.50E-06 | down | 56 | 37 | 62 | 27 | 3 | 15 | KERA | - | Uncharacterized protein | 0 | KERA |
| TMEM179B | 154.2568324 | 92.45280009 | 0.738545 | 2.66E-06 | up | 125 | 76 | 77 | 167 | 156 | 147 | TMEM179B | + | transmembrane protein 179B | 7.00E-80 | TMEM179B |
| RRAS2 | 147.4988257 | 63.47636894 | 1.216412 | 2.77E-06 | up | 55 | 56 | 75 | 166 | 119 | 164 | RRAS2 | + | Uncharacterized protein | 5.00E-97 | RRAS2 |
| RXRG | 273.7361288 | 1104.109533 | -2.01203 | 2.89E-06 | down | 1254 | 1234 | 755 | 379 | 145 | 321 | RXRG | - | Uncharacterized protein | 0 | RXRG |
| NEO1 | 178.1191839 | 390.0595363 | -1.13085 | 2.90E-06 | down | 471 | 267 | 434 | 248 | 124 | 180 | NEO1 | + | Uncharacterized protein | 0 | NEO1 |
| JARID2 | 77.55883949 | 167.0263217 | -1.10671 | 2.91E-06 | down | 206 | 133 | 161 | 131 | 59 | 56 | JARID2 | - | Uncharacterized protein | 0 | JARID2 |
| THOC5 | 853.3970252 | 513.8243712 | 0.731942 | 3.11E-06 | up | 542 | 486 | 487 | 910 | 783 | 899 | THOC5 | - | Uncharacterized protein | 0 | THOC5 |
| KIAA0100 | 777.2664819 | 1221.433127 | -0.65209 | 3.15E-06 | down | 1268 | 1106 | 1231 | 1075 | 652 | 687 | KIAA0100 | - | KIAA0100 ortholog | 0 | KIAA0100 |
| LOC101122981 | 4034.989413 | 6312.47709 | -0.64564 | 3.19E-06 | down | 7369 | 5901 | 5452 | 4159 | 3910 | 4166 | LOC101122981 | + | tumor protein, translationally-controlled 1 pseudogene | | |
| ARMC12 | 76.93863198 | 5.165218312 | 3.896807 | 3.33E-06 | up | 3 | 4 | 8 | 66 | 55 | 108 | ARMC12 | + | Uncharacterized protein | 4.00E-147 | ARMC12 |
| SH3GLB1 | 716.8303903 | 360.1106385 | 0.993192 | 3.39E-06 | up | 416 | 234 | 431 | 758 | 807 | 620 | SH3GLB1 | + | Uncharacterized protein | 0 | SH3GLB1 |
| MYOD1 | 37.50764368 | 232.4596229 | -2.63172 | 3.46E-06 | down | 195 | 275 | 201 | 86 | 9 | 28 | MYOD1 | - | Myoblast determination protein 1 | 1.00E-152 | MYOD1 |
| WNK2 | 1479.645644 | 2703.294233 | -0.86947 | 3.55E-06 | down | 2782 | 2608 | 2564 | 1553 | 1207 | 1719 | WNK2 | - | Uncharacterized protein | 0 | WNK2 |
| EIF4EBP1 | 1794.296709 | 734.2069564 | 1.28916 | 3.76E-06 | up | 766 | 835 | 544 | 1762 | 1683 | 1972 | EIF4EBP1 | + | Uncharacterized protein | 8.00E-59 | EIF4EBP1 |
| SPP1 | 242.2902196 | 2.935474933 | 6.366998 | 3.79E-06 | up | 10 | 0 | 0 | 8 | 401 | 280 | SPP1 | - | Osteopontin | 7.00E-78 | SPP1 |
| HADH | 1967.372451 | 3165.430494 | -0.68613 | 3.81E-06 | down | 3181 | 3091 | 3026 | 2374 | 1734 | 1927 | HADH | - | Uncharacterized protein | 2.00E-165 | HADH |
| PLA2G15 | 105.4887903 | 51.96641989 | 1.021438 | 4.04E-06 | up | 45 | 63 | 42 | 125 | 92 | 106 | PLA2G15 | + | Uncharacterized protein | 0 | PLA2G15 |
| LOC101120019 | 89.28175646 | 42.4721856 | 1.071847 | 4.14E-06 | up | 61 | 29 | 39 | 112 | 97 | 67 | LOC101120019 | + | Ribosomal protein | 2.00E-84 | RPL10A |
| XPO4 | 232.8144216 | 361.9606154 | -0.63665 | 4.23E-06 | down | 446 | 307 | 328 | 317 | 229 | 178 | XPO4 | + | Uncharacterized protein | 0 | XPO4 |
| GPRC5C | 288.0325192 | 545.0056056 | -0.92004 | 4.32E-06 | down | 648 | 495 | 477 | 372 | 186 | 327 | GPRC5C | - | Uncharacterized protein | 0 | GPRC5C |
| HDAC4 | 175.0001565 | 81.82322269 | 1.096774 | 4.33E-06 | up | 80 | 65 | 97 | 170 | 198 | 162 | HDAC4 | + | Histone deacetylase | 0 | HDAC4 |
| FAM53B | 95.35164475 | 204.6950275 | -1.10215 | 4.37E-06 | down | 213 | 187 | 204 | 87 | 68 | 130 | FAM53B | - | family with sequence similarity 53, | 4.00E-108 | FAM53B |

| | | | | | | | | | | | | | | | | |
|---|---|---|---|---|---|---|---|---|---|---|---|---|---|---|---|---|
| | | | | | | | | | | | | | | member B | | |
| LOC101112102 | 592.2116452 | 335.0061164 | 0.821925 | 4.38E-06 | up | 390 | 324 | 278 | 745 | 640 | 446 | LOC101112102 | + | Ribosomal protein | 7.00E-109 | RPL10A |
| MGST3 | 541.3641374 | 957.6110707 | -0.82284 | 4.54E-06 | down | 988 | 780 | 1069 | 642 | 428 | 585 | MGST3 | + | Uncharacterized protein | 6.00E-78 | MGST3 |
| GLRX5 | 613.4051571 | 1634.164859 | -1.41364 | 4.55E-06 | down | 1711 | 1693 | 1393 | 766 | 305 | 802 | GLRX5 | + | Uncharacterized protein | 5.00E-63 | GLRX5 |
| GREB1 | 39.09401383 | 9.259139586 | 2.077998 | 4.59E-06 | up | 14 | 8 | 6 | 32 | 44 | 41 | GREB1 | + | growth regulation by estrogen in breast cancer 1 | 0 | GREB1 |
| ITGAE | 27.6261905 | 62.03996566 | -1.16716 | 4.72E-06 | down | 64 | 49 | 71 | 27 | 36 | 21 | ITGAE | - | Uncharacterized protein | 0 | ITGAE |
| LOC101104557 | 859.0296977 | 413.5879643 | 1.054514 | 5.11E-06 | up | 470 | 272 | 498 | 1168 | 861 | 643 | LOC101104557 | + | Uncharacterized protein | 0 | AOX1 |
| IGDCC4 | 167.0726392 | 571.1539412 | -1.7734 | 5.27E-06 | down | 642 | 545 | 502 | 369 | 30 | 145 | IGDCC4 | - | immunoglobulin superfamily, DCC subclass, member 4 | 0 | IGDCC4 |
| CDH4 | 116.1155245 | 49.8086945 | 1.221091 | 5.35E-06 | up | 26 | 63 | 52 | 134 | 106 | 115 | CDH4 | - | Uncharacterized protein | 0 | CDH4 |
| LOC100037670 | 1182.271709 | 666.8458198 | 0.826136 | 5.47E-06 | up | 661 | 753 | 530 | 1527 | 1140 | 989 | LOC100037670 | + | uncharacterized LOC100037670 | | |
| CEBPB | 1512.302261 | 538.5327709 | 1.48964 | 5.81E-06 | up | 634 | 730 | 204 | 1509 | 1512 | 1556 | CEBPB | + | CCAAT/enhancer binding protein beta | 4.00E-55 | CEBPB |
| CDV3 | 183.0528756 | 116.5430414 | 0.651398 | 5.83E-06 | up | 126 | 88 | 133 | 198 | 190 | 170 | CDV3 | - | Uncharacterized protein | 3.00E-88 | CDV3 |
| ATPIF1 | 740.3536433 | 1225.738404 | -0.72736 | 5.87E-06 | down | 1264 | 1212 | 1127 | 952 | 626 | 705 | ATPIF1 | - | Uncharacterized protein | 4.00E-23 | ATPIF1 |
| MYLK3 | 94.11932658 | 265.7931307 | -1.49774 | 6.03E-06 | down | 292 | 201 | 299 | 183 | 54 | 66 | MYLK3 | - | Uncharacterized protein | 0 | MYLK3 |
| ZIC1 | 114.717677 | 213.9152849 | -0.89895 | 6.25E-06 | down | 225 | 192 | 215 | 117 | 86 | 143 | ZIC1 | - | Uncharacterized protein | 0 | ZIC1 |
| SLC2A12 | 61.54533187 | 157.6004315 | -1.35655 | 6.41E-06 | down | 162 | 127 | 178 | 110 | 37 | 49 | SLC2A12 | - | Uncharacterized protein | 0 | SLC2A12 |
| LCP1 | 226.1068491 | 388.9826224 | -0.7827 | 6.62E-06 | down | 477 | 363 | 317 | 299 | 221 | 181 | LCP1 | - | Uncharacterized protein | 0 | LCP1 |
| CA3 | 15687.05914 | 54091.28904 | -1.78582 | 6.94E-06 | down | 50313 | 61004 | 45913 | 14090 | 17423 | 15710 | CA3 | - | Uncharacterized protein | 3.00E-135 | CA3 |
| C18H14orf28 | 22.74155029 | 54.46721986 | -1.26006 | 7.42E-06 | down | 67 | 36 | 61 | 32 | 23 | 16 | C18H14orf28 | + | chromosome 18 open reading frame, human C14orf28 | 0 | C14orf28 |
| MSS51 | 848.5814506 | 2847.253308 | -1.74645 | 7.60E-06 | down | 2461 | 3005 | 2811 | 1122 | 365 | 1115 | MSS51 | - | Uncharacterized protein | 0 | MSS51 |
| ABR | 223.3755156 | 365.2826512 | -0.70954 | 7.70E-06 | down | 429 | 332 | 324 | 322 | 191 | 184 | ABR | + | Uncharacterized protein | 0 | ABR |
| OLFML2B | 90.60914137 | 292.381758 | -1.69012 | 7.82E-06 | down | 389 | 182 | 315 | 151 | 38 | 96 | OLFML2B | - | Uncharacterized protein | 0 | OLFML2B |
| ALKBH5 | 1301.584207 | 2247.439539 | -0.78801 | 8.04E-06 | down | 2553 | 2151 | 1945 | 1312 | 1151 | 1470 | ALKBH5 | - | Uncharacterized protein | 4.00E-155 | ALKBH5 |
| FAM165B | 205.7932855 | 324.3209829 | -0.65623 | 8.08E-06 | down | 406 | 241 | 327 | 258 | 173 | 202 | FAM165B | - | family with sequence similarity 165, member B | 4.00E-23 | SMIM11 |
| KCTD15 | 432.495795 | 889.5598049 | -1.04041 | 8.15E-06 | down | 1085 | 778 | 789 | 499 | 262 | 553 | KCTD15 | + | Uncharacterized protein | 2.00E-165 | KCTD15 |
| SMPDL3A | 96.04858704 | 25.04834202 | 1.939049 | 8.37E-06 | up | 24 | 20 | 30 | 153 | 81 | 69 | SMPDL3A | - | Uncharacterized protein | 0 | SMPDL3A |
| FOXS1 | 716.0702128 | 171.6611551 | 2.060537 | 8.46E-06 | up | 77 | 241 | 163 | 1041 | 461 | 725 | FOXS1 | - | Uncharacterized protein | 3.00E-146 | FOXS1 |
| LIN52 | 77.275749 | 196.3380557 | -1.34525 | 9.50E-06 | down | 181 | 157 | 241 | 99 | 62 | 77 | LIN52 | + | Uncharacterized protein | 7.00E-62 | LIN52 |
| GPI | 8221.674045 | 23688.58373 | -1.52669 | 9.52E-06 | down | 32740 | 20856 | 17552 | 10058 | 5779 | 9324 | GPI | + | Glucose-6-phosphate isomerase | 0 | GPI |
| PIAS3 | 195.8198874 | 305.2021731 | -0.64024 | 1.00E-05 | down | 348 | 293 | 262 | 244 | 165 | 193 | PIAS3 | - | Uncharacterized protein | 0 | PIAS3 |
| ZNF827 | 23.25806677 | 59.12931314 | -1.34614 | 1.01E-05 | down | 78 | 45 | 55 | 41 | 15 | 18 | ZNF827 | + | Uncharacterized protein | 0 | ZNF827 |

| | | | | | | | | | | | | | | | | |
|---|---|---|---|---|---|---|---|---|---|---|---|---|---|---|---|---|
| SLC8A3 | 216.9263439 | 344.0905334 | -0.66558 | 1.03E-05 | down | 356 | 307 | 353 | 305 | 184 | 186 | SLC8A3 | - | Uncharacterized protein | 0 | SLC8A3 |
| WIPF3 | 326.3358783 | 191.9183343 | 0.765865 | 1.11E-05 | up | 203 | 166 | 199 | 391 | 265 | 343 | WIPF3 | - | Uncharacterized protein | 3.00E-65 | WIPF3 |
| PPP1R1B | 53.11013624 | 152.0764303 | -1.51774 | 1.20E-05 | down | 212 | 134 | 111 | 83 | 42 | 42 | PPP1R1B | + | Uncharacterized protein | 3.00E-90 | PPP1R1B |
| MED22 | 206.4549181 | 118.7778011 | 0.797562 | 1.21E-05 | up | 135 | 79 | 142 | 244 | 180 | 208 | MED22 | + | Uncharacterized protein | 6.00E-75 | MED22 |
| DKK2 | 47.34898322 | 440.4565033 | -3.21759 | 1.32E-05 | down | 733 | 263 | 361 | 121 | 25 | 13 | DKK2 | + | Uncharacterized protein | 2.00E-136 | DKK2 |
| CPQ | 85.4946817 | 149.9105799 | -0.8102 | 1.33E-05 | down | 183 | 108 | 159 | 117 | 84 | 65 | CPQ | - | Uncharacterized protein | 0 | CPQ |
| GOLGB1 | 322.2172032 | 181.8633229 | 0.825179 | 1.37E-05 | up | 215 | 138 | 191 | 378 | 366 | 247 | GOLGB1 | - | Uncharacterized protein | 0 | GOLGB1 |
| AFMID | 30.6704503 | 7.416910928 | 2.047959 | 1.40E-05 | up | 5 | 3 | 14 | 38 | 22 | 34 | AFMID | - | Kynurenine formamidase | 7.00E-140 | AFMID |
| LOC101105598 | 878.2393951 | 1521.833798 | -0.79312 | 1.50E-05 | down | 1476 | 1332 | 1675 | 1169 | 833 | 721 | LOC101105598 | + | cytochrome c oxidase subunit 7C, mitochondrial-like | | |
| LOC101106131 | 3147.297014 | 5807.240663 | -0.88374 | 1.52E-05 | down | 5558 | 5577 | 5896 | 3173 | 2907 | 3438 | LOC101106131 | - | Cytochrome c oxidase subunit 6A, mitochondrial | 5.00E-34 | COX6A2 |
| TNFAIP2 | 65.65281677 | 149.8111372 | -1.19022 | 1.58E-05 | down | 208 | 125 | 118 | 88 | 51 | 64 | TNFAIP2 | + | tumor necrosis factor, alpha-induced protein 2 | 1.00E-137 | TNFAIP2 |
| FOXO1 | 1696.6063 | 203.6181372 | 3.058714 | 1.62E-05 | up | 296 | 184 | 133 | 1166 | 2232 | 1648 | FOXO1 | + | Uncharacterized protein | 0 | FOXO1 |
| ING2 | 59.03995468 | 138.1332482 | -1.2263 | 1.68E-05 | down | 178 | 122 | 113 | 81 | 30 | 71 | ING2 | + | Inhibitor of growth protein | 1.00E-140 | ING2 |
| AHCYL2 | 646.3219566 | 1451.630306 | -1.16735 | 1.69E-05 | down | 1397 | 1472 | 1379 | 989 | 456 | 579 | AHCYL2 | + | Adenosylhomocysteinase | 0 | AHCYL2 |
| TGM1 | 7.042394423 | 63.03679543 | -3.16206 | 1.82E-05 | down | 97 | 61 | 32 | 10 | 7 | 5 | TGM1 | + | Uncharacterized protein | 0 | TGM1 |
| REEP1 | 79.0183257 | 251.189764 | -1.66852 | 1.84E-05 | down | 225 | 192 | 324 | 86 | 66 | 88 | REEP1 | - | Uncharacterized protein | 1.00E-88 | REEP1 |
| KRCC1 | 295.3115328 | 176.0561954 | 0.746202 | 1.85E-05 | up | 154 | 156 | 206 | 316 | 300 | 283 | KRCC1 | - | lysine-rich coiled-coil 1 | 7.00E-103 | KRCC1 |
| CDKN2C | 171.2298884 | 463.3919251 | -1.4363 | 1.92E-05 | down | 387 | 445 | 519 | 196 | 122 | 203 | CDKN2C | + | Uncharacterized protein | 4.00E-80 | CDKN2C |
| COL15A1 | 1217.561699 | 2928.948733 | -1.26639 | 1.95E-05 | down | 4100 | 2007 | 2773 | 1635 | 939 | 1191 | COL15A1 | - | Uncharacterized protein | 0 | COL15A1 |
| DLAT | 812.4394665 | 1468.41131 | -0.85392 | 1.97E-05 | down | 1442 | 1430 | 1437 | 1153 | 697 | 681 | DLAT | + | Acetyltransferase component of pyruvate dehydrogenase complex | 0 | DLAT |
| CCDC137 | 151.1281116 | 65.10043584 | 1.215033 | 2.01E-05 | up | 87 | 43 | 67 | 150 | 180 | 129 | CCDC137 | - | Uncharacterized protein | 8.00E-109 | CCDC137 |
| EPS15 | 274.5702472 | 527.9643546 | -0.94327 | 2.07E-05 | down | 661 | 359 | 570 | 378 | 257 | 219 | EPS15 | - | Uncharacterized protein | 0 | EPS15 |
| B3GALNT1 | 795.6170577 | 247.9427724 | 1.682067 | 2.11E-05 | up | 223 | 171 | 340 | 1036 | 940 | 497 | B3GALNT1 | + | Uncharacterized protein | 0 | B3GALNT1 |
| CLIP1 | 2437.235435 | 1621.667124 | 0.587768 | 2.16E-05 | up | 1743 | 1194 | 1894 | 2841 | 2516 | 2119 | CLIP1 | + | Uncharacterized protein | 0 | CLIP1 |
| NDUFA6 | 270.6188406 | 445.0870151 | -0.71783 | 2.20E-05 | down | 430 | 419 | 458 | 276 | 280 | 265 | NDUFA6 | - | Uncharacterized protein | 3.00E-65 | NDUFA6 |
| LOC101111642 | 231.3327545 | 471.4914644 | -1.02726 | 2.28E-05 | down | 469 | 417 | 504 | 234 | 314 | 158 | LOC101111642 | + | Uncharacterized protein | 8.00E-167 | SLC25A30 |
| TMEM51 | 44.35254902 | 90.93242685 | -1.03578 | 2.35E-05 | down | 124 | 64 | 87 | 42 | 49 | 43 | TMEM51 | - | transmembrane protein 51 | 2.00E-113 | TMEM51 |
| DHDH | 381.7764968 | 941.3623591 | -1.30202 | 2.38E-05 | down | 1210 | 923 | 669 | 381 | 342 | 430 | DHDH | + | Uncharacterized protein | 2.00E-172 | DHDH |
| SSC5D | 18.67336649 | 70.1559433 | -1.90958 | 2.41E-05 | down | 104 | 52 | 57 | 36 | 10 | 14 | SSC5D | - | Uncharacterized protein | 2.00E-171 | SSC5D |
| SETD8 | 355.3638221 | 777.9235013 | -1.13033 | 2.45E-05 | down | 693 | 801 | 773 | 463 | 313 | 322 | SETD8 | - | Uncharacterized protein | 6.00E-174 | SETD8 |
| NDUFA4 | 1781.124056 | 2812.772422 | -0.6592 | 2.49E-05 | down | 2732 | 2548 | 2995 | 1976 | 1690 | 1765 | NDUFA4 | - | NADH dehydrogenase (ubiquinone) 1 alpha subcomplex, | 8.00E-38 | NDUFA4 |

| | | | | | | | | | | | | | | | | |
|---|---|---|---|---|---|---|---|---|---|---|---|---|---|---|---|---|
| | | | | | | | | | | | | | | 4, 9kDa | | |
| FMO2 | 38.43651518 | 7.513751338 | 2.354872 | 2.54E-05 | up | 3 | 3 | 16 | 33 | 37 | 45 | FMO2 | + | Dimethylaniline monooxygenase [N-oxide-forming] | 0 | FMO2 |
| CDKN1A | 532.8482013 | 110.6358942 | 2.267905 | 2.63E-05 | up | 181 | 107 | 47 | 438 | 501 | 650 | CDKN1A | + | CDKN1A | 6.00E-71 | CDKN1A |
| ALDH2 | 7973.550842 | 15161.7522 | -0.92714 | 2.65E-05 | down | 17634 | 13641 | 13754 | 6285 | 7844 | 9608 | ALDH2 | - | Uncharacterized protein | 0 | ALDH2 |
| LOC101102999 | 12727.70563 | 2629.161886 | 2.275297 | 2.66E-05 | up | 3375 | 2496 | 1965 | 10432 | 16379 | 11411 | LOC101102999 | - | Glutamine synthetase | 0 | GLUL |
| LOC101109747 | 958.755325 | 1885.227579 | -0.9755 | 2.68E-05 | down | 2230 | 1766 | 1599 | 1460 | 938 | 619 | LOC101109747 | - | MHC class II antigen | 8.00E-112 | HLA-DRA |
| ADAMTS8 | 0.731710296 | 20.5048853 | -4.80855 | 2.85E-05 | down | 28 | 7 | 28 | 0 | 0 | 2 | ADAMTS8 | - | Uncharacterized protein | 0 | ADAMTS8 |
| SREBF2 | 520.8913309 | 877.6665685 | -0.75269 | 2.94E-05 | down | 844 | 877 | 849 | 608 | 522 | 467 | SREBF2 | + | Uncharacterized protein | 0 | SREBF1 |
| MLH3 | 124.9033324 | 69.45657043 | 0.846629 | 2.95E-05 | up | 85 | 54 | 69 | 141 | 144 | 98 | MLH3 | - | Uncharacterized protein | 0 | MLH3 |
| LOC101106123 | 59.58520928 | 202.5474524 | -1.76523 | 2.96E-05 | down | 272 | 180 | 155 | 57 | 15 | 105 | LOC101106123 | + | dynein light chain 1, cytoplasmic-like | 5.00E-49 | DYNLL1 |
| BOP1 | 254.8108761 | 124.9422278 | 1.028166 | 2.97E-05 | up | 151 | 119 | 101 | 244 | 290 | 237 | BOP1 | + | Uncharacterized protein | 2.00E-138 | BOP1 |
| MCOLN1 | 455.8729054 | 253.1306818 | 0.848749 | 3.01E-05 | up | 231 | 264 | 243 | 458 | 465 | 458 | MCOLN1 | + | Uncharacterized protein | 0 | MCOLN1 |
| SNCG | 11.23408374 | 34.82723341 | -1.63233 | 3.09E-05 | down | 39 | 33 | 31 | 18 | 2 | 15 | SNCG | + | Gamma-synuclein | 2.00E-48 | SNCG |
| PPT1 | 259.8080597 | 405.2813439 | -0.64148 | 3.24E-05 | down | 423 | 348 | 428 | 352 | 263 | 193 | PPT1 | - | Palmitoyl protein thioesterase | | |
| GRIP2 | 81.07035042 | 271.9230085 | -1.74595 | 3.26E-05 | down | 349 | 269 | 191 | 157 | 49 | 55 | GRIP2 | + | Uncharacterized protein | 0 | GRIP2 |
| LOC101120042 | 247.1945317 | 445.9040708 | -0.85109 | 3.43E-05 | down | 453 | 439 | 418 | 228 | 234 | 281 | LOC101120042 | + | uncharacterized LOC101120042 | | |
| HPCAL4 | 87.27437847 | 41.49087573 | 1.072764 | 3.62E-05 | up | 34 | 54 | 31 | 113 | 85 | 72 | HPCAL4 | - | Uncharacterized protein | 3.00E-110 | HPCAL4 |
| DNAJA2 | 1338.71884 | 865.3882548 | 0.629434 | 3.70E-05 | up | 961 | 625 | 998 | 1733 | 1199 | 1203 | DNAJA2 | - | Uncharacterized protein | 0 | DNAJA2 |
| FAM13A | 919.576508 | 154.2369111 | 2.575822 | 3.76E-05 | up | 200 | 116 | 148 | 687 | 892 | 1149 | FAM13A | + | family with sequence similarity 13, member A | 0 | FAM13A |
| ORAI1 | 194.3727603 | 454.564658 | -1.22566 | 3.90E-05 | down | 464 | 423 | 452 | 313 | 84 | 212 | ORAI1 | - | ORAI calcium release-activated calcium modulator 1 | 4.00E-133 | ORAI1 |
| CCDC146 | 50.04095866 | 6.817098572 | 2.87588 | 3.93E-05 | up | 7 | 7 | 6 | 82 | 52 | 25 | CCDC146 | - | coiled-coil domain containing 146 | 0 | xp_003846748 |
| PNPLA2 | 2151.002912 | 1018.810065 | 1.078124 | 4.10E-05 | up | 862 | 1242 | 833 | 2179 | 2318 | 2031 | PNPLA2 | - | Uncharacterized protein | 5.00E-113 | PNPLA2 |
| EPHB2 | 21.54870428 | 80.25278069 | -1.89695 | 4.19E-05 | down | 114 | 44 | 87 | 35 | 16 | 17 | EPHB2 | - | Receptor protein-tyrosine kinase | 0 | EPHB2 |
| ZNHIT2 | 33.35579715 | 8.353368527 | 1.997508 | 4.45E-05 | up | 3 | 14 | 6 | 29 | 34 | 37 | ZNHIT2 | - | zinc finger, HIT-type containing 2 | 1.00E-97 | ZNHIT2 |
| CBFB | 125.1860522 | 58.97968388 | 1.085784 | 4.56E-05 | up | 92 | 34 | 55 | 129 | 128 | 123 | CBFB | + | Uncharacterized protein | 3.00E-73 | CBFB |
| GBGT1 | 92.9255825 | 49.6545435 | 0.90415 | 4.68E-05 | up | 59 | 42 | 47 | 93 | 88 | 100 | GBGT1 | + | Uncharacterized protein | 5.00E-167 | GBGT1 |
| ZNRF1 | 160.9587599 | 330.0564928 | -1.03602 | 5.23E-05 | down | 333 | 335 | 300 | 182 | 111 | 196 | ZNRF1 | + | Uncharacterized protein | 4.00E-81 | ZNRF1 |
| TEX2 | 1366.262109 | 2423.430393 | -0.82682 | 5.26E-05 | down | 2697 | 2121 | 2370 | 1787 | 864 | 1551 | TEX2 | - | Uncharacterized protein | 0 | TEX2 |
| C23H18orf21 | 85.43930093 | 40.7437303 | 1.068322 | 5.38E-05 | up | 43 | 32 | 46 | 105 | 99 | 60 | C23H18orf21 | - | chromosome 23 open reading frame, human C18orf21 | 2.00E-100 | C18orf21 |
| PRUNE | 160.9679687 | 297.3121289 | -0.8852 | 5.77E-05 | down | 270 | 277 | 324 | 186 | 144 | 162 | PRUNE | + | Uncharacterized protein | 0 | PRUNE |
| ADAMTS16 | 23.63000881 | 144.2434612 | -2.60981 | 5.82E-05 | down | 237 | 101 | 104 | 42 | 11 | 22 | ADAMTS16 | - | Uncharacterized protein | 0 | ADAMTS16 |

| | | | | | | | | | | | | | | | | |
|---|---|---|---|---|---|---|---|---|---|---|---|---|---|---|---|---|
| P2RX4 | 149.889564 | 94.13885239 | 0.671038 | 5.97E-05 | up | 99 | 93 | 85 | 160 | 165 | 132 | P2RX4 | - | P2X purinoceptor | 0 | P2RX4 |
| LOC101123010 | 109.3193571 | 39.89243197 | 1.454362 | 6.25E-05 | up | 45 | 38 | 35 | 123 | 108 | 103 | LOC101123010 | - | Uncharacterized protein | 8.00E-37 | CSTB |
| CCDC28B | 63.20981955 | 136.4047105 | -1.10967 | 6.73E-05 | down | 173 | 86 | 153 | 85 | 57 | 54 | CCDC28B | - | coiled-coil domain containing 28B | 2.00E-76 | CCDC28B |
| ITGB6 | 37.49323185 | 220.6260286 | -2.5569 | 6.83E-05 | down | 314 | 103 | 259 | 77 | 11 | 33 | ITGB6 | + | Integrin beta-6 | 0 | ITGB6 |
| MAPK4 | 3.466351013 | 55.71449938 | -4.00656 | 6.88E-05 | down | 102 | 33 | 38 | 2 | 1 | 7 | MAPK4 | + | Uncharacterized protein | 0 | MAPK4 |
| CD2 | 13.7128468 | 49.97107646 | -1.86557 | 6.97E-05 | down | 67 | 50 | 32 | 19 | 18 | 6 | CD2 | + | Cluster of differentiation 2 | 3.00E-104 | CD2 |
| SLAN | 50.15033744 | 16.08577619 | 1.640474 | 7.00E-05 | up | 18 | 12 | 18 | 78 | 46 | 34 | SLAN | - | Acidic leucine-rich nuclear phosphoprotein 32 family member B | 1.00E-67 | ANP32B |
| LOC101114597 | 239.9817185 | 143.8067609 | 0.738793 | 7.27E-05 | up | 181 | 107 | 144 | 287 | 267 | 185 | LOC101114597 | - | heterogeneous nuclear ribonucleoprotein A1-like | 0 | HNRNPA1 |
| HEYL | 186.3627207 | 310.0585124 | -0.73443 | 7.27E-05 | down | 395 | 239 | 297 | 246 | 139 | 190 | HEYL | - | Uncharacterized protein | 1.00E-162 | HEYL |
| LOC101107597 | 2734.811678 | 1606.255191 | 0.76774 | 7.51E-05 | up | 1557 | 1584 | 1567 | 2785 | 3009 | 2513 | LOC101107597 | - | 60S ribosomal protein L10a-like | 5.00E-125 | RPL10A |
| LOC101112277 | 4197.512291 | 1913.865445 | 1.133045 | 7.58E-05 | up | 1618 | 2001 | 1942 | 4382 | 3638 | 4694 | LOC101112277 | + | GATS-like protein 2-like | 0 | GATSL2 |
| KCNIP2 | 68.77604939 | 173.2599992 | -1.33296 | 7.76E-05 | down | 245 | 116 | 165 | 107 | 52 | 57 | KCNIP2 | - | Uncharacterized protein | 2.00E-164 | KCNIP2 |
| SDHC | 1779.835217 | 3580.428756 | -1.00839 | 7.90E-05 | down | 3207 | 3501 | 3753 | 2265 | 1506 | 1712 | SDHC | + | Uncharacterized protein | 3.00E-82 | SDHC |
| AZIN1 | 274.5965905 | 730.7821807 | -1.41213 | 7.94E-05 | down | 989 | 552 | 663 | 510 | 166 | 203 | AZIN1 | + | Uncharacterized protein | 0 | AZIN1 |
| SCN3B | 59.55849537 | 3.60684604 | 4.045498 | 8.23E-05 | up | 6 | 3 | 2 | 58 | 93 | 31 | SCN3B | - | Uncharacterized protein | 2.00E-123 | SCN3B |
| LOC101107153 | 937.1214042 | 1656.476729 | -0.82181 | 8.43E-05 | down | 1608 | 1441 | 1832 | 880 | 978 | 968 | LOC101107153 | - | Uncharacterized protein | 5.00E-27 | COX17 |
| SNX24 | 28.42129288 | 69.36743109 | -1.28729 | 8.44E-05 | down | 67 | 63 | 74 | 37 | 12 | 38 | SNX24 | - | Uncharacterized protein | 6.00E-83 | SNX24 |
| ATG7 | 211.9301296 | 115.338895 | 0.87771 | 8.83E-05 | up | 149 | 86 | 112 | 244 | 245 | 162 | ATG7 | + | Uncharacterized protein | 0 | ATG7 |
| CISH | 746.8521088 | 330.8242686 | 1.174758 | 9.07E-05 | up | 428 | 242 | 326 | 803 | 912 | 569 | CISH | + | Uncharacterized protein | 3.00E-125 | CISH |
| BTG1 | 3228.473692 | 670.5420525 | 2.267453 | 9.08E-05 | up | 759 | 607 | 622 | 2392 | 4003 | 3232 | BTG1 | - | Uncharacterized protein | 2.00E-99 | BTG1 |
| IDH1 | 98.84113166 | 288.3936029 | -1.54486 | 9.51E-05 | down | 405 | 179 | 293 | 167 | 83 | 64 | IDH1 | - | Cytosolic NADP-isocitrate dehydrongenase | 0 | IDH1 |
| ASAP1 | 154.281237 | 93.65545107 | 0.720128 | 9.83E-05 | up | 128 | 68 | 87 | 205 | 142 | 131 | ASAP1 | + | Uncharacterized protein | 0 | ASAP1 |
| LOC101103815 | 1515.690965 | 3256.519886 | -1.10336 | 9.86E-05 | down | 3716 | 3296 | 2601 | 1497 | 1182 | 1884 | LOC101103815 | - | triosephosphate isomerase-like | | |
| SETD3 | 719.0181229 | 1297.087802 | -0.85118 | 1.02E-04 | down | 1191 | 1237 | 1370 | 860 | 620 | 723 | SETD3 | - | Uncharacterized protein | 0 | SETD3 |
| HLF | 31.70405346 | 76.33043792 | -1.26759 | 1.02E-04 | down | 98 | 46 | 87 | 42 | 26 | 30 | HLF | + | Hepatic leukemia factor | 4.00E-160 | HLF |
| PLTP | 136.5850096 | 325.7274415 | -1.25387 | 1.06E-04 | down | 293 | 325 | 333 | 223 | 113 | 96 | PLTP | - | Uncharacterized protein | 0 | PLTP |
| CALCOCO2 | 2311.252428 | 1079.881002 | 1.097802 | 1.06E-04 | up | 996 | 959 | 1217 | 2482 | 2782 | 1801 | CALCOCO2 | - | Uncharacterized protein | 0 | CALCOCO2 |
| WBP1L | 233.2562417 | 80.31201066 | 1.538228 | 1.08E-04 | up | 87 | 92 | 56 | 213 | 211 | 276 | WBP1L | + | WW domain binding protein 1-like | 1.00E-167 | WBP1L |
| LNX2 | 108.6435978 | 58.25472319 | 0.899156 | 1.09E-04 | up | 89 | 45 | 43 | 121 | 113 | 98 | LNX2 | + | Uncharacterized protein | 0 | LNX2 |
| ADIPOR2 | 3218.549745 | 997.4543171 | 1.690088 | 1.09E-04 | up | 735 | 1337 | 772 | 2748 | 3337 | 3556 | ADIPOR2 | + | Uncharacterized protein | 0 | ADIPOR2 |
| THOP1 | 1992.644454 | 684.3121371 | 1.541958 | 1.10E-04 | up | 747 | 702 | 565 | 1722 | 2426 | 1848 | THOP1 | - | Uncharacterized protein | 0 | THOP1 |

| | | | | | | | | | | | | | | | | |
|---|---|---|---|---|---|---|---|---|---|---|---|---|---|---|---|---|
| TNK2 | 317.3406313 | 599.4584595 | -0.91763 | 1.16E-04 | down | 753 | 526 | 511 | 415 | 201 | 360 | TNK2 | - | Uncharacterized protein | 0 | TNK2 |
| LOC101123462 | 70.95731015 | 35.13652453 | 1.01398 | 1.19E-04 | up | 43 | 29 | 33 | 67 | 73 | 74 | LOC101123462 | - | ataxin-7-like protein 1-like | 0 | ATXN7L1 |
| PVALB | 3.840531094 | 124.9236699 | -5.0236 | 1.20E-04 | down | 269 | 3 | 131 | 10 | 3 | 0 | PVALB | - | Uncharacterized protein | 4.00E-51 | PVALB |
| KY | 233.7512189 | 1386.519932 | -2.56842 | 1.21E-04 | down | 1699 | 1573 | 815 | 565 | 100 | 111 | KY | + | Uncharacterized protein | 0 | KY |
| KANK1 | 553.0456863 | 1253.544507 | -1.18054 | 1.22E-04 | down | 1641 | 930 | 1204 | 913 | 320 | 510 | KANK1 | + | Uncharacterized protein | 0 | KANK1 |
| LOC101107831 | 108.6542605 | 288.0198182 | -1.40642 | 1.28E-04 | down | 338 | 288 | 226 | 107 | 52 | 166 | LOC101107831 | + | glutathione S-transferase Mu 1-like | 4.00E-85 | GSTM1 |
| RNF207 | 9.324224356 | 77.54209703 | -3.05592 | 1.35E-04 | down | 117 | 78 | 38 | 23 | 3 | 5 | RNF207 | - | Uncharacterized protein | 0 | RNF207 |
| CDHR3 | 4.020272671 | 26.72956643 | -2.73307 | 1.35E-04 | down | 33 | 29 | 17 | 13 | 0 | 1 | CDHR3 | + | Uncharacterized protein | 0 | CDHR3 |
| PTPRT | 3.065328725 | 23.11835173 | -2.91492 | 1.41E-04 | down | 38 | 15 | 18 | 7 | 0 | 3 | PTPRT | - | Uncharacterized protein | 0 | PTPRT |
| SHMT2 | 787.4430426 | 516.171291 | 0.609326 | 1.45E-04 | up | 635 | 456 | 448 | 809 | 826 | 756 | SHMT2 | - | Serine hydroxymethyltransferase | 0 | SHMT2 |
| LOC101113111 | 64.29963026 | 31.32979207 | 1.037275 | 1.47E-04 | up | 35 | 27 | 31 | 63 | 75 | 57 | LOC101113111 | - | nucleosome assembly protein 1-like 1-like | 0 | NAP1L1 |
| WDFY1 | 326.886564 | 196.0072393 | 0.737883 | 1.47E-04 | up | 198 | 183 | 196 | 331 | 368 | 294 | WDFY1 | - | Uncharacterized protein | 0 | WDFY1 |
| TRNAG-CCC | 1.967763281 | 18.37332472 | -3.22298 | 1.47E-04 | down | 15 | 14 | 25 | 7 | 0 | 0 | TRNAG-CCC | + | transfer RNA glycine (anticodon CCC) | | |
| SLC43A2 | 1254.043651 | 167.6897327 | 2.902721 | 1.52E-04 | up | 166 | 188 | 135 | 859 | 1729 | 1146 | SLC43A2 | - | Uncharacterized protein | 0 | SLC43A2 |
| IVD | 1198.27012 | 2141.552322 | -0.8377 | 1.58E-04 | down | 2013 | 2120 | 2134 | 1485 | 995 | 1201 | IVD | + | Uncharacterized protein | 0 | IVD |
| HPCAL1 | 118.4782517 | 213.2118495 | -0.84767 | 1.64E-04 | down | 201 | 181 | 246 | 152 | 112 | 102 | HPCAL1 | + | Hippocalcin-like protein 1 | 1.00E-112 | HPCAL1 |
| SFXN5 | 9.533046178 | 32.16821995 | -1.75463 | 1.65E-04 | down | 45 | 26 | 26 | 20 | 5 | 6 | SFXN5 | - | Uncharacterized protein | 0 | SFXN5 |
| B3GALTL | 90.14854734 | 168.4049198 | -0.90156 | 1.65E-04 | down | 193 | 142 | 166 | 123 | 50 | 105 | B3GALTL | - | Uncharacterized protein | 0 | B3GALTL |
| UCK2 | 356.5408736 | 59.18910833 | 2.590664 | 1.70E-04 | up | 91 | 30 | 61 | 423 | 201 | 461 | UCK2 | + | Uncharacterized protein | 3.00E-128 | UCK2 |
| CAPN1 | 1330.873902 | 2283.208211 | -0.77869 | 1.70E-04 | down | 2547 | 2202 | 1997 | 1701 | 955 | 1435 | CAPN1 | + | Calpain 1 80 kDa subunit | 0 | CAPN1 |
| GLP2R | 3.885947595 | 27.73884274 | -2.83557 | 1.73E-04 | down | 24 | 19 | 39 | 10 | 1 | 2 | GLP2R | + | Glucagon-like peptide-2 receptor | 0 | GLP2R |
| TLCD2 | 2.823549292 | 23.84783415 | -3.07828 | 1.79E-04 | down | 22 | 14 | 35 | 5 | 2 | 2 | TLCD2 | - | Uncharacterized protein | 5.00E-110 | TLCD2 |
| NSUN3 | 41.68325332 | 91.57032066 | -1.13541 | 1.85E-04 | down | 105 | 88 | 78 | 64 | 20 | 46 | NSUN3 | + | Uncharacterized protein | 0 | NSUN3 |
| INTS9 | 217.6782535 | 120.0905162 | 0.858075 | 1.86E-04 | up | 149 | 123 | 84 | 225 | 240 | 197 | INTS9 | - | Uncharacterized protein | 0 | INTS9 |
| FAM20A | 91.07609314 | 34.18869195 | 1.413553 | 2.00E-04 | up | 31 | 33 | 36 | 76 | 104 | 93 | FAM20A | + | Uncharacterized protein | 0 | FAM20A |
| COG7 | 293.7273151 | 195.8114153 | 0.585013 | 2.04E-04 | up | 201 | 197 | 177 | 326 | 268 | 301 | COG7 | - | Uncharacterized protein | 0 | COG7 |
| WDR45 | 806.2717033 | 454.5574279 | 0.826804 | 2.08E-04 | up | 442 | 490 | 395 | 873 | 918 | 672 | WDR45 | + | Uncharacterized protein | 0 | WDR45 |
| MYOZ3 | 1231.479604 | 4040.972473 | -1.71431 | 2.10E-04 | down | 3413 | 4226 | 4102 | 1708 | 392 | 1686 | MYOZ3 | + | Uncharacterized protein | 3.00E-104 | MYOZ3 |
| NRAP | 75678.16265 | 32551.13433 | 1.217169 | 2.17E-04 | up | 27144 | 35006 | 32250 | 78424 | 65688 | 84984 | NRAP | - | Uncharacterized protein | 0 | NRAP |
| SUGT1 | 306.622893 | 183.975619 | 0.736951 | 2.18E-04 | up | 183 | 158 | 202 | 304 | 330 | 295 | SUGT1 | - | Uncharacterized protein | 2.00E-167 | SUGT1 |
| TNFSF4 | 5.953125402 | 30.1333467 | -2.33964 | 2.20E-04 | down | 38 | 34 | 17 | 1 | 8 | 8 | TNFSF4 | - | Uncharacterized protein | 8.00E-68 | TNFSF4 |
| LOC101102411 | 21.87106735 | 59.15762107 | -1.43554 | 2.22E-04 | down | 85 | 38 | 57 | 32 | 13 | 23 | LOC101102411 | - | neuroendocrine secretory protein 55-like | 4.00E-68 | GNAS |

| | | | | | | | | | | | | | | | | |
|---|---|---|---|---|---|---|---|---|---|---|---|---|---|---|---|---|
| AGAP2 | 53.52194958 | 24.29085783 | 1.139717 | 2.22E-04 | up | 17 | 26 | 27 | 52 | 59 | 51 | AGAP2 | + | Uncharacterized protein | 0 | AGAP2 |
| LOC101117028 | 203.1255151 | 492.3352676 | -1.27727 | 2.25E-04 | down | 526 | 523 | 396 | 216 | 125 | 272 | LOC101117028 | - | Uncharacterized protein | 4.00E-145 | ECHDC2 |
| USP2 | 793.4655614 | 1294.397348 | -0.70604 | 2.26E-04 | down | 1345 | 1297 | 1162 | 947 | 658 | 824 | USP2 | - | Ubiquitin carboxyl-terminal hydrolase | 0 | USP2 |
| KCNA7 | 185.0037609 | 325.6886771 | -0.81594 | 2.27E-04 | down | 337 | 341 | 277 | 230 | 178 | 162 | KCNA7 | - | Uncharacterized protein | 2.00E-159 | KCNA7 |
| ASS1 | 207.4477519 | 897.0938098 | -2.11251 | 2.27E-04 | down | 1203 | 965 | 498 | 187 | 204 | 232 | ASS1 | - | Uncharacterized protein | 0 | ASS1 |
| CCDC8 | 155.6518289 | 328.2522161 | -1.07648 | 2.28E-04 | down | 392 | 260 | 329 | 254 | 76 | 159 | CCDC8 | + | Uncharacterized protein | 0 | CCDC8 |
| FILIP1 | 726.633726 | 461.7741227 | 0.654041 | 2.32E-04 | up | 497 | 338 | 541 | 965 | 650 | 635 | FILIP1 | - | filamin A interacting protein 1 | 0 | FILIP1 |
| DTL | 33.76738241 | 8.248980123 | 2.033343 | 2.41E-04 | up | 17 | 4 | 5 | 39 | 43 | 22 | DTL | - | Uncharacterized protein | 0 | DTL |
| MYOG | 148.6274671 | 501.8397817 | -1.75553 | 2.41E-04 | down | 729 | 391 | 399 | 288 | 49 | 139 | MYOG | - | Myogenin | 1.00E-113 | MYOG |
| DRG1 | 637.9714643 | 402.3975391 | 0.66487 | 2.42E-04 | up | 416 | 307 | 472 | 670 | 677 | 594 | DRG1 | + | Uncharacterized protein | 0 | DRG1 |
| NUFIP1 | 167.0873361 | 105.4569576 | 0.663948 | 2.43E-04 | up | 130 | 74 | 113 | 194 | 183 | 136 | NUFIP1 | - | Uncharacterized protein | 0 | NUFIP1 |
| ABHD6 | 16.62915353 | 41.04650966 | -1.30354 | 2.54E-04 | down | 57 | 31 | 36 | 21 | 11 | 19 | ABHD6 | - | Uncharacterized protein | 0 | ABHD6 |
| ASXL1 | 804.0872779 | 443.6637531 | 0.857885 | 2.63E-04 | up | 465 | 425 | 417 | 796 | 931 | 713 | ASXL1 | + | Uncharacterized protein | 0 | ASXL1 |
| IBA57 | 117.7212815 | 195.3305457 | -0.73054 | 2.66E-04 | down | 202 | 195 | 177 | 135 | 96 | 128 | IBA57 | - | Uncharacterized protein | 5.00E-130 | IBA57 |
| APOBEC2 | 5159.001159 | 9361.803127 | -0.85969 | 2.67E-04 | down | 10086 | 8688 | 8881 | 5986 | 3451 | 6265 | APOBEC2 | + | Uncharacterized protein | 2.00E-110 | APOBEC2 |
| LRIG1 | 710.9990028 | 1361.640908 | -0.93743 | 2.68E-04 | down | 1736 | 1040 | 1314 | 1131 | 574 | 536 | LRIG1 | + | Uncharacterized protein | 0 | LRIG1 |
| TBC1D7 | 50.86315983 | 97.91275521 | -0.94488 | 2.79E-04 | down | 119 | 92 | 80 | 55 | 36 | 63 | TBC1D7 | + | Uncharacterized protein | 1.00E-160 | TBC1D7 |
| KCTD11 | 30.42674644 | 62.3531662 | -1.03512 | 2.90E-04 | down | 81 | 59 | 46 | 39 | 29 | 26 | KCTD11 | + | Uncharacterized protein | 3.00E-119 | KCTD11 |
| PPARGC1B | 111.0494892 | 311.8478816 | -1.48964 | 2.96E-04 | down | 411 | 273 | 250 | 212 | 38 | 105 | PPARGC1B | + | Uncharacterized protein | 0 | PPARGC1B |
| ARHGEF9 | 40.46634483 | 108.6228524 | -1.42453 | 3.11E-04 | down | 137 | 91 | 97 | 56 | 39 | 31 | ARHGEF9 | - | Uncharacterized protein | 0 | ARHGEF9 |
| DNAJC28 | 48.79850517 | 86.82260886 | -0.83123 | 3.15E-04 | down | 118 | 65 | 79 | 64 | 45 | 42 | DNAJC28 | + | DnaJ (Hsp40) homolog, subfamily C, member 28 | 0 | DNAJC28 |
| FAM210B | 116.8508556 | 194.5360163 | -0.73537 | 3.16E-04 | down | 210 | 186 | 178 | 169 | 88 | 107 | FAM210B | - | family with sequence similarity 210, member B | 2.00E-73 | FAM210B |
| OXNAD1 | 168.5967434 | 91.33036275 | 0.88441 | 3.16E-04 | up | 111 | 74 | 88 | 180 | 201 | 134 | OXNAD1 | + | Uncharacterized protein | 2.00E-152 | OXNAD1 |
| SYPL2 | 753.2600265 | 1731.996694 | -1.20122 | 3.29E-04 | down | 2017 | 1681 | 1430 | 1001 | 342 | 969 | SYPL2 | + | Uncharacterized protein | 5.00E-125 | SYPL2 |
| CXXC5 | 103.2413007 | 238.6065279 | -1.20861 | 3.30E-04 | down | 327 | 212 | 177 | 145 | 68 | 107 | CXXC5 | + | CXXC finger protein 5 | 3.00E-100 | CXXC5 |
| LDHD | 150.6412313 | 280.1180929 | -0.89492 | 3.31E-04 | down | 262 | 289 | 267 | 201 | 140 | 126 | LDHD | - | Uncharacterized protein | 0 | LDHD |
| TMEM2 | 146.0440152 | 377.885611 | -1.37155 | 3.39E-04 | down | 348 | 282 | 487 | 167 | 131 | 148 | TMEM2 | + | Uncharacterized protein | 0 | TMEM2 |
| GDF11 | 5.8620073 | 25.12173772 | -2.09947 | 3.47E-04 | down | 39 | 15 | 23 | 8 | 2 | 8 | GDF11 | - | Uncharacterized protein | 0 | GDF11 |
| ATP13A1 | 388.7312941 | 220.7561814 | 0.816319 | 3.49E-04 | up | 240 | 261 | 144 | 462 | 394 | 338 | ATP13A1 | + | Uncharacterized protein | 0 | ATP13A1 |
| ABCD1 | 163.4665462 | 379.4074562 | -1.21475 | 3.50E-04 | down | 359 | 403 | 345 | 143 | 130 | 215 | ABCD1 | - | Uncharacterized protein | 0 | ABCD1 |
| LARP1 | 1097.297649 | 1775.84809 | -0.69455 | 3.52E-04 | down | 1694 | 1715 | 1797 | 1212 | 1192 | 950 | LARP1 | + | Uncharacterized protein | 0 | LARP1 |
| TP53INP1 | 77.99364618 | 40.59981207 | 0.941884 | 3.82E-04 | up | 28 | 43 | 46 | 93 | 85 | 62 | TP53INP1 | + | Uncharacterized protein | 1.00E-119 | TP53INP1 |

| | | | | | | | | | | | | | | | | |
|---|---|---|---|---|---|---|---|---|---|---|---|---|---|---|---|---|
| AR | 45.00919237 | 86.08420073 | -0.93553 | 3.85E-04 | down | 105 | 65 | 88 | 62 | 26 | 51 | AR | + | Androgen receptor | 0 | AR |
| C18H14orf2 | 885.8895667 | 1430.162069 | -0.69098 | 3.91E-04 | down | 1464 | 1432 | 1304 | 912 | 843 | 930 | C18H14orf2 | - | Uncharacterized protein | 6.00E-22 | C14orf2 |
| SERT | 52.95726538 | 17.06946468 | 1.633411 | 3.99E-04 | up | 25 | 11 | 16 | 84 | 45 | 38 | SERT | - | Transporter | 0 | SLC6A4 |
| LOC101118057 | 794.3283894 | 374.1819364 | 1.085996 | 4.03E-04 | up | 439 | 358 | 312 | 719 | 907 | 768 | LOC101118057 | + | Ribosomal protein | 1.00E-125 | RPL10A |
| NRF1 | 183.1791608 | 117.6321546 | 0.638973 | 4.28E-04 | up | 117 | 110 | 119 | 186 | 197 | 173 | NRF1 | + | Nuclear respiratory factor 1 | 0 | NRF1 |
| KIAA0226L | 12.93764443 | 41.41391209 | -1.67854 | 4.31E-04 | down | 49 | 23 | 53 | 16 | 15 | 9 | KIAA0226L | - | KIAA0226-like ortholog | 0 | KIAA0226L |
| LOC101102587 | 9.235315782 | 29.33897037 | -1.66759 | 4.43E-04 | down | 38 | 24 | 26 | 20 | 2 | 8 | LOC101102587 | + | ecto-ADP-ribosyltransferase 5-like | 6.00E-87 | ART5 |
| PMF1 | 213.8764533 | 134.7594244 | 0.666391 | 4.59E-04 | up | 146 | 119 | 134 | 211 | 218 | 218 | PMF1 | + | Uncharacterized protein | 2.00E-71 | PMF1 |
| KTN1 | 575.8367015 | 325.7624378 | 0.821839 | 4.69E-04 | up | 359 | 215 | 401 | 704 | 628 | 444 | KTN1 | + | Uncharacterized protein | 0 | KTN1 |
| TRAF3IP2 | 146.088334 | 70.4826266 | 1.051501 | 4.89E-04 | up | 77 | 53 | 80 | 132 | 163 | 145 | TRAF3IP2 | + | Uncharacterized protein | 0 | TRAF3IP2 |
| NPNT | 36.29650847 | 190.2292086 | -2.38984 | 4.99E-04 | down | 294 | 90 | 202 | 69 | 13 | 34 | NPNT | - | Uncharacterized protein | 0 | NPNT |
| FAM189B | 194.3989896 | 357.0121811 | -0.87695 | 5.22E-04 | down | 379 | 330 | 345 | 200 | 134 | 252 | FAM189B | - | Uncharacterized protein | 0 | FAM189B |
| CASQ1 | 17353.2746 | 45168.036 | -1.38009 | 5.41E-04 | down | 42680 | 45481 | 43948 | 16008 | 10373 | 25403 | CASQ1 | + | Calsequestrin | 0 | CASQ1 |
| CBY1 | 296.9379738 | 497.8886011 | -0.74566 | 5.48E-04 | down | 499 | 443 | 526 | 349 | 218 | 339 | CBY1 | + | Uncharacterized protein | 6.00E-57 | CBY1 |
| AK4 | 144.6412354 | 529.2371395 | -1.87144 | 5.60E-04 | down | 693 | 565 | 313 | 218 | 83 | 150 | AK4 | + | Uncharacterized protein | 9.00E-120 | AK4 |
| TM4SF5 | 2.172422637 | 14.20872784 | -2.7094 | 5.68E-04 | down | 12 | 17 | 12 | 0 | 1 | 5 | TM4SF5 | - | Uncharacterized protein | 1.00E-77 | TM4SF5 |
| EMILIN3 | 14.69242343 | 75.14096355 | -2.35453 | 6.00E-04 | down | 67 | 47 | 109 | 22 | 12 | 12 | EMILIN3 | - | elastin microfibril interfacer 3 | 0 | EMILIN3 |
| OSBPL11 | 344.4226363 | 160.2165827 | 1.104156 | 6.03E-04 | up | 136 | 157 | 174 | 484 | 362 | 230 | OSBPL11 | - | Oxysterol-binding protein | 0 | OSBPL11 |
| FOXO6 | 9.287104278 | 65.40202589 | -2.81603 | 6.04E-04 | down | 111 | 30 | 62 | 14 | 6 | 9 | FOXO6 | + | Uncharacterized protein | 3.00E-107 | FOXO6 |
| PSTPIP1 | 47.63252987 | 86.83785793 | -0.86638 | 6.13E-04 | down | 86 | 84 | 85 | 71 | 38 | 40 | PSTPIP1 | + | Uncharacterized protein | 0 | PSTPIP1 |
| DYNLL1 | 283.2590825 | 468.9995077 | -0.72746 | 6.17E-04 | down | 602 | 406 | 395 | 399 | 231 | 251 | DYNLL1 | + | Uncharacterized protein | 5.00E-49 | DYNLL1 |
| ARMC2 | 186.4942803 | 3.70368645 | 5.654026 | 6.34E-04 | up | 4 | 3 | 4 | 16 | 476 | 51 | ARMC2 | - | armadillo repeat containing 2 | 0 | ARMC2 |
| TPX2 | 79.20392609 | 161.4963857 | -1.02786 | 6.48E-04 | down | 175 | 159 | 142 | 131 | 51 | 68 | TPX2 | + | Uncharacterized protein | 0 | TPX2 |
| AGPAT9 | 120.9915832 | 226.7338866 | -0.90609 | 6.76E-04 | down | 244 | 216 | 209 | 145 | 108 | 118 | AGPAT9 | + | Uncharacterized protein | 0 | AGPAT9 |
| PCYT1A | 123.5074378 | 211.1827933 | -0.77389 | 6.89E-04 | down | 229 | 151 | 250 | 138 | 120 | 119 | PCYT1A | - | Uncharacterized protein | 0 | PCYT1A |
| LOC101112485 | 21.30884927 | 71.00387388 | -1.73645 | 7.39E-04 | down | 52 | 68 | 86 | 30 | 13 | 23 | LOC101112485 | - | cytochrome b-c1 complex subunit 7-like | | |
| DHRS7B | 112.4449846 | 281.1290178 | -1.32201 | 7.56E-04 | down | 332 | 288 | 211 | 154 | 48 | 144 | DHRS7B | + | Uncharacterized protein | 6.00E-159 | DHRS7B |
| DDX54 | 1150.593827 | 749.2953494 | 0.618772 | 7.60E-04 | up | 806 | 720 | 684 | 1161 | 1162 | 1163 | DDX54 | - | Uncharacterized protein | 0 | DDX54 |
| ERGIC3 | 674.5711201 | 447.8588717 | 0.590926 | 7.62E-04 | up | 594 | 361 | 391 | 783 | 593 | 686 | ERGIC3 | + | Uncharacterized protein | 0 | ERGIC3 |
| BNIP3 | 5520.951614 | 2061.477173 | 1.421238 | 7.94E-04 | up | 2360 | 2082 | 1645 | 4622 | 6200 | 5724 | BNIP3 | - | Uncharacterized protein | 2.00E-79 | BNIP3 |
| LOC101118607 | 133.0033924 | 76.29305121 | 0.801839 | 8.09E-04 | up | 70 | 53 | 103 | 166 | 129 | 115 | LOC101118607 | + | ribosomal protein S3A pseudogene | | |
| SF3A2 | 197.5904467 | 118.9827863 | 0.73176 | 8.11E-04 | up | 140 | 104 | 110 | 190 | 208 | 199 | SF3A2 | + | Uncharacterized protein | 2.00E-120 | SF3A2 |

| | | | | | | | | | | | | | | | | |
|---|---|---|---|---|---|---|---|---|---|---|---|---|---|---|---|---|
| LOC101104501 | 82.27234815 | 27.92903806 | 1.558642 | 8.40E-04 | up | 37 | 22 | 25 | 124 | 87 | 48 | LOC101104501 | + | Uncharacterized protein | 6.00E-137 | HNRNPA1 |
| AIF1 | 49.71796836 | 87.14396971 | -0.80963 | 8.43E-04 | down | 91 | 81 | 85 | 65 | 33 | 55 | AIF1 | - | Uncharacterized protein | 3.00E-70 | AIF1 |
| LOC101106204 | 676.0765502 | 1339.885328 | -0.98685 | 8.48E-04 | down | 1356 | 999 | 1623 | 787 | 544 | 733 | LOC101106204 | + | coiled-coil domain containing 47 pseudogene | | |
| SLC25A33 | 910.6108402 | 80.44052674 | 3.50084 | 8.63E-04 | up | 142 | 63 | 42 | 560 | 1358 | 785 | SLC25A33 | - | SLC25A33 | 0 | SLC25A33 |
| ELOVL6 | 8.689719058 | 370.9648541 | -5.41583 | 8.81E-04 | down | 1160 | 23 | 63 | 17 | 5 | 6 | ELOVL6 | + | Elongation of very long chain fatty acids protein | 1.00E-147 | ELOVL6 |
| C1QTNF7 | 36.87175564 | 14.59156775 | 1.337381 | 8.94E-04 | up | 23 | 7 | 15 | 52 | 35 | 28 | C1QTNF7 | + | Uncharacterized protein | 3.00E-163 | C1QTNF7 |
| ACTN2 | 62892.35221 | 38891.04253 | 0.693447 | 9.28E-04 | up | 41857 | 35246 | 37888 | 78324 | 50397 | 64455 | ACTN2 | + | Uncharacterized protein | 0 | ACTN2 |
| GALNTL2 | 65.11336396 | 8.378650186 | 2.958164 | 9.43E-04 | up | 3 | 7 | 14 | 120 | 52 | 37 | GALNTL2 | + | Polypeptide N-acetylgalactosaminyltransferase | 0 | GALNT15 |
| ABAT | 22.09843496 | 100.0567623 | -2.1788 | 9.54E-04 | down | 90 | 63 | 144 | 25 | 13 | 29 | ABAT | + | Uncharacterized protein | 0 | ABAT |
| GANC | 161.1101055 | 279.5570803 | -0.7951 | 9.63E-04 | down | 279 | 257 | 287 | 245 | 127 | 133 | GANC | + | Uncharacterized protein | 0 | GANC |
| CUTC | 606.5435876 | 389.1830387 | 0.640162 | 9.79E-04 | up | 347 | 319 | 479 | 697 | 623 | 538 | CUTC | + | Uncharacterized protein | 1.00E-151 | CUTC |
| CDC26 | 44.18053366 | 77.66785768 | -0.81391 | 9.84E-04 | down | 90 | 67 | 74 | 44 | 33 | 56 | CDC26 | + | Uncharacterized protein | 1.00E-40 | CDC26 |
| KHK | 56.42355937 | 101.2293862 | -0.84326 | 9.94E-04 | down | 115 | 93 | 92 | 52 | 44 | 73 | KHK | + | Uncharacterized protein | 3.00E-158 | KHK |
| LOC101107996 | 79.23165211 | 33.18186491 | 1.255682 | 9.98E-04 | up | 25 | 27 | 45 | 114 | 81 | 53 | LOC101107996 | - | Ig lambda chain V-I region BL2-like | | |
| DYNC1LI1 | 374.7389491 | 230.6639186 | 0.700094 | 0.00101065 | up | 258 | 144 | 290 | 429 | 360 | 357 | DYNC1LI1 | - | Uncharacterized protein | 0 | DYNC1LI1 |
| IGSF1 | 5.839299049 | 26.29910704 | -2.17115 | 0.00101574 | down | 42 | 14 | 25 | 8 | 3 | 7 | IGSF1 | + | Uncharacterized protein | 0 | IGSF1 |
| RPL3 | 6175.503665 | 3648.284374 | 0.759339 | 0.00101637 | up | 4990 | 2646 | 3389 | 8302 | 5257 | 5570 | RPL3 | - | Uncharacterized protein | 0 | RPL3 |
| SCD | 83.91646541 | 2310.114784 | -4.78287 | 0.00105746 | down | 5803 | 620 | 1072 | 77 | 45 | 128 | SCD | + | Stearoyl-CoA desaturase | 0 | SCD |
| CDH1 | 10.54586551 | 46.89853655 | -2.15287 | 0.00111649 | down | 75 | 36 | 32 | 21 | 5 | 8 | CDH1 | + | cadherin 1, type 1, E-cadherin (epithelial) | | |
| LOC101123261 | 35.48059376 | 13.36076193 | 1.409028 | 0.00112085 | up | 5 | 21 | 11 | 39 | 32 | 37 | LOC101123261 | + | ufm1-specific protease 1-like | 4.00E-58 | UFSP1 |
| SLC2A4 | 975.3060901 | 2020.969338 | -1.05112 | 0.00114159 | down | 1967 | 2051 | 1899 | 1556 | 737 | 779 | SLC2A4 | + | Uncharacterized protein | 0 | SLC2A4 |
| TNNC1 | 20984.0368 | 46777.41999 | -1.15652 | 0.00114566 | down | 48851 | 33644 | 56760 | 17941 | 23104 | 21901 | TNNC1 | + | Uncharacterized protein | 8.00E-87 | TNNC1 |
| C7H15orf52 | 72.86441831 | 138.9927068 | -0.93172 | 0.00115707 | down | 132 | 122 | 155 | 103 | 48 | 75 | C7H15orf52 | - | Uncharacterized protein | 5.00E-179 | C15orf52 |
| CBLB | 151.1247617 | 84.71483447 | 0.835054 | 0.00118667 | up | 131 | 55 | 73 | 188 | 135 | 142 | CBLB | - | Uncharacterized protein | 0 | CBLB |
| NFASC | 9.142529842 | 32.56163411 | -1.83251 | 0.00120475 | down | 51 | 26 | 22 | 6 | 10 | 11 | NFASC | + | Uncharacterized protein | 0 | NFASC |
| CAMK2 | 1177.641464 | 2817.942056 | -1.25874 | 0.00124391 | down | 2991 | 2879 | 2413 | 1711 | 480 | 1454 | CAMK2 | - | Ca2+/calmodulin-dependent protein kinase II | 0 | CAMK2A |
| GET4 | 959.7120508 | 1700.456642 | -0.82525 | 0.00125231 | down | 2018 | 1446 | 1603 | 847 | 783 | 1238 | GET4 | - | Uncharacterized protein | 1.00E-108 | GET4 |
| C1QTNF6 | 39.98632121 | 221.7138347 | -2.47112 | 0.00126631 | down | 269 | 94 | 311 | 36 | 22 | 61 | C1QTNF6 | - | Uncharacterized protein | 6.00E-105 | C1QTNF6 |
| BUB3 | 165.6427464 | 105.5623822 | 0.649979 | 0.00127142 | up | 114 | 97 | 101 | 183 | 191 | 133 | BUB3 | + | Uncharacterized protein | 0 | BUB3 |
| ORAOV1 | 90.31114013 | 42.03789923 | 1.103213 | 0.00127968 | up | 44 | 39 | 41 | 78 | 98 | 95 | ORAOV1 | - | oral cancer overexpressed 1 | 4.00E-71 | ORAOV1 |
| SCN4A | 2682.021695 | 4051.300213 | -0.59506 | 0.00130005 | down | 4716 | 3876 | 3410 | 3417 | 2234 | 2610 | SCN4A | - | Sodium channel protein | 0 | SCN4A |

| | | | | | | | | | | | | | | | | |
|---|---|---|---|---|---|---|---|---|---|---|---|---|---|---|---|---|
| QTRT1 | 105.3630467 | 61.79254218 | 0.769864 | 0.00134945 | up | 69 | 64 | 49 | 106 | 104 | 109 | QTRT1 | + | Queuine tRNA-ribosyltransferase | 0 | QTRT1 |
| KPNA2 | 126.8611314 | 203.065591 | -0.6787 | 0.00140109 | down | 253 | 156 | 200 | 136 | 157 | 95 | KPNA2 | - | Importin subunit alpha | 0 | KPNA2 |
| EIF2C2 | 265.4286867 | 133.0511167 | 0.996344 | 0.00140856 | up | 179 | 109 | 112 | 242 | 281 | 276 | EIF2C2 | + | Uncharacterized protein | 0 | AGO2 |
| SGCG | 431.4356062 | 1052.93683 | -1.2872 | 0.0014202 | down | 1087 | 869 | 1162 | 661 | 123 | 556 | SGCG | - | Uncharacterized protein | 7.00E-134 | SGCG |
| SLAMF8 | 4.049352916 | 26.21736261 | -2.69476 | 0.00142187 | down | 20 | 34 | 21 | 7 | 5 | 1 | SLAMF8 | + | Uncharacterized protein | 2.00E-118 | SLAMF8 |
| DBI | 254.7471847 | 448.8771444 | -0.81725 | 0.00143936 | down | 619 | 312 | 428 | 321 | 231 | 233 | DBI | + | Uncharacterized protein | 3.00E-42 | DBI |
| LOC101112367 | 22.16879775 | 52.1251986 | -1.23345 | 0.00146459 | down | 57 | 34 | 65 | 32 | 16 | 21 | LOC101112367 | - | dystrobrevin alpha-like | 2.00E-22 | DTNA |
| LOC101109877 | 28.54789173 | 79.80941552 | -1.48317 | 0.00149129 | down | 76 | 54 | 107 | 35 | 30 | 23 | LOC101109877 | - | uncharacterized LOC101109877 | | |
| CDC42EP3 | 103.3795031 | 370.2834387 | -1.84068 | 0.00149278 | down | 585 | 224 | 327 | 159 | 58 | 106 | CDC42EP3 | + | Uncharacterized protein | 5.00E-124 | CDC42EP3 |
| PDCD2 | 63.32200658 | 30.34610357 | 1.061196 | 0.00150899 | up | 28 | 28 | 33 | 74 | 77 | 44 | PDCD2 | - | Uncharacterized protein | 1.00E-137 | PDCD2 |
| LIN54 | 149.6212275 | 75.89630462 | 0.979213 | 0.00153177 | up | 73 | 55 | 97 | 164 | 178 | 116 | LIN54 | - | lin-54 homolog (C. elegans) | 0 | LIN54 |
| ATG13 | 304.8105807 | 190.4646566 | 0.67839 | 0.00156449 | up | 176 | 173 | 210 | 330 | 345 | 256 | ATG13 | + | ATG13 autophagy related 13-related protein | 0 | ATG13 |
| SCPEP1 | 1340.463141 | 257.3567107 | 2.38089 | 0.00157103 | up | 344 | 176 | 258 | 1421 | 1916 | 775 | SCPEP1 | + | Uncharacterized protein | 0 | SCPEP1 |
| LOC101115351 | 19.36988114 | 50.10407895 | -1.37111 | 0.00158914 | down | 44 | 51 | 51 | 24 | 8 | 27 | LOC101115351 | + | Uncharacterized protein | 0 | SLC4A3 |
| PTPN23 | 276.0397404 | 154.3996463 | 0.838207 | 0.00160734 | up | 153 | 160 | 139 | 282 | 325 | 233 | PTPN23 | - | Uncharacterized protein | 0 | PTPN23 |
| TMEM59L | 11.76526863 | 36.68551186 | -1.64068 | 0.00164994 | down | 35 | 32 | 41 | 12 | 1 | 22 | TMEM59L | - | transmembrane protein 59-like | 3.00E-139 | TMEM59L |
| C1QTNF2 | 9.20235817 | 36.91340005 | -2.00407 | 0.00165939 | down | 60 | 26 | 27 | 15 | 6 | 8 | C1QTNF2 | - | Uncharacterized protein | 5.00E-138 | C1QTNF2 |
| CDH15 | 263.6988255 | 122.5036723 | 1.106066 | 0.00166226 | up | 127 | 115 | 119 | 226 | 288 | 277 | CDH15 | + | Uncharacterized protein | 0 | CDH15 |
| PES1 | 853.1920522 | 322.3392165 | 1.404291 | 0.00166973 | up | 353 | 337 | 258 | 730 | 1061 | 776 | PES1 | - | Pescadillo homolog | 0 | PES1 |
| MGARP | 19.33278957 | 45.91316418 | -1.24786 | 0.00167401 | down | 43 | 48 | 43 | 32 | 12 | 17 | MGARP | + | Uncharacterized protein | 6.00E-82 | MGARP |
| TMEM141 | 116.9937907 | 61.27160106 | 0.933142 | 0.00172307 | up | 78 | 47 | 59 | 173 | 99 | 94 | TMEM141 | - | transmembrane protein 141 | 1.00E-32 | TMEM141 |
| TRIM13 | 67.56686713 | 203.9884834 | -1.5941 | 0.00173098 | down | 195 | 137 | 274 | 104 | 52 | 56 | TRIM13 | + | Uncharacterized protein | 0 | TRIM13 |
| PFKFB3 | 8379.261621 | 949.7301171 | 3.141234 | 0.00174486 | up | 1591 | 329 | 1039 | 4857 | 10387 | 9429 | PFKFB3 | + | Uncharacterized protein | 0 | PFKFB3 |
| C20H6orf136 | 170.4278723 | 314.2358384 | -0.88269 | 0.00175125 | down | 376 | 311 | 244 | 223 | 121 | 181 | C20H6orf136 | - | Uncharacterized protein | 9.00E-149 | C6orf136 |
| ZNF367 | 43.03729513 | 103.389867 | -1.26444 | 0.00175285 | down | 103 | 75 | 129 | 68 | 26 | 41 | ZNF367 | + | Uncharacterized protein | 8.00E-131 | ZNF367 |
| HYAL2 | 218.549492 | 126.6512656 | 0.787099 | 0.0017557 | up | 132 | 144 | 94 | 233 | 204 | 227 | HYAL2 | + | Hyaluronidase-2 | 0 | HYAL2 |
| LOC101109746 | 100.8312896 | 202.8110081 | -1.00819 | 0.00176819 | down | 241 | 198 | 162 | 164 | 71 | 83 | LOC101109746 | - | Uncharacterized protein | 2.00E-104 | HLA-DMB |
| RAB21 | 117.9725548 | 66.18563956 | 0.833861 | 0.00179145 | up | 65 | 51 | 80 | 134 | 137 | 91 | RAB21 | - | RAB21 | 4.00E-121 | RAB21 |
| TMEM63B | 333.0697227 | 576.8349194 | -0.79233 | 0.00179274 | down | 759 | 501 | 468 | 410 | 252 | 359 | TMEM63B | + | Uncharacterized protein | 0 | TMEM63B |
| PAK1 | 286.9866995 | 482.127318 | -0.74843 | 0.00180025 | down | 525 | 387 | 521 | 252 | 261 | 346 | PAK1 | + | Non-specific serine/threonine protein kinase | 0 | PAK1 |
| CCND1 | 10.88901241 | 39.23665141 | -1.84933 | 0.00182753 | down | 60 | 32 | 27 | 21 | 6 | 8 | CCND1 | + | Cyclin D1 | 5.00E-66 | CCND1 |
| LAMTOR2 | 492.2609454 | 744.1928348 | -0.59625 | 0.00183979 | down | 777 | 682 | 737 | 501 | 426 | 561 | LAMTOR2 | + | Uncharacterized protein | 6.00E-69 | LAMTOR2 |

| | | | | | | | | | | | | | | | | |
|---|---|---|---|---|---|---|---|---|---|---|---|---|---|---|---|---|
| MAFF | 239.2610273 | 73.10877508 | 1.710469 | 0.0018853 | up | 137 | 77 | 9 | 220 | 291 | 212 | MAFF | + | Uncharacterized protein | 8.00E-63 | MAFF |
| ECE1 | 2587.361447 | 1410.703799 | 0.875067 | 0.00188905 | up | 1640 | 1349 | 1190 | 2656 | 3072 | 2150 | ECE1 | + | Endothelin converting enzyme | 0 | ECE1 |
| PTPN1 | 200.8366147 | 130.476344 | 0.622234 | 0.00190046 | up | 120 | 125 | 137 | 235 | 171 | 208 | PTPN1 | + | Uncharacterized protein | 0 | PTPN1 |
| ARHGAP6 | 159.2160264 | 102.9094213 | 0.62961 | 0.00190163 | up | 134 | 90 | 84 | 190 | 174 | 126 | ARHGAP6 | - | Uncharacterized protein | 0 | ARHGAP6 |
| C12H1orf21 | 1446.553793 | 900.7149982 | 0.683477 | 0.00192184 | up | 755 | 815 | 1063 | 1557 | 1441 | 1406 | C12H1orf21 | + | chromosome 12 open reading frame, human C1orf21 | 3.00E-67 | C1orf21 |
| HSD17B12 | 66.9054626 | 209.7965294 | -1.64879 | 0.00193271 | down | 321 | 113 | 210 | 77 | 53 | 74 | HSD17B12 | + | Uncharacterized protein | 4.00E-157 | HSD17B12 |
| THSD4 | 17.31131336 | 89.39424929 | -2.36847 | 0.00198319 | down | 106 | 109 | 47 | 34 | 13 | 9 | THSD4 | + | Uncharacterized protein | 0 | THSD4 |
| CACNG6 | 142.7535284 | 217.9238232 | -0.6103 | 0.00199372 | down | 239 | 220 | 183 | 157 | 136 | 142 | CACNG6 | + | Voltage-dependent calcium channel gamma subunit | 7.00E-99 | CACNG6 |
| FAM178A | 393.1876279 | 199.1582618 | 0.981303 | 0.00204314 | up | 226 | 139 | 231 | 477 | 464 | 273 | FAM178A | + | Uncharacterized protein | 0 | FAM178A |
| LOC101115438 | 256.3030918 | 140.5249496 | 0.867025 | 0.00205935 | up | 182 | 96 | 146 | 245 | 285 | 245 | LOC101115438 | - | RNA-binding motif protein, X chromosome-like | 4.00E-48 | RBMX |
| LOC780457 | 1111.584303 | 2138.15702 | -0.94375 | 0.00206782 | down | 2043 | 1684 | 2592 | 1329 | 964 | 1113 | LOC780457 | - | | | |
| GRAMD1B | 72.20912919 | 26.22356822 | 1.461317 | 0.00207309 | up | 26 | 33 | 17 | 94 | 46 | 82 | GRAMD1B | + | GRAM domain containing 1B | 0 | GRAMD1B |
| THY1 | 412.6398603 | 844.1201743 | -1.03257 | 0.0020948 | down | 1068 | 602 | 870 | 665 | 243 | 389 | THY1 | - | Uncharacterized protein | 5.00E-57 | THY1 |
| ST3GAL6 | 329.3967309 | 173.3094203 | 0.926476 | 0.0021089 | up | 183 | 149 | 181 | 384 | 398 | 232 | ST3GAL6 | + | Uncharacterized protein | 5.00E-172 | ST3GAL6 |
| C26H8orf4 | 1629.153758 | 624.5091095 | 1.383328 | 0.00213222 | up | 1091 | 610 | 199 | 1795 | 1405 | 1756 | C26H8orf4 | + | chromosome 26 open reading frame, human C8orf4 | 1.00E-51 | C8orf4 |
| LOC101109590 | 88.69209746 | 49.76425436 | 0.833696 | 0.00217425 | up | 45 | 37 | 65 | 120 | 77 | 78 | LOC101109590 | - | ribosomal protein S3A pseudogene | | |
| SP1 | 962.3952451 | 626.5191603 | 0.619271 | 0.00219926 | up | 735 | 606 | 515 | 1059 | 1082 | 802 | SP1 | - | Sp1 transcription factor | | |
| IRS1 | 413.8593775 | 887.8892018 | -1.10124 | 0.00224192 | down | 886 | 838 | 887 | 724 | 243 | 347 | IRS1 | - | Uncharacterized protein | 0 | IRS1 |
| LOC101118433 | 1931.381984 | 4075.965162 | -1.07751 | 0.00224502 | down | 3993 | 3020 | 5072 | 2216 | 2088 | 1618 | LOC101118433 | + | tropomyosin alpha-3 chain-like | 1.00E-130 | TPM3 |
| NDUFS1 | 1905.335649 | 3552.03239 | -0.8986 | 0.00225116 | down | 3263 | 3092 | 4085 | 2587 | 1786 | 1545 | NDUFS1 | - | Uncharacterized protein | 0 | NDUFS1 |
| PID1 | 641.3657562 | 265.4927888 | 1.272475 | 0.00226214 | up | 281 | 107 | 414 | 627 | 688 | 626 | PID1 | - | Uncharacterized protein | 3.00E-126 | PID1 |
| LOC780509 | 27537.55444 | 77386.23824 | -1.49068 | 0.00234337 | down | 70028 | 80769 | 74730 | 20557 | 19111 | 41549 | LOC780509 | - | Myoglobin | 5.00E-72 | MB |
| LOC101102833 | 88.51489327 | 333.2993866 | -1.91283 | 0.00236757 | down | 384 | 363 | 234 | 214 | 24 | 55 | LOC101102833 | - | uncharacterized LOC101102833 | 9.00E-66 | MAFB |
| FZD2 | 69.04857691 | 159.6101762 | -1.20887 | 0.00243947 | down | 161 | 125 | 187 | 124 | 41 | 55 | FZD2 | + | Frizzled 2 | | |
| RELT | 8.522436371 | 32.54271114 | -1.933 | 0.00247016 | down | 49 | 31 | 18 | 9 | 10 | 7 | RELT | + | Uncharacterized protein | 6.00E-166 | RELT |
| FXC1 | 70.04339215 | 37.54270963 | 0.899716 | 0.0025263 | up | 55 | 27 | 32 | 98 | 62 | 58 | FXC1 | - | Uncharacterized protein | 1.00E-45 | TIMM10B |
| DNAJC1 | 155.6884928 | 64.99237353 | 1.26032 | 0.00253834 | up | 67 | 57 | 68 | 168 | 202 | 107 | DNAJC1 | - | Uncharacterized protein | 0 | DNAJC1 |
| TBC1D4 | 387.5120193 | 725.8558488 | -0.90544 | 0.00254656 | down | 697 | 569 | 880 | 459 | 330 | 397 | TBC1D4 | - | Uncharacterized protein | 0 | TBC1D4 |
| YBX2 | 20.41594319 | 5.407166258 | 1.916752 | 0.00261447 | up | 6 | 5 | 5 | 23 | 14 | 25 | YBX2 | - | Uncharacterized protein | 2.00E-113 | YBX2 |
| FAM168B | 279.2631146 | 180.1272489 | 0.632609 | 0.00262258 | up | 204 | 171 | 158 | 276 | 286 | 283 | FAM168B | - | Uncharacterized protein | 6.00E-101 | FAM168B |
| METTL3 | 379.3370063 | 170.0169641 | 1.157801 | 0.0026464 | up | 184 | 168 | 149 | 345 | 471 | 330 | METTL3 | + | Uncharacterized protein | 0 | METTL3 |

| | | | | | | | | | | | | | | | | |
|---|---|---|---|---|---|---|---|---|---|---|---|---|---|---|---|---|
| CSGALNACT1 | 169.9707708 | 76.10159593 | 1.159288 | 0.00268193 | up | 62 | 78 | 81 | 242 | 183 | 107 | CSGALNACT1 | + | Uncharacterized protein | 0 | CSGALNACT 1 |
| PIEZO1 | 216.5560982 | 370.8346895 | -0.77604 | 0.00277776 | down | 461 | 300 | 349 | 342 | 175 | 165 | PIEZO1 | - | Uncharacterized protein | 0 | PIEZO1 |
| SMS | 42.23358272 | 19.16149544 | 1.14018 | 0.00281733 | up | 9 | 25 | 20 | 57 | 38 | 36 | SMS | + | spermine synthase | 0 | SMS |
| SNRNP35 | 51.57824877 | 94.60581534 | -0.87517 | 0.00291784 | down | 112 | 87 | 82 | 56 | 33 | 67 | SNRNP35 | - | Uncharacterized protein | 1.00E-112 | SNRNP35 |
| MAP7D1 | 2194.334645 | 4255.475006 | -0.95554 | 0.00293745 | down | 4875 | 3646 | 4131 | 2319 | 1289 | 3007 | MAP7D1 | + | Uncharacterized protein | 0 | MAP7D1 |
| NUDT19 | 78.30932365 | 43.72881474 | 0.8406 | 0.00296908 | up | 42 | 44 | 42 | 86 | 65 | 87 | NUDT19 | + | Uncharacterized protein | 3.00E-146 | NUDT19 |
| VWA2 | 2.058881384 | 12.75673401 | -2.63133 | 0.00302507 | down | 10 | 13 | 14 | 0 | 6 | 0 | VWA2 | + | Uncharacterized protein | 0 | np_940898 |
| SPARC | 6012.829769 | 13052.238 | -1.11818 | 0.00306237 | down | 17928 | 8107 | 13599 | 6530 | 5452 | 6304 | SPARC | - | Secreted, acidic, cysteine-rich protein | 1.00E-177 | SPARC |
| LOC101114717 | 13.46248585 | 48.12129981 | -1.83773 | 0.00313106 | down | 70 | 35 | 41 | 33 | 9 | 3 | LOC101114717 | - | pleckstrin homology domain-containing family A member 6-like | 0 | PLEKHA6 |
| LOC101110545 | 11.33961367 | 31.42042687 | -1.47033 | 0.00321916 | down | 27 | 28 | 37 | 20 | 6 | 10 | LOC101110545 | - | Uncharacterized protein | 6.00E-110 | HLA-DOA |
| TTL | 113.0510083 | 246.8392345 | -1.1266 | 0.00322563 | down | 247 | 263 | 212 | 169 | 62 | 121 | TTL | - | Uncharacterized protein | 0 | TTL |
| IMPDH2 | 1949.012083 | 1275.794157 | 0.611347 | 0.00335029 | up | 1391 | 1124 | 1264 | 1972 | 2190 | 1758 | IMPDH2 | + | Inosine-5'-monophosphate dehydrogenase | 0 | IMPDH2 |
| MT1A | 51.14509567 | 2.735588508 | 4.224673 | 0.00337185 | up | 8 | 1 | 0 | 18 | 65 | 65 | MT1A | - | Metallothionein-1A | 5.00E-13 | MT1E |
| IPP | 72.28201511 | 184.3476963 | -1.35072 | 0.00343756 | down | 148 | 159 | 232 | 101 | 65 | 59 | IPP | - | Uncharacterized protein | 0 | IPP |
| TRIM68 | 130.0900793 | 210.8946507 | -0.69701 | 0.00357446 | down | 215 | 175 | 234 | 179 | 96 | 128 | TRIM68 | + | Uncharacterized protein | 0 | TRIM68 |
| MMAB | 154.505996 | 269.4487855 | -0.80235 | 0.00366352 | down | 268 | 241 | 285 | 147 | 123 | 194 | MMAB | + | Uncharacterized protein | 1.00E-113 | MMAB |
| PTPN14 | 413.8985788 | 642.9691596 | -0.63547 | 0.00368892 | down | 746 | 611 | 548 | 558 | 313 | 409 | PTPN14 | + | Protein-tyrosine-phosphatase | 0 | PTPN14 |
| MT2A | 137.9798492 | 27.50145185 | 2.326878 | 0.00369392 | up | 61 | 8 | 19 | 128 | 100 | 185 | MT2A | - | Metallothionein-2 | 3.00E-14 | MT2A |
| THRSP | 20.26496824 | 354.2948679 | -4.12789 | 0.00372846 | down | 911 | 91 | 151 | 4 | 11 | 42 | THRSP | - | Uncharacterized protein | 1.00E-55 | THRSP |
| MOCS2 | 377.2894007 | 783.4246818 | -1.05412 | 0.00376393 | down | 723 | 607 | 983 | 431 | 353 | 369 | MOCS2 | + | Molybdopterin synthase sulfur carrier subunit | 5.00E-92 | MOCS2 |
| LOC100037702 | 59.12082342 | 104.5929419 | -0.82305 | 0.00376491 | down | 136 | 84 | 94 | 69 | 39 | 72 | LOC100037702 | + | Transmembrane 7 superfamily member 2 | 0 | TM7SF2 |
| SLC27A6 | 15.61857223 | 1.658238945 | 3.235539 | 0.00386684 | up | 2 | 1 | 2 | 27 | 17 | 6 | SLC27A6 | - | Uncharacterized protein | 0 | SLC27A6 |
| PCDH1 | 153.9721739 | 355.3643162 | -1.20663 | 0.00387547 | down | 339 | 281 | 430 | 262 | 104 | 122 | PCDH1 | - | Uncharacterized protein | 0 | PCDH1 |
| INCA1 | 132.7081852 | 73.07954868 | 0.860718 | 0.00388966 | up | 65 | 90 | 56 | 166 | 142 | 102 | INCA1 | + | Uncharacterized protein | 1.00E-93 | INCA1 |
| RHOBTB1 | 1024.024568 | 1540.884476 | -0.58951 | 0.0039018 | down | 1496 | 1389 | 1649 | 1337 | 875 | 951 | RHOBTB1 | - | Uncharacterized protein | 0 | RHOBTB1 |
| N4BP2 | 125.6999173 | 29.83757367 | 2.074782 | 0.0041176 | up | 49 | 9 | 35 | 150 | 172 | 67 | N4BP2 | + | Uncharacterized protein | 0 | N4BP2 |
| DVL1 | 1425.181295 | 2286.054664 | -0.68171 | 0.0041237 | down | 2359 | 2230 | 2135 | 1541 | 1153 | 1630 | DVL1 | + | Uncharacterized protein | 0 | DVL1 |
| HNRNPU | 1481.220881 | 913.1363352 | 0.697885 | 0.00412542 | up | 1117 | 706 | 912 | 1852 | 1649 | 1079 | HNRNPU | + | Uncharacterized protein | 0 | HNRNPU |
| CASK | 73.65457419 | 123.8895829 | -0.75021 | 0.00414342 | down | 123 | 96 | 148 | 84 | 68 | 73 | CASK | - | Uncharacterized protein | 0 | CASK |
| SLC38A4 | 16.30041846 | 41.30872297 | -1.34154 | 0.00426066 | down | 44 | 38 | 40 | 30 | 8 | 14 | SLC38A4 | + | Uncharacterized protein | 0 | SLC38A4 |
| LOC101115204 | 247.765987 | 161.7180683 | 0.615497 | 0.00435988 | up | 194 | 154 | 132 | 251 | 268 | 233 | LOC101115204 | + | selenoprotein O-like | 1.00E-158 | SELO |

| | | | | | | | | | | | | | | | | |
|---|---|---|---|---|---|---|---|---|---|---|---|---|---|---|---|---|
| LOC101113032 | 59.36153373 | 26.57388463 | 1.159519 | 0.00440077 | up | 33 | 18 | 29 | 53 | 72 | 54 | LOC101113032 | - | Uncharacterized protein | 0 | RNF146 |
| NMNAT2 | 24.64061862 | 74.52525449 | -1.59669 | 0.00442375 | down | 74 | 56 | 91 | 53 | 6 | 21 | NMNAT2 | - | Uncharacterized protein | 1.00E-170 | NMNAT2 |
| AQP3 | 86.69029318 | 7.988109203 | 3.439945 | 0.00443547 | up | 12 | 8 | 4 | 170 | 43 | 66 | AQP3 | + | Aquaporin 3 | 2.00E-133 | AQP3 |
| PA2G4 | 1422.570445 | 728.7797367 | 0.964945 | 0.00448039 | up | 791 | 704 | 655 | 1367 | 1724 | 1221 | PA2G4 | - | Uncharacterized protein | 0 | PA2G4 |
| RAB3A | 45.54037726 | 95.19409964 | -1.06373 | 0.00448703 | down | 104 | 99 | 77 | 56 | 25 | 58 | RAB3A | + | Rab3A | | |
| MEOX1 | 25.76562498 | 77.72098802 | -1.59286 | 0.00452901 | down | 121 | 56 | 60 | 43 | 26 | 13 | MEOX1 | - | Uncharacterized protein | 3.00E-130 | MEOX1 |
| MKL1 | 158.2914457 | 352.2580047 | -1.15405 | 0.00464145 | down | 330 | 369 | 329 | 235 | 79 | 178 | MKL1 | - | Uncharacterized protein | 4.00E-168 | MKL1 |
| NMNAT3 | 88.45141566 | 183.4607304 | -1.05251 | 0.00464461 | down | 206 | 141 | 200 | 153 | 45 | 82 | NMNAT3 | + | Uncharacterized protein | 8.00E-87 | NMNAT3 |
| STAC3 | 2569.446591 | 5060.113731 | -0.97771 | 0.00464877 | down | 6394 | 3858 | 4940 | 3201 | 1392 | 3258 | STAC3 | + | Uncharacterized protein | 0 | STAC3 |
| DHDDS | 92.08587615 | 54.61049044 | 0.753802 | 0.00471419 | up | 55 | 49 | 57 | 125 | 70 | 90 | DHDDS | - | Uncharacterized protein | 0 | DHDDS |
| LOC101114955 | 771.3872545 | 1537.491541 | -0.99505 | 0.00471726 | down | 1524 | 1669 | 1298 | 1065 | 626 | 703 | LOC101114955 | - | uncharacterized LOC101114955 | 2.00E-93 | GAMT |
| CWF19L2 | 99.35844642 | 54.31138499 | 0.871388 | 0.0047258 | up | 75 | 26 | 65 | 121 | 103 | 82 | CWF19L2 | + | Uncharacterized protein | 0 | CWF19L2 |
| RAD50 | 128.6799012 | 64.87120527 | 0.988137 | 0.00480186 | up | 80 | 38 | 78 | 155 | 151 | 91 | RAD50 | - | Uncharacterized protein | 0 | RAD50 |
| SPTBN2 | 1.575037416 | 13.65296704 | -3.11576 | 0.00480628 | down | 24 | 10 | 8 | 3 | 0 | 2 | SPTBN2 | - | Uncharacterized protein | 0 | SPTBN2 |
| WDR3 | 280.3574584 | 182.2835142 | 0.621083 | 0.00482846 | up | 195 | 141 | 206 | 302 | 318 | 236 | WDR3 | + | Uncharacterized protein | 0 | WDR3 |
| CA4 | 305.2311466 | 160.4150914 | 0.928092 | 0.00493966 | up | 210 | 165 | 102 | 385 | 234 | 319 | CA4 | - | Uncharacterized protein | 5.00E-111 | CA4 |
| C22H10orf137 | 174.3494004 | 84.77459433 | 1.040278 | 0.00501963 | up | 85 | 60 | 107 | 209 | 210 | 119 | C22H10orf137 | + | chromosome 22 open reading frame, human C10orf137 | 0 | EDRF1 |
| DNAJC15 | 85.25454159 | 129.3121866 | -0.60101 | 0.00503607 | down | 148 | 99 | 139 | 119 | 71 | 75 | DNAJC15 | + | Uncharacterized protein | 8.00E-55 | DNAJC15 |
| LOC101108849 | 268.3145162 | 1336.155241 | -2.31609 | 0.00504056 | down | 1367 | 694 | 1948 | 302 | 365 | 159 | LOC101108849 | + | Uncharacterized protein | 5.00E-146 | AKR1C4 |
| ADCY1 | 3.065328725 | 15.98590951 | -2.38269 | 0.00504588 | down | 13 | 12 | 22 | 7 | 0 | 3 | ADCY1 | - | Uncharacterized protein | 0 | ADCY1 |
| PAPSS2 | 45.73790911 | 10.539708 | 2.117556 | 0.00505225 | up | 13 | 5 | 14 | 47 | 66 | 27 | PAPSS2 | + | Adenylyl-sulfate kinase | 0 | PAPSS2 |
| JAM3 | 743.8513411 | 456.6192549 | 0.704023 | 0.00508015 | up | 492 | 385 | 477 | 795 | 639 | 823 | JAM3 | - | Uncharacterized protein | 2.00E-150 | JAM3 |
| BCL9L | 156.1731351 | 364.4586717 | -1.22261 | 0.00516096 | down | 368 | 377 | 323 | 287 | 91 | 121 | BCL9L | - | Uncharacterized protein | 0 | BCL9L |
| SMAD3 | 159.9461116 | 312.761389 | -0.96748 | 0.00531507 | down | 348 | 256 | 326 | 283 | 117 | 110 | SMAD3 | + | Smad3 | 0 | SMAD3 |
| ARX | 5.339118826 | 25.10630025 | -2.23338 | 0.00532583 | down | 20 | 17 | 37 | 5 | 4 | 7 | ARX | - | Uncharacterized protein | 5.00E-77 | ARX |
| MPP5 | 83.22505406 | 138.541829 | -0.73523 | 0.00533978 | down | 165 | 102 | 148 | 123 | 81 | 57 | MPP5 | + | Uncharacterized protein | 0 | MPP5 |
| IL11RA | 207.5430182 | 348.8706157 | -0.74928 | 0.00537337 | down | 355 | 336 | 335 | 264 | 138 | 235 | IL11RA | - | Uncharacterized protein | 0 | IL11RA |
| TFAP4 | 77.47353744 | 18.34788998 | 2.078089 | 0.00539248 | up | 23 | 22 | 9 | 66 | 112 | 56 | TFAP4 | - | Uncharacterized protein | 4.00E-152 | TFAP4 |
| RHBDD2 | 102.5370313 | 65.58065783 | 0.644803 | 0.00546314 | up | 59 | 69 | 63 | 135 | 88 | 94 | RHBDD2 | + | Uncharacterized protein | 0 | RHBDD2 |
| NDRG1 | 450.6306637 | 237.7062677 | 0.922766 | 0.00553639 | up | 367 | 182 | 174 | 517 | 379 | 479 | NDRG1 | + | Uncharacterized protein | 0 | NDRG1 |
| RTN4 | 1304.10679 | 2785.308239 | -1.09477 | 0.00555593 | down | 2653 | 2257 | 3312 | 1954 | 819 | 1295 | RTN4 | + | Reticulon | 1.00E-111 | RTN4 |
| PPAR | 201.0202621 | 322.606399 | -0.68243 | 0.0056039 | down | 315 | 272 | 365 | 266 | 160 | 195 | PPAR | + | | | |

| | | | | | | | | | | | | | | | | |
|---|---|---|---|---|---|---|---|---|---|---|---|---|---|---|---|---|
| HLCS | 112.2425632 | 217.3646475 | -0.9535 | 0.00563056 | down | 273 | 176 | 202 | 168 | 53 | 128 | HLCS | - | Uncharacterized protein | 0 | HLCS |
| PGM2L1 | 84.59072796 | 219.7124152 | -1.37704 | 0.00573128 | down | 313 | 180 | 170 | 160 | 60 | 52 | PGM2L1 | - | Uncharacterized protein | 0 | PGM2L1 |
| LOC101114981 | 6.606176485 | 26.27382538 | -1.99174 | 0.00573147 | down | 42 | 21 | 17 | 3 | 4 | 12 | LOC101114981 | - | dnaJ homolog subfamily A member 1-like | | |
| PACRG | 13.55773791 | 51.12591955 | -1.91494 | 0.00580677 | down | 39 | 38 | 73 | 13 | 15 | 13 | PACRG | + | PARK2 co-regulated | 4.00E-159 | PACRG |
| STMN1 | 123.6087483 | 204.2885074 | -0.72483 | 0.00581258 | down | 240 | 154 | 217 | 102 | 121 | 146 | STMN1 | + | Stathmin | 1.00E-77 | STMN1 |
| LOC101112287 | 433.9551814 | 212.2366628 | 1.031872 | 0.00585147 | up | 193 | 166 | 267 | 483 | 338 | 498 | LOC101112287 | - | Uncharacterized protein | 0 | SMS |
| ANKRD23 | 3673.936454 | 8727.326014 | -1.24821 | 0.00597372 | down | 9232 | 8134 | 8386 | 4550 | 1176 | 5443 | ANKRD23 | + | Uncharacterized protein | 1.00E-118 | ANKRD23 |
| LOC101114319 | 1049.335429 | 1785.569883 | -0.76691 | 0.00599805 | down | 1642 | 1587 | 2015 | 1200 | 900 | 1102 | LOC101114319 | - | phosphatidylethanolamine binding protein 1 pseudogene | | |
| LOC101107119 | 34.796538 | 100.8593343 | -1.53533 | 0.0060906 | down | 105 | 59 | 138 | 46 | 36 | 26 | LOC101107119 | + | Uncharacterized protein | 9.00E-149 | AKR1C3 |
| ZNF385A | 31.42710688 | 93.8859416 | -1.5789 | 0.00612187 | down | 130 | 45 | 112 | 45 | 27 | 26 | ZNF385A | + | Uncharacterized protein | 2.00E-174 | ZNF385A |
| TRIM3 | 172.3036764 | 102.67939 | 0.746807 | 0.00616865 | up | 108 | 110 | 83 | 178 | 158 | 186 | TRIM3 | + | Uncharacterized protein | 0 | TRIM3 |
| BROX | 147.676985 | 89.20293531 | 0.727282 | 0.00630041 | up | 108 | 54 | 107 | 199 | 133 | 126 | BROX | + | Uncharacterized protein | 0 | BROX |
| C1QTNF3 | 41.52403898 | 142.106955 | -1.77496 | 0.00636643 | down | 213 | 81 | 141 | 88 | 18 | 29 | C1QTNF3 | + | Uncharacterized protein | 9.00E-168 | C1QTNF3 |
| PCCA | 205.6498087 | 317.5831442 | -0.62694 | 0.00647558 | down | 377 | 250 | 322 | 285 | 143 | 209 | PCCA | + | Uncharacterized protein | 0 | PCCA |
| GTF3A | 266.8037974 | 147.4836349 | 0.855224 | 0.00652103 | up | 152 | 127 | 157 | 242 | 301 | 261 | GTF3A | - | Uncharacterized protein | 3.00E-169 | GTF3A |
| SLC25A42 | 237.1965437 | 110.3878584 | 1.103501 | 0.00662194 | up | 105 | 126 | 90 | 331 | 161 | 243 | SLC25A42 | - | Uncharacterized protein | 5.00E-103 | SLC25A42 |
| SIRPA | 185.6548876 | 352.2666831 | -0.92404 | 0.00684896 | down | 511 | 253 | 305 | 235 | 179 | 159 | SIRPA | - | Uncharacterized protein | 0 | SIRPA |
| ENAM | 38.59716929 | 5.116798107 | 2.915182 | 0.00714384 | up | 4 | 4 | 7 | 71 | 17 | 35 | ENAM | + | Uncharacterized protein | 0 | ENAM |
| MRPL16 | 440.2241838 | 249.9493142 | 0.816603 | 0.00722214 | up | 259 | 269 | 204 | 596 | 333 | 433 | MRPL16 | - | Uncharacterized protein | 3.00E-122 | MRPL16 |
| PIGZ | 32.75172632 | 102.1158457 | -1.64056 | 0.0072824 | down | 153 | 55 | 105 | 28 | 16 | 53 | PIGZ | - | Uncharacterized protein | 0 | PIGZ |
| HADHB | 4656.47436 | 2153.572991 | 1.112506 | 0.00742215 | up | 1366 | 2177 | 2660 | 5961 | 5229 | 3243 | HADHB | + | Uncharacterized protein | 0 | HADHB |
| PPM1J | 141.6196554 | 360.8402623 | -1.34934 | 0.00746312 | down | 297 | 410 | 336 | 190 | 95 | 152 | PPM1J | - | Uncharacterized protein | 0 | PPM1J |
| STK10 | 478.595967 | 211.6649347 | 1.177026 | 0.00750647 | up | 205 | 204 | 212 | 528 | 347 | 577 | STK10 | - | Uncharacterized protein | 0 | STK10 |
| AIG1 | 41.87854713 | 77.38400129 | -0.88582 | 0.00752946 | down | 78 | 63 | 88 | 48 | 55 | 26 | AIG1 | + | Uncharacterized protein | 8.00E-131 | AIG1 |
| RAI14 | 67.34306328 | 205.2797556 | -1.60799 | 0.00755091 | down | 217 | 242 | 140 | 112 | 32 | 68 | RAI14 | - | Uncharacterized protein | 0 | RAI14 |
| PCCB | 540.8106434 | 929.1569466 | -0.7808 | 0.00759932 | down | 855 | 946 | 912 | 532 | 589 | 517 | PCCB | - | Propionyl coenzyme A carboxylase beta polypeptide | 0 | PCCB |
| LOC101108329 | 7.003064816 | 76.33621961 | -3.44631 | 0.00765894 | down | 74 | 28 | 128 | 11 | 5 | 6 | LOC101108329 | + | prostaglandin F synthase 1-like | | |
| CAST | 1951.309208 | 1068.649835 | 0.868653 | 0.00776369 | up | 1072 | 845 | 1248 | 2344 | 1490 | 2135 | CAST | + | Calpastatin isoform III | 0 | CAST |
| RAB31 | 282.4836092 | 448.7660558 | -0.6678 | 0.00777541 | down | 479 | 326 | 532 | 323 | 291 | 251 | RAB31 | + | Uncharacterized protein | 9.00E-103 | RAB31 |
| LRRC17 | 14.00199568 | 46.2634806 | -1.72424 | 0.00780959 | down | 50 | 33 | 55 | 35 | 10 | 2 | LRRC17 | + | Uncharacterized protein | 0 | LRRC17 |
| COL21A1 | 18.34876538 | 90.24595975 | -2.29818 | 0.00781512 | down | 152 | 41 | 87 | 32 | 7 | 19 | COL21A1 | + | Uncharacterized protein | 0 | COL21A1 |
| MX2 | 31.82292609 | 96.10986796 | -1.59462 | 0.00788063 | down | 88 | 87 | 107 | 35 | 63 | 1 | MX2 | + | Myxovirus resistance 2 | 0 | MX2 |

| | | | | | | | | | | | | | | | | |
|---|---|---|---|---|---|---|---|---|---|---|---|---|---|---|---|---|
| PIK3R2 | 91.5192105 | 147.4821394 | -0.68839 | 0.00802944 | down | 178 | 117 | 146 | 126 | 59 | 98 | PIK3R2 | - | Uncharacterized protein | 0 | PIK3R2 |
| ISG20L2 | 362.487071 | 223.0604274 | 0.700495 | 0.00807485 | up | 251 | 233 | 173 | 364 | 401 | 335 | ISG20L2 | - | Uncharacterized protein | 5.00E-176 | ISG20L2 |
| ZNF175 | 79.73625139 | 32.05187957 | 1.314827 | 0.00814719 | up | 23 | 15 | 57 | 97 | 90 | 59 | ZNF175 | + | Uncharacterized protein | 0 | ZNF175 |
| LRRC39 | 454.5346012 | 866.9276335 | -0.93152 | 0.00815682 | down | 838 | 671 | 1056 | 634 | 431 | 351 | LRRC39 | - | leucine rich repeat containing 39 | 2.00E-167 | LRRC39 |
| HSP90AB1 | 11797.09423 | 6339.885693 | 0.895903 | 0.00815883 | up | 9127 | 5448 | 4536 | 12651 | 13409 | 9948 | HSP90AB1 | + | Uncharacterized protein | 0 | HSP90AB1 |
| CD2BP2 | 998.1260003 | 600.636606 | 0.73273 | 0.00825996 | up | 585 | 599 | 576 | 965 | 1116 | 940 | CD2BP2 | - | Uncharacterized protein | 4.00E-149 | CD2BP2 |
| ANXA9 | 9.61391492 | 0.97748289 | 3.297981 | 0.00831648 | up | 1 | 0 | 2 | 8 | 14 | 7 | ANXA9 | + | Annexin | 2.00E-166 | ANXA9 |
| ANGPTL4 | 810.5107478 | 187.0153882 | 2.115674 | 0.00833026 | up | 97 | 307 | 116 | 798 | 544 | 1092 | ANGPTL4 | + | Uncharacterized protein | 9.00E-152 | ANGPTL4 |
| CCDC62 | 49.70610818 | 21.69918755 | 1.195782 | 0.0084428 | up | 24 | 14 | 27 | 73 | 52 | 31 | CCDC62 | - | Uncharacterized protein | 0 | CCDC62 |
| CTXN3 | 7.650029005 | 150.660469 | -4.29969 | 0.0086161 | down | 48 | 33 | 362 | 12 | 5 | 7 | CTXN3 | - | Uncharacterized protein | 7.00E-39 | CTXN3 |
| SH3GLB2 | 394.1555439 | 250.6841544 | 0.652894 | 0.00862667 | up | 302 | 233 | 210 | 430 | 339 | 429 | SH3GLB2 | + | Uncharacterized protein | 0 | SH3GLB2 |
| CBS | 273.2813509 | 111.9863728 | 1.287064 | 0.00865666 | up | 99 | 102 | 127 | 279 | 361 | 194 | CBS | - | Cystathionine beta-synthase | 0 | CBS |
| EPC2 | 155.1711211 | 90.6420587 | 0.775608 | 0.00865781 | up | 122 | 63 | 89 | 180 | 180 | 117 | EPC2 | - | Enhancer of polycomb homolog | 0 | EPC2 |
| TRMT1 | 426.4312666 | 192.8552158 | 1.144795 | 0.00865803 | up | 231 | 194 | 146 | 357 | 479 | 442 | TRMT1 | + | Uncharacterized protein | 0 | TRMT1 |
| LOC101122774 | 20.31681376 | 4.771651066 | 2.090114 | 0.00869695 | up | 5 | 5 | 4 | 32 | 17 | 15 | LOC101122774 | + | voltage-dependent N-type calcium channel subunit alpha-1B-like | 0 | CACNA1B |
| LOC101123553 | 239.1881557 | 397.8277763 | -0.734 | 0.00873138 | down | 517 | 367 | 304 | 310 | 200 | 228 | LOC101123553 | + | Uncharacterized protein | 0 | P4HA2 |
| ADAMTS18 | 0.686293795 | 10.57874325 | -3.9462 | 0.00873843 | down | 7 | 7 | 17 | 0 | 2 | 0 | ADAMTS18 | - | Uncharacterized protein | 0 | ADAMTS18 |
| DPF3 | 55.73313162 | 121.3086753 | -1.12208 | 0.00875409 | down | 176 | 73 | 121 | 65 | 42 | 63 | DPF3 | - | Uncharacterized protein | 2.00E-165 | DPF3 |
| IKKBETA | 265.0470513 | 174.3848152 | 0.603974 | 0.00876525 | up | 239 | 142 | 144 | 302 | 235 | 272 | IKKBETA | + | IKB kinase beta | 0 | IKBKB |
| EIF4G3 | 1116.885033 | 502.4953739 | 1.152298 | 0.00876558 | up | 550 | 418 | 524 | 1188 | 1434 | 795 | EIF4G3 | + | Uncharacterized protein | 0 | EIF4G3 |
| MAB21L1 | 42.11366947 | 78.97275395 | -0.90707 | 0.00880233 | down | 78 | 83 | 70 | 63 | 37 | 32 | MAB21L1 | + | Uncharacterized protein | 0 | MAB21L1 |
| MED6 | 115.9654619 | 73.34455275 | 0.660933 | 0.00886709 | up | 80 | 59 | 79 | 128 | 135 | 92 | MED6 | - | Uncharacterized protein | 2.00E-138 | MED6 |
| PDHB | 1409.303438 | 2302.230298 | -0.70805 | 0.00888725 | down | 2201 | 1912 | 2678 | 1694 | 1249 | 1379 | PDHB | + | Uncharacterized protein | 0 | PDHB |
| AMY2B | 310.7383607 | 50.29568731 | 2.627194 | 0.00892927 | up | 44 | 25 | 81 | 263 | 479 | 198 | AMY2B | + | Alpha-amylase | 0 | AMY2B |
| ALG3 | 104.7675859 | 60.60578791 | 0.789665 | 0.00909605 | up | 61 | 67 | 49 | 106 | 98 | 113 | ALG3 | + | Uncharacterized protein | 0 | ALG3 |
| LOC101119560 | 129.553563 | 329.2842777 | -1.34578 | 0.00910599 | down | 470 | 200 | 333 | 192 | 55 | 155 | LOC101119560 | - | uncharacterized LOC101119560 | 4.00E-105 | PITX1 |
| SARS2 | 150.9951123 | 83.69766868 | 0.851243 | 0.00923271 | up | 109 | 77 | 64 | 141 | 155 | 159 | SARS2 | - | Uncharacterized protein | 0 | SARS2 |
| PC | 140.2922277 | 463.209737 | -1.72323 | 0.00935364 | down | 677 | 449 | 265 | 214 | 80 | 144 | PC | - | Pyruvate carboxylase | 0 | PC |
| TMEM86A | 75.55870306 | 5.310478927 | 3.830684 | 0.00939108 | up | 0 | 4 | 11 | 25 | 156 | 41 | TMEM86A | - | Uncharacterized protein | 3.00E-122 | TMEM86A |
| PCK1 | 7.494920107 | 97.48680577 | -3.70122 | 0.00950295 | down | 240 | 8 | 70 | 6 | 2 | 14 | PCK1 | - | Phosphoenolpyruvate carboxykinase | | |
| MRPS7 | 535.2088045 | 882.9331371 | -0.7222 | 0.00976419 | down | 1070 | 812 | 744 | 694 | 360 | 592 | MRPS7 | - | Uncharacterized protein | 3.00E-122 | MRPS7 |
| CRY2 | 214.9062504 | 460.1215124 | -1.09831 | 0.00981801 | down | 479 | 320 | 572 | 335 | 161 | 179 | CRY2 | + | Cryptochrome 2 | 0 | CRY2 |

| | | | | | | | | | | | | | | | | |
|---|---|---|---|---|---|---|---|---|---|---|---|---|---|---|---|---|
| WDR62 | 68.4605715 | 29.06568824 | 1.235956 | 0.00993693 | up | 21 | 30 | 33 | 56 | 79 | 70 | WDR62 | + | Uncharacterized protein | 0 | WDR62 |
| ASAP3 | 71.64829384 | 160.1385475 | -1.16032 | 0.01031589 | down | 207 | 133 | 140 | 120 | 22 | 83 | ASAP3 | + | Uncharacterized protein | 0 | ASAP3 |
| C9H8orf22 | 218.8311855 | 47.14696093 | 2.214582 | 0.01036371 | up | 9 | 54 | 69 | 140 | 297 | 212 | C9H8orf22 | + | chromosome 9 open reading frame, human C8orf22 | 2.00E-23 | C8orf22 |
| LOC101121068 | 29.68005411 | 75.45972205 | -1.34621 | 0.01044043 | down | 63 | 80 | 76 | 44 | 11 | 37 | LOC101121068 | + | uncharacterized LOC101121068 | | |
| GCNT1 | 27.09219736 | 64.34325782 | -1.24791 | 0.01055057 | down | 64 | 47 | 80 | 40 | 11 | 33 | GCNT1 | - | Uncharacterized protein | 0 | GCNT1 |
| TRIM11 | 120.3221673 | 78.00346669 | 0.625292 | 0.01072919 | up | 92 | 69 | 71 | 120 | 118 | 126 | TRIM11 | + | tripartite motif containing 11 | 0 | TRIM11 |
| EEPD1 | 381.9898375 | 583.1753286 | -0.6104 | 0.01114391 | down | 705 | 538 | 491 | 506 | 285 | 388 | EEPD1 | - | Uncharacterized protein | 0 | EEPD1 |
| WDR20 | 341.2610149 | 192.8962411 | 0.823051 | 0.01124285 | up | 234 | 183 | 156 | 319 | 392 | 320 | WDR20 | + | WD repeat domain 20 | 0 | WDR20 |
| ASB12 | 740.3091534 | 1588.350596 | -1.10133 | 0.01129986 | down | 1341 | 1592 | 1691 | 708 | 588 | 928 | ASB12 | - | Uncharacterized protein | 2.00E-157 | ASB12 |
| HIGD1B | 0.28110904 | 10.16188438 | -5.17589 | 0.01135247 | down | 18 | 2 | 12 | 1 | 0 | 0 | HIGD1B | + | Uncharacterized protein | 2.00E-45 | HIGD1B |
| AGL | 1334.639865 | 4059.580292 | -1.60488 | 0.01138939 | down | 4542 | 2550 | 5085 | 2648 | 880 | 788 | AGL | + | Uncharacterized protein | 0 | AGL |
| UTP3 | 109.437631 | 61.85597594 | 0.823124 | 0.01140278 | up | 57 | 45 | 81 | 113 | 124 | 96 | UTP3 | + | Uncharacterized protein | 0 | UTP3 |
| CD4 | 10.81893472 | 28.05840196 | -1.37487 | 0.01159258 | down | 39 | 27 | 18 | 7 | 13 | 12 | CD4 | - | T-cell surface glycoprotein CD4 | 1.00E-145 | CD4 |
| FOSL1 | 27.59045316 | 6.142548123 | 2.16726 | 0.01160788 | up | 12 | 5 | 2 | 46 | 15 | 26 | FOSL1 | - | Uncharacterized protein | 5.00E-108 | FOSL1 |
| SLC46A3 | 320.3485123 | 213.2516149 | 0.587086 | 0.01171579 | up | 209 | 198 | 220 | 328 | 361 | 285 | SLC46A3 | + | Uncharacterized protein | 0 | SLC46A3 |
| ATP8B1 | 151.4525417 | 43.18546515 | 1.810248 | 0.01175855 | up | 66 | 35 | 30 | 229 | 177 | 72 | ATP8B1 | - | Phospholipid-translocating ATPase | 0 | ATP8B1 |
| IDH2 | 4927.240112 | 7686.788795 | -0.6416 | 0.0118348 | down | 9060 | 7634 | 6057 | 5869 | 4717 | 4534 | IDH2 | - | Uncharacterized protein | 0 | IDH2 |
| CDK8 | 99.05491423 | 53.44418995 | 0.890195 | 0.01222895 | up | 82 | 37 | 44 | 113 | 114 | 77 | CDK8 | - | Uncharacterized protein | 0 | CDK8 |
| FGFR4 | 24.80399543 | 67.66157266 | -1.44776 | 0.01229511 | down | 85 | 37 | 83 | 33 | 9 | 34 | FGFR4 | + | Fibroblast growth factor receptor | 0 | FGFR4 |
| UBXN10 | 12.17490096 | 2.184349437 | 2.478634 | 0.01236574 | up | 1 | 4 | 1 | 8 | 14 | 14 | UBXN10 | - | UBX domain protein 10 | 4.00E-112 | UBXN10 |
| DCLK3 | 84.43808519 | 140.4281092 | -0.73387 | 0.01258324 | down | 184 | 96 | 144 | 120 | 71 | 72 | DCLK3 | + | Uncharacterized protein | 6.00E-151 | DCLK3 |
| RBKS | 50.96010824 | 18.5700318 | 1.456392 | 0.01264814 | up | 18 | 14 | 23 | 73 | 29 | 56 | RBKS | - | Uncharacterized protein | 4.00E-164 | RBKS |
| DPP6 | 22.7808799 | 0 | 20 | 0.01268674 | up | 0 | 0 | 0 | 31 | 25 | 15 | DPP6 | + | Uncharacterized protein | 0 | DPP6 |
| LGALS12 | 1.39529584 | 15.84670142 | -3.50554 | 0.01290613 | down | 30 | 12 | 7 | 0 | 3 | 1 | LGALS12 | + | Galectin | 3.00E-160 | LGALS12 |
| LOC101105659 | 73.77810822 | 170.0785256 | -1.20494 | 0.01293571 | down | 209 | 181 | 113 | 113 | 51 | 67 | LOC101105659 | - | basement membrane-specific heparan sulfate proteoglycan core protein-like | 2.00E-119 | FGFRL1 |
| HSF4 | 182.3378437 | 112.4849054 | 0.696883 | 0.01301648 | up | 105 | 127 | 95 | 231 | 195 | 138 | HSF4 | + | Uncharacterized protein | 0 | HSF4 |
| NCOA5 | 310.5349843 | 182.0044504 | 0.770782 | 0.01323162 | up | 202 | 165 | 172 | 327 | 378 | 243 | NCOA5 | - | Uncharacterized protein | 0 | NCOA5 |
| SLCO2B1 | 236.340886 | 487.9911909 | -1.04599 | 0.01330691 | down | 402 | 453 | 569 | 321 | 236 | 178 | SLCO2B1 | + | Uncharacterized protein | 0 | SLCO2B1 |
| MIIP | 188.4382235 | 122.8173321 | 0.617577 | 0.01333118 | up | 120 | 122 | 118 | 245 | 148 | 188 | MIIP | - | migration and invasion inhibitory protein | 7.00E-125 | MIIP |
| LIPG | 188.1968859 | 48.02262244 | 1.970457 | 0.01338105 | up | 55 | 47 | 40 | 241 | 93 | 242 | LIPG | + | Endothelial lipase | 0 | LIPG |
| TEX10 | 134.9927663 | 70.87167211 | 0.929601 | 0.01338528 | up | 94 | 42 | 79 | 138 | 162 | 111 | TEX10 | + | testis expressed 10 | 0 | TEX10 |

| | | | | | | | | | | | | | | | | |
|---|---|---|---|---|---|---|---|---|---|---|---|---|---|---|---|---|
| BDH1 | 17.85688158 | 60.07323631 | -1.75024 | 0.01352225 | down | 77 | 27 | 79 | 20 | 9 | 25 | BDH1 | - | Uncharacterized protein | 2.00E-169 | BDH1 |
| PPP2R3A | 364.1065841 | 740.6440765 | -1.02442 | 0.01356942 | down | 795 | 517 | 898 | 514 | 221 | 393 | PPP2R3A | - | Uncharacterized protein | 0 | PPP2R3A |
| LOC101110539 | 87.92105755 | 132.3647674 | -0.59024 | 0.01362289 | down | 140 | 105 | 148 | 121 | 75 | 77 | LOC101110539 | - | lysosome membrane protein 2-like | | |
| ZNF830 | 152.7092359 | 96.00666887 | 0.669581 | 0.01372326 | up | 92 | 89 | 101 | 154 | 176 | 134 | ZNF830 | - | Uncharacterized protein | 3.00E-163 | ZNF830 |
| CCT6A | 733.180231 | 409.8775777 | 0.838975 | 0.0137569 | up | 538 | 293 | 405 | 787 | 881 | 573 | CCT6A | + | Uncharacterized protein | 0 | CCT6A |
| SAYSD1 | 49.1704187 | 21.67072655 | 1.182043 | 0.01376604 | up | 25 | 22 | 17 | 48 | 40 | 60 | SAYSD1 | - | Uncharacterized protein | 9.00E-55 | SAYSD1 |
| RTN4IP1 | 99.05481444 | 204.9477381 | -1.04896 | 0.01385364 | down | 262 | 177 | 174 | 142 | 38 | 126 | RTN4IP1 | + | Uncharacterized protein | 0 | RTN4IP1 |
| STC1 | 121.4288988 | 47.21537567 | 1.362783 | 0.01388986 | up | 67 | 42 | 33 | 187 | 77 | 116 | STC1 | + | Uncharacterized protein | | |
| PPP2R2D | 67.58639651 | 1.797140875 | 5.232957 | 0.01391466 | up | 1 | 3 | 1 | 67 | 12 | 122 | PPP2R2D | + | protein phosphatase 2, regulatory subunit B, delta | 2.00E-51 | PPP2R2D |
| TBX3 | 25.24078357 | 50.46029481 | -0.99939 | 0.01392126 | down | 73 | 37 | 43 | 26 | 32 | 19 | TBX3 | + | Uncharacterized protein | 0 | TBX3 |
| ABHD11 | 126.6597507 | 196.6984518 | -0.63503 | 0.01392398 | down | 261 | 175 | 153 | 151 | 126 | 112 | ABHD11 | + | Uncharacterized protein | 2.00E-128 | ABHD11 |
| KCP | 26.05500194 | 69.88752438 | -1.42347 | 0.01406914 | down | 111 | 46 | 57 | 18 | 26 | 33 | KCP | - | Uncharacterized protein | 0 | KCP |
| KHNYN | 167.2166719 | 100.4511421 | 0.735225 | 0.01412772 | up | 89 | 116 | 86 | 231 | 152 | 137 | KHNYN | - | KH and NYN domain containing | 0 | KHNYN |
| SLC27A1 | 197.0205452 | 396.6039886 | -1.00935 | 0.01422012 | down | 393 | 434 | 331 | 297 | 156 | 164 | SLC27A1 | - | Uncharacterized protein | 0 | SLC27A1 |
| LOC101118085 | 1301.37185 | 2086.500186 | -0.68105 | 0.01425168 | down | 2117 | 2050 | 1963 | 1862 | 1025 | 1165 | LOC101118085 | - | ATP synthase subunit alpha, mitochondrial-like | | |
| PLIN2 | 724.6671744 | 237.154381 | 1.611492 | 0.01436325 | up | 220 | 209 | 268 | 530 | 765 | 856 | PLIN2 | - | Perilipin | 0 | PLIN2 |
| DGAT2 | 39.34193718 | 641.442781 | -4.02718 | 0.01497868 | down | 1676 | 144 | 274 | 69 | 40 | 17 | DGAT2 | + | Uncharacterized protein | 0 | DGAT2 |
| TEF | 253.365987 | 422.3297778 | -0.73715 | 0.01500502 | down | 391 | 358 | 494 | 320 | 215 | 245 | TEF | + | Thyrotrophic embryonic factor isoform 1 | 1.00E-172 | TEF |
| QSOX2 | 388.7050649 | 192.348146 | 1.014956 | 0.01506825 | up | 167 | 205 | 187 | 398 | 489 | 298 | QSOX2 | + | quiescin Q6 sulfhydryl oxidase 2 | 0 | QSOX2 |
| PPT2 | 957.0734034 | 573.9544236 | 0.737693 | 0.01526819 | up | 553 | 557 | 573 | 913 | 959 | 1015 | PPT2 | - | Uncharacterized protein | 4.00E-161 | PPT2 |
| LGI4 | 182.7924506 | 298.7877559 | -0.70891 | 0.01531915 | down | 306 | 265 | 311 | 229 | 118 | 213 | LGI4 | - | Uncharacterized protein | 0 | LGI4 |
| LOC101123148 | 89.92427308 | 145.045574 | -0.68972 | 0.01533487 | down | 163 | 100 | 171 | 116 | 86 | 76 | LOC101123148 | + | L-lactate dehydrogenase B chain-like | 2.00E-177 | LDHB |
| LOC101120455 | 105.154453 | 210.9274097 | -1.00424 | 0.01548375 | down | 198 | 218 | 200 | 68 | 110 | 132 | LOC101120455 | - | histone H2B type 2-F-like | 1.00E-68 | HIST2H2BF |
| HSDL2 | 796.6074396 | 307.0053194 | 1.375605 | 0.01550288 | up | 209 | 237 | 450 | 756 | 1023 | 637 | HSDL2 | - | Uncharacterized protein | 0 | HSDL2 |
| TCEAL1 | 103.5653458 | 43.30138162 | 1.258056 | 0.01559778 | up | 58 | 29 | 44 | 83 | 124 | 103 | TCEAL1 | + | transcription elongation factor A (SII)-like 1 | 4.00E-53 | TCEAL1 |
| SERPINF1 | 553.7479315 | 998.3276588 | -0.85028 | 0.0160024 | down | 1276 | 673 | 1062 | 778 | 374 | 565 | SERPINF1 | + | SERPINF1 | 0 | SERPINF1 |
| SLC6A6 | 8.191748363 | 23.15420765 | -1.49903 | 0.01609229 | down | 33 | 18 | 19 | 4 | 11 | 9 | SLC6A6 | - | Transporter | 8.00E-111 | SLC6A6 |
| CXCL14 | 24.67607086 | 54.80616129 | -1.15123 | 0.01657394 | down | 60 | 36 | 68 | 41 | 17 | 20 | CXCL14 | - | Uncharacterized protein | 5.00E-44 | CXCL14 |
| ZNF768 | 516.7708882 | 951.8444268 | -0.8812 | 0.01661736 | down | 1080 | 1008 | 715 | 603 | 416 | 559 | ZNF768 | - | Uncharacterized protein | 0 | ZNF768 |
| FNBP1L | 108.0811231 | 56.65643251 | 0.931803 | 0.01663167 | up | 92 | 28 | 55 | 143 | 104 | 88 | FNBP1L | + | Uncharacterized protein | 0 | FNBP1L |
| CCDC41 | 94.90003138 | 56.94829613 | 0.736756 | 0.01667516 | up | 68 | 39 | 64 | 104 | 105 | 81 | CCDC41 | - | Uncharacterized protein | 0 | CEP83 |

| | | | | | | | | | | | | | | | | |
|---|---|---|---|---|---|---|---|---|---|---|---|---|---|---|---|---|
| FOXO3 | 1304.648395 | 292.1399396 | 2.15893 | 0.01694808 | up | 301 | 279 | 280 | 816 | 1691 | 1353 | FOXO3 | - | Forkhead box O3 | 0 | FOXO3 |
| DHX34 | 72.87359854 | 42.29980638 | 0.784745 | 0.01697412 | up | 47 | 48 | 29 | 90 | 80 | 55 | DHX34 | + | Uncharacterized protein | 0 | DHX34 |
| ATP5A1 | 7191.29801 | 11921.13286 | -0.7292 | 0.01702261 | down | 11882 | 10717 | 12526 | 10677 | 5714 | 6093 | ATP5A1 | - | ATP synthase subunit alpha | 0 | ATP5A1 |
| PTPN3 | 454.2008626 | 155.5045384 | 1.546374 | 0.01714043 | up | 93 | 158 | 196 | 403 | 615 | 355 | PTPN3 | + | Tyrosine-protein phosphatase non-receptor type | 0 | PTPN3 |
| LPP | 86.81989985 | 44.6458077 | 0.959501 | 0.01716874 | up | 75 | 24 | 39 | 103 | 94 | 70 | LPP | - | Uncharacterized protein | 0 | LPP |
| UCP2 | 520.489767 | 227.466513 | 1.194216 | 0.0171968 | up | 281 | 234 | 159 | 644 | 647 | 321 | UCP2 | - | uncoupling protein 2 (mitochondrial, proton carrier) | | |
| LOC101120670 | 1544.277872 | 373.8100475 | 2.046555 | 0.01720657 | up | 412 | 336 | 359 | 990 | 1928 | 1652 | LOC101120670 | - | uncharacterized LOC101120670 | 3.00E-81 | MAX |
| KCNQ5 | 160.3008764 | 85.95529607 | 0.899124 | 0.01819141 | up | 79 | 57 | 119 | 219 | 163 | 117 | KCNQ5 | - | Potassium voltage-gated channel subfamily Q member 5 | 0 | KCNQ5 |
| CXCR3 | 4.477245921 | 19.52207995 | -2.12442 | 0.0186567 | down | 27 | 22 | 9 | 6 | 6 | 2 | CXCR3 | - | Uncharacterized protein | 8.00E-169 | CXCR3 |
| LOC101120779 | 79.7899358 | 43.3352475 | 0.880666 | 0.01890003 | up | 44 | 45 | 38 | 71 | 88 | 81 | LOC101120779 | - | ferritin, heavy polypeptide 1 pseudogene | | |
| CASQ2 | 248.0998682 | 620.41971 | -1.32232 | 0.01900703 | down | 722 | 359 | 788 | 390 | 234 | 159 | CASQ2 | - | Calsequestrin | 0 | CASQ2 |
| LOC100125616 | 2.025638672 | 43.06264834 | -4.40999 | 0.01914295 | down | 0 | 7 | 118 | 2 | 0 | 4 | LOC100125616 | - | Myodulin | 2.00E-175 | TNMD |
| WBSCR17 | 13.49346201 | 66.21141578 | -2.29482 | 0.01922547 | down | 100 | 21 | 84 | 7 | 8 | 24 | WBSCR17 | - | Williams-Beuren syndrome chromosome region 17 | 0 | WBSCR17 |
| KCNS3 | 421.8590394 | 1395.093535 | -1.72553 | 0.01939371 | down | 1110 | 1631 | 1280 | 621 | 114 | 569 | KCNS3 | + | Uncharacterized protein | 0 | KCNS3 |
| UBXN8 | 49.38813565 | 16.20774517 | 1.607481 | 0.01946115 | up | 24 | 6 | 20 | 39 | 64 | 45 | UBXN8 | + | UBX domain protein 8 | 3.00E-109 | UBXN8 |
| NEURL | 377.1765864 | 595.3395745 | -0.65847 | 0.01955253 | down | 595 | 606 | 544 | 422 | 311 | 415 | NEURL | + | Uncharacterized protein | 0 | NEURL1 |
| ACSM3 | 0.646964188 | 13.62967541 | -4.39692 | 0.0195692 | down | 33 | 4 | 7 | 1 | 0 | 1 | ACSM3 | + | Uncharacterized protein | 0 | ACSM3 |
| LOC101106304 | 398.2632854 | 193.8034252 | 1.039128 | 0.01971261 | up | 232 | 148 | 200 | 384 | 507 | 318 | LOC101106304 | - | T-complex protein 1 subunit beta-like | | |
| PSMC1 | 5260.111006 | 3139.922455 | 0.744364 | 0.01987851 | up | 3112 | 3247 | 2834 | 5426 | 6115 | 4473 | PSMC1 | + | Uncharacterized protein | 0 | PSMC1 |
| ACP5 | 388.3466223 | 233.8721929 | 0.731625 | 0.01994591 | up | 232 | 240 | 213 | 393 | 459 | 329 | ACP5 | - | Uncharacterized protein | 1.00E-163 | ACP5 |
| FRG1 | 185.8667742 | 108.2866664 | 0.779413 | 0.02004817 | up | 113 | 73 | 137 | 201 | 216 | 151 | FRG1 | + | FSHD region gene 1 | 2.00E-135 | FRG1 |
| SLC12A2 | 81.5420206 | 256.4203237 | -1.6529 | 0.02008413 | down | 351 | 125 | 307 | 152 | 63 | 47 | SLC12A2 | - | Uncharacterized protein | 0 | SLC12A2 |
| LRCH4 | 625.6822096 | 322.128956 | 0.957792 | 0.02047368 | up | 276 | 386 | 268 | 620 | 567 | 702 | LRCH4 | + | Uncharacterized protein | 0 | LRCH4 |
| LOC101106865 | 111.8739996 | 383.4421946 | -1.77713 | 0.0208315 | down | 551 | 161 | 466 | 146 | 103 | 97 | LOC101106865 | + | Uncharacterized protein | 1.00E-147 | AKR1C1 |
| ARHGEF37 | 49.32748053 | 95.06201563 | -0.94648 | 0.02085798 | down | 79 | 92 | 106 | 68 | 39 | 46 | ARHGEF37 | + | Uncharacterized protein | 0 | ARHGEF37 |
| ST6GALNAC4 | 107.0321958 | 191.6966517 | -0.84078 | 0.02106978 | down | 184 | 171 | 209 | 117 | 70 | 137 | ST6GALNAC4 | + | Uncharacterized protein | 5.00E-164 | ST6GALNAC 4 |
| LOC101117646 | 5763.994199 | 9731.29392 | -0.75556 | 0.02114096 | down | 10624 | 8950 | 9203 | 5884 | 4208 | 7287 | LOC101117646 | - | uncharacterized LOC101117646 | 2.00E-67 | ANK1 |
| KIAA1024 | 1.097565444 | 9.91039841 | -3.17464 | 0.02126459 | down | 18 | 4 | 9 | 0 | 0 | 3 | KIAA1024 | + | KIAA1024 ortholog | 0 | KIAA1024 |
| EXD3 | 41.36533782 | 19.71273451 | 1.069294 | 0.02127681 | up | 16 | 22 | 19 | 64 | 34 | 32 | EXD3 | + | Uncharacterized protein | 0 | EXD3 |
| PDE4B | 74.50132252 | 470.9403784 | -2.66021 | 0.02153714 | down | 793 | 419 | 222 | 180 | 43 | 25 | PDE4B | + | Uncharacterized protein | 0 | PDE4B |
| LOC101105651 | 312.9768411 | 678.2624846 | -1.11579 | 0.0217991 | down | 774 | 703 | 523 | 535 | 187 | 269 | LOC101105651 | - | Uncharacterized protein | 1.00E-175 | CYP2D6 |

| | | | | | | | | | | | | | | | | |
|---|---|---|---|---|---|---|---|---|---|---|---|---|---|---|---|---|
| DAGLA | 18.86556696 | 40.94361672 | -1.11788 | 0.02183243 | down | 45 | 31 | 46 | 34 | 9 | 17 | DAGLA | + | Uncharacterized protein | 0 | DAGLA |
| C4H7orf31 | 20.27581632 | 3.461738504 | 2.550191 | 0.02188102 | up | 1 | 2 | 7 | 12 | 29 | 19 | C4H7orf31 | + | chromosome 4 open reading frame, human C7orf31 | 0 | C7orf31 |
| PRKACA | 1622.440084 | 2453.779664 | -0.59684 | 0.0221068 | down | 2742 | 2326 | 2188 | 2033 | 1183 | 1763 | PRKACA | + | cAMP-dependent protein kinase catalytic subunit alpha | 0 | PRKACA |
| PLXNA1 | 112.0365212 | 218.0994171 | -0.96102 | 0.0221745 | down | 311 | 180 | 167 | 147 | 76 | 122 | PLXNA1 | - | Uncharacterized protein | 0 | PLXNA1 |
| LOC101120582 | 69.98413403 | 32.5652727 | 1.103694 | 0.02230923 | up | 26 | 22 | 48 | 75 | 86 | 53 | LOC101120582 | - | eukaryotic translation initiation factor 2, subunit 2 beta, 38kDa pseudogene | | |
| FAM20B | 124.3273154 | 252.9230119 | -1.02456 | 0.02261886 | down | 262 | 217 | 269 | 230 | 95 | 74 | FAM20B | + | Uncharacterized protein | 0 | FAM20B |
| DDO | 1630.251523 | 371.9829266 | 2.131786 | 0.02280982 | up | 248 | 430 | 388 | 1029 | 2137 | 1661 | DDO | + | Uncharacterized protein | 6.00E-172 | DDO |
| LOC101108705 | 907.6684326 | 1735.212775 | -0.93488 | 0.02311561 | down | 1696 | 1726 | 1664 | 1367 | 522 | 941 | LOC101108705 | + | glutathione S-transferase Mu 1-like | 1.00E-107 | GSTM1 |
| RUVBL1 | 1308.020392 | 588.2095698 | 1.152983 | 0.02341512 | up | 799 | 508 | 459 | 1108 | 1355 | 1453 | RUVBL1 | + | Uncharacterized protein | 0 | RUVBL1 |
| PARP3 | 282.7152961 | 73.57573803 | 1.942048 | 0.02343484 | up | 37 | 71 | 103 | 502 | 225 | 176 | PARP3 | - | poly (ADP-ribose) polymerase family, member 3 | 0 | PARP3 |
| DLGAP4 | 499.4010865 | 988.2007237 | -0.98461 | 0.02345896 | down | 831 | 1016 | 1026 | 474 | 454 | 575 | DLGAP4 | + | Uncharacterized protein | 0 | DLGAP4 |
| C18H15orf39 | 56.91547168 | 29.25469425 | 0.960153 | 0.02348131 | up | 44 | 21 | 24 | 81 | 43 | 53 | C18H15orf39 | - | chromosome 18 open reading frame, human C15orf39 | 0 | C15orf39 |
| PRKAG3 | 519.9357598 | 1091.878498 | -1.07041 | 0.02354311 | down | 1308 | 772 | 1196 | 405 | 355 | 777 | PRKAG3 | - | 5'-AMP-activated protein kinase subunit gamma-3 | 0 | PRKAG3 |
| CACHD1 | 255.5905403 | 395.7754756 | -0.63085 | 0.02377574 | down | 369 | 390 | 399 | 341 | 232 | 219 | CACHD1 | + | cache domain containing 1 | 0 | CACHD1 |
| LOC101111988 | 41.29908047 | 86.64402403 | -1.06899 | 0.02386756 | down | 98 | 85 | 73 | 27 | 30 | 64 | LOC101111988 | + | Uncharacterized protein | 9.00E-42 | ASIP |
| WDFY4 | 82.93070211 | 227.7624391 | -1.45755 | 0.02399407 | down | 262 | 258 | 149 | 102 | 124 | 32 | WDFY4 | + | WDFY family member 4 | 0 | WDFY4 |
| FTSJ3 | 287.3123412 | 129.7847076 | 1.1465 | 0.02399486 | up | 140 | 116 | 128 | 257 | 373 | 238 | FTSJ3 | - | pre-rRNA processing protein FTSJ3 | 0 | FTSJ3 |
| RPL36A | 1736.150786 | 1042.609314 | 0.735694 | 0.02428319 | up | 1137 | 664 | 1321 | 2271 | 1764 | 1346 | RPL36A | + | Uncharacterized protein | 4.00E-71 | RPL36A |
| PDGFRL | 140.7711252 | 235.9144493 | -0.74491 | 0.02428739 | down | 253 | 164 | 287 | 182 | 130 | 123 | PDGFRL | - | platelet-derived growth factor receptor-like | 0 | PDGFRL |
| GLO1 | 498.3501635 | 876.9826448 | -0.81539 | 0.02430738 | down | 826 | 699 | 1064 | 497 | 495 | 516 | GLO1 | - | Lactoylglutathione lyase | 7.00E-98 | GLO1 |
| FOXP1 | 382.0938421 | 233.3081894 | 0.71169 | 0.02450942 | up | 301 | 188 | 211 | 395 | 453 | 316 | FOXP1 | + | Uncharacterized protein | 0 | FOXP1 |
| LPIN3 | 113.4797281 | 63.08536872 | 0.847057 | 0.0247466 | up | 88 | 60 | 41 | 130 | 92 | 124 | LPIN3 | + | Uncharacterized protein | 0 | LPIN3 |
| PLCL1 | 87.90888377 | 209.3696733 | -1.25197 | 0.02515371 | down | 246 | 114 | 272 | 119 | 83 | 71 | PLCL1 | + | Phosphoinositide phospholipase C | 0 | PLCL1 |
| DRG2 | 598.1373582 | 396.1988462 | 0.594252 | 0.02524375 | up | 461 | 392 | 319 | 596 | 629 | 587 | DRG2 | - | Uncharacterized protein | 0 | DRG2 |
| CITED2 | 289.668839 | 155.8895215 | 0.893881 | 0.02542677 | up | 105 | 134 | 214 | 366 | 233 | 292 | CITED2 | - | Cbp/p300-interacting transactivator with Glu/Asp-rich carboxy-terminal domain 2 | | |
| NDE1 | 83.17680081 | 131.7003319 | -0.66301 | 0.02578912 | down | 164 | 116 | 113 | 113 | 56 | 88 | NDE1 | + | Uncharacterized protein | 1.00E-170 | NDE1 |
| CHRNE | 313.9368171 | 834.4670671 | -1.41038 | 0.02583509 | down | 877 | 993 | 563 | 267 | 291 | 380 | CHRNE | + | Uncharacterized protein | 0 | CHRNE |
| RHOBTB3 | 940.6464431 | 1771.08699 | -0.91291 | 0.0258507 | down | 1720 | 1305 | 2225 | 1096 | 935 | 852 | RHOBTB3 | + | Uncharacterized protein | 0 | RHOBTB3 |
| LOC101119498 | 87.44275879 | 263.6174479 | -1.59204 | 0.02607505 | down | 377 | 259 | 154 | 156 | 46 | 76 | LOC101119498 | - | uncharacterized LOC101119498 | 2.00E-69 | MAF |

| | | | | | | | | | | | | | | | | |
|---|---|---|---|---|---|---|---|---|---|---|---|---|---|---|---|---|
| SIGLEC1 | 7.739222679 | 23.2480218 | -1.58685 | 0.02613107 | down | 24 | 18 | 27 | 8 | 16 | 0 | SIGLEC1 | + | Uncharacterized protein | 0 | SIGLEC1 |
| ERBA | 171.2922113 | 317.5982755 | -0.89074 | 0.02664569 | down | 299 | 250 | 389 | 188 | 133 | 199 | ERBA | + | | | |
| NAA40 | 43.73851393 | 93.83622611 | -1.10124 | 0.02678063 | down | 77 | 93 | 103 | 29 | 44 | 56 | NAA40 | + | Uncharacterized protein | 1.00E-137 | NAA40 |
| LOC101107475 | 49.70251592 | 15.22827225 | 1.706567 | 0.02687263 | up | 14 | 19 | 11 | 55 | 71 | 27 | LOC101107475 | - | Ig alpha-1 chain C region-like | | |
| PCED1A | 101.2497315 | 66.07727109 | 0.615692 | 0.02687561 | up | 61 | 67 | 65 | 103 | 103 | 101 | PCED1A | + | PC-esterase domain containing 1A | 0 | PCED1A |
| CCDC130 | 131.0688149 | 73.07502695 | 0.842874 | 0.02689786 | up | 84 | 80 | 51 | 123 | 133 | 139 | CCDC130 | - | Uncharacterized protein | 0 | CCDC130 |
| PPP1R3B | 181.5400756 | 657.3736407 | -1.85643 | 0.0270473 | down | 863 | 397 | 732 | 490 | 53 | 70 | PPP1R3B | - | Uncharacterized protein | 1.00E-154 | PPP1R3B |
| LOC101114192 | 128.5925749 | 80.85799793 | 0.669345 | 0.02718991 | up | 99 | 64 | 79 | 122 | 133 | 133 | LOC101114192 | + | uncharacterized LOC101114192 | 3.00E-27 | C19orf24 |
| C11H17orf80 | 113.3236071 | 72.94596921 | 0.635548 | 0.02723211 | up | 66 | 73 | 74 | 140 | 126 | 84 | C11H17orf80 | - | chromosome 11 open reading frame, human C17orf80 | 1.00E-145 | C17orf80 |
| COL11A2 | 6.908069348 | 43.64821253 | -2.65957 | 0.02772726 | down | 82 | 17 | 38 | 7 | 8 | 6 | COL11A2 | - | Uncharacterized protein | 0 | COL11A2 |
| TCAIM | 30.89175952 | 79.18144833 | -1.35794 | 0.02803715 | down | 63 | 64 | 105 | 47 | 27 | 23 | TCAIM | + | Uncharacterized protein | 0 | TCAIM |
| C3H9orf114 | 116.6899164 | 76.28925955 | 0.613128 | 0.02810887 | up | 103 | 58 | 69 | 138 | 98 | 121 | C3H9orf114 | + | Uncharacterized protein | 4.00E-160 | C9orf114 |
| LOC101103243 | 103.9320848 | 23.59286268 | 2.139219 | 0.02812601 | up | 39 | 19 | 14 | 79 | 155 | 78 | LOC101103243 | - | uncharacterized LOC101103243 | | |
| KANK3 | 227.1506161 | 150.6827833 | 0.592135 | 0.02819158 | up | 177 | 164 | 103 | 257 | 219 | 218 | KANK3 | - | KN motif and ankyrin repeat domains 3 | 1.00E-180 | KANK3 |
| TRNAR-CCG | 3.797039021 | 14.72744342 | -1.95556 | 0.02836906 | down | 15 | 9 | 20 | 7 | 0 | 5 | TRNAR-CCG | + | transfer RNA arginine (anticodon CCG) | | |
| ANK2 | 804.1814038 | 1543.790054 | -0.94088 | 0.02859466 | down | 1782 | 1268 | 1549 | 1432 | 535 | 596 | ANK2 | - | Uncharacterized protein | 0 | ANK2 |
| CASP16 | 10.52508169 | 34.32927776 | -1.70561 | 0.0286596 | down | 55 | 24 | 26 | 18 | 1 | 14 | CASP16 | + | Uncharacterized protein | 3.00E-73 | xp_003403727 |
| GPT2 | 1993.4454 | 4285.902795 | -1.10434 | 0.02869046 | down | 4111 | 4801 | 3568 | 2562 | 1447 | 2123 | GPT2 | + | Uncharacterized protein | 0 | GPT2 |
| PRKCQ | 1528.81324 | 756.1949813 | 1.015582 | 0.02883199 | up | 759 | 603 | 877 | 1511 | 1293 | 1805 | PRKCQ | - | Protein kinase C | 0 | PRKCQ |
| KLHL33 | 255.1147504 | 709.462218 | -1.47558 | 0.02949802 | down | 513 | 690 | 853 | 279 | 146 | 346 | KLHL33 | + | kelch-like 33 (Drosophila) | 0 | KLHL33 |
| PCMT1 | 458.5493144 | 835.6270796 | -0.86578 | 0.02991334 | down | 744 | 710 | 1001 | 518 | 381 | 498 | PCMT1 | + | Protein-L-isoaspartate O-methyltransferase | 7.00E-117 | PCMT1 |
| REV3L | 843.0103598 | 290.0467078 | 1.539265 | 0.02995098 | up | 345 | 174 | 355 | 945 | 1142 | 507 | REV3L | + | DNA polymerase | 0 | REV3L |
| APIP | 399.2160341 | 178.3036018 | 1.162834 | 0.02996103 | up | 154 | 183 | 182 | 320 | 430 | 442 | APIP | - | Methylthioribulose-1-phosphate dehydratase | 4.00E-134 | APIP |
| GPSM2 | 31.84061657 | 88.99079075 | -1.48279 | 0.02997061 | down | 86 | 56 | 123 | 52 | 31 | 18 | GPSM2 | + | Uncharacterized protein | 0 | GPSM2 |
| HCLS1 | 76.09340894 | 121.2520595 | -0.67217 | 0.0299891 | down | 162 | 87 | 117 | 83 | 61 | 87 | HCLS1 | - | Uncharacterized protein | 0 | HCLS1 |
| CCNG2 | 124.1034688 | 74.15179952 | 0.74299 | 0.02999529 | up | 68 | 64 | 86 | 124 | 146 | 107 | CCNG2 | + | Uncharacterized protein | 2.00E-177 | CCNG2 |
| ME2 | 84.20242116 | 168.5529712 | -1.00127 | 0.03076315 | down | 218 | 104 | 188 | 136 | 70 | 60 | ME2 | + | Malic enzyme | 0 | ME2 |
| EBNA1BP2 | 180.2240662 | 85.08675864 | 1.082785 | 0.03144092 | up | 104 | 57 | 95 | 154 | 221 | 167 | EBNA1BP2 | - | Uncharacterized protein | 4.00E-120 | EBNA1BP2 |
| SLMAP | 696.1450272 | 449.3423881 | 0.631573 | 0.03167238 | up | 435 | 337 | 559 | 801 | 768 | 567 | SLMAP | - | Uncharacterized protein | 0 | SLMAP |
| AMOTL1 | 947.9953063 | 1647.020188 | -0.79691 | 0.03194727 | down | 2088 | 1398 | 1441 | 1449 | 599 | 916 | AMOTL1 | - | Uncharacterized protein | 0 | AMOTL1 |
| POPDC2 | 67.22068391 | 173.2479766 | -1.36586 | 0.03260907 | down | 204 | 155 | 156 | 152 | 17 | 51 | POPDC2 | - | Uncharacterized protein | 1.00E-179 | POPDC2 |

| | | | | | | | | | | | | | | | | |
|---|---|---|---|---|---|---|---|---|---|---|---|---|---|---|---|---|
| LRRC30 | 166.5258451 | 356.803263 | -1.09938 | 0.03343811 | down | 284 | 390 | 358 | 183 | 136 | 187 | LRRC30 | + | leucine rich repeat containing 30 | 3.00E-116 | LRRC30 |
| CCDC86 | 416.1718415 | 168.3254363 | 1.305926 | 0.03354934 | up | 218 | 167 | 116 | 330 | 516 | 400 | CCDC86 | + | coiled-coil domain containing 86 | 4.00E-122 | CCDC86 |
| FAM175A | 98.93358974 | 31.89056917 | 1.633331 | 0.03366872 | up | 39 | 21 | 36 | 74 | 136 | 86 | FAM175A | - | Uncharacterized protein | 0 | FAM175A |
| LOC101107556 | 53.74036503 | 162.2733581 | -1.59435 | 0.03385201 | down | 224 | 85 | 186 | 17 | 35 | 101 | LOC101107556 | + | uncharacterized LOC101107556 | 9.00E-100 | MKRN2OS |
| LOC101120934 | 9873.350111 | 1810.974005 | 2.446774 | 0.03405086 | up | 2248 | 1754 | 1380 | 6648 | 14017 | 8732 | LOC101120934 | - | glutamine synthetase-like | 6.00E-159 | GLUL |
| DAB2 | 182.173298 | 316.6880533 | -0.79775 | 0.03407739 | down | 425 | 206 | 328 | 262 | 151 | 155 | DAB2 | + | Uncharacterized protein | 0 | DAB2 |
| ECE2 | 143.7929761 | 45.21865453 | 1.669003 | 0.03472317 | up | 48 | 38 | 48 | 106 | 200 | 124 | ECE2 | - | Uncharacterized protein | 0 | ECE2 |
| METTL14 | 77.20822296 | 46.53492577 | 0.730441 | 0.03555502 | up | 50 | 39 | 49 | 102 | 86 | 52 | METTL14 | - | Uncharacterized protein | 0 | METTL14 |
| USP49 | 46.26410475 | 20.64179719 | 1.164325 | 0.03571248 | up | 18 | 22 | 20 | 75 | 35 | 36 | USP49 | - | Ubiquitin carboxyl-terminal hydrolase | 0 | USP49 |
| NAT9 | 79.22165934 | 43.26185179 | 0.872799 | 0.0358424 | up | 29 | 50 | 45 | 85 | 93 | 64 | NAT9 | + | Uncharacterized protein | 3.00E-101 | NAT9 |
| PPP3R1 | 1009.912295 | 1793.588443 | -0.82862 | 0.03590715 | down | 1549 | 1831 | 1842 | 1036 | 1057 | 973 | PPP3R1 | + | Uncharacterized protein | 2.00E-88 | PPP3R1 |
| TH | 3.89231959 | 14.724111 | -1.91948 | 0.03619934 | down | 24 | 11 | 10 | 4 | 7 | 1 | TH | + | Uncharacterized protein | 7.00E-76 | TH |
| LOC443090 | 1479.471576 | 515.5194552 | 1.520983 | 0.03658979 | up | 496 | 444 | 579 | 1747 | 1989 | 836 | LOC443090 | - | Glycerol-3-phoshate dehdrogenase | 0 | GPD2 |
| CREBRF | 137.5006382 | 56.32201281 | 1.287668 | 0.03666082 | up | 80 | 38 | 53 | 150 | 184 | 88 | CREBRF | + | Uncharacterized protein | 0 | CREBRF |
| LOC443301 | 2228.663276 | 1254.810875 | 0.828709 | 0.03678634 | up | 1313 | 1257 | 1119 | 2313 | 1878 | 2553 | LOC443301 | + | 14-3-3 protein | 7.00E-124 | YWHAH |
| LOC101113344 | 2826.367139 | 1843.027804 | 0.616871 | 0.03685682 | up | 1823 | 1539 | 2082 | 3021 | 2527 | 3034 | LOC101113344 | - | 60S ribosomal protein L13 | 9.00E-100 | RPL13 |
| ITIH5 | 316.4822224 | 610.8927756 | -0.94879 | 0.03727152 | down | 891 | 420 | 546 | 446 | 252 | 286 | ITIH5 | - | Uncharacterized protein | 0 | ITIH5 |
| FREM2 | 24.43403484 | 116.118976 | -2.24864 | 0.03766223 | down | 192 | 51 | 117 | 63 | 10 | 9 | FREM2 | - | Uncharacterized protein | 0 | FREM2 |
| MYL6B | 1654.633855 | 12018.29032 | -2.86065 | 0.03769217 | down | 15878 | 3921 | 17075 | 618 | 3161 | 1083 | MYL6B | - | myosin, light chain 6B, alkali, smooth muscle and non-muscle | 1.00E-89 | MYL6B |
| PQLC1 | 40.62722702 | 75.47546569 | -0.89356 | 0.03784144 | down | 66 | 76 | 78 | 53 | 27 | 45 | PQLC1 | + | PQ loop repeat containing 1 | 2.00E-135 | PQLC1 |
| BLVRB | 449.2771351 | 718.4194143 | -0.67722 | 0.03786356 | down | 930 | 654 | 562 | 465 | 391 | 504 | BLVRB | - | Uncharacterized protein | 9.00E-79 | BLVRB |
| OSBPL6 | 99.76438669 | 176.7000946 | -0.82471 | 0.03805243 | down | 221 | 121 | 190 | 128 | 59 | 119 | OSBPL6 | - | Oxysterol-binding protein | 0 | OSBPL6 |
| PPTC7 | 429.4324477 | 761.0685362 | -0.8256 | 0.03846031 | down | 932 | 654 | 685 | 700 | 259 | 393 | PPTC7 | - | Uncharacterized protein | 4.00E-173 | PPTC7 |
| PPP3CB | 1122.042201 | 1977.985914 | -0.81791 | 0.03849721 | down | 1773 | 1823 | 2198 | 1554 | 856 | 1070 | PPP3CB | - | Serine/threonine-protein phosphatase | 0 | PPP3CB |
| LOC101119893 | 143.8046082 | 74.34597491 | 0.951783 | 0.03857205 | up | 99 | 41 | 86 | 139 | 173 | 124 | LOC101119893 | - | Uncharacterized protein | 0 | VRK2 |
| KLF11 | 1014.826315 | 361.1453378 | 1.490581 | 0.03861923 | up | 191 | 394 | 446 | 761 | 1158 | 1103 | KLF11 | + | Uncharacterized protein | 0 | KLF11 |
| LOC100885761 | 27.75414358 | 50.17946468 | -0.85439 | 0.03864165 | down | 68 | 33 | 51 | 36 | 29 | 21 | LOC100885761 | - | Wee1 kinase | 0 | WEE1 |
| INSR | 764.8249693 | 444.1883564 | 0.783958 | 0.03908419 | up | 508 | 339 | 479 | 772 | 929 | 626 | INSR | - | Insulin receptor | 0 | INSR |
| LOC101107037 | 12.13528625 | 35.4765022 | -1.54766 | 0.03944308 | down | 34 | 42 | 27 | 16 | 2 | 19 | LOC101107037 | - | Uncharacterized protein | 2.00E-177 | PTPN11 |
| ATP2A2 | 11031.15487 | 20408.00091 | -0.88755 | 0.04023567 | down | 19825 | 16994 | 23418 | 13885 | 13340 | 6971 | ATP2A2 | + | Plasma membrane calcium ATPase | | |
| FBLIM1 | 163.238979 | 311.8106479 | -0.93368 | 0.04106581 | down | 262 | 279 | 371 | 208 | 123 | 171 | FBLIM1 | - | Uncharacterized protein | 5.00E-143 | FBLIM1 |
| NOL6 | 279.9361655 | 161.1669823 | 0.796542 | 0.04119265 | up | 179 | 156 | 141 | 256 | 331 | 258 | NOL6 | + | Uncharacterized protein | 0 | NOL6 |

| | | | | | | | | | | | | | | | | |
|---|---|---|---|---|---|---|---|---|---|---|---|---|---|---|---|---|
| NFATC1 | 138.4233515 | 226.9638472 | -0.71338 | 0.04123504 | down | 265 | 236 | 169 | 142 | 141 | 137 | NFATC1 | - | Uncharacterized protein | 0 | NFATC1 |
| DDX56 | 416.4641407 | 180.0069638 | 1.21014 | 0.04143185 | up | 223 | 166 | 147 | 366 | 546 | 345 | DDX56 | + | Uncharacterized protein | 0 | DDX56 |
| IL4R | 369.1360838 | 221.7751018 | 0.735055 | 0.04158798 | up | 292 | 188 | 185 | 510 | 385 | 256 | IL4R | + | Uncharacterized protein | 0 | IL4R |
| OGFR | 133.3547787 | 76.5296767 | 0.801178 | 0.04187305 | up | 73 | 69 | 83 | 123 | 129 | 149 | OGFR | - | Uncharacterized protein | 3.00E-176 | OGFR |
| LOC101117648 | 128.6360955 | 80.37678681 | 0.678445 | 0.04260789 | up | 57 | 84 | 91 | 142 | 137 | 114 | LOC101117648 | - | UPF0368 protein Cxorf26-like | 9.00E-98 | PBDC1 |
| FBN3 | 21.94111653 | 48.14792386 | -1.13384 | 0.04283115 | down | 52 | 39 | 52 | 29 | 5 | 33 | FBN3 | - | Uncharacterized protein | 0 | FBN3 |
| SLC31A1 | 71.95328005 | 131.1003665 | -0.86554 | 0.04309803 | down | 174 | 121 | 97 | 110 | 62 | 54 | SLC31A1 | - | Uncharacterized protein | 5.00E-91 | SLC31A1 |
| VPS37C | 138.8008102 | 287.4893862 | -1.05049 | 0.04357875 | down | 278 | 333 | 225 | 158 | 113 | 152 | VPS37C | - | Uncharacterized protein | 2.00E-88 | VPS37C |
| PLXND1 | 329.5293026 | 574.188482 | -0.80112 | 0.04418327 | down | 525 | 570 | 583 | 492 | 263 | 276 | PLXND1 | + | Uncharacterized protein | 0 | PLXND1 |
| LOC101102211 | 833.5468211 | 1255.320217 | -0.59072 | 0.04423108 | down | 1180 | 1073 | 1443 | 953 | 820 | 777 | LOC101102211 | + | electron-transfer-flavoprotein, alpha polypeptide pseudogene | | |
| SEMA4D | 642.9237161 | 1289.660104 | -1.00427 | 0.0446806 | down | 1176 | 1344 | 1240 | 536 | 524 | 854 | SEMA4D | - | Uncharacterized protein | 0 | SEMA4D |
| NTSR2 | 41.84663013 | 10.25037608 | 2.029435 | 0.0456643 | up | 9 | 17 | 3 | 44 | 23 | 59 | NTSR2 | - | Uncharacterized protein | 3.00E-152 | NTSR2 |
| DYNLL2 | 960.5707306 | 1919.769166 | -0.99897 | 0.04592753 | down | 1603 | 1866 | 2125 | 1243 | 669 | 1043 | DYNLL2 | + | Uncharacterized protein | 7.00E-49 | DYNLL2 |
| CSRP3 | 18425.39633 | 4896.283026 | 1.911937 | 0.04606541 | up | 3025 | 879 | 10726 | 16929 | 16708 | 21684 | CSRP3 | + | Uncharacterized protein | 1.00E-105 | CSRP3 |
| KAT5 | 329.2406527 | 218.998488 | 0.588222 | 0.04653321 | up | 281 | 198 | 175 | 331 | 361 | 307 | KAT5 | + | Histone acetyltransferase | 0 | KAT5 |
| DCTD | 115.5635843 | 46.25834655 | 1.320901 | 0.04678384 | up | 101 | 27 | 18 | 154 | 104 | 100 | DCTD | - | Uncharacterized protein | 2.00E-97 | DCTD |
| NTRK2 | 24.57277897 | 69.67721678 | -1.50363 | 0.04760877 | down | 106 | 36 | 72 | 46 | 19 | 14 | NTRK2 | - | Tyrosine-protein kinase receptor | 0 | NTRK2 |
| NUDT1 | 71.16417903 | 27.56830047 | 1.368141 | 0.04783309 | up | 27 | 26 | 28 | 50 | 79 | 82 | NUDT1 | - | Uncharacterized protein | 3.00E-80 | NUDT1 |
| LOC101110918 | 664.7189467 | 194.6919218 | 1.771551 | 0.04798638 | up | 207 | 156 | 215 | 483 | 922 | 581 | LOC101110918 | + | uncharacterized LOC101110918 | | |
| PTPN21 | 723.6985456 | 1130.673209 | -0.64372 | 0.04817136 | down | 1073 | 1121 | 1116 | 860 | 575 | 778 | PTPN21 | - | Protein-tyrosine-phosphatase | 0 | PTPN21 |
| LOC101120404 | 445.0648045 | 807.7077091 | -0.85982 | 0.04818131 | down | 958 | 526 | 944 | 539 | 542 | 294 | LOC101120404 | + | atlastin-2-like | 0 | ATL2 |
| PION | 8.150209228 | 21.65379361 | -1.40971 | 0.04818955 | down | 32 | 14 | 20 | 15 | 4 | 7 | PION | + | Uncharacterized protein | 0 | GSAP |
| GAS2L2 | 0.343146897 | 8.829716425 | -4.68547 | 0.04820846 | down | 21 | 6 | 1 | 0 | 1 | 0 | GAS2L2 | + | Uncharacterized protein | 0 | GAS2L2 |
| GABARAPL1 | 1598.550021 | 577.8455735 | 1.468008 | 0.0484231 | up | 678 | 482 | 562 | 1181 | 1983 | 1602 | GABARAPL1 | - | Uncharacterized protein | 7.00E-64 | GABARAPL1 |
| ARID5B | 4477.814012 | 449.4212476 | 3.316654 | 0.04857925 | up | 460 | 237 | 651 | 2070 | 6641 | 4420 | ARID5B | + | Uncharacterized protein | 0 | ARID5B |
| GTF3C3 | 88.82894567 | 50.98322596 | 0.801007 | 0.04923005 | up | 73 | 41 | 40 | 123 | 92 | 62 | GTF3C3 | - | Uncharacterized protein | 0 | GTF3C3 |
| LOC101121811 | 157.1213222 | 247.3502019 | -0.65468 | 0.04950804 | down | 342 | 187 | 218 | 158 | 144 | 173 | LOC101121811 | + | Uncharacterized protein | 2.00E-24 | COX8A |
| SLC33A1 | 77.18356178 | 46.85494424 | 0.720092 | 0.04973326 | up | 41 | 44 | 52 | 88 | 91 | 58 | SLC33A1 | + | Uncharacterized protein | 0 | SLC33A1 |
| IL13RA1 | 242.8365005 | 151.6939672 | 0.678822 | 0.0498748 | up | 190 | 102 | 165 | 242 | 277 | 218 | IL13RA1 | - | Interleukin 13 receptor alpha 1 | 0 | IL13RA1 |

## Table S2: GO-analysis of all DEGs (STH vs QHMM)

| Biological process category | | | | | | | | | |
|---|---|---|---|---|---|---|---|---|---|
| **GOID** | **GOTerm** | **DifGene** | **AllDifGene** | **GeneInGO** | **AllGene** | **P-Value** | **FDR** | **Enrichment** | **(-log2P)** |
| GO:0044255 | cellular lipid metabolic process | 24 | 834 | 164 | 16650 | 1.45E-05 | 0.027502 | 2.9215652 | 16.07802992 |
| GO:0044281 | small molecule metabolic process | 120 | 834 | 1537 | 16650 | 1.75E-05 | 0.027502 | 1.558675 | 15.80499925 |
| GO:0009653 | anatomical structure morphogenesis | 20 | 834 | 138 | 16650 | 7.91E-05 | 0.0640725 | 2.8933375 | 13.62550448 |
| GO:0051260 | protein homooligomerization | 24 | 834 | 188 | 16650 | 1.01E-04 | 0.0640725 | 2.5485994 | 13.27904568 |
| GO:0009267 | cellular response to starvation | 11 | 834 | 48 | 16650 | 1.02E-04 | 0.0640725 | 4.5750899 | 13.26290028 |
| GO:0045214 | sarcomere organization | 9 | 834 | 35 | 16650 | 2.05E-04 | 0.1038021 | 5.1336074 | 12.25400353 |
| GO:0005975 | carbohydrate metabolic process | 41 | 834 | 431 | 16650 | 2.53E-04 | 0.1038021 | 1.8991303 | 11.94659673 |
| GO:0006006 | glucose metabolic process | 18 | 834 | 129 | 16650 | 2.64E-04 | 0.1038021 | 2.7856784 | 11.88877035 |
| GO:0046627 | negative regulation of insulin receptor signaling pathway | 8 | 834 | 29 | 16650 | 3.06E-04 | 0.107226 | 5.5073183 | 11.67202612 |
| GO:0030049 | muscle filament sliding | 9 | 834 | 39 | 16650 | 4.07E-04 | 0.1281805 | 4.6070836 | 11.26250099 |
| GO:0006470 | protein dephosphorylation | 22 | 834 | 190 | 16650 | 6.30E-04 | 0.1804448 | 2.3116244 | 10.63161699 |
| GO:0014883 | transition between fast and slow fiber | 4 | 834 | 6 | 16650 | 8.70E-04 | 0.2156276 | 13.309353 | 10.16594883 |
| GO:0008652 | cellular amino acid biosynthetic process | 8 | 834 | 35 | 16650 | 8.90E-04 | 0.2156276 | 4.5632066 | 10.13362475 |
| GO:0060070 | canonical Wnt signaling pathway | 13 | 834 | 88 | 16650 | 0.001106572 | 0.2235456 | 2.9492315 | 9.819687348 |
| GO:0055114 | oxidation-reduction process | 62 | 834 | 790 | 16650 | 0.001109917 | 0.2235456 | 1.5667972 | 9.815332834 |
| GO:0060048 | cardiac muscle contraction | 9 | 834 | 46 | 16650 | 0.001139789 | 0.2235456 | 3.9060056 | 9.777016985 |
| GO:0035567 | non-canonical Wnt signaling pathway | 6 | 834 | 20 | 16650 | 0.00120682 | 0.2235456 | 5.9892086 | 9.69457405 |
| GO:0042593 | glucose homeostasis | 15 | 834 | 113 | 16650 | 0.00128088 | 0.2240829 | 2.6500923 | 9.608648683 |
| GO:0001944 | vasculature development | 8 | 834 | 39 | 16650 | 0.001627529 | 0.2697415 | 4.0951854 | 9.263101298 |
| GO:0006631 | fatty acid metabolic process | 18 | 834 | 155 | 16650 | 0.001787843 | 0.2715711 | 2.3184033 | 9.127564089 |
| GO:0007519 | skeletal muscle tissue development | 10 | 834 | 60 | 16650 | 0.001811049 | 0.2715711 | 3.3273381 | 9.108958942 |
| GO:0045444 | fat cell differentiation | 11 | 834 | 73 | 16650 | 0.002241236 | 0.3103756 | 3.0082783 | 8.801489834 |
| GO:0006099 | tricarboxylic acid cycle | 7 | 834 | 32 | 16650 | 0.002291162 | 0.3103756 | 4.3671313 | 8.769704683 |
| GO:0006094 | gluconeogenesis | 8 | 834 | 42 | 16650 | 0.002445764 | 0.3103756 | 3.8026721 | 8.675498796 |
| GO:0032868 | response to insulin | 11 | 834 | 74 | 16650 | 0.002464081 | 0.3103756 | 2.9676259 | 8.664734845 |
| GO:0006936 | muscle contraction | 14 | 834 | 111 | 16650 | 0.00278151 | 0.3368837 | 2.5179856 | 8.489915998 |

| | | | | | | | | | |
|---|---|---|---|---|---|---|---|---|---|
| GO:0003009 | skeletal muscle contraction | 6 | 834 | 25 | 16650 | 0.003142168 | 0.3470016 | 4.7913669 | 8.31402415 |
| GO:0008016 | regulation of heart contraction | 8 | 834 | 44 | 16650 | 0.003149137 | 0.3470016 | 3.6298234 | 8.310827892 |
| GO:0000466 | maturation of 5.8S rRNA from tricistronic rRNA transcript (SSU-rRNA, 5.8S rRNA, LSU-rRNA) | 3 | 834 | 4 | 16650 | 0.003305827 | 0.3470016 | 14.973022 | 8.240773261 |
| GO:0051100 | negative regulation of binding | 3 | 834 | 4 | 16650 | 0.003305827 | 0.3470016 | 14.973022 | 8.240773261 |
| GO:0046034 | ATP metabolic process | 7 | 834 | 35 | 16650 | 0.003544872 | 0.3488376 | 3.9928058 | 8.140050697 |
| GO:0002027 | regulation of heart rate | 7 | 834 | 35 | 16650 | 0.003544872 | 0.3488376 | 3.9928058 | 8.140050697 |
| GO:0005978 | glycogen biosynthetic process | 6 | 834 | 26 | 16650 | 0.003712011 | 0.3542158 | 4.6070836 | 8.07358338 |
| GO:0046676 | negative regulation of insulin secretion | 7 | 834 | 36 | 16650 | 0.004061063 | 0.3761261 | 3.8818945 | 7.943926734 |
| GO:0006768 | biotin metabolic process | 4 | 834 | 11 | 16650 | 0.004665504 | 0.408102 | 7.2596468 | 7.7437513 |
| GO:0071395 | cellular response to jasmonic acid stimulus | 4 | 834 | 11 | 16650 | 0.004665504 | 0.408102 | 7.2596468 | 7.7437513 |
| GO:0006090 | pyruvate metabolic process | 6 | 834 | 28 | 16650 | 0.005076334 | 0.4313915 | 4.2780062 | 7.621997312 |
| GO:0032869 | cellular response to insulin stimulus | 11 | 834 | 84 | 16650 | 0.005828273 | 0.4313915 | 2.6143371 | 7.422715726 |
| GO:0051149 | positive regulation of muscle cell differentiation | 6 | 834 | 29 | 16650 | 0.005881487 | 0.4313915 | 4.1304887 | 7.409603254 |
| GO:0048741 | skeletal muscle fiber development | 6 | 834 | 29 | 16650 | 0.005881487 | 0.4313915 | 4.1304887 | 7.409603254 |
| GO:0051412 | response to corticosterone | 6 | 834 | 29 | 16650 | 0.005881487 | 0.4313915 | 4.1304887 | 7.409603254 |
| GO:0008406 | gonad development | 6 | 834 | 30 | 16650 | 0.006775742 | 0.4313915 | 3.9928058 | 7.2054053 |
| GO:0040008 | regulation of growth | 10 | 834 | 74 | 16650 | 0.006874561 | 0.4313915 | 2.6978417 | 7.184516686 |
| GO:0035914 | skeletal muscle cell differentiation | 8 | 834 | 51 | 16650 | 0.006901635 | 0.4313915 | 3.1316124 | 7.178846058 |
| GO:0045663 | positive regulation of myoblast differentiation | 5 | 834 | 21 | 16650 | 0.007088716 | 0.4313915 | 4.7533402 | 7.140259904 |
| GO:0070542 | response to fatty acid | 5 | 834 | 21 | 16650 | 0.007088716 | 0.4313915 | 4.7533402 | 7.140259904 |
| GO:0008285 | negative regulation of cell proliferation | 35 | 834 | 433 | 16650 | 0.007306876 | 0.4313915 | 1.6137206 | 7.096529647 |
| GO:0048743 | positive regulation of skeletal muscle fiber development | 3 | 834 | 6 | 16650 | 0.007380301 | 0.4313915 | 9.9820144 | 7.082104607 |
| GO:0046498 | S-adenosylhomocysteine metabolic process | 3 | 834 | 6 | 16650 | 0.007380301 | 0.4313915 | 9.9820144 | 7.082104607 |
| GO:0042693 | muscle cell fate commitment | 3 | 834 | 6 | 16650 | 0.007380301 | 0.4313915 | 9.9820144 | 7.082104607 |
| GO:0001780 | neutrophil homeostasis | 3 | 834 | 6 | 16650 | 0.007380301 | 0.4313915 | 9.9820144 | 7.082104607 |
| GO:0006089 | lactate metabolic process | 3 | 834 | 6 | 16650 | 0.007380301 | 0.4313915 | 9.9820144 | 7.082104607 |
| GO:0097193 | intrinsic apoptotic signaling pathway | 10 | 834 | 75 | 16650 | 0.007462625 | 0.4313915 | 2.6618705 | 7.066101071 |
| GO:0055003 | cardiac myofibril assembly | 4 | 834 | 13 | 16650 | 0.007534624 | 0.4313915 | 6.1427781 | 7.052248674 |
| GO:0090179 | planar cell polarity pathway involved in neural tube closure | 4 | 834 | 13 | 16650 | 0.007534624 | 0.4313915 | 6.1427781 | 7.052248674 |

| | | | | | | | | | |
|---|---|---|---|---|---|---|---|---|---|
| GO:0001937 | negative regulation of endothelial cell proliferation | 6 | 834 | 31 | 16650 | 0.007764385 | 0.436608 | 3.8640056 | 7.00891268 |
| GO:0048545 | response to steroid hormone | 7 | 834 | 42 | 16650 | 0.008434865 | 0.457955 | 3.3273381 | 6.889419368 |
| GO:0006635 | fatty acid beta-oxidation | 7 | 834 | 42 | 16650 | 0.008434865 | 0.457955 | 3.3273381 | 6.889419368 |
| GO:1900740 | positive regulation of protein insertion into mitochondrial membrane involved in apoptotic signaling pathway | 6 | 834 | 32 | 16650 | 0.008852637 | 0.4646159 | 3.7432554 | 6.819677079 |
| GO:0006730 | one-carbon metabolic process | 6 | 834 | 32 | 16650 | 0.008852637 | 0.4646159 | 3.7432554 | 6.819677079 |
| GO:0016311 | dephosphorylation | 22 | 834 | 242 | 16650 | 0.009103103 | 0.4660647 | 1.8149117 | 6.779425815 |
| GO:0000463 | maturation of LSU-rRNA from tricistronic rRNA transcript (SSU-rRNA, 5.8S rRNA, LSU-rRNA) | 4 | 834 | 14 | 16650 | 0.009324254 | 0.4660647 | 5.7040082 | 6.744796048 |
| GO:0045780 | positive regulation of bone resorption | 4 | 834 | 14 | 16650 | 0.009324254 | 0.4660647 | 5.7040082 | 6.744796048 |
| GO:0030913 | paranodal junction assembly | 3 | 834 | 7 | 16650 | 0.010170237 | 0.4709717 | 8.5560123 | 6.619502927 |
| GO:0010694 | positive regulation of alkaline phosphatase activity | 3 | 834 | 7 | 16650 | 0.010170237 | 0.4709717 | 8.5560123 | 6.619502927 |
| GO:0014894 | response to denervation involved in regulation of muscle adaptation | 3 | 834 | 7 | 16650 | 0.010170237 | 0.4709717 | 8.5560123 | 6.619502927 |
| GO:0051170 | nuclear import | 3 | 834 | 7 | 16650 | 0.010170237 | 0.4709717 | 8.5560123 | 6.619502927 |
| GO:0043462 | regulation of ATPase activity | 3 | 834 | 7 | 16650 | 0.010170237 | 0.4709717 | 8.5560123 | 6.619502927 |
| GO:0044597 | daunorubicin metabolic process | 4 | 834 | 15 | 16650 | 0.011368866 | 0.4760465 | 5.323741 | 6.458767808 |
| GO:0044598 | doxorubicin metabolic process | 4 | 834 | 15 | 16650 | 0.011368866 | 0.4760465 | 5.323741 | 6.458767808 |
| GO:0016236 | macroautophagy | 8 | 834 | 57 | 16650 | 0.0122065 | 0.4760465 | 2.801969 | 6.35620661 |
| GO:0032259 | methylation | 19 | 834 | 206 | 16650 | 0.012708722 | 0.4760465 | 1.8413425 | 6.298037287 |
| GO:1903146 | regulation of mitochondrion degradation | 6 | 834 | 35 | 16650 | 0.012765818 | 0.4760465 | 3.4224049 | 6.291570241 |
| GO:0098734 | macromolecule depalmitoylation | 2 | 834 | 2 | 16650 | 0.012840427 | 0.4760465 | 19.964029 | 6.283163026 |
| GO:0006097 | glyoxylate cycle | 2 | 834 | 2 | 16650 | 0.012840427 | 0.4760465 | 19.964029 | 6.283163026 |
| GO:0031652 | positive regulation of heat generation | 2 | 834 | 2 | 16650 | 0.012840427 | 0.4760465 | 19.964029 | 6.283163026 |
| GO:0071954 | chemokine (C-C motif) ligand 11 production | 2 | 834 | 2 | 16650 | 0.012840427 | 0.4760465 | 19.964029 | 6.283163026 |
| GO:0007522 | visceral muscle development | 2 | 834 | 2 | 16650 | 0.012840427 | 0.4760465 | 19.964029 | 6.283163026 |
| GO:2000570 | positive regulation of T-helper 2 cell activation | 2 | 834 | 2 | 16650 | 0.012840427 | 0.4760465 | 19.964029 | 6.283163026 |
| GO:0019087 | transformation of host cell by virus | 2 | 834 | 2 | 16650 | 0.012840427 | 0.4760465 | 19.964029 | 6.283163026 |
| GO:0031444 | slow-twitch skeletal muscle fiber contraction | 2 | 834 | 2 | 16650 | 0.012840427 | 0.4760465 | 19.964029 | 6.283163026 |
| GO:0006656 | phosphatidylcholine biosynthetic process | 5 | 834 | 25 | 16650 | 0.013108209 | 0.4760465 | 3.9928058 | 6.253385655 |
| GO:0055010 | ventricular cardiac muscle tissue morphogenesis | 5 | 834 | 25 | 16650 | 0.013108209 | 0.4760465 | 3.9928058 | 6.253385655 |
| GO:0060412 | ventricular septum morphogenesis | 5 | 834 | 25 | 16650 | 0.013108209 | 0.4760465 | 3.9928058 | 6.253385655 |

| | | | | | | | | | |
|---|---|---|---|---|---|---|---|---|---|
| GO:0048102 | autophagic cell death | 3 | 834 | 8 | 16650 | 0.013490611 | 0.4760465 | 7.4865108 | 6.2119005 |
| GO:0002318 | myeloid progenitor cell differentiation | 3 | 834 | 8 | 16650 | 0.013490611 | 0.4760465 | 7.4865108 | 6.2119005 |
| GO:0060056 | mammary gland involution | 3 | 834 | 8 | 16650 | 0.013490611 | 0.4760465 | 7.4865108 | 6.2119005 |
| GO:0007420 | brain development | 22 | 834 | 252 | 16650 | 0.013545574 | 0.4760465 | 1.7428914 | 6.206034683 |
| GO:1901741 | positive regulation of myoblast fusion | 4 | 834 | 16 | 16650 | 0.013680466 | 0.4760465 | 4.9910072 | 6.191738805 |
| GO:0007405 | neuroblast proliferation | 4 | 834 | 16 | 16650 | 0.013680466 | 0.4760465 | 4.9910072 | 6.191738805 |
| GO:0007050 | cell cycle arrest | 15 | 834 | 151 | 16650 | 0.014192031 | 0.4760465 | 1.9831817 | 6.138775084 |
| GO:0043433 | negative regulation of sequence-specific DNA binding transcription factor activity | 9 | 834 | 71 | 16650 | 0.014261829 | 0.4760465 | 2.5306515 | 6.131697152 |
| GO:0030308 | negative regulation of cell growth | 13 | 834 | 124 | 16650 | 0.014778677 | 0.4760465 | 2.093003 | 6.080339042 |
| GO:0008152 | metabolic process | 129 | 834 | 2068 | 16650 | 0.015372461 | 0.4760465 | 1.2453384 | 6.023508017 |
| GO:0035690 | cellular response to drug | 8 | 834 | 60 | 16650 | 0.015766299 | 0.4760465 | 2.6618705 | 5.987012184 |
| GO:0005977 | glycogen metabolic process | 6 | 834 | 37 | 16650 | 0.015963438 | 0.4760465 | 3.2374101 | 5.969084754 |
| GO:0019674 | NAD metabolic process | 4 | 834 | 17 | 16650 | 0.016269651 | 0.4760465 | 4.6974185 | 5.941672917 |
| GO:0070848 | response to growth factor | 4 | 834 | 17 | 16650 | 0.016269651 | 0.4760465 | 4.6974185 | 5.941672917 |
| GO:0045944 | positive regulation of transcription from RNA polymerase II promoter | 66 | 834 | 978 | 16650 | 0.016771994 | 0.4760465 | 1.3472657 | 5.897801962 |
| GO:0051289 | protein homotetramerization | 8 | 834 | 61 | 16650 | 0.017103987 | 0.4760465 | 2.6182333 | 5.869523488 |
| GO:0071340 | skeletal muscle acetylcholine-gated channel clustering | 3 | 834 | 9 | 16650 | 0.01735447 | 0.4760465 | 6.6546763 | 5.848548862 |
| GO:0033173 | calcineurin-NFAT signaling cascade | 3 | 834 | 9 | 16650 | 0.01735447 | 0.4760465 | 6.6546763 | 5.848548862 |
| GO:2001259 | positive regulation of cation channel activity | 3 | 834 | 9 | 16650 | 0.01735447 | 0.4760465 | 6.6546763 | 5.848548862 |
| GO:0006405 | RNA export from nucleus | 3 | 834 | 9 | 16650 | 0.01735447 | 0.4760465 | 6.6546763 | 5.848548862 |
| GO:0006195 | purine nucleotide catabolic process | 3 | 834 | 9 | 16650 | 0.01735447 | 0.4760465 | 6.6546763 | 5.848548862 |
| GO:0006767 | water-soluble vitamin metabolic process | 10 | 834 | 87 | 16650 | 0.017928102 | 0.4760465 | 2.294716 | 5.801633429 |
| GO:0007507 | heart development | 19 | 834 | 215 | 16650 | 0.018435383 | 0.4760465 | 1.764263 | 5.761378772 |
| GO:0030178 | negative regulation of Wnt signaling pathway | 7 | 834 | 50 | 16650 | 0.018658473 | 0.4760465 | 2.794964 | 5.744025307 |
| GO:0044237 | cellular metabolic process | 17 | 834 | 186 | 16650 | 0.018925356 | 0.4760465 | 1.8246693 | 5.723535745 |
| GO:0051897 | positive regulation of protein kinase B signaling | 10 | 834 | 88 | 16650 | 0.01913124 | 0.4760465 | 2.2686396 | 5.707925771 |
| GO:0055117 | regulation of cardiac muscle contraction | 4 | 834 | 18 | 16650 | 0.019145594 | 0.4760465 | 4.4364508 | 5.706843779 |
| GO:0033365 | protein localization to organelle | 4 | 834 | 18 | 16650 | 0.019145594 | 0.4760465 | 4.4364508 | 5.706843779 |
| GO:0055007 | cardiac muscle cell differentiation | 5 | 834 | 28 | 16650 | 0.019399128 | 0.4760465 | 3.5650051 | 5.687864421 |

| | | | | | | | | | |
|---|---|---|---|---|---|---|---|---|---|
| GO:0010508 | positive regulation of autophagy | 5 | 834 | 28 | 16650 | 0.019399128 | 0.4760465 | 3.5650051 | 5.687864421 |
| GO:0042692 | muscle cell differentiation | 6 | 834 | 39 | 16650 | 0.019674276 | 0.4760465 | 3.071389 | 5.667545632 |
| GO:0001889 | liver development | 11 | 834 | 102 | 16650 | 0.019915735 | 0.4760465 | 2.1529835 | 5.649947473 |
| GO:0007517 | muscle organ development | 11 | 834 | 102 | 16650 | 0.019915735 | 0.4760465 | 2.1529835 | 5.649947473 |
| GO:0019432 | triglyceride biosynthetic process | 7 | 834 | 51 | 16650 | 0.020365323 | 0.4760465 | 2.7401608 | 5.6177415 |
| GO:0003007 | heart morphogenesis | 7 | 834 | 51 | 16650 | 0.020365323 | 0.4760465 | 2.7401608 | 5.6177415 |
| GO:0071400 | cellular response to oleic acid | 2 | 834 | 3 | 16650 | 0.020723529 | 0.4760465 | 13.309353 | 5.592586479 |
| GO:0006542 | glutamine biosynthetic process | 2 | 834 | 3 | 16650 | 0.020723529 | 0.4760465 | 13.309353 | 5.592586479 |
| GO:0061056 | sclerotome development | 2 | 834 | 3 | 16650 | 0.020723529 | 0.4760465 | 13.309353 | 5.592586479 |
| GO:0014733 | regulation of skeletal muscle adaptation | 2 | 834 | 3 | 16650 | 0.020723529 | 0.4760465 | 13.309353 | 5.592586479 |
| GO:0006982 | response to lipid hydroperoxide | 2 | 834 | 3 | 16650 | 0.020723529 | 0.4760465 | 13.309353 | 5.592586479 |
| GO:0045626 | negative regulation of T-helper 1 cell differentiation | 2 | 834 | 3 | 16650 | 0.020723529 | 0.4760465 | 13.309353 | 5.592586479 |
| GO:0048133 | male germ-line stem cell asymmetric division | 2 | 834 | 3 | 16650 | 0.020723529 | 0.4760465 | 13.309353 | 5.592586479 |
| GO:0045726 | positive regulation of integrin biosynthetic process | 2 | 834 | 3 | 16650 | 0.020723529 | 0.4760465 | 13.309353 | 5.592586479 |
| GO:0071453 | cellular response to oxygen levels | 2 | 834 | 3 | 16650 | 0.020723529 | 0.4760465 | 13.309353 | 5.592586479 |
| GO:1990166 | protein localization to site of double-strand break | 2 | 834 | 3 | 16650 | 0.020723529 | 0.4760465 | 13.309353 | 5.592586479 |
| GO:0034628 | 'de novo' NAD biosynthetic process from aspartate | 2 | 834 | 3 | 16650 | 0.020723529 | 0.4760465 | 13.309353 | 5.592586479 |
| GO:2000041 | negative regulation of planar cell polarity pathway involved in axis elongation | 2 | 834 | 3 | 16650 | 0.020723529 | 0.4760465 | 13.309353 | 5.592586479 |
| GO:0060420 | regulation of heart growth | 2 | 834 | 3 | 16650 | 0.020723529 | 0.4760465 | 13.309353 | 5.592586479 |
| GO:0086070 | SA node cell to atrial cardiac muscle cell communication | 2 | 834 | 3 | 16650 | 0.020723529 | 0.4760465 | 13.309353 | 5.592586479 |
| GO:0003179 | heart valve morphogenesis | 2 | 834 | 3 | 16650 | 0.020723529 | 0.4760465 | 13.309353 | 5.592586479 |
| GO:0071279 | cellular response to cobalt ion | 2 | 834 | 3 | 16650 | 0.020723529 | 0.4760465 | 13.309353 | 5.592586479 |
| GO:0032929 | negative regulation of superoxide anion generation | 2 | 834 | 3 | 16650 | 0.020723529 | 0.4760465 | 13.309353 | 5.592586479 |
| GO:0042262 | DNA protection | 2 | 834 | 3 | 16650 | 0.020723529 | 0.4760465 | 13.309353 | 5.592586479 |
| GO:0015701 | bicarbonate transport | 6 | 834 | 40 | 16650 | 0.021732456 | 0.4760465 | 2.9946043 | 5.524004964 |
| GO:0055013 | cardiac muscle cell development | 3 | 834 | 10 | 16650 | 0.021769035 | 0.4760465 | 5.9892086 | 5.521578739 |
| GO:0031017 | exocrine pancreas development | 3 | 834 | 10 | 16650 | 0.021769035 | 0.4760465 | 5.9892086 | 5.521578739 |
| GO:1903214 | regulation of protein targeting to mitochondrion | 3 | 834 | 10 | 16650 | 0.021769035 | 0.4760465 | 5.9892086 | 5.521578739 |
| GO:2000643 | positive regulation of early endosome to late endosome transport | 3 | 834 | 10 | 16650 | 0.021769035 | 0.4760465 | 5.9892086 | 5.521578739 |

| | | | | | | | | | |
|---|---|---|---|---|---|---|---|---|---|
| GO:0035413 | positive regulation of catenin import into nucleus | 3 | 834 | 10 | 16650 | 0.021769035 | 0.4760465 | 5.9892086 | 5.521578739 |
| GO:0009629 | response to gravity | 3 | 834 | 10 | 16650 | 0.021769035 | 0.4760465 | 5.9892086 | 5.521578739 |
| GO:0046928 | regulation of neurotransmitter secretion | 4 | 834 | 19 | 16650 | 0.022316046 | 0.4780492 | 4.2029534 | 5.485774785 |
| GO:0002026 | regulation of the force of heart contraction | 4 | 834 | 19 | 16650 | 0.022316046 | 0.4780492 | 4.2029534 | 5.485774785 |
| GO:0031668 | cellular response to extracellular stimulus | 4 | 834 | 19 | 16650 | 0.022316046 | 0.4780492 | 4.2029534 | 5.485774785 |
| GO:0051216 | cartilage development | 9 | 834 | 78 | 16650 | 0.023468224 | 0.4958693 | 2.3035418 | 5.41314753 |
| GO:0051924 | regulation of calcium ion transport | 5 | 834 | 30 | 16650 | 0.024533089 | 0.4958693 | 3.3273381 | 5.349127277 |
| GO:0006941 | striated muscle contraction | 4 | 834 | 20 | 16650 | 0.025787344 | 0.4958693 | 3.9928058 | 5.277192979 |
| GO:0045445 | myoblast differentiation | 4 | 834 | 20 | 16650 | 0.025787344 | 0.4958693 | 3.9928058 | 5.277192979 |
| GO:0002028 | regulation of sodium ion transport | 4 | 834 | 20 | 16650 | 0.025787344 | 0.4958693 | 3.9928058 | 5.277192979 |
| GO:0006807 | nitrogen compound metabolic process | 4 | 834 | 20 | 16650 | 0.025787344 | 0.4958693 | 3.9928058 | 5.277192979 |
| GO:0003151 | outflow tract morphogenesis | 6 | 834 | 42 | 16650 | 0.026272962 | 0.4958693 | 2.8520041 | 5.250277305 |
| GO:0015909 | long-chain fatty acid transport | 3 | 834 | 11 | 16650 | 0.026736389 | 0.4958693 | 5.4447351 | 5.22505155 |
| GO:0034383 | low-density lipoprotein particle clearance | 3 | 834 | 11 | 16650 | 0.026736389 | 0.4958693 | 5.4447351 | 5.22505155 |
| GO:0060416 | response to growth hormone | 3 | 834 | 11 | 16650 | 0.026736389 | 0.4958693 | 5.4447351 | 5.22505155 |
| GO:0002639 | positive regulation of immunoglobulin production | 3 | 834 | 11 | 16650 | 0.026736389 | 0.4958693 | 5.4447351 | 5.22505155 |
| GO:0006629 | lipid metabolic process | 38 | 834 | 530 | 16650 | 0.026824273 | 0.4958693 | 1.4313832 | 5.220317107 |
| GO:0009725 | response to hormone | 8 | 834 | 67 | 16650 | 0.026879168 | 0.4958693 | 2.3837646 | 5.21736771 |
| GO:0010718 | positive regulation of epithelial to mesenchymal transition | 5 | 834 | 31 | 16650 | 0.027397177 | 0.4958693 | 3.2200046 | 5.189828918 |
| GO:0001649 | osteoblast differentiation | 11 | 834 | 108 | 16650 | 0.027916742 | 0.4958693 | 2.0333733 | 5.162725628 |
| GO:0006766 | vitamin metabolic process | 10 | 834 | 95 | 16650 | 0.02925646 | 0.4958693 | 2.1014767 | 5.095100997 |
| GO:0006103 | 2-oxoglutarate metabolic process | 4 | 834 | 21 | 16650 | 0.029564442 | 0.4958693 | 3.8026721 | 5.079993145 |
| GO:0071398 | cellular response to fatty acid | 4 | 834 | 21 | 16650 | 0.029564442 | 0.4958693 | 3.8026721 | 5.079993145 |
| GO:0006597 | spermine biosynthetic process | 2 | 834 | 4 | 16650 | 0.030105841 | 0.4958693 | 9.9820144 | 5.053812787 |
| GO:0002175 | protein localization to paranode region of axon | 2 | 834 | 4 | 16650 | 0.030105841 | 0.4958693 | 9.9820144 | 5.053812787 |
| GO:2000035 | regulation of stem cell division | 2 | 834 | 4 | 16650 | 0.030105841 | 0.4958693 | 9.9820144 | 5.053812787 |
| GO:0070814 | hydrogen sulfide biosynthetic process | 2 | 834 | 4 | 16650 | 0.030105841 | 0.4958693 | 9.9820144 | 5.053812787 |
| GO:0010002 | cardioblast differentiation | 2 | 834 | 4 | 16650 | 0.030105841 | 0.4958693 | 9.9820144 | 5.053812787 |
| GO:1901203 | positive regulation of extracellular matrix assembly | 2 | 834 | 4 | 16650 | 0.030105841 | 0.4958693 | 9.9820144 | 5.053812787 |

| | | | | | | | | | |
|---|---|---|---|---|---|---|---|---|---|
| GO:0033076 | isoquinoline alkaloid metabolic process | 2 | 834 | 4 | 16650 | 0.030105841 | 0.4958693 | 9.9820144 | 5.053812787 |
| GO:0086015 | SA node cell action potential | 2 | 834 | 4 | 16650 | 0.030105841 | 0.4958693 | 9.9820144 | 5.053812787 |
| GO:0002503 | peptide antigen assembly with MHC class II protein complex | 2 | 834 | 4 | 16650 | 0.030105841 | 0.4958693 | 9.9820144 | 5.053812787 |
| GO:0010693 | negative regulation of alkaline phosphatase activity | 2 | 834 | 4 | 16650 | 0.030105841 | 0.4958693 | 9.9820144 | 5.053812787 |
| GO:0060931 | sinoatrial node cell development | 2 | 834 | 4 | 16650 | 0.030105841 | 0.4958693 | 9.9820144 | 5.053812787 |
| GO:0003062 | regulation of heart rate by chemical signal | 2 | 834 | 4 | 16650 | 0.030105841 | 0.4958693 | 9.9820144 | 5.053812787 |
| GO:0006880 | intracellular sequestering of iron ion | 2 | 834 | 4 | 16650 | 0.030105841 | 0.4958693 | 9.9820144 | 5.053812787 |
| GO:0090175 | regulation of establishment of planar polarity | 2 | 834 | 4 | 16650 | 0.030105841 | 0.4958693 | 9.9820144 | 5.053812787 |
| GO:0032909 | regulation of transforming growth factor beta2 production | 2 | 834 | 4 | 16650 | 0.030105841 | 0.4958693 | 9.9820144 | 5.053812787 |
| GO:0015721 | bile acid and bile salt transport | 5 | 834 | 32 | 16650 | 0.030464801 | 0.4958693 | 3.1193795 | 5.036712856 |
| GO:0034641 | cellular nitrogen compound metabolic process | 17 | 834 | 198 | 16650 | 0.030689647 | 0.4958693 | 1.7140833 | 5.026104141 |
| GO:0045725 | positive regulation of glycogen biosynthetic process | 3 | 834 | 12 | 16650 | 0.03225411 | 0.4958693 | 4.9910072 | 4.954373161 |
| GO:0033235 | positive regulation of protein sumoylation | 3 | 834 | 12 | 16650 | 0.03225411 | 0.4958693 | 4.9910072 | 4.954373161 |
| GO:0034375 | high-density lipoprotein particle remodeling | 3 | 834 | 12 | 16650 | 0.03225411 | 0.4958693 | 4.9910072 | 4.954373161 |
| GO:2001275 | positive regulation of glucose import in response to insulin stimulus | 3 | 834 | 12 | 16650 | 0.03225411 | 0.4958693 | 4.9910072 | 4.954373161 |
| GO:0061053 | somite development | 3 | 834 | 12 | 16650 | 0.03225411 | 0.4958693 | 4.9910072 | 4.954373161 |
| GO:0010510 | regulation of acetyl-CoA biosynthetic process from pyruvate | 3 | 834 | 12 | 16650 | 0.03225411 | 0.4958693 | 4.9910072 | 4.954373161 |
| GO:2000573 | positive regulation of DNA biosynthetic process | 3 | 834 | 12 | 16650 | 0.03225411 | 0.4958693 | 4.9910072 | 4.954373161 |
| GO:0010039 | response to iron ion | 4 | 834 | 22 | 16650 | 0.033650938 | 0.4958693 | 3.6298234 | 4.893209491 |
| GO:0010881 | regulation of cardiac muscle contraction by regulation of the release of sequestered calcium ion | 4 | 834 | 22 | 16650 | 0.033650938 | 0.4958693 | 3.6298234 | 4.893209491 |
| GO:0030511 | positive regulation of transforming growth factor beta receptor signaling pathway | 4 | 834 | 22 | 16650 | 0.033650938 | 0.4958693 | 3.6298234 | 4.893209491 |
| GO:0006778 | porphyrin-containing compound metabolic process | 4 | 834 | 22 | 16650 | 0.033650938 | 0.4958693 | 3.6298234 | 4.893209491 |
| GO:0050771 | negative regulation of axonogenesis | 4 | 834 | 22 | 16650 | 0.033650938 | 0.4958693 | 3.6298234 | 4.893209491 |
| GO:0006096 | glycolytic process | 6 | 834 | 45 | 16650 | 0.034192448 | 0.4958693 | 2.6618705 | 4.870178479 |
| GO:0001666 | response to hypoxia | 16 | 834 | 186 | 16650 | 0.034669943 | 0.4958693 | 1.7173358 | 4.850170734 |
| GO:0006091 | generation of precursor metabolites and energy | 7 | 834 | 58 | 16650 | 0.035415372 | 0.4958693 | 2.4094517 | 4.819480492 |
| GO:0016055 | Wnt signaling pathway | 17 | 834 | 202 | 16650 | 0.035611376 | 0.4958693 | 1.680141 | 4.811518026 |
| GO:0051726 | regulation of cell cycle | 14 | 834 | 157 | 16650 | 0.036290042 | 0.4958693 | 1.7802319 | 4.784282482 |
| GO:0035872 | nucleotide-binding domain, leucine rich repeat containing receptor signaling pathway | 6 | 834 | 46 | 16650 | 0.037138962 | 0.4958693 | 2.6040038 | 4.750922705 |

| | | | | | | | | | |
|---|---|---|---|---|---|---|---|---|---|
| GO:0090090 | negative regulation of canonical Wnt signaling pathway | 14 | 834 | 158 | 16650 | 0.037823342 | 0.4958693 | 1.7689646 | 4.724579357 |
| GO:0032526 | response to retinoic acid | 7 | 834 | 59 | 16650 | 0.038036857 | 0.4958693 | 2.3686136 | 4.71645814 |
| GO:0006783 | heme biosynthetic process | 4 | 834 | 23 | 16650 | 0.038049121 | 0.4958693 | 3.472005 | 4.715993081 |
| GO:0042326 | negative regulation of phosphorylation | 4 | 834 | 23 | 16650 | 0.038049121 | 0.4958693 | 3.472005 | 4.715993081 |
| GO:0010867 | positive regulation of triglyceride biosynthetic process | 3 | 834 | 13 | 16650 | 0.038315843 | 0.4958693 | 4.6070836 | 4.705915156 |
| GO:0051593 | response to folic acid | 3 | 834 | 13 | 16650 | 0.038315843 | 0.4958693 | 4.6070836 | 4.705915156 |
| GO:0042993 | positive regulation of transcription factor import into nucleus | 3 | 834 | 13 | 16650 | 0.038315843 | 0.4958693 | 4.6070836 | 4.705915156 |
| GO:0051168 | nuclear export | 3 | 834 | 13 | 16650 | 0.038315843 | 0.4958693 | 4.6070836 | 4.705915156 |
| GO:0046685 | response to arsenic-containing substance | 3 | 834 | 13 | 16650 | 0.038315843 | 0.4958693 | 4.6070836 | 4.705915156 |
| GO:0043434 | response to peptide hormone | 8 | 834 | 73 | 16650 | 0.039971756 | 0.4958693 | 2.1878388 | 4.64487525 |
| GO:0060333 | interferon-gamma-mediated signaling pathway | 8 | 834 | 73 | 16650 | 0.039971756 | 0.4958693 | 2.1878388 | 4.64487525 |
| GO:0006664 | glycolipid metabolic process | 2 | 834 | 5 | 16650 | 0.040825846 | 0.4958693 | 7.9856115 | 4.614373397 |
| GO:0019626 | short-chain fatty acid catabolic process | 2 | 834 | 5 | 16650 | 0.040825846 | 0.4958693 | 7.9856115 | 4.614373397 |
| GO:0080009 | mRNA methylation | 2 | 834 | 5 | 16650 | 0.040825846 | 0.4958693 | 7.9856115 | 4.614373397 |
| GO:0038031 | non-canonical Wnt signaling pathway via JNK cascade | 2 | 834 | 5 | 16650 | 0.040825846 | 0.4958693 | 7.9856115 | 4.614373397 |
| GO:0044259 | multicellular organismal macromolecule metabolic process | 2 | 834 | 5 | 16650 | 0.040825846 | 0.4958693 | 7.9856115 | 4.614373397 |
| GO:0055129 | L-proline biosynthetic process | 2 | 834 | 5 | 16650 | 0.040825846 | 0.4958693 | 7.9856115 | 4.614373397 |
| GO:0006538 | glutamate catabolic process | 2 | 834 | 5 | 16650 | 0.040825846 | 0.4958693 | 7.9856115 | 4.614373397 |
| GO:0018916 | nitrobenzene metabolic process | 2 | 834 | 5 | 16650 | 0.040825846 | 0.4958693 | 7.9856115 | 4.614373397 |
| GO:0060236 | regulation of mitotic spindle organization | 2 | 834 | 5 | 16650 | 0.040825846 | 0.4958693 | 7.9856115 | 4.614373397 |
| GO:0010815 | bradykinin catabolic process | 2 | 834 | 5 | 16650 | 0.040825846 | 0.4958693 | 7.9856115 | 4.614373397 |
| GO:0033157 | regulation of intracellular protein transport | 2 | 834 | 5 | 16650 | 0.040825846 | 0.4958693 | 7.9856115 | 4.614373397 |
| GO:0036155 | acylglycerol acyl-chain remodeling | 2 | 834 | 5 | 16650 | 0.040825846 | 0.4958693 | 7.9856115 | 4.614373397 |
| GO:2001257 | regulation of cation channel activity | 2 | 834 | 5 | 16650 | 0.040825846 | 0.4958693 | 7.9856115 | 4.614373397 |
| GO:0032446 | protein modification by small protein conjugation | 2 | 834 | 5 | 16650 | 0.040825846 | 0.4958693 | 7.9856115 | 4.614373397 |
| GO:0033014 | tetrapyrrole biosynthetic process | 2 | 834 | 5 | 16650 | 0.040825846 | 0.4958693 | 7.9856115 | 4.614373397 |
| GO:0045542 | positive regulation of cholesterol biosynthetic process | 2 | 834 | 5 | 16650 | 0.040825846 | 0.4958693 | 7.9856115 | 4.614373397 |
| GO:0010882 | regulation of cardiac muscle contraction by calcium ion signaling | 2 | 834 | 5 | 16650 | 0.040825846 | 0.4958693 | 7.9856115 | 4.614373397 |
| GO:0035279 | mRNA cleavage involved in gene silencing by miRNA | 2 | 834 | 5 | 16650 | 0.040825846 | 0.4958693 | 7.9856115 | 4.614373397 |

| | | | | | | | | | |
|---|---|---|---|---|---|---|---|---|---|
| GO:0006102 | isocitrate metabolic process | 2 | 834 | 5 | 16650 | 0.040825846 | 0.4958693 | 7.9856115 | 4.614373397 |
| GO:0060666 | dichotomous subdivision of terminal units involved in salivary gland branching | 2 | 834 | 5 | 16650 | 0.040825846 | 0.4958693 | 7.9856115 | 4.614373397 |
| GO:0070458 | cellular detoxification of nitrogen compound | 2 | 834 | 5 | 16650 | 0.040825846 | 0.4958693 | 7.9856115 | 4.614373397 |
| GO:0033601 | positive regulation of mammary gland epithelial cell proliferation | 2 | 834 | 5 | 16650 | 0.040825846 | 0.4958693 | 7.9856115 | 4.614373397 |
| GO:0061157 | mRNA destabilization | 2 | 834 | 5 | 16650 | 0.040825846 | 0.4958693 | 7.9856115 | 4.614373397 |
| GO:0060028 | convergent extension involved in axis elongation | 2 | 834 | 5 | 16650 | 0.040825846 | 0.4958693 | 7.9856115 | 4.614373397 |
| GO:0060029 | convergent extension involved in organogenesis | 2 | 834 | 5 | 16650 | 0.040825846 | 0.4958693 | 7.9856115 | 4.614373397 |
| GO:0071205 | protein localization to juxtaparanode region of axon | 2 | 834 | 5 | 16650 | 0.040825846 | 0.495869 | 7.985612 | 4.614373397 |
| GO:0051823 | regulation of synapse structural plasticity | 2 | 834 | 5 | 16650 | 0.040825846 | 0.495869 | 7.985612 | 4.614373397 |
| GO:0071392 | cellular response to estradiol stimulus | 4 | 834 | 24 | 16650 | 0.04276002 | 0.495869 | 3.327338 | 4.547593666 |
| GO:0032312 | regulation of ARF GTPase activity | 4 | 834 | 24 | 16650 | 0.04276002 | 0.495869 | 3.327338 | 4.547593666 |
| GO:0048041 | focal adhesion assembly | 4 | 834 | 24 | 16650 | 0.04276002 | 0.495869 | 3.327338 | 4.547593666 |
| GO:0033197 | response to vitamin E | 3 | 834 | 14 | 16650 | 0.044911818 | 0.495869 | 4.278006 | 4.476761068 |
| GO:0060766 | negative regulation of androgen receptor signaling pathway | 3 | 834 | 14 | 16650 | 0.044911818 | 0.495869 | 4.278006 | 4.476761068 |
| GO:0086004 | regulation of cardiac muscle cell contraction | 3 | 834 | 14 | 16650 | 0.044911818 | 0.495869 | 4.278006 | 4.476761068 |
| GO:0045926 | negative regulation of growth | 3 | 834 | 14 | 16650 | 0.044911818 | 0.495869 | 4.278006 | 4.476761068 |
| GO:0043568 | positive regulation of insulin-like growth factor receptor signaling pathway | 3 | 834 | 14 | 16650 | 0.044911818 | 0.495869 | 4.278006 | 4.476761068 |
| GO:0030239 | myofibril assembly | 3 | 834 | 14 | 16650 | 0.044911818 | 0.495869 | 4.278006 | 4.476761068 |
| GO:0001558 | regulation of cell growth | 8 | 834 | 75 | 16650 | 0.04513733 | 0.495869 | 2.129496 | 4.469535111 |
| GO:0007520 | myoblast fusion | 4 | 834 | 25 | 16650 | 0.047783458 | 0.495869 | 3.194245 | 4.387344914 |
| GO:0071377 | cellular response to glucagon stimulus | 5 | 834 | 37 | 16650 | 0.048955952 | 0.495869 | 2.697842 | 4.352371913 |
| GO:0035335 | peptidyl-tyrosine dephosphorylation | 10 | 834 | 105 | 16650 | 0.049542189 | 0.495869 | 1.901336 | 4.335198562 |
| GO:0038095 | Fc-epsilon receptor signaling pathway | 23 | 834 | 308 | 16650 | 0.049794359 | 0.495869 | 1.49082 | 4.327873875 |
| **Molecular function category** | | | | | | | | | |
| **GO ID** | **GO Term** | **DifGene** | **AllDifGene** | **GeneInGO** | **AllGene** | **P-Value** | **FDR** | **Enrichment** | **(-log2P)** |
| GO:0003824 | catalytic activity | 61 | 810 | 727 | 16096 | 0.000305599 | 0.173132 | 1.6673561 | 11.67607129 |
| GO:0016740 | transferase activity | 109 | 810 | 1507 | 16096 | 0.000556499 | 0.173132 | 1.4372959 | 10.81133253 |
| GO:0016491 | oxidoreductase activity | 52 | 810 | 619 | 16096 | 0.000748565 | 0.173132 | 1.6693432 | 10.38358494 |
| GO:0051287 | NAD binding | 9 | 810 | 43 | 16096 | 0.000775454 | 0.173132 | 4.1591731 | 10.33267086 |
| GO:0042803 | protein homodimerization activity | 57 | 810 | 700 | 16096 | 0.000869136 | 0.173132 | 1.6181164 | 10.16812967 |
| GO:0042813 | Wnt-activated receptor activity | 6 | 810 | 20 | 16096 | 0.001235263 | 0.2050536 | 5.9614815 | 9.66096631 |
| GO:0070888 | E-box binding | 7 | 810 | 29 | 16096 | 0.001447424 | 0.2059477 | 4.7965943 | 9.432297057 |

| | | | | | | | | | |
|---|---|---|---|---|---|---|---|---|---|
| GO:0005159 | insulin-like growth factor receptor binding | 5 | 810 | 15 | 16096 | 0.002164822 | 0.2242243 | 6.6238683 | 8.851535625 |
| GO:0016616 | oxidoreductase activity, acting on the CH-OH group of donors, NAD or NADP as acceptor | 8 | 810 | 41 | 16096 | 0.002204909 | 0.2242243 | 3.8773863 | 8.825065129 |
| GO:0001968 | fibronectin binding | 6 | 810 | 23 | 16096 | 0.002251248 | 0.2242243 | 5.1838969 | 8.795059465 |
| GO:0019899 | enzyme binding | 31 | 810 | 343 | 16096 | 0.002777114 | 0.2399359 | 1.795976 | 8.492197923 |
| GO:0016829 | lyase activity | 17 | 810 | 151 | 16096 | 0.003285272 | 0.2399359 | 2.2372006 | 8.249771381 |
| GO:0004723 | calcium-dependent protein serine/threonine phosphatase activity | 3 | 810 | 4 | 16096 | 0.003348351 | 0.2399359 | 14.903704 | 8.222333518 |
| GO:0030170 | pyridoxal phosphate binding | 9 | 810 | 55 | 16096 | 0.003452681 | 0.2399359 | 3.2517172 | 8.178067282 |
| GO:0047115 | trans-1,2-dihydrobenzene-1,2-diol dehydrogenase activity | 4 | 810 | 10 | 16096 | 0.003613493 | 0.2399359 | 7.948642 | 8.112390132 |
| GO:0030971 | receptor tyrosine kinase binding | 7 | 810 | 36 | 16096 | 0.004162611 | 0.2591225 | 3.8639232 | 7.908295672 |
| GO:0009374 | biotin binding | 3 | 810 | 5 | 16096 | 0.005165936 | 0.3026631 | 11.922963 | 7.596754467 |
| GO:0031406 | carboxylic acid binding | 4 | 810 | 12 | 16096 | 0.006082281 | 0.3196876 | 6.6238683 | 7.3611719 |
| GO:0003727 | single-stranded RNA binding | 7 | 810 | 39 | 16096 | 0.006098459 | 0.3196876 | 3.5666983 | 7.357339552 |
| GO:0019901 | protein kinase binding | 32 | 810 | 384 | 16096 | 0.007141676 | 0.3556555 | 1.6559671 | 7.129521512 |
| GO:0004089 | carbonate dehydratase activity | 4 | 810 | 14 | 16096 | 0.009469974 | 0.4031263 | 5.6776014 | 6.722423745 |
| GO:0015459 | potassium channel regulator activity | 6 | 810 | 33 | 16096 | 0.010257415 | 0.4031263 | 3.6130191 | 6.607189005 |
| GO:0045503 | dynein light chain binding | 2 | 810 | 2 | 16096 | 0.01295185 | 0.4031263 | 19.871605 | 6.27069798 |
| GO:0016768 | spermine synthase activity | 2 | 810 | 2 | 16096 | 0.01295185 | 0.4031263 | 19.871605 | 6.27069798 |
| GO:0072572 | poly-ADP-D-ribose binding | 2 | 810 | 2 | 16096 | 0.01295185 | 0.4031263 | 19.871605 | 6.27069798 |
| GO:0098599 | palmitoyl hydrolase activity | 2 | 810 | 2 | 16096 | 0.01295185 | 0.4031263 | 19.871605 | 6.27069798 |
| GO:0004368 | glycerol-3-phosphate dehydrogenase activity | 2 | 810 | 2 | 16096 | 0.01295185 | 0.4031263 | 19.871605 | 6.27069798 |
| GO:0030395 | lactose binding | 2 | 810 | 2 | 16096 | 0.01295185 | 0.4031263 | 19.871605 | 6.27069798 |
| GO:0004658 | propionyl-CoA carboxylase activity | 2 | 810 | 2 | 16096 | 0.01295185 | 0.4031263 | 19.871605 | 6.27069798 |
| GO:0016422 | mRNA (2'-O-methyladenosine-N6-)-methyltransferase activity | 2 | 810 | 2 | 16096 | 0.01295185 | 0.4031263 | 19.871605 | 6.27069798 |
| GO:0004457 | lactate dehydrogenase activity | 2 | 810 | 2 | 16096 | 0.01295185 | 0.4031263 | 19.871605 | 6.27069798 |
| GO:0004450 | isocitrate dehydrogenase (NADP+) activity | 2 | 810 | 2 | 16096 | 0.01295185 | 0.4031263 | 19.871605 | 6.27069798 |
| GO:0003857 | 3-hydroxyacyl-CoA dehydrogenase activity | 3 | 810 | 8 | 16096 | 0.013655144 | 0.4068799 | 7.4518519 | 6.194411645 |
| GO:0008199 | ferric iron binding | 4 | 810 | 16 | 16096 | 0.013889476 | 0.4068799 | 4.9679012 | 6.169864033 |
| GO:0005178 | integrin binding | 12 | 810 | 110 | 16096 | 0.01487595 | 0.413949 | 2.1678114 | 6.070874434 |
| GO:0017147 | Wnt-protein binding | 5 | 810 | 26 | 16096 | 0.015294151 | 0.413949 | 3.8214625 | 6.030876196 |
| GO:0016655 | oxidoreductase activity, acting on NAD(P)H, quinone or similar compound as acceptor | 4 | 810 | 17 | 16096 | 0.01651539 | 0.413949 | 4.6756718 | 5.920045141 |
| GO:0018636 | phenanthrene 9,10-monooxygenase activity | 3 | 810 | 9 | 16096 | 0.017563277 | 0.413949 | 6.6238683 | 5.831294117 |
| GO:0047086 | ketosteroid monooxygenase activity | 3 | 810 | 9 | 16096 | 0.017563277 | 0.413949 | 6.6238683 | 5.831294117 |

| | | | | | | | | | |
|---|---|---|---|---|---|---|---|---|---|
| GO:0031994 | insulin-like growth factor I binding | 3 | 810 | 9 | 16096 | 0.017563277 | 0.413949 | 6.6238683 | 5.831294117 |
| GO:0023026 | MHC class II protein complex binding | 4 | 810 | 18 | 16096 | 0.019431461 | 0.413949 | 4.4159122 | 5.685461805 |
| GO:0004721 | phosphoprotein phosphatase activity | 15 | 810 | 157 | 16096 | 0.019681191 | 0.413949 | 1.898561 | 5.667038669 |
| GO:0042802 | identical protein binding | 41 | 810 | 565 | 16096 | 0.020439047 | 0.413949 | 1.4420103 | 5.612528269 |
| GO:0008168 | methyltransferase activity | 17 | 810 | 187 | 16096 | 0.020525908 | 0.413949 | 1.8065095 | 5.606410131 |
| GO:0004766 | spermidine synthase activity | 2 | 810 | 3 | 16096 | 0.020900294 | 0.413949 | 13.247737 | 5.58033297 |
| GO:0000309 | nicotinamide-nucleotide adenylyltransferase activity | 2 | 810 | 3 | 16096 | 0.020900294 | 0.413949 | 13.247737 | 5.58033297 |
| GO:0003876 | AMP deaminase activity | 2 | 810 | 3 | 16096 | 0.020900294 | 0.413949 | 13.247737 | 5.58033297 |
| GO:0008474 | palmitoyl-(protein) hydrolase activity | 2 | 810 | 3 | 16096 | 0.020900294 | 0.413949 | 13.247737 | 5.58033297 |
| GO:0004356 | glutamate-ammonia ligase activity | 2 | 810 | 3 | 16096 | 0.020900294 | 0.413949 | 13.247737 | 5.58033297 |
| GO:0032810 | sterol response element binding | 2 | 810 | 3 | 16096 | 0.020900294 | 0.413949 | 13.247737 | 5.58033297 |
| GO:0016790 | thiolester hydrolase activity | 3 | 810 | 10 | 16096 | 0.022027405 | 0.413949 | 5.9614815 | 5.504556634 |
| GO:0047718 | indanol dehydrogenase activity | 3 | 810 | 10 | 16096 | 0.022027405 | 0.413949 | 5.9614815 | 5.504556634 |
| GO:0043522 | leucine zipper domain binding | 3 | 810 | 10 | 16096 | 0.022027405 | 0.413949 | 5.9614815 | 5.504556634 |
| GO:0004033 | aldo-keto reductase (NADP) activity | 4 | 810 | 19 | 16096 | 0.022645412 | 0.417682 | 4.1834958 | 5.464637408 |
| GO:0031369 | translation initiation factor binding | 4 | 810 | 20 | 16096 | 0.02616353 | 0.4325426 | 3.974321 | 5.256298985 |
| GO:0004683 | calmodulin-dependent protein kinase activity | 4 | 810 | 20 | 16096 | 0.02616353 | 0.4325426 | 3.974321 | 5.256298985 |
| GO:0030554 | adenyl nucleotide binding | 4 | 810 | 20 | 16096 | 0.02616353 | 0.4325426 | 3.974321 | 5.256298985 |
| GO:0031748 | D1 dopamine receptor binding | 3 | 810 | 11 | 16096 | 0.027049381 | 0.4325426 | 5.4195286 | 5.2082606 |
| GO:0004861 | cyclin-dependent protein serine/threonine kinase inhibitor activity | 3 | 810 | 11 | 16096 | 0.027049381 | 0.4325426 | 5.4195286 | 5.2082606 |
| GO:0003705 | RNA polymerase II distal enhancer sequence-specific DNA binding transcription factor activity | 8 | 810 | 67 | 16096 | 0.027511225 | 0.4325426 | 2.3727289 | 5.183835813 |
| GO:0031014 | troponin T binding | 2 | 810 | 4 | 16096 | 0.030358222 | 0.4325426 | 9.9358025 | 5.04176887 |
| GO:0043560 | insulin receptor substrate binding | 3 | 810 | 12 | 16096 | 0.032626502 | 0.4325426 | 4.9679012 | 4.937811868 |
| GO:0043295 | glutathione binding | 3 | 810 | 12 | 16096 | 0.032626502 | 0.4325426 | 4.9679012 | 4.937811868 |
| GO:0030235 | nitric-oxide synthase regulator activity | 3 | 810 | 12 | 16096 | 0.032626502 | 0.4325426 | 4.9679012 | 4.937811868 |
| GO:0008483 | transaminase activity | 4 | 810 | 22 | 16096 | 0.034130407 | 0.4325426 | 3.6130191 | 4.872798571 |
| GO:0043021 | ribonucleoprotein complex binding | 4 | 810 | 23 | 16096 | 0.038584846 | 0.4325426 | 3.4559313 | 4.695821831 |
| GO:0050840 | extracellular matrix binding | 4 | 810 | 23 | 16096 | 0.038584846 | 0.4325426 | 3.4559313 | 4.695821831 |
| GO:0004032 | alditol:NADP+ 1-oxidoreductase activity | 3 | 810 | 13 | 16096 | 0.038752092 | 0.4325426 | 4.585755 | 4.689582009 |
| GO:0000988 | protein binding transcription factor activity | 2 | 810 | 5 | 16096 | 0.041162171 | 0.4325426 | 7.948642 | 4.602537112 |
| GO:0043559 | insulin binding | 2 | 810 | 5 | 16096 | 0.041162171 | 0.4325426 | 7.948642 | 4.602537112 |
| GO:0046923 | ER retention sequence binding | 2 | 810 | 5 | 16096 | 0.041162171 | 0.4325426 | 7.948642 | 4.602537112 |

| | | | | | | | | | |
|---|---|---|---|---|---|---|---|---|---|
| GO:0015125 | bile acid transmembrane transporter activity | 2 | 810 | 5 | 16096 | 0.041162171 | 0.4325426 | 7.948642 | 4.602537112 |
| GO:0004300 | enoyl-CoA hydratase activity | 2 | 810 | 5 | 16096 | 0.041162171 | 0.4325426 | 7.948642 | 4.602537112 |
| GO:0004515 | nicotinate-nucleotide adenylyltransferase activity | 2 | 810 | 5 | 16096 | 0.041162171 | 0.4325426 | 7.948642 | 4.602537112 |
| GO:0004351 | glutamate decarboxylase activity | 2 | 810 | 5 | 16096 | 0.041162171 | 0.4325426 | 7.948642 | 4.602537112 |
| GO:0005007 | fibroblast growth factor-activated receptor activity | 2 | 810 | 5 | 16096 | 0.041162171 | 0.4325426 | 7.948642 | 4.602537112 |
| GO:0004459 | L-lactate dehydrogenase activity | 2 | 810 | 5 | 16096 | 0.041162171 | 0.4325426 | 7.948642 | 4.602537112 |
| GO:0047042 | androsterone dehydrogenase (B-specific) activity | 2 | 810 | 5 | 16096 | 0.041162171 | 0.4325426 | 7.948642 | 4.602537112 |
| GO:0008060 | ARF GTPase activator activity | 4 | 810 | 24 | 16096 | 0.043354909 | 0.4325426 | 3.3119342 | 4.527660828 |
| GO:0022891 | substrate-specific transmembrane transporter activity | 3 | 810 | 14 | 16096 | 0.045416031 | 0.4325426 | 4.2582011 | 4.460654544 |
| GO:0009055 | electron carrier activity | 10 | 810 | 104 | 16096 | 0.048368377 | 0.4325426 | 1.9107312 | 4.369792057 |
| **Cellular component category** | | | | | | | | | |
| **GOID** | **GOTerm** | **DifGene** | **AllDifGene** | **GeneInGO** | **AllGene** | **P-Value** | **FDR** | **Enrichment** | **(-log2P)** |
| GO:0030018 | Z disc | 21 | 853 | 119 | 17318 | 3.05082E-06 | 0.0014552 | 3.5827874 | 18.32237141 |
| GO:0005829 | cytosol | 212 | 853 | 3053 | 17318 | 1.52001E-05 | 0.0032697 | 1.4098008 | 16.0055618 |
| GO:0016529 | sarcoplasmic reticulum | 12 | 853 | 48 | 17318 | 2.05641E-05 | 0.0032697 | 5.0756155 | 15.56951448 |
| GO:0005739 | mitochondrion | 125 | 853 | 1671 | 17318 | 3.3237E-05 | 0.0039635 | 1.5187359 | 14.87684885 |
| GO:0005759 | mitochondrial matrix | 31 | 853 | 271 | 17318 | 5.51566E-05 | 0.0052619 | 2.3224218 | 14.14610762 |
| GO:0033017 | sarcoplasmic reticulum membrane | 8 | 853 | 31 | 17318 | 0.000402662 | 0.0320116 | 5.239345 | 11.27814404 |
| GO:0042383 | sarcolemma | 13 | 853 | 92 | 17318 | 0.001373588 | 0.0936002 | 2.8688261 | 9.507834969 |
| GO:0005604 | basement membrane | 11 | 853 | 91 | 17318 | 0.008765807 | 0.3809176 | 2.4541437 | 6.833897315 |
| GO:0031362 | anchored component of external side of plasma membrane | 4 | 853 | 15 | 17318 | 0.010760724 | 0.3809176 | 5.4139898 | 6.538081025 |
| GO:0031224 | intrinsic component of membrane | 4 | 853 | 15 | 17318 | 0.010760724 | 0.3809176 | 5.4139898 | 6.538081025 |
| GO:0030016 | myofibril | 7 | 853 | 46 | 17318 | 0.011821594 | 0.3809176 | 3.0895051 | 6.402431593 |
| GO:0005712 | chiasma | 2 | 853 | 2 | 17318 | 0.012447791 | 0.3809176 | 20.302462 | 6.327966478 |
| GO:0005861 | troponin complex | 3 | 853 | 8 | 17318 | 0.012916155 | 0.3809176 | 7.6134232 | 6.274679476 |
| GO:0005751 | mitochondrial respiratory chain complex IV | 3 | 853 | 8 | 17318 | 0.012916155 | 0.3809176 | 7.6134232 | 6.274679476 |
| GO:0033018 | sarcoplasmic reticulum lumen | 3 | 853 | 8 | 17318 | 0.012916155 | 0.3809176 | 7.6134232 | 6.274679476 |
| GO:0031672 | A band | 4 | 853 | 16 | 17318 | 0.012956463 | 0.3809176 | 5.0756155 | 6.27018422 |
| GO:0005730 | nucleolus | 120 | 853 | 1944 | 17318 | 0.015644076 | 0.3809176 | 1.2532384 | 5.998239719 |
| GO:0000307 | cyclin-dependent protein kinase holoenzyme complex | 3 | 853 | 9 | 17318 | 0.016624952 | 0.3809176 | 6.7674873 | 5.910506049 |
| GO:0070062 | extracellular vesicular exosome | 177 | 853 | 2993 | 17318 | 0.018746895 | 0.3809176 | 1.2006468 | 5.737204509 |
| GO:0009331 | glycerol-3-phosphate dehydrogenase complex | 2 | 853 | 3 | 17318 | 0.020100228 | 0.3809176 | 13.534975 | 5.636644295 |

| | | | | | | | | | |
|---|---|---|---|---|---|---|---|---|---|
| GO:0005899 | insulin receptor complex | 2 | 853 | 3 | 17318 | 0.020100228 | 0.3809176 | 13.534975 | 5.636644295 |
| GO:0070545 | PeBoW complex | 2 | 853 | 3 | 17318 | 0.020100228 | 0.3809176 | 13.534975 | 5.636644295 |
| GO:0005955 | calcineurin complex | 2 | 853 | 3 | 17318 | 0.020100228 | 0.3809176 | 13.534975 | 5.636644295 |
| GO:0036396 | MIS complex | 2 | 853 | 3 | 17318 | 0.020100228 | 0.3809176 | 13.534975 | 5.636644295 |
| GO:0030017 | sarcomere | 7 | 853 | 52 | 17318 | 0.020517501 | 0.3809176 | 2.7330237 | 5.607001188 |
| GO:0005737 | cytoplasm | 358 | 853 | 6352 | 17318 | 0.020762805 | 0.3809176 | 1.1442508 | 5.589854858 |
| GO:0031012 | extracellular matrix | 20 | 853 | 242 | 17318 | 0.024658425 | 0.422294 | 1.6778894 | 5.341775532 |
| GO:0005791 | rough endoplasmic reticulum | 6 | 853 | 43 | 17318 | 0.026860912 | 0.422294 | 2.8329017 | 5.218347897 |
| GO:0045254 | pyruvate dehydrogenase complex | 2 | 853 | 4 | 17318 | 0.02921531 | 0.422294 | 10.151231 | 5.097131586 |
| GO:0030687 | preribosome, large subunit precursor | 2 | 853 | 4 | 17318 | 0.02921531 | 0.422294 | 10.151231 | 5.097131586 |
| GO:0032777 | Piccolo NuA4 histone acetyltransferase complex | 2 | 853 | 4 | 17318 | 0.02921531 | 0.422294 | 10.151231 | 5.097131586 |
| GO:0097386 | glial cell projection | 2 | 853 | 4 | 17318 | 0.02921531 | 0.422294 | 10.151231 | 5.097131586 |
| GO:0033010 | paranodal junction | 2 | 853 | 4 | 17318 | 0.02921531 | 0.422294 | 10.151231 | 5.097131586 |
| GO:0097038 | perinuclear endoplasmic reticulum | 3 | 853 | 12 | 17318 | 0.030950474 | 0.4303855 | 5.0756155 | 5.013894669 |
| GO:0005891 | voltage-gated calcium channel complex | 5 | 853 | 33 | 17318 | 0.031792791 | 0.4303855 | 3.0761306 | 4.975156536 |
| GO:0030176 | integral component of endoplasmic reticulum membrane | 8 | 853 | 71 | 17318 | 0.032481928 | 0.4303855 | 2.2876013 | 4.944218937 |
| GO:0046658 | anchored component of plasma membrane | 4 | 853 | 23 | 17318 | 0.036184741 | 0.4501782 | 3.5308629 | 4.788474735 |
| GO:0030659 | cytoplasmic vesicle membrane | 12 | 853 | 130 | 17318 | 0.036744952 | 0.4501782 | 1.8740734 | 4.766310133 |
| GO:0030424 | axon | 19 | 853 | 240 | 17318 | 0.039266895 | 0.4501782 | 1.6072782 | 4.670542681 |
| GO:0032300 | mismatch repair complex | 2 | 853 | 5 | 17318 | 0.039638334 | 0.4501782 | 8.1209848 | 4.656959848 |
| GO:0005652 | nuclear lamina | 2 | 853 | 5 | 17318 | 0.039638334 | 0.4501782 | 8.1209848 | 4.656959848 |
| GO:0005927 | muscle tendon junction | 2 | 853 | 5 | 17318 | 0.039638334 | 0.4501782 | 8.1209848 | 4.656959848 |
| GO:0001725 | stress fiber | 7 | 853 | 62 | 17318 | 0.043427502 | 0.4758579 | 2.2922134 | 4.525247224 |

## Table S3: GO-analysis of up-regulated DEGs (STH vs QHMM)

| Biological process category | | | | | | | | | |
|---|---|---|---|---|---|---|---|---|---|
| **GOID** | **GOTerm** | **DifGene** | **AllDifGene** | **GeneInGO** | **AllGene** | **P-Value** | **FDR** | **Enrichment** | **(-log2P)** |
| GO:0046627 | negative regulation of insulin receptor signaling pathway | 6 | 345 | 27 | 16457 | 5.41617E-05 | 0.0873629 | 10.600322 | 14.17236613 |
| GO:0042254 | ribosome biogenesis | 8 | 345 | 81 | 16457 | 0.000544836 | 0.3374783 | 4.7112542 | 10.84188939 |
| GO:0009267 | cellular response to starvation | 6 | 345 | 47 | 16457 | 0.000786244 | 0.3374783 | 6.0895467 | 10.31273539 |
| GO:0051170 | nuclear import | 3 | 345 | 7 | 16457 | 0.000947037 | 0.3374783 | 20.443478 | 10.04429154 |
| GO:0008652 | cellular amino acid biosynthetic process | 5 | 345 | 35 | 16457 | 0.001363636 | 0.3374783 | 6.8144928 | 9.518325983 |
| GO:0006405 | RNA export from nucleus | 3 | 345 | 9 | 16457 | 0.001683017 | 0.3374783 | 15.900483 | 9.214734296 |
| GO:0051726 | regulation of cell cycle | 10 | 345 | 149 | 16457 | 0.001928425 | 0.3374783 | 3.2014395 | 9.018361053 |
| GO:2001235 | positive regulation of apoptotic signaling pathway | 5 | 345 | 39 | 16457 | 0.002101173 | 0.3374783 | 6.1155704 | 8.894589009 |
| GO:0045444 | fat cell differentiation | 6 | 345 | 60 | 16457 | 0.002473326 | 0.3374783 | 4.7701449 | 8.659332035 |
| GO:0019087 | transformation of host cell by virus | 2 | 345 | 2 | 16457 | 0.002481381 | 0.3374783 | 47.701449 | 8.654640997 |
| GO:0032929 | negative regulation of superoxide anion generation | 2 | 345 | 2 | 16457 | 0.002481381 | 0.3374783 | 47.701449 | 8.654640997 |
| GO:0010508 | positive regulation of autophagy | 4 | 345 | 24 | 16457 | 0.002510688 | 0.3374783 | 7.9502415 | 8.637701551 |
| GO:0071353 | cellular response to interleukin-4 | 4 | 345 | 26 | 16457 | 0.003251537 | 0.3448734 | 7.3386845 | 8.2646625 |
| GO:0006366 | transcription from RNA polymerase II promoter | 21 | 345 | 495 | 16457 | 0.003331363 | 0.3448734 | 2.0236978 | 8.229671539 |
| GO:0006536 | glutamate metabolic process | 3 | 345 | 13 | 16457 | 0.004026313 | 0.3448734 | 11.008027 | 7.956324811 |
| GO:2000649 | regulation of sodium ion transmembrane transporter activity | 3 | 345 | 13 | 16457 | 0.004026313 | 0.3448734 | 11.008027 | 7.956324811 |
| GO:0051168 | nuclear export | 3 | 345 | 13 | 16457 | 0.004026313 | 0.3448734 | 11.008027 | 7.956324811 |
| GO:0006542 | glutamine biosynthetic process | 2 | 345 | 3 | 16457 | 0.004078746 | 0.3448734 | 31.800966 | 7.93765861 |
| GO:0000466 | maturation of 5.8S rRNA from tricistronic rRNA transcript (SSU-rRNA, 5.8S rRNA, LSU-rRNA) | 2 | 345 | 3 | 16457 | 0.004078746 | 0.3448734 | 31.800966 | 7.93765861 |
| GO:0002028 | regulation of sodium ion transport | 3 | 345 | 14 | 16457 | 0.004814059 | 0.3448734 | 10.221739 | 7.698530329 |
| GO:0051260 | protein homooligomerization | 10 | 345 | 173 | 16457 | 0.005227014 | 0.3448734 | 2.7573092 | 7.57979724 |
| GO:0040008 | regulation of growth | 6 | 345 | 72 | 16457 | 0.005664385 | 0.3448734 | 3.9751208 | 7.463864972 |
| GO:0006880 | intracellular sequestering of iron ion | 2 | 345 | 4 | 16457 | 0.006034112 | 0.3448734 | 23.850725 | 7.372642906 |
| GO:0006597 | spermine biosynthetic process | 2 | 345 | 4 | 16457 | 0.006034112 | 0.3448734 | 23.850725 | 7.372642906 |

| | | | | | | | | | |
|---|---|---|---|---|---|---|---|---|---|
| GO:0070814 | hydrogen sulfide biosynthetic process | 2 | 345 | 4 | 16457 | 0.006034112 | 0.3448734 | 23.850725 | 7.372642906 |
| GO:0061157 | mRNA destabilization | 2 | 345 | 4 | 16457 | 0.006034112 | 0.3448734 | 23.850725 | 7.372642906 |
| GO:0080009 | mRNA methylation | 2 | 345 | 4 | 16457 | 0.006034112 | 0.3448734 | 23.850725 | 7.372642906 |
| GO:0000398 | mRNA splicing, via spliceosome | 11 | 345 | 207 | 16457 | 0.00632651 | 0.3448734 | 2.5348596 | 7.30437435 |
| GO:0046426 | negative regulation of JAK-STAT cascade | 3 | 345 | 16 | 16457 | 0.006651444 | 0.3448734 | 8.9440217 | 7.232116724 |
| GO:0006417 | regulation of translation | 8 | 345 | 126 | 16457 | 0.007071235 | 0.3448734 | 3.0286634 | 7.143821979 |
| GO:0033365 | protein localization to organelle | 3 | 345 | 17 | 16457 | 0.007705551 | 0.3448734 | 8.4179028 | 7.019886193 |
| GO:0071354 | cellular response to interleukin-6 | 3 | 345 | 17 | 16457 | 0.007705551 | 0.3448734 | 8.4179028 | 7.019886193 |
| GO:0006538 | glutamate catabolic process | 2 | 345 | 5 | 16457 | 0.008331972 | 0.3448734 | 19.08058 | 6.907126378 |
| GO:0010815 | bradykinin catabolic process | 2 | 345 | 5 | 16457 | 0.008331972 | 0.3448734 | 19.08058 | 6.907126378 |
| GO:0000463 | maturation of LSU-rRNA from tricistronic rRNA transcript (SSU-rRNA, 5.8S rRNA, LSU-rRNA) | 2 | 345 | 5 | 16457 | 0.008331972 | 0.3448734 | 19.08058 | 6.907126378 |
| GO:0000380 | alternative mRNA splicing, via spliceosome | 3 | 345 | 18 | 16457 | 0.008852483 | 0.3448734 | 7.9502415 | 6.819702101 |
| GO:0008285 | negative regulation of cell proliferation | 17 | 345 | 414 | 16457 | 0.009955552 | 0.3448734 | 1.9587552 | 6.650283 |
| GO:0048102 | autophagic cell death | 2 | 345 | 6 | 16457 | 0.01095731 | 0.3448734 | 15.900483 | 6.511962593 |
| GO:0006555 | methionine metabolic process | 2 | 345 | 6 | 16457 | 0.01095731 | 0.3448734 | 15.900483 | 6.511962593 |
| GO:0006563 | L-serine metabolic process | 2 | 345 | 6 | 16457 | 0.01095731 | 0.3448734 | 15.900483 | 6.511962593 |
| GO:2000643 | positive regulation of early endosome to late endosome transport | 2 | 345 | 6 | 16457 | 0.01095731 | 0.3448734 | 15.900483 | 6.511962593 |
| GO:0010656 | negative regulation of muscle cell apoptotic process | 2 | 345 | 6 | 16457 | 0.01095731 | 0.3448734 | 15.900483 | 6.511962593 |
| GO:0010324 | membrane invagination | 2 | 345 | 6 | 16457 | 0.01095731 | 0.3448734 | 15.900483 | 6.511962593 |
| GO:0070295 | renal water absorption | 2 | 345 | 6 | 16457 | 0.01095731 | 0.3448734 | 15.900483 | 6.511962593 |
| GO:0034405 | response to fluid shear stress | 2 | 345 | 6 | 16457 | 0.01095731 | 0.3448734 | 15.900483 | 6.511962593 |
| GO:0006413 | translational initiation | 12 | 345 | 256 | 16457 | 0.011049715 | 0.3448734 | 2.2360054 | 6.499847051 |
| GO:0034641 | cellular nitrogen compound metabolic process | 10 | 345 | 195 | 16457 | 0.011127475 | 0.3448734 | 2.4462282 | 6.489729878 |
| GO:0045736 | negative regulation of cyclin-dependent protein serine/threonine kinase activity | 3 | 345 | 20 | 16457 | 0.011431026 | 0.3448734 | 7.1552174 | 6.45090131 |
| GO:0006807 | nitrogen compound metabolic process | 3 | 345 | 20 | 16457 | 0.011431026 | 0.3448734 | 7.1552174 | 6.45090131 |
| GO:0006595 | polyamine metabolic process | 3 | 345 | 20 | 16457 | 0.011431026 | 0.3448734 | 7.1552174 | 6.45090131 |
| GO:0006914 | autophagy | 7 | 345 | 113 | 16457 | 0.012751399 | 0.3448734 | 2.954957 | 6.293200633 |
| GO:0007346 | regulation of mitotic cell cycle | 4 | 345 | 41 | 16457 | 0.013831863 | 0.3448734 | 4.6537999 | 6.175860666 |
| GO:0071455 | cellular response to hyperoxia | 2 | 345 | 7 | 16457 | 0.013895586 | 0.3448734 | 13.628986 | 6.169229556 |

| | | | | | | | | | |
|---|---|---|---|---|---|---|---|---|---|
| GO:0071493 | cellular response to UV-B | 2 | 345 | 7 | 16457 | 0.013895586 | 0.3448734 | 13.628986 | 6.169229556 |
| GO:0001504 | neurotransmitter uptake | 2 | 345 | 7 | 16457 | 0.013895586 | 0.3448734 | 13.628986 | 6.169229556 |
| GO:0071340 | skeletal muscle acetylcholine-gated channel clustering | 2 | 345 | 7 | 16457 | 0.013895586 | 0.3448734 | 13.628986 | 6.169229556 |
| GO:0031017 | exocrine pancreas development | 2 | 345 | 8 | 16457 | 0.017132723 | 0.3448734 | 11.925362 | 5.867101748 |
| GO:0042118 | endothelial cell activation | 2 | 345 | 8 | 16457 | 0.017132723 | 0.3448734 | 11.925362 | 5.867101748 |
| GO:0071294 | cellular response to zinc ion | 2 | 345 | 8 | 16457 | 0.017132723 | 0.3448734 | 11.925362 | 5.867101748 |
| GO:0032873 | negative regulation of stress-activated MAPK cascade | 2 | 345 | 8 | 16457 | 0.017132723 | 0.3448734 | 11.925362 | 5.867101748 |
| GO:0000045 | autophagic vacuole assembly | 4 | 345 | 44 | 16457 | 0.017205391 | 0.3448734 | 4.3364954 | 5.86099551 |
| GO:0048041 | focal adhesion assembly | 3 | 345 | 24 | 16457 | 0.017758048 | 0.3448734 | 5.9626812 | 5.815383161 |
| GO:0032312 | regulation of ARF GTPase activity | 3 | 345 | 24 | 16457 | 0.017758048 | 0.3448734 | 5.9626812 | 5.815383161 |
| GO:0051412 | response to corticosterone | 3 | 345 | 25 | 16457 | 0.019587732 | 0.3448734 | 5.7241739 | 5.673905864 |
| GO:0045944 | positive regulation of transcription from RNA polymerase II promoter | 29 | 345 | 891 | 16457 | 0.020598087 | 0.3448734 | 1.5525724 | 5.601345831 |
| GO:0000185 | activation of MAPKKK activity | 2 | 345 | 9 | 16457 | 0.020655094 | 0.3448734 | 10.600322 | 5.597358576 |
| GO:0042117 | monocyte activation | 2 | 345 | 9 | 16457 | 0.020655094 | 0.3448734 | 10.600322 | 5.597358576 |
| GO:0055013 | cardiac muscle cell development | 2 | 345 | 9 | 16457 | 0.020655094 | 0.3448734 | 10.600322 | 5.597358576 |
| GO:0001558 | regulation of cell growth | 5 | 345 | 71 | 16457 | 0.020765101 | 0.3448734 | 3.359257 | 5.589695335 |
| GO:0035872 | nucleotide-binding domain, leucine rich repeat containing receptor signaling pathway | 4 | 345 | 47 | 16457 | 0.021049747 | 0.3448734 | 4.0596978 | 5.570053297 |
| GO:0051899 | membrane depolarization | 3 | 345 | 26 | 16457 | 0.021517302 | 0.3448734 | 5.5040134 | 5.538359016 |
| GO:2000377 | regulation of reactive oxygen species metabolic process | 3 | 345 | 26 | 16457 | 0.021517302 | 0.3448734 | 5.5040134 | 5.538359016 |
| GO:0097193 | intrinsic apoptotic signaling pathway | 5 | 345 | 73 | 16457 | 0.022945367 | 0.3448734 | 3.2672226 | 5.445653313 |
| GO:0043066 | negative regulation of apoptotic process | 19 | 345 | 530 | 16457 | 0.023209302 | 0.3448734 | 1.710052 | 5.429153027 |
| GO:0045840 | positive regulation of mitosis | 3 | 345 | 27 | 16457 | 0.023546831 | 0.3448734 | 5.300161 | 5.408323271 |
| GO:0006497 | protein lipidation | 2 | 345 | 10 | 16457 | 0.024449509 | 0.3448734 | 9.5402899 | 5.354050688 |
| GO:2000177 | regulation of neural precursor cell proliferation | 2 | 345 | 10 | 16457 | 0.024449509 | 0.3448734 | 9.5402899 | 5.354050688 |
| GO:0030238 | male sex determination | 2 | 345 | 10 | 16457 | 0.024449509 | 0.3448734 | 9.5402899 | 5.354050688 |
| GO:0043068 | positive regulation of programmed cell death | 2 | 345 | 10 | 16457 | 0.024449509 | 0.3448734 | 9.5402899 | 5.354050688 |
| GO:0002639 | positive regulation of immunoglobulin production | 2 | 345 | 10 | 16457 | 0.024449509 | 0.3448734 | 9.5402899 | 5.354050688 |
| GO:0045892 | negative regulation of transcription, DNA-templated | 18 | 345 | 499 | 16457 | 0.025144416 | 0.3448734 | 1.7206936 | 5.313618141 |
| GO:0006486 | protein glycosylation | 8 | 345 | 161 | 16457 | 0.0252521 | 0.3448734 | 2.3702583 | 5.30745284 |

| | | | | | | | | | |
|---|---|---|---|---|---|---|---|---|---|
| GO:0019228 | neuronal action potential | 3 | 345 | 29 | 16457 | 0.027905319 | 0.3448734 | 4.9346327 | 5.163316026 |
| GO:0042273 | ribosomal large subunit biogenesis | 3 | 345 | 29 | 16457 | 0.027905319 | 0.3448734 | 4.9346327 | 5.163316026 |
| GO:0006364 | rRNA processing | 7 | 345 | 134 | 16457 | 0.027989184 | 0.3448734 | 2.4918668 | 5.158986751 |
| GO:0051593 | response to folic acid | 2 | 345 | 11 | 16457 | 0.028503204 | 0.3448734 | 8.6729908 | 5.132732097 |
| GO:2000573 | positive regulation of DNA biosynthetic process | 2 | 345 | 11 | 16457 | 0.028503204 | 0.3448734 | 8.6729908 | 5.132732097 |
| GO:0071850 | mitotic cell cycle arrest | 2 | 345 | 11 | 16457 | 0.028503204 | 0.3448734 | 8.6729908 | 5.132732097 |
| GO:0043129 | surfactant homeostasis | 2 | 345 | 11 | 16457 | 0.028503204 | 0.3448734 | 8.6729908 | 5.132732097 |
| GO:0051290 | protein heterotetramerization | 2 | 345 | 11 | 16457 | 0.028503204 | 0.3448734 | 8.6729908 | 5.132732097 |
| GO:0015909 | long-chain fatty acid transport | 2 | 345 | 11 | 16457 | 0.028503204 | 0.3448734 | 8.6729908 | 5.132732097 |
| GO:0006879 | cellular iron ion homeostasis | 5 | 345 | 78 | 16457 | 0.029024656 | 0.3448734 | 3.0577852 | 5.10657721 |
| GO:0048147 | negative regulation of fibroblast proliferation | 3 | 345 | 30 | 16457 | 0.03023371 | 0.3448734 | 4.7701449 | 5.047698151 |
| GO:0060397 | JAK-STAT cascade involved in growth hormone signaling pathway | 3 | 345 | 30 | 16457 | 0.03023371 | 0.3448734 | 4.7701449 | 5.047698151 |
| GO:0055086 | nucleobase-containing small molecule metabolic process | 5 | 345 | 79 | 16457 | 0.030350736 | 0.3448734 | 3.0190791 | 5.042124671 |
| GO:0043065 | positive regulation of apoptotic process | 13 | 345 | 334 | 16457 | 0.030992995 | 0.3448734 | 1.8566432 | 5.011913995 |
| GO:0042593 | glucose homeostasis | 6 | 345 | 108 | 16457 | 0.031537357 | 0.3448734 | 2.6500805 | 4.986794409 |
| GO:0007050 | cell cycle arrest | 7 | 345 | 138 | 16457 | 0.031877811 | 0.3448734 | 2.4196387 | 4.971303617 |
| GO:0033235 | positive regulation of protein sumoylation | 2 | 345 | 12 | 16457 | 0.032803826 | 0.3448734 | 7.9502415 | 4.929992092 |
| GO:0002181 | cytoplasmic translation | 2 | 345 | 12 | 16457 | 0.032803826 | 0.3448734 | 7.9502415 | 4.929992092 |
| GO:0030330 | DNA damage response, signal transduction by p53 class mediator | 2 | 345 | 12 | 16457 | 0.032803826 | 0.3448734 | 7.9502415 | 4.929992092 |
| GO:0006978 | DNA damage response, signal transduction by p53 class mediator resulting in transcription of p21 class mediator | 2 | 345 | 12 | 16457 | 0.032803826 | 0.3448734 | 7.9502415 | 4.929992092 |
| GO:0019985 | translesion synthesis | 2 | 345 | 12 | 16457 | 0.032803826 | 0.3448734 | 7.9502415 | 4.929992092 |
| GO:0045926 | negative regulation of growth | 2 | 345 | 12 | 16457 | 0.032803826 | 0.3448734 | 7.9502415 | 4.929992092 |
| GO:0055093 | response to hyperoxia | 2 | 345 | 12 | 16457 | 0.032803826 | 0.3448734 | 7.9502415 | 4.929992092 |
| GO:0009062 | fatty acid catabolic process | 2 | 345 | 12 | 16457 | 0.032803826 | 0.3448734 | 7.9502415 | 4.929992092 |
| GO:1901653 | cellular response to peptide | 2 | 345 | 12 | 16457 | 0.032803826 | 0.3448734 | 7.9502415 | 4.929992092 |
| GO:0010467 | gene expression | 29 | 345 | 932 | 16457 | 0.033061788 | 0.3448734 | 1.4842726 | 4.918691444 |
| GO:0006730 | one-carbon metabolic process | 3 | 345 | 32 | 16457 | 0.035186415 | 0.3448734 | 4.4720109 | 4.828837669 |
| GO:0051289 | protein homotetramerization | 4 | 345 | 56 | 16457 | 0.035535694 | 0.3448734 | 3.4072464 | 4.814587306 |
| GO:0001510 | RNA methylation | 2 | 345 | 13 | 16457 | 0.037339426 | 0.3448734 | 7.3386845 | 4.743156454 |
| GO:0016180 | snRNA processing | 2 | 345 | 13 | 16457 | 0.037339426 | 0.3448734 | 7.3386845 | 4.743156454 |
| GO:0014898 | cardiac muscle hypertrophy in response to stress | 2 | 345 | 13 | 16457 | 0.037339426 | 0.3448734 | 7.3386845 | 4.743156454 |
| GO:0045947 | negative regulation of translational initiation | 2 | 345 | 13 | 16457 | 0.037339426 | 0.3448734 | 7.3386845 | 4.743156454 |

| | | | | | | | | | |
|---|---|---|---|---|---|---|---|---|---|
| GO:0030488 | tRNA methylation | 2 | 345 | 13 | 16457 | 0.037339426 | 0.3448734 | 7.3386845 | 4.743156454 |
| GO:0051898 | negative regulation of protein kinase B signaling | 3 | 345 | 33 | 16457 | 0.037809431 | 0.3448734 | 4.3364954 | 4.725110054 |
| GO:0000422 | mitochondrion degradation | 3 | 345 | 34 | 16457 | 0.04052918 | 0.3448734 | 4.2089514 | 4.624895186 |
| GO:0042501 | serine phosphorylation of STAT protein | 1 | 345 | 1 | 16457 | 0.04075791 | 0.3448734 | 47.701449 | 4.616776133 |
| GO:0009386 | translational attenuation | 1 | 345 | 1 | 16457 | 0.04075791 | 0.3448734 | 47.701449 | 4.616776133 |
| GO:0060448 | dichotomous subdivision of terminal units involved in lung branching | 1 | 345 | 1 | 16457 | 0.04075791 | 0.3448734 | 47.701449 | 4.616776133 |
| GO:0001100 | negative regulation of exit from mitosis | 1 | 345 | 1 | 16457 | 0.04075791 | 0.3448734 | 47.701449 | 4.616776133 |
| GO:0070346 | positive regulation of fat cell proliferation | 1 | 345 | 1 | 16457 | 0.04075791 | 0.3448734 | 47.701449 | 4.616776133 |
| GO:0090138 | regulation of actin cytoskeleton organization by cell-cell adhesion | 1 | 345 | 1 | 16457 | 0.04075791 | 0.3448734 | 47.701449 | 4.616776133 |
| GO:0048685 | negative regulation of collateral sprouting of intact axon in response to injury | 1 | 345 | 1 | 16457 | 0.04075791 | 0.3448734 | 47.701449 | 4.616776133 |
| GO:0001544 | initiation of primordial ovarian follicle growth | 1 | 345 | 1 | 16457 | 0.04075791 | 0.3448734 | 47.701449 | 4.616776133 |
| GO:0044773 | mitotic DNA damage checkpoint | 1 | 345 | 1 | 16457 | 0.04075791 | 0.3448734 | 47.701449 | 4.616776133 |
| GO:0006535 | cysteine biosynthetic process from serine | 1 | 345 | 1 | 16457 | 0.04075791 | 0.3448734 | 47.701449 | 4.616776133 |
| GO:0006592 | ornithine biosynthetic process | 1 | 345 | 1 | 16457 | 0.04075791 | 0.3448734 | 47.701449 | 4.616776133 |
| GO:0021941 | negative regulation of cerebellar granule cell precursor proliferation | 1 | 345 | 1 | 16457 | 0.04075791 | 0.3448734 | 47.701449 | 4.616776133 |
| GO:0072619 | interleukin-21 secretion | 1 | 345 | 1 | 16457 | 0.04075791 | 0.3448734 | 47.701449 | 4.616776133 |
| GO:0006014 | D-ribose metabolic process | 1 | 345 | 1 | 16457 | 0.04075791 | 0.3448734 | 47.701449 | 4.616776133 |
| GO:0002030 | inhibitory G-protein coupled receptor phosphorylation | 1 | 345 | 1 | 16457 | 0.04075791 | 0.3448734 | 47.701449 | 4.616776133 |
| GO:0031930 | mitochondria-nucleus signaling pathway | 1 | 345 | 1 | 16457 | 0.04075791 | 0.3448734 | 47.701449 | 4.616776133 |
| GO:0015876 | acetyl-CoA transport | 1 | 345 | 1 | 16457 | 0.04075791 | 0.3448734 | 47.701449 | 4.616776133 |
| GO:0090232 | positive regulation of spindle checkpoint | 1 | 345 | 1 | 16457 | 0.04075791 | 0.3448734 | 47.701449 | 4.616776133 |
| GO:2000659 | regulation of interleukin-1-mediated signaling pathway | 1 | 345 | 1 | 16457 | 0.04075791 | 0.3448734 | 47.701449 | 4.616776133 |
| GO:0090298 | negative regulation of mitochondrial DNA replication | 1 | 345 | 1 | 16457 | 0.04075791 | 0.3448734 | 47.701449 | 4.616776133 |
| GO:0034959 | endothelin maturation | 1 | 345 | 1 | 16457 | 0.04075791 | 0.3448734 | 47.701449 | 4.616776133 |
| GO:0003421 | growth plate cartilage axis specification | 1 | 345 | 1 | 16457 | 0.04075791 | 0.3448734 | 47.701449 | 4.616776133 |
| GO:0002764 | immune response-regulating signaling pathway | 1 | 345 | 1 | 16457 | 0.04075791 | 0.3448734 | 47.701449 | 4.616776133 |
| GO:0072703 | cellular response to methyl methanesulfonate | 1 | 345 | 1 | 16457 | 0.04075791 | 0.3448734 | 47.701449 | 4.616776133 |
| GO:0061308 | cardiac neural crest cell development involved in heart development | 1 | 345 | 1 | 16457 | 0.04075791 | 0.3448734 | 47.701449 | 4.616776133 |
| GO:1901991 | negative regulation of mitotic cell cycle phase transition | 1 | 345 | 1 | 16457 | 0.04075791 | 0.3448734 | 47.701449 | 4.616776133 |
| GO:1900075 | positive regulation of neuromuscular synaptic transmission | 1 | 345 | 1 | 16457 | 0.04075791 | 0.3448734 | 47.701449 | 4.616776133 |
| GO:0043418 | homocysteine catabolic process | 1 | 345 | 1 | 16457 | 0.04075791 | 0.3448734 | 47.701449 | 4.616776133 |
| GO:1990264 | peptidyl-tyrosine dephosphorylation involved in inactivation of protein kinase activity | 1 | 345 | 1 | 16457 | 0.04075791 | 0.3448734 | 47.701449 | 4.616776133 |

| | | | | | | | | | |
|---|---|---|---|---|---|---|---|---|---|
| GO:0050746 | regulation of lipoprotein metabolic process | 1 | 345 | 1 | 16457 | 0.04075791 | 0.3448734 | 47.701449 | 4.616776133 |
| GO:1900424 | regulation of defense response to bacterium | 1 | 345 | 1 | 16457 | 0.04075791 | 0.3448734 | 47.701449 | 4.616776133 |
| GO:0035772 | interleukin-13-mediated signaling pathway | 1 | 345 | 1 | 16457 | 0.04075791 | 0.3448734 | 47.701449 | 4.616776133 |
| GO:0051389 | inactivation of MAPKK activity | 1 | 345 | 1 | 16457 | 0.04075791 | 0.3448734 | 47.701449 | 4.616776133 |
| GO:0010635 | regulation of mitochondrial fusion | 1 | 345 | 1 | 16457 | 0.04075791 | 0.3448734 | 47.701449 | 4.616776133 |
| GO:0002684 | positive regulation of immune system process | 1 | 345 | 1 | 16457 | 0.04075791 | 0.3448734 | 47.701449 | 4.616776133 |
| GO:1902213 | positive regulation of prolactin signaling pathway | 1 | 345 | 1 | 16457 | 0.04075791 | 0.3448734 | 47.701449 | 4.616776133 |
| GO:1990519 | mitochondrial pyrimidine nucleotide import | 1 | 345 | 1 | 16457 | 0.04075791 | 0.3448734 | 47.701449 | 4.616776133 |
| GO:0010813 | neuropeptide catabolic process | 1 | 345 | 1 | 16457 | 0.04075791 | 0.3448734 | 47.701449 | 4.616776133 |
| GO:0010814 | substance P catabolic process | 1 | 345 | 1 | 16457 | 0.04075791 | 0.3448734 | 47.701449 | 4.616776133 |
| GO:0010816 | calcitonin catabolic process | 1 | 345 | 1 | 16457 | 0.04075791 | 0.3448734 | 47.701449 | 4.616776133 |
| GO:0006713 | glucocorticoid catabolic process | 1 | 345 | 1 | 16457 | 0.04075791 | 0.3448734 | 47.701449 | 4.616776133 |
| GO:0046398 | UDP-glucuronate metabolic process | 1 | 345 | 1 | 16457 | 0.04075791 | 0.3448734 | 47.701449 | 4.616776133 |
| GO:0034402 | recruitment of 3'-end processing factors to RNA polymerase II holoenzyme complex | 1 | 345 | 1 | 16457 | 0.04075791 | 0.3448734 | 47.701449 | 4.616776133 |
| GO:0090096 | positive regulation of metanephric cap mesenchymal cell proliferation | 1 | 345 | 1 | 16457 | 0.04075791 | 0.3448734 | 47.701449 | 4.616776133 |
| GO:0019343 | cysteine biosynthetic process via cystathionine | 1 | 345 | 1 | 16457 | 0.04075791 | 0.3448734 | 47.701449 | 4.616776133 |
| GO:1901626 | regulation of postsynaptic membrane organization | 1 | 345 | 1 | 16457 | 0.04075791 | 0.3448734 | 47.701449 | 4.616776133 |
| GO:0000453 | enzyme-directed rRNA 2'-O-methylation | 1 | 345 | 1 | 16457 | 0.04075791 | 0.3448734 | 47.701449 | 4.616776133 |
| GO:1903403 | negative regulation of renal phosphate excretion | 1 | 345 | 1 | 16457 | 0.04075791 | 0.3448734 | 47.701449 | 4.616776133 |
| GO:0098902 | regulation of membrane depolarization during action potential | 1 | 345 | 1 | 16457 | 0.04075791 | 0.3448734 | 47.701449 | 4.616776133 |
| GO:0006238 | CMP salvage | 1 | 345 | 1 | 16457 | 0.04075791 | 0.3448734 | 47.701449 | 4.616776133 |
| GO:0032222 | regulation of synaptic transmission, cholinergic | 1 | 345 | 1 | 16457 | 0.04075791 | 0.3448734 | 47.701449 | 4.616776133 |
| GO:0097581 | lamellipodium organization | 1 | 345 | 1 | 16457 | 0.04075791 | 0.3448734 | 47.701449 | 4.616776133 |
| GO:1990144 | intrinsic apoptotic signaling pathway in response to hypoxia | 1 | 345 | 1 | 16457 | 0.04075791 | 0.3448734 | 47.701449 | 4.616776133 |
| GO:0051610 | serotonin uptake | 1 | 345 | 1 | 16457 | 0.04075791 | 0.3448734 | 47.701449 | 4.616776133 |
| GO:1901256 | regulation of macrophage colony-stimulating factor production | 1 | 345 | 1 | 16457 | 0.04075791 | 0.3448734 | 47.701449 | 4.616776133 |
| GO:2001182 | regulation of interleukin-12 secretion | 1 | 345 | 1 | 16457 | 0.04075791 | 0.3448734 | 47.701449 | 4.616776133 |
| GO:1990166 | protein localization to site of double-strand break | 1 | 345 | 1 | 16457 | 0.04075791 | 0.3448734 | 47.701449 | 4.616776133 |
| GO:0045732 | positive regulation of protein catabolic process | 4 | 345 | 59 | 16457 | 0.041373183 | 0.3448734 | 3.2339966 | 4.595160243 |
| GO:0033280 | response to vitamin D | 2 | 345 | 14 | 16457 | 0.042098442 | 0.3448734 | 6.8144928 | 4.570089359 |
| GO:0030308 | negative regulation of cell growth | 6 | 345 | 118 | 16457 | 0.044517418 | 0.3448734 | 2.4254974 | 4.489486257 |
| GO:0006950 | response to stress | 4 | 345 | 61 | 16457 | 0.045547242 | 0.3448734 | 3.1279639 | 4.456492477 |

| | | | | | | | | | |
|---|---|---|---|---|---|---|---|---|---|
| GO:0016032 | viral process | 23 | 345 | 729 | 16457 | 0.045669406 | 0.3448734 | 1.504984 | 4.452628154 |
| GO:0086010 | membrane depolarization during action potential | 3 | 345 | 36 | 16457 | 0.046255167 | 0.3448734 | 3.9751208 | 4.434241651 |
| GO:0006596 | polyamine biosynthetic process | 2 | 345 | 15 | 16457 | 0.047069693 | 0.3448734 | 6.3601932 | 4.409057758 |
| GO:0045429 | positive regulation of nitric oxide biosynthetic process | 3 | 345 | 37 | 16457 | 0.049259326 | 0.3448734 | 3.8676851 | 4.343459295 |
| **Molecular function category** | | | | | | | | | |
| **GOID** | **GOTerm** | **DifGene** | **AllDifGene** | **GeneInGO** | **AllGene** | **P-Value** | **FDR** | **Enrichment** | **(-log2P)** |
| GO:0044822 | poly(A) RNA binding | 54 | 336 | 1302 | 16096 | 1.37472E-05 | 0.007396 | 1.9868334 | 16.15049773 |
| GO:0003723 | RNA binding | 34 | 336 | 860 | 16096 | 0.000827255 | 0.1471504 | 1.8939092 | 10.23938075 |
| GO:0005515 | protein binding | 181 | 336 | 6561 | 16096 | 0.001852958 | 0.1471504 | 1.321561 | 9.075954208 |
| GO:0003727 | single-stranded RNA binding | 5 | 336 | 39 | 16096 | 0.00206539 | 0.1471504 | 6.1416361 | 8.919370345 |
| GO:0043021 | ribonucleoprotein complex binding | 4 | 336 | 23 | 16096 | 0.002156416 | 0.1471504 | 8.3312629 | 8.857148562 |
| GO:0045503 | dynein light chain binding | 2 | 336 | 2 | 16096 | 0.002461623 | 0.1471504 | 47.904762 | 8.666174363 |
| GO:0016768 | spermine synthase activity | 2 | 336 | 2 | 16096 | 0.002461623 | 0.1471504 | 47.904762 | 8.666174363 |
| GO:0072572 | poly-ADP-D-ribose binding | 2 | 336 | 2 | 16096 | 0.002461623 | 0.1471504 | 47.904762 | 8.666174363 |
| GO:0016422 | mRNA (2'-O-methyladenosine-N6-)-methyltransferase activity | 2 | 336 | 2 | 16096 | 0.002461623 | 0.1471504 | 47.904762 | 8.666174363 |
| GO:0004766 | spermidine synthase activity | 2 | 336 | 3 | 16096 | 0.00404649 | 0.1979102 | 31.936508 | 7.949113111 |
| GO:0004356 | glutamate-ammonia ligase activity | 2 | 336 | 3 | 16096 | 0.00404649 | 0.1979102 | 31.936508 | 7.949113111 |
| GO:0070888 | E-box binding | 4 | 336 | 29 | 16096 | 0.004556788 | 0.204296 | 6.6075534 | 7.77776707 |
| GO:0005159 | insulin-like growth factor receptor binding | 3 | 336 | 15 | 16096 | 0.005625294 | 0.2328006 | 9.5809524 | 7.473855699 |
| GO:0008199 | ferric iron binding | 3 | 336 | 16 | 16096 | 0.006578143 | 0.2527887 | 8.9821429 | 7.248103826 |
| GO:0043559 | insulin binding | 2 | 336 | 5 | 16096 | 0.00826698 | 0.2573258 | 19.161905 | 6.918423953 |
| GO:0004351 | glutamate decarboxylase activity | 2 | 336 | 5 | 16096 | 0.00826698 | 0.2573258 | 19.161905 | 6.918423953 |
| GO:0008408 | 3'-5' exonuclease activity | 3 | 336 | 18 | 16096 | 0.00875596 | 0.2573258 | 7.984127 | 6.835518962 |
| GO:0031369 | translation initiation factor binding | 3 | 336 | 20 | 16096 | 0.011307716 | 0.2573258 | 7.1857143 | 6.466548675 |
| GO:0016787 | hydrolase activity | 51 | 336 | 1689 | 16096 | 0.011883791 | 0.2573258 | 1.4465026 | 6.39486101 |
| GO:0003824 | catalytic activity | 25 | 336 | 727 | 16096 | 0.016234182 | 0.2573258 | 1.6473439 | 5.944821535 |
| GO:0003857 | 3-hydroxyacyl-CoA dehydrogenase activity | 2 | 336 | 8 | 16096 | 0.017001832 | 0.2573258 | 11.97619 | 5.878165959 |
| GO:0008060 | ARF GTPase activator activity | 3 | 336 | 24 | 16096 | 0.017570587 | 0.2573258 | 5.9880952 | 5.830693803 |
| GO:0030515 | snoRNA binding | 3 | 336 | 24 | 16096 | 0.017570587 | 0.2573258 | 5.9880952 | 5.830693803 |
| GO:0003697 | single-stranded DNA binding | 6 | 336 | 94 | 16096 | 0.017625911 | 0.2573258 | 3.0577508 | 5.826158377 |
| GO:0003729 | mRNA binding | 6 | 336 | 95 | 16096 | 0.01841947 | 0.2573258 | 3.0255639 | 5.762624669 |
| GO:0016740 | transferase activity | 45 | 336 | 1507 | 16096 | 0.019311644 | 0.2573258 | 1.4304673 | 5.694385224 |

| | | | | | | | | | |
|---|---|---|---|---|---|---|---|---|---|
| GO:0031994 | insulin-like growth factor I binding | 2 | 336 | 9 | 16096 | 0.020498391 | 0.2573258 | 10.645503 | 5.608345543 |
| GO:0016790 | thiolester hydrolase activity | 2 | 336 | 10 | 16096 | 0.024265314 | 0.2573258 | 9.5809524 | 5.364960687 |
| GO:0043522 | leucine zipper domain binding | 2 | 336 | 10 | 16096 | 0.024265314 | 0.2573258 | 9.5809524 | 5.364960687 |
| GO:0004322 | ferroxidase activity | 2 | 336 | 10 | 16096 | 0.024265314 | 0.2573258 | 9.5809524 | 5.364960687 |
| GO:0017080 | sodium channel regulator activity | 3 | 336 | 28 | 16096 | 0.02541107 | 0.2573258 | 5.1326531 | 5.298399075 |
| GO:0035259 | glucocorticoid receptor binding | 2 | 336 | 11 | 16096 | 0.028289973 | 0.2573258 | 8.7099567 | 5.143565402 |
| GO:0004861 | cyclin-dependent protein serine/threonine kinase inhibitor activity | 2 | 336 | 11 | 16096 | 0.028289973 | 0.2573258 | 8.7099567 | 5.143565402 |
| GO:0001047 | core promoter binding | 4 | 336 | 53 | 16096 | 0.0298267 | 0.2573258 | 3.6154537 | 5.067251824 |
| GO:0001227 | RNA polymerase II transcription regulatory region sequence-specific DNA binding transcription factor activity involved in negative regulation of transcription | 3 | 336 | 31 | 16096 | 0.032329193 | 0.2573258 | 4.6359447 | 4.951018716 |
| GO:0043560 | insulin receptor substrate binding | 2 | 336 | 12 | 16096 | 0.032560147 | 0.2573258 | 7.984127 | 4.94074898 |
| GO:0016595 | glutamate binding | 2 | 336 | 12 | 16096 | 0.032560147 | 0.2573258 | 7.984127 | 4.94074898 |
| GO:0070491 | repressing transcription factor binding | 3 | 336 | 32 | 16096 | 0.034831003 | 0.2573258 | 4.4910714 | 4.843484178 |
| GO:0001784 | phosphotyrosine binding | 2 | 336 | 13 | 16096 | 0.03706401 | 0.2573258 | 7.3699634 | 4.753837204 |
| GO:0030108 | HLA-A specific activating MHC class I receptor activity | 1 | 336 | 1 | 16096 | 0.04059325 | 0.2573258 | 47.904762 | 4.62261633 |
| GO:0010859 | calcium-dependent cysteine-type endopeptidase inhibitor activity | 1 | 336 | 1 | 16096 | 0.04059325 | 0.2573258 | 47.904762 | 4.62261633 |
| GO:0004655 | porphobilinogen synthase activity | 1 | 336 | 1 | 16096 | 0.04059325 | 0.2573258 | 47.904762 | 4.62261633 |
| GO:0050104 | L-gulonate 3-dehydrogenase activity | 1 | 336 | 1 | 16096 | 0.04059325 | 0.2573258 | 47.904762 | 4.62261633 |
| GO:0019912 | cyclin-dependent protein kinase activating kinase activity | 1 | 336 | 1 | 16096 | 0.04059325 | 0.2573258 | 47.904762 | 4.62261633 |
| GO:0019811 | cocaine binding | 1 | 336 | 1 | 16096 | 0.04059325 | 0.2573258 | 47.904762 | 4.62261633 |
| GO:0032791 | lead ion binding | 1 | 336 | 1 | 16096 | 0.04059325 | 0.2573258 | 47.904762 | 4.62261633 |
| GO:0042801 | polo kinase kinase activity | 1 | 336 | 1 | 16096 | 0.04059325 | 0.2573258 | 47.904762 | 4.62261633 |
| GO:0008955 | peptidoglycan glycosyltransferase activity | 1 | 336 | 1 | 16096 | 0.04059325 | 0.2573258 | 47.904762 | 4.62261633 |
| GO:0047693 | ATP diphosphatase activity | 1 | 336 | 1 | 16096 | 0.04059325 | 0.2573258 | 47.904762 | 4.62261633 |
| GO:0052925 | dol-P-Man:Man(5)GlcNAc(2)-PP-Dol alpha-1,3-mannosyltransferase activity | 1 | 336 | 1 | 16096 | 0.04059325 | 0.2573258 | 47.904762 | 4.62261633 |
| GO:0005009 | insulin-activated receptor activity | 1 | 336 | 1 | 16096 | 0.04059325 | 0.2573258 | 47.904762 | 4.62261633 |
| GO:0003983 | UTP:glucose-1-phosphate uridylyltransferase activity | 1 | 336 | 1 | 16096 | 0.04059325 | 0.2573258 | 47.904762 | 4.62261633 |
| GO:0008521 | acetyl-CoA transporter activity | 1 | 336 | 1 | 16096 | 0.04059325 | 0.2573258 | 47.904762 | 4.62261633 |
| GO:0019778 | Atg12 activating enzyme activity | 1 | 336 | 1 | 16096 | 0.04059325 | 0.2573258 | 47.904762 | 4.62261633 |
| GO:0019779 | Atg8 activating enzyme activity | 1 | 336 | 1 | 16096 | 0.04059325 | 0.2573258 | 47.904762 | 4.62261633 |
| GO:0046570 | methylthioribulose 1-phosphate dehydratase activity | 1 | 336 | 1 | 16096 | 0.04059325 | 0.2573258 | 47.904762 | 4.62261633 |
| GO:0002135 | CTP binding | 1 | 336 | 1 | 16096 | 0.04059325 | 0.2573258 | 47.904762 | 4.62261633 |
| GO:0008445 | D-aspartate oxidase activity | 1 | 336 | 1 | 16096 | 0.04059325 | 0.2573258 | 47.904762 | 4.62261633 |

| | | | | | | | | | |
|---|---|---|---|---|---|---|---|---|---|
| GO:0016428 | tRNA (cytosine-5-)-methyltransferase activity | 1 | 336 | 1 | 16096 | 0.04059325 | 0.2573258 | 47.904762 | 4.62261633 |
| GO:0032557 | pyrimidine ribonucleotide binding | 1 | 336 | 1 | 16096 | 0.04059325 | 0.2573258 | 47.904762 | 4.62261633 |
| GO:0052590 | sn-glycerol-3-phosphate:ubiquinone oxidoreductase activity | 1 | 336 | 1 | 16096 | 0.04059325 | 0.2573258 | 47.904762 | 4.62261633 |
| GO:0052591 | sn-glycerol-3-phosphate:ubiquinone-8 oxidoreductase activity | 1 | 336 | 1 | 16096 | 0.04059325 | 0.2573258 | 47.904762 | 4.62261633 |
| GO:0070551 | endoribonuclease activity, cleaving siRNA-paired mRNA | 1 | 336 | 1 | 16096 | 0.04059325 | 0.2573258 | 47.904762 | 4.62261633 |
| GO:0016515 | interleukin-13 receptor activity | 1 | 336 | 1 | 16096 | 0.04059325 | 0.2573258 | 47.904762 | 4.62261633 |
| GO:0005335 | serotonin:sodium symporter activity | 1 | 336 | 1 | 16096 | 0.04059325 | 0.2573258 | 47.904762 | 4.62261633 |
| GO:0004061 | arylformamidase activity | 1 | 336 | 1 | 16096 | 0.04059325 | 0.2573258 | 47.904762 | 4.62261633 |
| GO:0036219 | GTP diphosphatase activity | 1 | 336 | 1 | 16096 | 0.04059325 | 0.2573258 | 47.904762 | 4.62261633 |
| GO:0004747 | ribokinase activity | 1 | 336 | 1 | 16096 | 0.04059325 | 0.2573258 | 47.904762 | 4.62261633 |
| GO:0004122 | cystathionine beta-synthase activity | 1 | 336 | 1 | 16096 | 0.04059325 | 0.2573258 | 47.904762 | 4.62261633 |
| GO:0004132 | dCMP deaminase activity | 1 | 336 | 1 | 16096 | 0.04059325 | 0.2573258 | 47.904762 | 4.62261633 |
| GO:0004349 | glutamate 5-kinase activity | 1 | 336 | 1 | 16096 | 0.04059325 | 0.2573258 | 47.904762 | 4.62261633 |
| GO:0004350 | glutamate-5-semialdehyde dehydrogenase activity | 1 | 336 | 1 | 16096 | 0.04059325 | 0.2573258 | 47.904762 | 4.62261633 |
| GO:0052798 | beta-galactoside alpha-2,3-sialyltransferase activity | 1 | 336 | 1 | 16096 | 0.04059325 | 0.2573258 | 47.904762 | 4.62261633 |
| GO:0047277 | globoside alpha-N-acetylgalactosaminyltransferase activity | 1 | 336 | 1 | 16096 | 0.04059325 | 0.2573258 | 47.904762 | 4.62261633 |
| GO:0030294 | receptor signaling protein tyrosine kinase inhibitor activity | 1 | 336 | 1 | 16096 | 0.04059325 | 0.2573258 | 47.904762 | 4.62261633 |
| GO:0047273 | galactosylgalactosylglucosylceramide beta-D-acetylgalactosaminyltransferase activity | 1 | 336 | 1 | 16096 | 0.04059325 | 0.2573258 | 47.904762 | 4.62261633 |
| GO:0004089 | carbonate dehydratase activity | 2 | 336 | 14 | 16096 | 0.041790121 | 0.2573258 | 6.8435374 | 4.580694249 |
| GO:0030971 | receptor tyrosine kinase binding | 3 | 336 | 36 | 16096 | 0.04579834 | 0.2573258 | 3.9920635 | 4.448560884 |
| GO:0016829 | lyase activity | 7 | 336 | 151 | 16096 | 0.046130859 | 0.2573258 | 2.2207506 | 4.438124045 |
| GO:0003713 | transcription coactivator activity | 10 | 336 | 254 | 16096 | 0.049008178 | 0.2573258 | 1.8860142 | 4.350833687 |
| GO:0046872 | metal ion binding | 87 | 336 | 3374 | 16096 | 0.049354185 | 0.2573258 | 1.2352443 | 4.340683778 |
| GO:0042803 | protein homodimerization activity | 22 | 336 | 700 | 16096 | 0.04944924 | 0.2573258 | 1.5055782 | 4.337907855 |
| **Cellular component category** | | | | | | | | | |
| **GOID** | **GOTerm** | **DifGene** | **AllDifGene** | **GeneInGO** | **AllGene** | **P-Value** | **FDR** | **Enrichment** | **(-log2P)** |
| GO:0005730 | nucleolus | 77 | 362 | 1944 | 17318 | 1.81969E-06 | 0.0005586 | 1.8948884 | 19.06787531 |
| GO:0005634 | nucleus | 177 | 362 | 6067 | 17318 | 0.000253429 | 0.0389013 | 1.3956883 | 11.94613296 |
| GO:0005829 | cytosol | 95 | 362 | 3053 | 17318 | 0.000621489 | 0.063599 | 1.4886273 | 10.65198362 |
| GO:0019013 | viral nucleocapsid | 6 | 362 | 52 | 17318 | 0.001248202 | 0.0957995 | 5.5199745 | 9.645932421 |
| GO:0030529 | ribonucleoprotein complex | 21 | 362 | 471 | 17318 | 0.001852223 | 0.0961032 | 2.1329838 | 9.076526599 |
| GO:0071013 | catalytic step 2 spliceosome | 8 | 362 | 100 | 17318 | 0.001878239 | 0.0961032 | 3.8271823 | 9.056403715 |

| | | | | | | | | | |
|---|---|---|---|---|---|---|---|---|---|
| GO:0070545 | PeBoW complex | 2 | 362 | 3 | 17318 | 0.004054609 | 0.1275121 | 31.893186 | 7.946221439 |
| GO:0036396 | MIS complex | 2 | 362 | 3 | 17318 | 0.004054609 | 0.1275121 | 31.893186 | 7.946221439 |
| GO:0005654 | nucleoplasm | 78 | 362 | 2619 | 17318 | 0.00407534 | 0.1275121 | 1.4247815 | 7.938863971 |
| GO:0031362 | anchored component of external side of plasma membrane | 3 | 362 | 15 | 17318 | 0.005640043 | 0.1275121 | 9.5679558 | 7.470078094 |
| GO:0030687 | preribosome, large subunit precursor | 2 | 362 | 4 | 17318 | 0.005998671 | 0.1275121 | 23.91989 | 7.381141397 |
| GO:0032777 | Piccolo NuA4 histone acetyltransferase complex | 2 | 362 | 4 | 17318 | 0.005998671 | 0.1275121 | 23.91989 | 7.381141397 |
| GO:0097386 | glial cell projection | 2 | 362 | 4 | 17318 | 0.005998671 | 0.1275121 | 23.91989 | 7.381141397 |
| GO:0015934 | large ribosomal subunit | 4 | 362 | 32 | 17318 | 0.006264951 | 0.1275121 | 5.9799724 | 7.318481115 |
| GO:0035267 | NuA4 histone acetyltransferase complex | 3 | 362 | 16 | 17318 | 0.006595335 | 0.1275121 | 8.9699586 | 7.244338359 |
| GO:0022625 | cytosolic large ribosomal subunit | 8 | 362 | 125 | 17318 | 0.006645583 | 0.1275121 | 3.0617459 | 7.233388536 |
| GO:0005737 | cytoplasm | 166 | 362 | 6352 | 17318 | 0.011302323 | 0.2041067 | 1.2502209 | 6.467236822 |
| GO:0008043 | intracellular ferritin complex | 2 | 362 | 7 | 17318 | 0.013815804 | 0.2356362 | 13.668508 | 6.177536712 |
| GO:0005791 | rough endoplasmic reticulum | 4 | 362 | 43 | 17318 | 0.015866009 | 0.2522103 | 4.450212 | 5.977916946 |
| GO:0043231 | intracellular membrane-bounded organelle | 23 | 362 | 656 | 17318 | 0.016798508 | 0.2522103 | 1.6773093 | 5.89552308 |
| GO:0000407 | pre-autophagosomal structure | 3 | 362 | 24 | 17318 | 0.017615302 | 0.2522103 | 5.9799724 | 5.827026989 |
| GO:0032040 | small-subunit processome | 3 | 362 | 25 | 17318 | 0.019431236 | 0.2522103 | 5.7407735 | 5.685478506 |
| GO:0016604 | nuclear body | 4 | 362 | 46 | 17318 | 0.019517153 | 0.2522103 | 4.1599808 | 5.679113584 |
| GO:0000812 | Swr1 complex | 2 | 362 | 9 | 17318 | 0.020538298 | 0.2522103 | 10.631062 | 5.605539578 |
| GO:0000307 | cyclin-dependent protein kinase holoenzyme complex | 2 | 362 | 9 | 17318 | 0.020538298 | 0.2522103 | 10.631062 | 5.605539578 |
| GO:0022626 | cytosolic ribosome | 2 | 362 | 10 | 17318 | 0.024312313 | 0.2870723 | 9.5679558 | 5.362169037 |
| GO:0031594 | neuromuscular junction | 4 | 362 | 52 | 17318 | 0.02826384 | 0.3172236 | 3.679983 | 5.144898683 |
| GO:0071339 | MLL1 complex | 3 | 362 | 30 | 17318 | 0.029999487 | 0.3172236 | 4.7839779 | 5.058918367 |
| GO:0005681 | spliceosomal complex | 8 | 362 | 168 | 17318 | 0.030566625 | 0.3172236 | 2.2780847 | 5.03189891 |
| GO:0070062 | extracellular vesicular exosome | 80 | 362 | 2993 | 17318 | 0.030999048 | 0.3172236 | 1.2787111 | 5.011632295 |
| GO:0017119 | Golgi transport complex | 2 | 362 | 12 | 17318 | 0.032622565 | 0.3224168 | 7.9732965 | 4.93798598 |
| GO:0032039 | integrator complex | 2 | 362 | 13 | 17318 | 0.037134693 | 0.3224168 | 7.359966 | 4.751088537 |
| GO:0005898 | interleukin-13 receptor complex | 1 | 362 | 1 | 17318 | 0.040638387 | 0.3224168 | 47.839779 | 4.621013059 |
| GO:0031302 | intrinsic component of endosome membrane | 1 | 362 | 1 | 17318 | 0.040638387 | 0.3224168 | 47.839779 | 4.621013059 |
| GO:0044195 | nucleoplasmic reticulum | 1 | 362 | 1 | 17318 | 0.040638387 | 0.3224168 | 47.839779 | 4.621013059 |
| GO:0019898 | extrinsic component of membrane | 5 | 362 | 87 | 17318 | 0.041857049 | 0.3224168 | 2.7494126 | 4.578385585 |
| GO:0005916 | fascia adherens | 2 | 362 | 14 | 17318 | 0.041869401 | 0.3224168 | 6.8342541 | 4.577959919 |
| GO:0031011 | Ino80 complex | 2 | 362 | 14 | 17318 | 0.041869401 | 0.3224168 | 6.8342541 | 4.577959919 |

| | | | | | | | | | |
|---|---|---|---|---|---|---|---|---|---|
| GO:0005844 | polysome | 3 | 362 | 35 | 17318 | 0.043019294 | 0.3224168 | 4.1005525 | 4.538872325 |
| GO:0016592 | mediator complex | 3 | 362 | 35 | 17318 | 0.043019294 | 0.3224168 | 4.1005525 | 4.538872325 |
| GO:0048471 | perinuclear region of cytoplasm | 19 | 362 | 574 | 17318 | 0.04305892 | 0.3224168 | 1.5835467 | 4.537544066 |
| GO:0030018 | Z disc | 6 | 362 | 119 | 17318 | 0.045406136 | 0.3270609 | 2.4120897 | 4.460968913 |
| GO:0000139 | Golgi membrane | 18 | 362 | 541 | 17318 | 0.045895475 | 0.3270609 | 1.5917117 | 4.445504268 |
| GO:0000776 | kinetochore | 6 | 362 | 120 | 17318 | 0.046875174 | 0.3270609 | 2.391989 | 4.415032136 |

## Table S4: GO-analysis of down-regulated DEGs (STH vs QHMM)

| Biological process category | | | | | | | | | |
|---|---|---|---|---|---|---|---|---|---|
| **GOID** | **GO Term** | **DifGene** | **AllDifGene** | **GeneInGO** | **AllGene** | **P-Value** | **FDR** | **Enrichment** | **(-log2P)** |
| GO:0044255 | cellular lipid metabolic process | 18 | 484 | 156 | 16457 | 3.74152E-06 | 0.007362256 | 3.9233153 | 18.02794537 |
| GO:0044281 | small molecule metabolic process | 79 | 484 | 1500 | 16457 | 6.91616E-06 | 0.007362256 | 1.7907755 | 17.14159664 |
| GO:0006006 | glucose metabolic process | 14 | 484 | 124 | 16457 | 5.17045E-05 | 0.023172809 | 3.8389429 | 14.23934961 |
| GO:0046676 | negative regulation of insulin secretion | 7 | 484 | 28 | 16457 | 5.40113E-05 | 0.023172809 | 8.5005165 | 14.17637844 |
| GO:0030049 | muscle filament sliding | 8 | 484 | 39 | 16457 | 5.44218E-05 | 0.023172809 | 6.9747828 | 14.16545538 |
| GO:0006099 | tricarboxylic acid cycle | 7 | 484 | 32 | 16457 | 0.000111627 | 0.039608929 | 7.437952 | 13.12902824 |
| GO:0002027 | regulation of heart rate | 7 | 484 | 34 | 16457 | 0.000155109 | 0.047175212 | 7.0004254 | 12.6544325 |
| GO:0048741 | skeletal muscle fiber development | 6 | 484 | 24 | 16457 | 0.000184933 | 0.049215286 | 8.5005165 | 12.40070995 |
| GO:0005975 | carbohydrate metabolic process | 28 | 484 | 428 | 16457 | 0.000212632 | 0.050299352 | 2.2244342 | 12.1993516 |
| GO:0055114 | oxidation-reduction process | 43 | 484 | 784 | 16457 | 0.000260641 | 0.055490543 | 1.8649092 | 11.90564641 |
| GO:0035567 | non-canonical Wnt signaling pathway | 5 | 484 | 18 | 16457 | 0.000426712 | 0.082588144 | 9.4450184 | 11.19445011 |
| GO:0006094 | gluconeogenesis | 7 | 484 | 42 | 16457 | 0.000484289 | 0.085921009 | 5.667011 | 11.01184298 |
| GO:0008016 | regulation of heart contraction | 7 | 484 | 43 | 16457 | 0.000549126 | 0.089929981 | 5.5352201 | 10.83057455 |
| GO:0006936 | muscle contraction | 11 | 484 | 108 | 16457 | 0.000689725 | 0.095613229 | 3.4631734 | 10.5016906 |
| GO:0006768 | biotin metabolic process | 4 | 484 | 11 | 16457 | 0.000718559 | 0.095613229 | 12.364388 | 10.44260618 |
| GO:0071395 | cellular response to jasmonic acid stimulus | 4 | 484 | 11 | 16457 | 0.000718559 | 0.095613229 | 12.364388 | 10.44260618 |
| GO:0045214 | sarcomere organization | 6 | 484 | 33 | 16457 | 0.000813554 | 0.101885726 | 6.1821938 | 10.26347354 |

| | | | | | | | | | |
|---|---|---|---|---|---|---|---|---|---|
| GO:0009725 | response to hormone | 8 | 484 | 62 | 16457 | 0.000905966 | 0.107155603 | 4.3873634 | 10.10825604 |
| GO:0006089 | lactate metabolic process | 3 | 484 | 5 | 16457 | 0.00118537 | 0.121290344 | 20.40124 | 9.720446291 |
| GO:0032868 | response to insulin | 8 | 484 | 65 | 16457 | 0.001195081 | 0.121290344 | 4.1848697 | 9.708676271 |
| GO:0090179 | planar cell polarity pathway involved in neural tube closure | 4 | 484 | 13 | 16457 | 0.001196382 | 0.121290344 | 10.462174 | 9.707106207 |
| GO:0044237 | cellular metabolic process | 14 | 484 | 175 | 16457 | 0.001289868 | 0.124824028 | 2.7201653 | 9.598561038 |
| GO:0005977 | glycogen metabolic process | 6 | 484 | 37 | 16457 | 0.001377732 | 0.127530049 | 5.5138486 | 9.503489153 |
| GO:0055010 | ventricular cardiac muscle tissue morphogenesis | 5 | 484 | 25 | 16457 | 0.001527895 | 0.135537028 | 6.8004132 | 9.354238784 |
| GO:0044598 | doxorubicin metabolic process | 4 | 484 | 15 | 16457 | 0.001860736 | 0.136280795 | 9.0672176 | 9.069911202 |
| GO:0044597 | daunorubicin metabolic process | 4 | 484 | 15 | 16457 | 0.001860736 | 0.136280795 | 9.0672176 | 9.069911202 |
| GO:0006635 | fatty acid beta-oxidation | 6 | 484 | 41 | 16457 | 0.002199784 | 0.136280795 | 4.9759121 | 8.828422166 |
| GO:0006629 | lipid metabolic process | 29 | 484 | 530 | 16457 | 0.002227958 | 0.136280795 | 1.8604904 | 8.810062248 |
| GO:0019674 | NAD metabolic process | 4 | 484 | 16 | 16457 | 0.002273085 | 0.136280795 | 8.5005165 | 8.781132946 |
| GO:0007405 | neuroblast proliferation | 4 | 484 | 16 | 16457 | 0.002273085 | 0.136280795 | 8.5005165 | 8.781132946 |
| GO:0007275 | multicellular organismal development | 49 | 484 | 1041 | 16457 | 0.00228149 | 0.136280795 | 1.6004815 | 8.77580799 |
| GO:0006090 | pyruvate metabolic process | 5 | 484 | 28 | 16457 | 0.002368722 | 0.136280795 | 6.0717975 | 8.721675386 |
| GO:0001937 | negative regulation of endothelial cell proliferation | 5 | 484 | 28 | 16457 | 0.002368722 | 0.136280795 | 6.0717975 | 8.721675386 |
| GO:0005978 | glycogen biosynthetic process | 5 | 484 | 28 | 16457 | 0.002368722 | 0.136280795 | 6.0717975 | 8.721675386 |
| GO:0008406 | gonad development | 5 | 484 | 28 | 16457 | 0.002368722 | 0.136280795 | 6.0717975 | 8.721675386 |
| GO:0010694 | positive regulation of alkaline phosphatase activity | 3 | 484 | 7 | 16457 | 0.002432443 | 0.136280795 | 14.572314 | 8.683378545 |
| GO:0043462 | regulation of ATPase activity | 3 | 484 | 7 | 16457 | 0.002432443 | 0.136280795 | 14.572314 | 8.683378545 |
| GO:0030913 | paranodal junction assembly | 3 | 484 | 7 | 16457 | 0.002432443 | 0.136280795 | 14.572314 | 8.683378545 |
| GO:0032869 | cellular response to insulin stimulus | 8 | 484 | 75 | 16457 | 0.002720296 | 0.146060866 | 3.6268871 | 8.522020775 |
| GO:0006103 | 2-oxoglutarate metabolic process | 4 | 484 | 17 | 16457 | 0.002744215 | 0.146060866 | 8.0004861 | 8.509390547 |
| GO:0051260 | protein homooligomerization | 13 | 484 | 173 | 16457 | 0.003103447 | 0.161152174 | 2.5550686 | 8.331912672 |
| GO:0060070 | canonical Wnt signaling pathway | 8 | 484 | 79 | 16457 | 0.003642092 | 0.1846194 | 3.4432472 | 8.101016746 |
| GO:0006631 | fatty acid metabolic process | 12 | 484 | 157 | 16457 | 0.003851375 | 0.190687869 | 2.598884 | 8.020410504 |
| GO:0060416 | response to growth hormone | 3 | 484 | 9 | 16457 | 0.004271094 | 0.20595401 | 11.334022 | 7.871178762 |
| GO:0006941 | striated muscle contraction | 4 | 484 | 20 | 16457 | 0.004548719 | 0.20595401 | 6.8004132 | 7.780324046 |
| GO:0006097 | glyoxylate cycle | 2 | 484 | 2 | 16457 | 0.004740135 | 0.20595401 | 34.002066 | 7.72085627 |

| | | | | | | | | | |
|---|---|---|---|---|---|---|---|---|---|
| GO:0031444 | slow-twitch skeletal muscle fiber contraction | 2 | 484 | 2 | 16457 | 0.004740135 | 0.20595401 | 34.002066 | 7.72085627 |
| GO:0071954 | chemokine (C-C motif) ligand 11 production | 2 | 484 | 2 | 16457 | 0.004740135 | 0.20595401 | 34.002066 | 7.72085627 |
| GO:0007522 | visceral muscle development | 2 | 484 | 2 | 16457 | 0.004740135 | 0.20595401 | 34.002066 | 7.72085627 |
| GO:0030178 | negative regulation of Wnt signaling pathway | 6 | 484 | 49 | 16457 | 0.004884891 | 0.207998645 | 4.1635183 | 7.677458007 |
| GO:0061053 | somite development | 3 | 484 | 10 | 16457 | 0.005434162 | 0.222487119 | 10.20062 | 7.5237268 |
| GO:0035413 | positive regulation of catenin import into nucleus | 3 | 484 | 10 | 16457 | 0.005434162 | 0.222487119 | 10.20062 | 7.5237268 |
| GO:0009058 | biosynthetic process | 6 | 484 | 51 | 16457 | 0.00582424 | 0.233958619 | 4.0002431 | 7.423714499 |
| GO:0010881 | regulation of cardiac muscle contraction by regulation of the release of sequestered calcium ion | 4 | 484 | 22 | 16457 | 0.006114039 | 0.23967888 | 6.1821938 | 7.353658518 |
| GO:0006767 | water-soluble vitamin metabolic process | 8 | 484 | 87 | 16457 | 0.006191798 | 0.23967888 | 3.1266268 | 7.335425827 |
| GO:0006633 | fatty acid biosynthetic process | 7 | 484 | 70 | 16457 | 0.006736519 | 0.242630882 | 3.4002066 | 7.213780993 |
| GO:0071377 | cellular response to glucagon stimulus | 5 | 484 | 37 | 16457 | 0.006856079 | 0.242630882 | 4.5948738 | 7.188400546 |
| GO:0035690 | cellular response to drug | 6 | 484 | 53 | 16457 | 0.006888635 | 0.242630882 | 3.8492905 | 7.181566138 |
| GO:0001944 | vasculature development | 5 | 484 | 38 | 16457 | 0.007576914 | 0.242630882 | 4.4739561 | 7.044173877 |
| GO:0006749 | glutathione metabolic process | 5 | 484 | 38 | 16457 | 0.007576914 | 0.242630882 | 4.4739561 | 7.044173877 |
| GO:0006470 | protein dephosphorylation | 11 | 484 | 151 | 16457 | 0.007593151 | 0.242630882 | 2.4769717 | 7.041085519 |
| GO:0045726 | positive regulation of integrin biosynthetic process | 2 | 484 | 3 | 16457 | 0.007749601 | 0.242630882 | 22.668044 | 7.011662302 |
| GO:2000041 | negative regulation of planar cell polarity pathway involved in axis elongation | 2 | 484 | 3 | 16457 | 0.007749601 | 0.242630882 | 22.668044 | 7.011662302 |
| GO:0046498 | S-adenosylhomocysteine metabolic process | 2 | 484 | 3 | 16457 | 0.007749601 | 0.242630882 | 22.668044 | 7.011662302 |
| GO:0060420 | regulation of heart growth | 2 | 484 | 3 | 16457 | 0.007749601 | 0.242630882 | 22.668044 | 7.011662302 |
| GO:0071400 | cellular response to oleic acid | 2 | 484 | 3 | 16457 | 0.007749601 | 0.242630882 | 22.668044 | 7.011662302 |
| GO:0003179 | heart valve morphogenesis | 2 | 484 | 3 | 16457 | 0.007749601 | 0.242630882 | 22.668044 | 7.011662302 |
| GO:0061056 | sclerotome development | 2 | 484 | 3 | 16457 | 0.007749601 | 0.242630882 | 22.668044 | 7.011662302 |
| GO:0007519 | skeletal muscle tissue development | 6 | 484 | 55 | 16457 | 0.00808691 | 0.249522196 | 3.7093163 | 6.950195719 |
| GO:0010867 | positive regulation of triglyceride biosynthetic process | 3 | 484 | 12 | 16457 | 0.008281779 | 0.250345 | 8.5005165 | 6.915843647 |
| GO:0007528 | neuromuscular junction development | 5 | 484 | 39 | 16457 | 0.008348753 | 0.250345 | 4.3592392 | 6.90422357 |
| GO:0060412 | ventricular septum morphogenesis | 4 | 484 | 25 | 16457 | 0.009072102 | 0.26012757 | 5.4403306 | 6.784347482 |
| GO:0003151 | outflow tract morphogenesis | 5 | 484 | 40 | 16457 | 0.009173284 | 0.26012757 | 4.2502583 | 6.76834605 |
| GO:0006766 | vitamin metabolic process | 8 | 484 | 95 | 16457 | 0.00990725 | 0.26012757 | 2.8633319 | 6.657299625 |
| GO:0042993 | positive regulation of transcription factor import into nucleus | 3 | 484 | 13 | 16457 | 0.009976908 | 0.26012757 | 7.8466306 | 6.647191524 |
| GO:0055003 | cardiac myofibril assembly | 3 | 484 | 13 | 16457 | 0.009976908 | 0.26012757 | 7.8466306 | 6.647191524 |
| GO:0048545 | response to steroid hormone | 5 | 484 | 41 | 16457 | 0.010052152 | 0.26012757 | 4.1465934 | 6.636351839 |
| GO:0071333 | cellular response to glucose stimulus | 5 | 484 | 41 | 16457 | 0.010052152 | 0.26012757 | 4.1465934 | 6.636351839 |

| | | | | | | | | | |
|---|---|---|---|---|---|---|---|---|---|
| GO:0006091 | generation of precursor metabolites and energy | 6 | 484 | 58 | 16457 | 0.010154326 | 0.26012757 | 3.5174551 | 6.621761765 |
| GO:0019886 | antigen processing and presentation of exogenous peptide antigen via MHC class II | 8 | 484 | 96 | 16457 | 0.010467348 | 0.26012757 | 2.8335055 | 6.577960212 |
| GO:0002503 | peptide antigen assembly with MHC class II protein complex | 2 | 484 | 4 | 16457 | 0.011403331 | 0.26012757 | 17.001033 | 6.454400885 |
| GO:0002175 | protein localization to paranode region of axon | 2 | 484 | 4 | 16457 | 0.011403331 | 0.26012757 | 17.001033 | 6.454400885 |
| GO:0051100 | negative regulation of binding | 2 | 484 | 4 | 16457 | 0.011403331 | 0.26012757 | 17.001033 | 6.454400885 |
| GO:0060028 | convergent extension involved in axis elongation | 2 | 484 | 4 | 16457 | 0.011403331 | 0.26012757 | 17.001033 | 6.454400885 |
| GO:0090175 | regulation of establishment of planar polarity | 2 | 484 | 4 | 16457 | 0.011403331 | 0.26012757 | 17.001033 | 6.454400885 |
| GO:0032909 | regulation of transforming growth factor beta2 production | 2 | 484 | 4 | 16457 | 0.011403331 | 0.26012757 | 17.001033 | 6.454400885 |
| GO:0033135 | regulation of peptidyl-serine phosphorylation | 2 | 484 | 4 | 16457 | 0.011403331 | 0.26012757 | 17.001033 | 6.454400885 |
| GO:0045542 | positive regulation of cholesterol biosynthetic process | 2 | 484 | 4 | 16457 | 0.011403331 | 0.26012757 | 17.001033 | 6.454400885 |
| GO:0003062 | regulation of heart rate by chemical signal | 2 | 484 | 4 | 16457 | 0.011403331 | 0.26012757 | 17.001033 | 6.454400885 |
| GO:0033076 | isoquinoline alkaloid metabolic process | 2 | 484 | 4 | 16457 | 0.011403331 | 0.26012757 | 17.001033 | 6.454400885 |
| GO:0010693 | negative regulation of alkaline phosphatase activity | 2 | 484 | 4 | 16457 | 0.011403331 | 0.26012757 | 17.001033 | 6.454400885 |
| GO:0060931 | sinoatrial node cell development | 2 | 484 | 4 | 16457 | 0.011403331 | 0.26012757 | 17.001033 | 6.454400885 |
| GO:0010718 | positive regulation of epithelial to mesenchymal transition | 4 | 484 | 27 | 16457 | 0.0114852 | 0.26012757 | 5.0373431 | 6.444080165 |
| GO:0001816 | cytokine production | 4 | 484 | 27 | 16457 | 0.0114852 | 0.26012757 | 5.0373431 | 6.444080165 |
| GO:0045780 | positive regulation of bone resorption | 3 | 484 | 14 | 16457 | 0.011858449 | 0.260274622 | 7.286157 | 6.397940837 |
| GO:0043568 | positive regulation of insulin-like growth factor receptor signaling pathway | 3 | 484 | 14 | 16457 | 0.011858449 | 0.260274622 | 7.286157 | 6.397940837 |
| GO:0060317 | cardiac epithelial to mesenchymal transition | 3 | 484 | 14 | 16457 | 0.011858449 | 0.260274622 | 7.286157 | 6.397940837 |
| GO:0015721 | bile acid and bile salt transport | 4 | 484 | 28 | 16457 | 0.012831893 | 0.278766336 | 4.857438 | 6.284122135 |
| GO:1902600 | hydrogen ion transmembrane transport | 7 | 484 | 81 | 16457 | 0.013555505 | 0.288854585 | 2.9384502 | 6.20497733 |
| GO:0042448 | progesterone metabolic process | 3 | 484 | 15 | 16457 | 0.013929502 | 0.288854585 | 6.8004132 | 6.165712524 |
| GO:0006096 | glycolytic process | 5 | 484 | 45 | 16457 | 0.014142253 | 0.288854585 | 3.7780073 | 6.143844264 |
| GO:0051149 | positive regulation of muscle cell differentiation | 4 | 484 | 29 | 16457 | 0.014275089 | 0.288854585 | 4.6899402 | 6.130356411 |
| GO:0007517 | muscle organ development | 8 | 484 | 103 | 16457 | 0.015063076 | 0.288854585 | 2.6409372 | 6.052839788 |
| GO:0051897 | positive regulation of protein kinase B signaling | 7 | 484 | 83 | 16457 | 0.015188376 | 0.288854585 | 2.8676441 | 6.040888527 |
| GO:0018916 | nitrobenzene metabolic process | 2 | 484 | 5 | 16457 | 0.01566182 | 0.288854585 | 13.600826 | 5.99660436 |
| GO:0006102 | isocitrate metabolic process | 2 | 484 | 5 | 16457 | 0.01566182 | 0.288854585 | 13.600826 | 5.99660436 |
| GO:0060029 | convergent extension involved in organogenesis | 2 | 484 | 5 | 16457 | 0.01566182 | 0.288854585 | 13.600826 | 5.99660436 |
| GO:0033173 | calcineurin-NFAT signaling cascade | 2 | 484 | 5 | 16457 | 0.01566182 | 0.288854585 | 13.600826 | 5.99660436 |
| GO:0019626 | short-chain fatty acid catabolic process | 2 | 484 | 5 | 16457 | 0.01566182 | 0.288854585 | 13.600826 | 5.99660436 |
| GO:0044259 | multicellular organismal macromolecule metabolic process | 2 | 484 | 5 | 16457 | 0.01566182 | 0.288854585 | 13.600826 | 5.99660436 |

| | | | | | | | | | |
|---|---|---|---|---|---|---|---|---|---|
| GO:0070458 | cellular detoxification of nitrogen compound | 2 | 484 | 5 | 16457 | 0.01566182 | 0.288854585 | 13.600826 | 5.99660436 |
| GO:0060666 | dichotomous subdivision of terminal units involved in salivary gland branching | 2 | 484 | 5 | 16457 | 0.01566182 | 0.288854585 | 13.600826 | 5.99660436 |
| GO:2001257 | regulation of cation channel activity | 2 | 484 | 5 | 16457 | 0.01566182 | 0.288854585 | 13.600826 | 5.99660436 |
| GO:0001780 | neutrophil homeostasis | 2 | 484 | 5 | 16457 | 0.01566182 | 0.288854585 | 13.600826 | 5.99660436 |
| GO:0038031 | non-canonical Wnt signaling pathway via JNK cascade | 2 | 484 | 5 | 16457 | 0.01566182 | 0.288854585 | 13.600826 | 5.99660436 |
| GO:0014902 | myotube differentiation | 3 | 484 | 16 | 16457 | 0.016192396 | 0.288854585 | 6.3753874 | 5.948539754 |
| GO:0051453 | regulation of intracellular pH | 3 | 484 | 16 | 16457 | 0.016192396 | 0.288854585 | 6.3753874 | 5.948539754 |
| GO:0042136 | neurotransmitter biosynthetic process | 3 | 484 | 16 | 16457 | 0.016192396 | 0.288854585 | 6.3753874 | 5.948539754 |
| GO:0070848 | response to growth factor | 3 | 484 | 16 | 16457 | 0.016192396 | 0.288854585 | 6.3753874 | 5.948539754 |
| GO:0043434 | response to peptide hormone | 6 | 484 | 65 | 16457 | 0.016383236 | 0.288854585 | 3.1386523 | 5.931635834 |
| GO:0009267 | cellular response to starvation | 5 | 484 | 47 | 16457 | 0.016552494 | 0.288854585 | 3.6172411 | 5.916807596 |
| GO:0019432 | triglyceride biosynthetic process | 5 | 484 | 47 | 16457 | 0.016552494 | 0.288854585 | 3.6172411 | 5.916807596 |
| GO:0030198 | extracellular matrix organization | 17 | 484 | 316 | 16457 | 0.017743444 | 0.300810118 | 1.8292251 | 5.816570125 |
| GO:0035914 | skeletal muscle cell differentiation | 5 | 484 | 48 | 16457 | 0.017853621 | 0.300810118 | 3.5418819 | 5.807639501 |
| GO:0007420 | brain development | 13 | 484 | 220 | 16457 | 0.018445673 | 0.300810118 | 2.009213 | 5.760573751 |
| GO:0042632 | cholesterol homeostasis | 6 | 484 | 67 | 16457 | 0.018559081 | 0.300810118 | 3.0449611 | 5.751730923 |
| GO:0070542 | response to fatty acid | 3 | 484 | 17 | 16457 | 0.018648745 | 0.300810118 | 6.0003646 | 5.744777682 |
| GO:0042593 | glucose homeostasis | 8 | 484 | 108 | 16457 | 0.019136182 | 0.300810118 | 2.5186716 | 5.70755317 |
| GO:0015701 | bicarbonate transport | 4 | 484 | 32 | 16457 | 0.019203657 | 0.300810118 | 4.2502583 | 5.702475097 |
| GO:1900740 | positive regulation of protein insertion into mitochondrial membrane involved in apoptotic signaling pathway | 4 | 484 | 32 | 16457 | 0.019203657 | 0.300810118 | 4.2502583 | 5.702475097 |
| GO:0003007 | heart morphogenesis | 5 | 484 | 49 | 16457 | 0.019220386 | 0.300810118 | 3.4695986 | 5.701218902 |
| GO:0016055 | Wnt signaling pathway | 12 | 484 | 198 | 16457 | 0.019408382 | 0.300810118 | 2.0607313 | 5.687176343 |
| GO:0001889 | liver development | 7 | 484 | 88 | 16457 | 0.019871919 | 0.300810118 | 2.7047098 | 5.653124998 |
| GO:0007229 | integrin-mediated signaling pathway | 8 | 484 | 109 | 16457 | 0.02003632 | 0.300810118 | 2.4955645 | 5.641238604 |
| GO:0046825 | regulation of protein export from nucleus | 2 | 484 | 6 | 16457 | 0.020487303 | 0.300810118 | 11.334022 | 5.609126143 |
| GO:0045823 | positive regulation of heart contraction | 2 | 484 | 6 | 16457 | 0.020487303 | 0.300810118 | 11.334022 | 5.609126143 |
| GO:0048743 | positive regulation of skeletal muscle fiber development | 2 | 484 | 6 | 16457 | 0.020487303 | 0.300810118 | 11.334022 | 5.609126143 |
| GO:0014894 | response to denervation involved in regulation of muscle adaptation | 2 | 484 | 6 | 16457 | 0.020487303 | 0.300810118 | 11.334022 | 5.609126143 |
| GO:0018026 | peptidyl-lysine monomethylation | 2 | 484 | 6 | 16457 | 0.020487303 | 0.300810118 | 11.334022 | 5.609126143 |
| GO:0035356 | cellular triglyceride homeostasis | 2 | 484 | 6 | 16457 | 0.020487303 | 0.300810118 | 11.334022 | 5.609126143 |
| GO:0019217 | regulation of fatty acid metabolic process | 2 | 484 | 6 | 16457 | 0.020487303 | 0.300810118 | 11.334022 | 5.609126143 |
| GO:0042693 | muscle cell fate commitment | 2 | 484 | 6 | 16457 | 0.020487303 | 0.300810118 | 11.334022 | 5.609126143 |

| | | | | | | | | | |
|---|---|---|---|---|---|---|---|---|---|
| GO:0015672 | monovalent inorganic cation transport | 2 | 484 | 6 | 16457 | 0.020487303 | 0.300810118 | 11.334022 | 5.609126143 |
| GO:1901621 | negative regulation of smoothened signaling pathway involved in dorsal/ventral neural tube patterning | 2 | 484 | 6 | 16457 | 0.020487303 | 0.300810118 | 11.334022 | 5.609126143 |
| GO:0030497 | fatty acid elongation | 2 | 484 | 6 | 16457 | 0.020487303 | 0.300810118 | 11.334022 | 5.609126143 |
| GO:0007155 | cell adhesion | 28 | 484 | 610 | 16457 | 0.020813281 | 0.303503249 | 1.5607506 | 5.58635181 |
| GO:0060047 | heart contraction | 3 | 484 | 18 | 16457 | 0.021299499 | 0.308480505 | 5.667011 | 5.553036668 |
| GO:0007507 | heart development | 12 | 484 | 203 | 16457 | 0.022793227 | 0.32788365 | 2.0099744 | 5.455251003 |
| GO:0046928 | regulation of neurotransmitter secretion | 3 | 484 | 19 | 16457 | 0.024144996 | 0.342697971 | 5.3687473 | 5.372131987 |
| GO:0036342 | post-anal tail morphogenesis | 3 | 484 | 19 | 16457 | 0.024144996 | 0.342697971 | 5.3687473 | 5.372131987 |
| GO:0060056 | mammary gland involution | 2 | 484 | 7 | 16457 | 0.02584369 | 0.346045389 | 9.714876 | 5.274044092 |
| GO:0051533 | positive regulation of NFAT protein import into nucleus | 2 | 484 | 7 | 16457 | 0.02584369 | 0.346045389 | 9.714876 | 5.274044092 |
| GO:2000188 | regulation of cholesterol homeostasis | 2 | 484 | 7 | 16457 | 0.02584369 | 0.346045389 | 9.714876 | 5.274044092 |
| GO:0090244 | Wnt signaling pathway involved in somitogenesis | 2 | 484 | 7 | 16457 | 0.02584369 | 0.346045389 | 9.714876 | 5.274044092 |
| GO:0006657 | CDP-choline pathway | 2 | 484 | 7 | 16457 | 0.02584369 | 0.346045389 | 9.714876 | 5.274044092 |
| GO:2001275 | positive regulation of glucose import in response to insulin stimulus | 2 | 484 | 7 | 16457 | 0.02584369 | 0.346045389 | 9.714876 | 5.274044092 |
| GO:0071786 | endoplasmic reticulum tubular network organization | 2 | 484 | 7 | 16457 | 0.02584369 | 0.346045389 | 9.714876 | 5.274044092 |
| GO:0043931 | ossification involved in bone maturation | 2 | 484 | 7 | 16457 | 0.02584369 | 0.346045389 | 9.714876 | 5.274044092 |
| GO:0070206 | protein trimerization | 2 | 484 | 7 | 16457 | 0.02584369 | 0.346045389 | 9.714876 | 5.274044092 |
| GO:0009653 | anatomical structure morphogenesis | 8 | 484 | 115 | 16457 | 0.026074734 | 0.346956926 | 2.3653611 | 5.261203668 |
| GO:0014068 | positive regulation of phosphatidylinositol 3-kinase signaling | 5 | 484 | 54 | 16457 | 0.027076629 | 0.354746999 | 3.1483395 | 5.206808029 |
| GO:0002504 | antigen processing and presentation of peptide or polysaccharide antigen via MHC class II | 3 | 484 | 20 | 16457 | 0.027185 | 0.354746999 | 5.1003099 | 5.201045355 |
| GO:0034113 | heterotypic cell-cell adhesion | 3 | 484 | 20 | 16457 | 0.027185 | 0.354746999 | 5.1003099 | 5.201045355 |
| GO:0050790 | regulation of catalytic activity | 8 | 484 | 117 | 16457 | 0.028340812 | 0.354746999 | 2.3249276 | 5.140975078 |
| GO:0006641 | triglyceride metabolic process | 4 | 484 | 37 | 16457 | 0.02950781 | 0.354746999 | 3.675899 | 5.08275936 |
| GO:0060314 | regulation of ryanodine-sensitive calcium-release channel activity | 3 | 484 | 21 | 16457 | 0.030418755 | 0.354746999 | 4.857438 | 5.038895102 |
| GO:0071526 | semaphorin-plexin signaling pathway | 3 | 484 | 21 | 16457 | 0.030418755 | 0.354746999 | 4.857438 | 5.038895102 |
| GO:0030511 | positive regulation of transforming growth factor beta receptor signaling pathway | 3 | 484 | 21 | 16457 | 0.030418755 | 0.354746999 | 4.857438 | 5.038895102 |
| GO:0045471 | response to ethanol | 7 | 484 | 97 | 16457 | 0.030671344 | 0.354746999 | 2.4537573 | 5.02696479 |
| GO:0001501 | skeletal system development | 10 | 484 | 165 | 16457 | 0.030815099 | 0.354746999 | 2.0607313 | 5.020218746 |
| GO:0048170 | positive regulation of long-term neuronal synaptic plasticity | 2 | 484 | 8 | 16457 | 0.031696502 | 0.354746999 | 8.5005165 | 4.979532539 |
| GO:0045162 | clustering of voltage-gated sodium channels | 2 | 484 | 8 | 16457 | 0.031696502 | 0.354746999 | 8.5005165 | 4.979532539 |
| GO:0046683 | response to organophosphorus | 2 | 484 | 8 | 16457 | 0.031696502 | 0.354746999 | 8.5005165 | 4.979532539 |
| GO:0002318 | myeloid progenitor cell differentiation | 2 | 484 | 8 | 16457 | 0.031696502 | 0.354746999 | 8.5005165 | 4.979532539 |

| | | | | | | | | | |
|---|---|---|---|---|---|---|---|---|---|
| GO:0035176 | social behavior | 4 | 484 | 38 | 16457 | 0.031892202 | 0.354746999 | 3.5791649 | 4.970652467 |
| GO:0007612 | learning | 5 | 484 | 57 | 16457 | 0.032641261 | 0.354746999 | 2.9826374 | 4.937159414 |
| GO:0016311 | dephosphorylation | 13 | 484 | 241 | 16457 | 0.033796572 | 0.354746999 | 1.8341363 | 4.886979256 |
| GO:0046034 | ATP metabolic process | 3 | 484 | 22 | 16457 | 0.033845017 | 0.354746999 | 4.6366454 | 4.884912741 |
| GO:0010880 | regulation of release of sequestered calcium ion into cytosol by sarcoplasmic reticulum | 3 | 484 | 22 | 16457 | 0.033845017 | 0.354746999 | 4.6366454 | 4.884912741 |
| GO:0090103 | cochlea morphogenesis | 3 | 484 | 22 | 16457 | 0.033845017 | 0.354746999 | 4.6366454 | 4.884912741 |
| GO:0042574 | retinal metabolic process | 3 | 484 | 22 | 16457 | 0.033845017 | 0.354746999 | 4.6366454 | 4.884912741 |
| GO:0050771 | negative regulation of axonogenesis | 3 | 484 | 22 | 16457 | 0.033845017 | 0.354746999 | 4.6366454 | 4.884912741 |
| GO:0042326 | negative regulation of phosphorylation | 3 | 484 | 22 | 16457 | 0.033845017 | 0.354746999 | 4.6366454 | 4.884912741 |
| GO:0008152 | metabolic process | 77 | 484 | 2059 | 16457 | 0.034111345 | 0.354746999 | 1.2715683 | 4.873604541 |
| GO:0042692 | muscle cell differentiation | 4 | 484 | 39 | 16457 | 0.034386078 | 0.354746999 | 3.4873914 | 4.862031618 |
| GO:0045668 | negative regulation of osteoblast differentiation | 4 | 484 | 39 | 16457 | 0.034386078 | 0.354746999 | 3.4873914 | 4.862031618 |
| GO:0048839 | inner ear development | 5 | 484 | 59 | 16457 | 0.036717687 | 0.354746999 | 2.881531 | 4.767381021 |
| GO:0010811 | positive regulation of cell-substrate adhesion | 4 | 484 | 40 | 16457 | 0.03698981 | 0.354746999 | 3.4002066 | 4.756728282 |
| GO:0051480 | cytosolic calcium ion homeostasis | 3 | 484 | 23 | 16457 | 0.037462101 | 0.354746999 | 4.4350521 | 4.738424367 |
| GO:0045672 | positive regulation of osteoclast differentiation | 3 | 484 | 23 | 16457 | 0.037462101 | 0.354746999 | 4.4350521 | 4.738424367 |
| GO:0006783 | heme biosynthetic process | 3 | 484 | 23 | 16457 | 0.037462101 | 0.354746999 | 4.4350521 | 4.738424367 |
| GO:0019511 | peptidyl-proline hydroxylation | 2 | 484 | 9 | 16457 | 0.038012804 | 0.354746999 | 7.5560147 | 4.717370745 |
| GO:0010886 | positive regulation of cholesterol storage | 2 | 484 | 9 | 16457 | 0.038012804 | 0.354746999 | 7.5560147 | 4.717370745 |
| GO:0048739 | cardiac muscle fiber development | 2 | 484 | 9 | 16457 | 0.038012804 | 0.354746999 | 7.5560147 | 4.717370745 |
| GO:0006942 | regulation of striated muscle contraction | 2 | 484 | 9 | 16457 | 0.038012804 | 0.354746999 | 7.5560147 | 4.717370745 |
| GO:0033148 | positive regulation of intracellular estrogen receptor signaling pathway | 2 | 484 | 9 | 16457 | 0.038012804 | 0.354746999 | 7.5560147 | 4.717370745 |
| GO:0071514 | genetic imprinting | 2 | 484 | 9 | 16457 | 0.038012804 | 0.354746999 | 7.5560147 | 4.717370745 |
| GO:0008090 | retrograde axon cargo transport | 2 | 484 | 9 | 16457 | 0.038012804 | 0.354746999 | 7.5560147 | 4.717370745 |
| GO:0061045 | negative regulation of wound healing | 2 | 484 | 9 | 16457 | 0.038012804 | 0.354746999 | 7.5560147 | 4.717370745 |
| GO:0045580 | regulation of T cell differentiation | 2 | 484 | 9 | 16457 | 0.038012804 | 0.354746999 | 7.5560147 | 4.717370745 |
| GO:0060539 | diaphragm development | 2 | 484 | 9 | 16457 | 0.038012804 | 0.354746999 | 7.5560147 | 4.717370745 |
| GO:0042416 | dopamine biosynthetic process | 2 | 484 | 9 | 16457 | 0.038012804 | 0.354746999 | 7.5560147 | 4.717370745 |
| GO:0006195 | purine nucleotide catabolic process | 2 | 484 | 9 | 16457 | 0.038012804 | 0.354746999 | 7.5560147 | 4.717370745 |
| GO:0043534 | blood vessel endothelial cell migration | 2 | 484 | 9 | 16457 | 0.038012804 | 0.354746999 | 7.5560147 | 4.717370745 |
| GO:0060048 | cardiac muscle contraction | 4 | 484 | 41 | 16457 | 0.039703618 | 0.354746999 | 3.3172747 | 4.654585713 |
| GO:0006656 | phosphatidylcholine biosynthetic process | 3 | 484 | 24 | 16457 | 0.041267913 | 0.354746999 | 4.2502583 | 4.598835721 |

| | | | | | | | | | |
|---|---|---|---|---|---|---|---|---|---|
| GO:0055007 | cardiac muscle cell differentiation | 3 | 484 | 24 | 16457 | 0.041267913 | 0.354746999 | 4.2502583 | 4.598835721 |
| GO:0051924 | regulation of calcium ion transport | 3 | 484 | 24 | 16457 | 0.041267913 | 0.354746999 | 4.2502583 | 4.598835721 |
| GO:0000226 | microtubule cytoskeleton organization | 6 | 484 | 82 | 16457 | 0.041299928 | 0.354746999 | 2.4879561 | 4.597716926 |
| GO:0032355 | response to estradiol | 6 | 484 | 82 | 16457 | 0.041299928 | 0.354746999 | 2.4879561 | 4.597716926 |
| GO:0007411 | axon guidance | 19 | 484 | 407 | 16457 | 0.042292784 | 0.354746999 | 1.58732 | 4.56344467 |
| GO:0007409 | axonogenesis | 7 | 484 | 105 | 16457 | 0.043082088 | 0.354746999 | 2.2668044 | 4.536768015 |
| GO:0030857 | negative regulation of epithelial cell differentiation | 2 | 484 | 10 | 16457 | 0.044761146 | 0.354746999 | 6.8004132 | 4.481609219 |
| GO:0033555 | multicellular organismal response to stress | 2 | 484 | 10 | 16457 | 0.044761146 | 0.354746999 | 6.8004132 | 4.481609219 |
| GO:0061037 | negative regulation of cartilage development | 2 | 484 | 10 | 16457 | 0.044761146 | 0.354746999 | 6.8004132 | 4.481609219 |
| GO:0001774 | microglial cell activation | 2 | 484 | 10 | 16457 | 0.044761146 | 0.354746999 | 6.8004132 | 4.481609219 |
| GO:0009072 | aromatic amino acid family metabolic process | 2 | 484 | 10 | 16457 | 0.044761146 | 0.354746999 | 6.8004132 | 4.481609219 |
| GO:0071380 | cellular response to prostaglandin E stimulus | 2 | 484 | 10 | 16457 | 0.044761146 | 0.354746999 | 6.8004132 | 4.481609219 |
| GO:0051412 | response to corticosterone | 3 | 484 | 25 | 16457 | 0.045259984 | 0.354746999 | 4.0802479 | 4.465620104 |
| **Molecular function category** | | | | | | | | | |
| **GO ID** | **GO Term** | **DifGene** | **AllDifGene** | **GeneInGO** | **AllGene** | **P-Value** | **FDR** | **Enrichment** | **(-log2P)** |
| GO:0042813 | Wnt-activated receptor activity | 6 | 474 | 20 | 16096 | 7.97996E-05 | 0.036726146 | 10.187342 | 13.61325842 |
| GO:0016491 | oxidoreductase activity | 37 | 474 | 619 | 16096 | 0.000145694 | 0.036726146 | 2.0297881 | 12.74476816 |
| GO:0001968 | fibronectin binding | 6 | 474 | 23 | 16096 | 0.000152813 | 0.036726146 | 8.8585581 | 12.67594162 |
| GO:0019899 | enzyme binding | 24 | 474 | 343 | 16096 | 0.000233419 | 0.042073725 | 2.3760564 | 12.06479208 |
| GO:0016616 | oxidoreductase activity, acting on the CH-OH group of donors, NAD or NADP as acceptor | 7 | 474 | 41 | 16096 | 0.000429276 | 0.057031377 | 5.7976742 | 11.18580661 |
| GO:0047115 | trans-1,2-dihydrobenzene-1,2-diol dehydrogenase activity | 4 | 474 | 10 | 16096 | 0.000542038 | 0.057031377 | 13.583122 | 10.84931842 |
| GO:0051287 | NAD binding | 7 | 474 | 43 | 16096 | 0.000553703 | 0.057031377 | 5.5280149 | 10.81860085 |
| GO:0004723 | calcium-dependent protein serine/threonine phosphatase activity | 3 | 474 | 4 | 16096 | 0.000760085 | 0.068502664 | 25.468354 | 10.36155155 |
| GO:0031406 | carboxylic acid binding | 4 | 474 | 12 | 16096 | 0.000940976 | 0.075382655 | 11.319269 | 10.05355403 |
| GO:0009374 | biotin binding | 3 | 474 | 5 | 16096 | 0.001189992 | 0.085798416 | 20.374684 | 9.714832523 |
| GO:0017147 | Wnt-protein binding | 5 | 474 | 26 | 16096 | 0.001789423 | 0.117288522 | 6.5303473 | 9.126290086 |
| GO:0030170 | pyridoxal phosphate binding | 7 | 474 | 55 | 16096 | 0.002016697 | 0.121169891 | 4.3219026 | 8.953789793 |
| GO:0005178 | integrin binding | 10 | 474 | 110 | 16096 | 0.002586378 | 0.137061749 | 3.0870733 | 8.594851164 |
| GO:0016655 | oxidoreductase activity, acting on NAD(P)H, quinone or similar compound as acceptor | 4 | 474 | 17 | 16096 | 0.002757701 | 0.137061749 | 7.990072 | 8.502318443 |
| GO:0042803 | protein homodimerization activity | 35 | 474 | 700 | 16096 | 0.003581698 | 0.137061749 | 1.6978903 | 8.125140628 |
| GO:0005516 | calmodulin binding | 13 | 474 | 176 | 16096 | 0.003596939 | 0.137061749 | 2.508247 | 8.11901466 |
| GO:0003824 | catalytic activity | 36 | 474 | 727 | 16096 | 0.003682704 | 0.137061749 | 1.681542 | 8.085018789 |

| | | | | | | | | | |
|---|---|---|---|---|---|---|---|---|---|
| GO:0004033 | aldo-keto reductase (NADP) activity | 4 | 474 | 19 | 16096 | 0.003897099 | 0.137061749 | 7.1490118 | 8.003383626 |
| GO:0018636 | phenanthrene 9,10-monooxygenase activity | 3 | 474 | 9 | 16096 | 0.004287216 | 0.137061749 | 11.319269 | 7.865743125 |
| GO:0047086 | ketosteroid monooxygenase activity | 3 | 474 | 9 | 16096 | 0.004287216 | 0.137061749 | 11.319269 | 7.865743125 |
| GO:0008013 | beta-catenin binding | 7 | 474 | 65 | 16096 | 0.004706509 | 0.137061749 | 3.6569945 | 7.731126926 |
| GO:0004658 | propionyl-CoA carboxylase activity | 2 | 474 | 2 | 16096 | 0.004752488 | 0.137061749 | 33.957806 | 7.717101348 |
| GO:0004457 | lactate dehydrogenase activity | 2 | 474 | 2 | 16096 | 0.004752488 | 0.137061749 | 33.957806 | 7.717101348 |
| GO:0004450 | isocitrate dehydrogenase (NADP+) activity | 2 | 474 | 2 | 16096 | 0.004752488 | 0.137061749 | 33.957806 | 7.717101348 |
| GO:0030395 | lactose binding | 2 | 474 | 2 | 16096 | 0.004752488 | 0.137061749 | 33.957806 | 7.717101348 |
| GO:0004872 | receptor activity | 17 | 474 | 273 | 16096 | 0.005090736 | 0.141170033 | 2.1145886 | 7.617909952 |
| GO:0047718 | indanol dehydrogenase activity | 3 | 474 | 10 | 16096 | 0.005454508 | 0.145655562 | 10.187342 | 7.518335252 |
| GO:0016740 | transferase activity | 64 | 474 | 1507 | 16096 | 0.005720927 | 0.147313875 | 1.4421364 | 7.449535305 |
| GO:0008483 | transaminase activity | 4 | 474 | 22 | 16096 | 0.006143072 | 0.149604072 | 6.1741465 | 7.346824041 |
| GO:0005509 | calcium ion binding | 33 | 474 | 676 | 16096 | 0.006224857 | 0.149604072 | 1.6577035 | 7.327743501 |
| GO:0031748 | D1 dopamine receptor binding | 3 | 474 | 11 | 16096 | 0.006794272 | 0.158021625 | 9.2612198 | 7.20146523 |
| GO:0000309 | nicotinamide-nucleotide adenylyltransferase activity | 2 | 474 | 3 | 16096 | 0.007769588 | 0.169753728 | 22.638537 | 7.00794617 |
| GO:0032810 | sterol response element binding | 2 | 474 | 3 | 16096 | 0.007769588 | 0.169753728 | 22.638537 | 7.00794617 |
| GO:0043295 | glutathione binding | 3 | 474 | 12 | 16096 | 0.008312282 | 0.176269268 | 8.4894515 | 6.910539737 |
| GO:0004129 | cytochrome-c oxidase activity | 5 | 474 | 40 | 16096 | 0.009224113 | 0.190016736 | 4.2447257 | 6.760374034 |
| GO:0004721 | phosphoprotein phosphatase activity | 11 | 474 | 157 | 16096 | 0.009918232 | 0.195125052 | 2.3792093 | 6.655701252 |
| GO:0004032 | alditol:NADP+ 1-oxidoreductase activity | 3 | 474 | 13 | 16096 | 0.010013352 | 0.195125052 | 7.8364167 | 6.641931161 |
| GO:0031014 | troponin T binding | 2 | 474 | 4 | 16096 | 0.011432436 | 0.216915435 | 16.978903 | 6.450723316 |
| GO:0022891 | substrate-specific transmembrane transporter activity | 3 | 474 | 14 | 16096 | 0.011901409 | 0.220023479 | 7.2766727 | 6.392723841 |
| GO:0000988 | protein binding transcription factor activity | 2 | 474 | 5 | 16096 | 0.015701377 | 0.244539088 | 13.583122 | 5.992965126 |
| GO:0005007 | fibroblast growth factor-activated receptor activity | 2 | 474 | 5 | 16096 | 0.015701377 | 0.244539088 | 13.583122 | 5.992965126 |
| GO:0046923 | ER retention sequence binding | 2 | 474 | 5 | 16096 | 0.015701377 | 0.244539088 | 13.583122 | 5.992965126 |
| GO:0015125 | bile acid transmembrane transporter activity | 2 | 474 | 5 | 16096 | 0.015701377 | 0.244539088 | 13.583122 | 5.992965126 |
| GO:0004459 | L-lactate dehydrogenase activity | 2 | 474 | 5 | 16096 | 0.015701377 | 0.244539088 | 13.583122 | 5.992965126 |
| GO:0047042 | androsterone dehydrogenase (B-specific) activity | 2 | 474 | 5 | 16096 | 0.015701377 | 0.244539088 | 13.583122 | 5.992965126 |
| GO:0004515 | nicotinate-nucleotide adenylyltransferase activity | 2 | 474 | 5 | 16096 | 0.015701377 | 0.244539088 | 13.583122 | 5.992965126 |
| GO:0009055 | electron carrier activity | 8 | 474 | 104 | 16096 | 0.015940828 | 0.244539088 | 2.6121389 | 5.971129606 |
| GO:0016829 | lyase activity | 10 | 474 | 151 | 16096 | 0.018814787 | 0.279401172 | 2.2488613 | 5.731989235 |
| GO:0003774 | motor activity | 7 | 474 | 88 | 16096 | 0.020004303 | 0.279401172 | 2.7011891 | 5.643545835 |

| | | | | | | | | | |
|---|---|---|---|---|---|---|---|---|---|
| GO:0032036 | myosin heavy chain binding | 2 | 474 | 6 | 16096 | 0.020538505 | 0.279401172 | 11.319269 | 5.605525018 |
| GO:0004075 | biotin carboxylase activity | 2 | 474 | 6 | 16096 | 0.020538505 | 0.279401172 | 11.319269 | 5.605525018 |
| GO:0047023 | androsterone dehydrogenase activity | 2 | 474 | 6 | 16096 | 0.020538505 | 0.279401172 | 11.319269 | 5.605525018 |
| GO:0047006 | 17-alpha,20-alpha-dihydroxypregn-4-en-3-one dehydrogenase activity | 2 | 474 | 6 | 16096 | 0.020538505 | 0.279401172 | 11.319269 | 5.605525018 |
| GO:0015459 | potassium channel regulator activity | 4 | 474 | 33 | 16096 | 0.021144441 | 0.280195253 | 4.1160977 | 5.563577766 |
| GO:0023026 | MHC class II protein complex binding | 3 | 474 | 18 | 16096 | 0.021374118 | 0.280195253 | 5.6596343 | 5.547991315 |
| GO:0019901 | protein kinase binding | 19 | 474 | 384 | 16096 | 0.026857679 | 0.310997203 | 1.6802039 | 5.218521555 |
| GO:0004683 | calmodulin-dependent protein kinase activity | 3 | 474 | 20 | 16096 | 0.027278635 | 0.310997203 | 5.0936709 | 5.196084718 |
| GO:0030971 | receptor tyrosine kinase binding | 4 | 474 | 36 | 16096 | 0.027349453 | 0.310997203 | 3.7730895 | 5.192344195 |
| GO:0030165 | PDZ domain binding | 7 | 474 | 95 | 16096 | 0.028172353 | 0.310997203 | 2.5021541 | 5.149576099 |
| GO:0003779 | actin binding | 18 | 474 | 365 | 16096 | 0.03123132 | 0.310997203 | 1.6746315 | 5.000862656 |
| GO:0034235 | GPI anchor binding | 2 | 474 | 8 | 16096 | 0.031774056 | 0.310997203 | 8.4894515 | 4.976006943 |
| GO:0004722 | protein serine/threonine phosphatase activity | 5 | 474 | 58 | 16096 | 0.034813665 | 0.310997203 | 2.9273971 | 4.844202495 |
| GO:0001085 | RNA polymerase II transcription factor binding | 4 | 474 | 40 | 16096 | 0.037144252 | 0.310997203 | 3.3957806 | 4.750717199 |
| GO:0017124 | SH3 domain binding | 8 | 474 | 124 | 16096 | 0.037583938 | 0.310997203 | 2.1908262 | 4.733739947 |
| GO:0050840 | extracellular matrix binding | 3 | 474 | 23 | 16096 | 0.03758786 | 0.310997203 | 4.429279 | 4.733589409 |
| GO:0015297 | antiporter activity | 4 | 474 | 41 | 16096 | 0.039868168 | 0.310997203 | 3.3129567 | 4.648618861 |
| GO:0017154 | semaphorin receptor activity | 2 | 474 | 10 | 16096 | 0.044868345 | 0.310997203 | 6.7915612 | 4.478158235 |
| GO:0000983 | RNA polymerase II core promoter sequence-specific DNA binding transcription factor activity | 2 | 474 | 10 | 16096 | 0.044868345 | 0.310997203 | 6.7915612 | 4.478158235 |
| GO:0032052 | bile acid binding | 2 | 474 | 10 | 16096 | 0.044868345 | 0.310997203 | 6.7915612 | 4.478158235 |
| GO:0005518 | collagen binding | 5 | 474 | 63 | 16096 | 0.045987002 | 0.310997203 | 2.695064 | 4.442630044 |
| **Cellular component category** | | | | | | | | | |
| **GOID** | **GOTerm** | **DifGene** | **AllDifGene** | **GeneInGO** | **AllGene** | **P-Value** | **FDR** | **Enrichment** | **(-log2P)** |
| GO:0005759 | mitochondrial matrix | 27 | 491 | 271 | 17318 | 1.39715E-07 | 4.91798E-05 | 3.5140725 | 22.77100756 |
| GO:0005739 | mitochondrion | 88 | 491 | 1671 | 17318 | 6.50518E-07 | 0.000114491 | 1.8574728 | 20.55190852 |
| GO:0016529 | sarcoplasmic reticulum | 10 | 491 | 48 | 17318 | 4.2825E-06 | 0.000502479 | 7.3480991 | 17.83311689 |
| GO:0030018 | Z disc | 15 | 491 | 119 | 17318 | 5.79511E-06 | 0.000509969 | 4.4459087 | 17.39673306 |
| GO:0031012 | extracellular matrix | 20 | 491 | 242 | 17318 | 5.96347E-05 | 0.004198283 | 2.9149484 | 14.0334884 |
| GO:0033017 | sarcoplasmic reticulum membrane | 7 | 491 | 31 | 17318 | 7.5238E-05 | 0.004413963 | 7.9643913 | 13.69817888 |
| GO:0005604 | basement membrane | 11 | 491 | 91 | 17318 | 0.000134304 | 0.006753578 | 4.2635125 | 12.86220898 |
| GO:0042383 | sarcolemma | 10 | 491 | 92 | 17318 | 0.000571837 | 0.025160838 | 3.8337908 | 10.77210781 |
| GO:0030424 | axon | 17 | 491 | 240 | 17318 | 0.001012898 | 0.03961555 | 2.4983537 | 9.947295972 |

| | | | | | | | | | |
|---|---|---|---|---|---|---|---|---|---|
| GO:0031224 | intrinsic component of membrane | 4 | 491 | 15 | 17318 | 0.001633178 | 0.057487857 | 9.4055669 | 9.258102461 |
| GO:0005861 | troponin complex | 3 | 491 | 8 | 17318 | 0.00295913 | 0.068214504 | 13.226578 | 8.400611427 |
| GO:0005751 | mitochondrial respiratory chain complex IV | 3 | 491 | 8 | 17318 | 0.00295913 | 0.068214504 | 13.226578 | 8.400611427 |
| GO:0033018 | sarcoplasmic reticulum lumen | 3 | 491 | 8 | 17318 | 0.00295913 | 0.068214504 | 13.226578 | 8.400611427 |
| GO:0005829 | cytosol | 117 | 491 | 3053 | 17318 | 0.002962568 | 0.068214504 | 1.3516844 | 8.398936031 |
| GO:0001725 | stress fiber | 7 | 491 | 62 | 17318 | 0.003033237 | 0.068214504 | 3.9821957 | 8.364925889 |
| GO:0030016 | myofibril | 6 | 491 | 46 | 17318 | 0.003100659 | 0.068214504 | 4.600549 | 8.333209295 |
| GO:0005955 | calcineurin complex | 2 | 491 | 3 | 17318 | 0.007231088 | 0.14685784 | 23.513917 | 7.111571534 |
| GO:0097038 | perinuclear endoplasmic reticulum | 3 | 491 | 12 | 17318 | 0.007509776 | 0.14685784 | 8.8177189 | 7.057014426 |
| GO:0016459 | myosin complex | 6 | 491 | 57 | 17318 | 0.007993083 | 0.14808238 | 3.7127238 | 6.967032209 |
| GO:0043025 | neuronal cell body | 17 | 491 | 305 | 17318 | 0.0096411 | 0.169683352 | 1.9659177 | 6.696586597 |
| GO:0005753 | mitochondrial proton-transporting ATP synthase complex | 4 | 491 | 27 | 17318 | 0.010176802 | 0.170360496 | 5.2253149 | 6.618571883 |
| GO:0045254 | pyruvate dehydrogenase complex | 2 | 491 | 4 | 17318 | 0.010647531 | 0.170360496 | 17.635438 | 6.553337257 |
| GO:0030315 | T-tubule | 5 | 491 | 45 | 17318 | 0.012284103 | 0.185668122 | 3.9189862 | 6.347063639 |
| GO:0005859 | muscle myosin complex | 3 | 491 | 15 | 17318 | 0.01265919 | 0.185668122 | 7.0541752 | 6.303671081 |
| GO:0005652 | nuclear lamina | 2 | 491 | 5 | 17318 | 0.014633586 | 0.199375067 | 14.10835 | 6.094572874 |
| GO:0031672 | A band | 3 | 491 | 16 | 17318 | 0.014726567 | 0.199375067 | 6.6132892 | 6.085434995 |
| GO:0005578 | proteinaceous extracellular matrix | 16 | 491 | 297 | 17318 | 0.015424469 | 0.201089369 | 1.9001145 | 6.018635402 |
| GO:0005938 | cell cortex | 9 | 491 | 131 | 17318 | 0.016599063 | 0.206017282 | 2.4231899 | 5.912754408 |
| GO:0032982 | myosin filament | 3 | 491 | 17 | 17318 | 0.016973015 | 0.206017282 | 6.2242722 | 5.880613354 |
| GO:0005891 | voltage-gated calcium channel complex | 4 | 491 | 33 | 17318 | 0.018740431 | 0.217502438 | 4.2752577 | 5.737702042 |
| GO:0030314 | junctional membrane complex | 2 | 491 | 6 | 17318 | 0.019155044 | 0.217502438 | 11.756959 | 5.706131828 |
| GO:0030017 | sarcomere | 5 | 491 | 52 | 17318 | 0.020723065 | 0.227953712 | 3.3914304 | 5.592618814 |
| GO:0005875 | microtubule associated complex | 4 | 491 | 35 | 17318 | 0.022347169 | 0.238369802 | 4.0309572 | 5.483764118 |
| GO:0032589 | neuron projection membrane | 2 | 491 | 7 | 17318 | 0.024179161 | 0.240369741 | 10.077393 | 5.370091997 |
| GO:0030132 | clathrin coat of coated pit | 2 | 491 | 7 | 17318 | 0.024179161 | 0.240369741 | 10.077393 | 5.370091997 |
| GO:0042613 | MHC class II protein complex | 3 | 491 | 20 | 17318 | 0.024796408 | 0.240369741 | 5.2906314 | 5.333725022 |
| GO:0030136 | clathrin-coated vesicle | 5 | 491 | 55 | 17318 | 0.025266138 | 0.240369741 | 3.2064433 | 5.306651056 |
| GO:0043203 | axon hillock | 2 | 491 | 8 | 17318 | 0.029674597 | 0.261136456 | 8.8177189 | 5.074627738 |
| GO:0042587 | glycogen granule | 2 | 491 | 8 | 17318 | 0.029674597 | 0.261136456 | 8.8177189 | 5.074627738 |
| GO:0014701 | junctional sarcoplasmic reticulum membrane | 2 | 491 | 8 | 17318 | 0.029674597 | 0.261136456 | 8.8177189 | 5.074627738 |
| GO:0008180 | COP9 signalosome | 4 | 491 | 39 | 17318 | 0.03075047 | 0.264004033 | 3.6175257 | 5.023247741 |

| | | | | | | | | | |
|---|---|---|---|---|---|---|---|---|---|
| GO:0005789 | endoplasmic reticulum membrane | 34 | 491 | 835 | 17318 | 0.03282758 | 0.275126389 | 1.4361794 | 4.928947768 |
| GO:0031588 | AMP-activated protein kinase complex | 2 | 491 | 9 | 17318 | 0.035611368 | 0.291516314 | 7.8379724 | 4.811518336 |
| GO:0031966 | mitochondrial membrane | 8 | 491 | 129 | 17318 | 0.037692249 | 0.30153799 | 2.1873411 | 4.729588318 |
| GO:0097225 | sperm midpiece | 2 | 491 | 10 | 17318 | 0.041960791 | 0.314406485 | 7.0541752 | 4.57481432 |
| GO:0005905 | coated pit | 5 | 491 | 64 | 17318 | 0.042511081 | 0.314406485 | 2.7555372 | 4.55601725 |
| GO:0043204 | perikaryon | 5 | 491 | 65 | 17318 | 0.044773399 | 0.314406485 | 2.7131443 | 4.481214355 |
| GO:0030425 | dendrite | 14 | 491 | 290 | 17318 | 0.046526958 | 0.314406485 | 1.7027319 | 4.425789324 |
| GO:0014731 | spectrin-associated cytoskeleton | 2 | 491 | 11 | 17318 | 0.048695438 | 0.314406485 | 6.4128865 | 4.36006958 |
| GO:0008091 | spectrin | 2 | 491 | 11 | 17318 | 0.048695438 | 0.314406485 | 6.4128865 | 4.36006958 |

**Table S5: Selected significant enrichment GO terms**

| GOID | GO Term | DifGene | AllDifGene | GeneInGO | AllGene | P-Value | FDR | Enrichment | (-log2P) |
|---|---|---|---|---|---|---|---|---|---|
| GO:0044255 | cellular lipid metabolic process | 24 | 834 | 164 | 16650 | 1.45E-05 | 0.02750197 | 2.921565187 | 16.07802992 |
| GO:0044281 | small molecule metabolic process | 120 | 834 | 1537 | 16650 | 1.75E-05 | 0.02750197 | 1.558674986 | 15.80499925 |
| GO:0045214 | sarcomere organization | 9 | 834 | 35 | 16650 | 2.05E-04 | 0.10380211 | 5.1336074 | 12.25400353 |
| GO:0005975 | carbohydrate metabolic process | 41 | 834 | 431 | 16650 | 2.53E-04 | 0.10380211 | 1.899130348 | 11.94659673 |
| GO:0006006 | glucose metabolic process | 18 | 834 | 129 | 16650 | 2.64E-04 | 0.10380211 | 2.785678434 | 11.88877035 |
| GO:0046627 | negative regulation of insulin receptor signaling pathway | 8 | 834 | 29 | 16650 | 3.06E-04 | 0.10722602 | 5.507318283 | 11.67202612 |
| GO:0030049 | muscle filament sliding | 9 | 834 | 39 | 16650 | 4.07E-04 | 0.12818052 | 4.607083564 | 11.26250099 |
| GO:0006470 | protein dephosphorylation | 22 | 834 | 190 | 16650 | 6.30E-04 | 0.1804448 | 2.311624385 | 10.63161699 |
| GO:0060070 | canonical Wnt signaling pathway | 13 | 834 | 88 | 16650 | 0.001106572 | 0.22354562 | 2.949231524 | 9.819687348 |
| GO:0055114 | oxidation-reduction process | 62 | 834 | 790 | 16650 | 0.001109917 | 0.22354562 | 1.566797195 | 9.815332834 |
| GO:0035567 | non-canonical Wnt signaling pathway | 6 | 834 | 20 | 16650 | 0.00120682 | 0.22354562 | 5.989208633 | 9.69457405 |
| GO:0006099 | tricarboxylic acid cycle | 7 | 834 | 32 | 16650 | 0.002291162 | 0.31037559 | 4.367131295 | 8.769704683 |
| GO:0006094 | gluconeogenesis | 8 | 834 | 42 | 16650 | 0.002445764 | 0.31037559 | 3.802672148 | 8.675498796 |
| GO:0032868 | response to insulin | 11 | 834 | 74 | 16650 | 0.002464081 | 0.31037559 | 2.967625899 | 8.664734845 |
| GO:0006936 | muscle contraction | 14 | 834 | 111 | 16650 | 0.00278151 | 0.33688365 | 2.517985612 | 8.489915998 |
| GO:0046034 | ATP metabolic process | 7 | 834 | 35 | 16650 | 0.003544872 | 0.34883757 | 3.992805755 | 8.140050697 |
| GO:0005978 | glycogen biosynthetic process | 6 | 834 | 26 | 16650 | 0.003712011 | 0.35421582 | 4.607083564 | 8.07358338 |
| GO:0046676 | negative regulation of insulin secretion | 7 | 834 | 36 | 16650 | 0.004061063 | 0.37612614 | 3.881894484 | 7.943926734 |
| GO:0006768 | biotin metabolic process | 4 | 834 | 11 | 16650 | 0.004665504 | 0.40810202 | 7.259646828 | 7.7437513 |

| | | | | | | | | | |
|---|---|---|---|---|---|---|---|---|---|
| GO:0006090 | pyruvate metabolic process | 6 | 834 | 28 | 16650 | 0.005076334 | 0.4313915 | 4.278006166 | 7.621997312 |
| GO:0008285 | negative regulation of cell proliferation | 35 | 834 | 433 | 16650 | 0.007306876 | 0.4313915 | 1.613720571 | 7.096529647 |
| GO:0046498 | S-adenosylhomocysteine metabolic process | 3 | 834 | 6 | 16650 | 0.007380301 | 0.4313915 | 9.982014388 | 7.082104607 |
| GO:0042693 | muscle cell fate commitment | 3 | 834 | 6 | 16650 | 0.007380301 | 0.4313915 | 9.982014388 | 7.082104607 |
| GO:0097193 | intrinsic apoptotic signaling pathway | 10 | 834 | 75 | 16650 | 0.007462625 | 0.4313915 | 2.661870504 | 7.066101071 |
| GO:0045780 | positive regulation of bone resorption | 4 | 834 | 14 | 16650 | 0.009324254 | 0.46606467 | 5.704008222 | 6.744796048 |
| GO:0043462 | regulation of ATPase activity | 3 | 834 | 7 | 16650 | 0.010170237 | 0.4709717 | 8.556012333 | 6.619502927 |
| GO:0032259 | methylation | 19 | 834 | 206 | 16650 | 0.012708722 | 0.47604647 | 1.84134246 | 6.298037287 |
| GO:0031444 | slow-twitch skeletal muscle fiber contraction | 2 | 834 | 2 | 16650 | 0.012840427 | 0.47604647 | 19.96402878 | 6.283163026 |
| GO:1901741 | positive regulation of myoblast fusion | 4 | 834 | 16 | 16650 | 0.013680466 | 0.47604647 | 4.991007194 | 6.191738805 |
| GO:0030308 | negative regulation of cell growth | 13 | 834 | 124 | 16650 | 0.014778677 | 0.47604647 | 2.093003017 | 6.080339042 |
| GO:0005977 | glycogen metabolic process | 6 | 834 | 37 | 16650 | 0.015963438 | 0.47604647 | 3.237410072 | 5.969084754 |
| GO:0070848 | response to growth factor | 4 | 834 | 17 | 16650 | 0.016269651 | 0.47604647 | 4.697418536 | 5.941672917 |
| GO:0045944 | positive regulation of transcription from RNA polymerase II promoter | 66 | 834 | 978 | 16650 | 0.016771994 | 0.47604647 | 1.347265746 | 5.897801962 |
| GO:0071340 | skeletal muscle acetylcholine-gated channel clustering | 3 | 834 | 9 | 16650 | 0.01735447 | 0.47604647 | 6.654676259 | 5.848548862 |
| GO:0006767 | water-soluble vitamin metabolic process | 10 | 834 | 87 | 16650 | 0.017928102 | 0.47604647 | 2.294715951 | 5.801633429 |
| GO:0030178 | negative regulation of Wnt signaling pathway | 7 | 834 | 50 | 16650 | 0.018658473 | 0.47604647 | 2.794964029 | 5.744025307 |
| GO:0044237 | cellular metabolic process | 17 | 834 | 186 | 16650 | 0.018925356 | 0.47604647 | 1.824669297 | 5.723535745 |
| GO:0007517 | muscle organ development | 11 | 834 | 102 | 16650 | 0.019915735 | 0.47604647 | 2.152983496 | 5.649947473 |
| GO:0071400 | cellular response to oleic acid | 2 | 834 | 3 | 16650 | 0.020723529 | 0.47604647 | 13.30935252 | 5.592586479 |
| GO:0006542 | glutamine biosynthetic process | 2 | 834 | 3 | 16650 | 0.020723529 | 0.47604647 | 13.30935252 | 5.592586479 |
| GO:0061056 | sclerotome development | 2 | 834 | 3 | 16650 | 0.020723529 | 0.47604647 | 13.30935252 | 5.592586479 |
| GO:0014733 | regulation of skeletal muscle adaptation | 2 | 834 | 3 | 16650 | 0.020723529 | 0.47604647 | 13.30935252 | 5.592586479 |
| GO:0006982 | response to lipid hydroperoxide | 2 | 834 | 3 | 16650 | 0.020723529 | 0.47604647 | 13.30935252 | 5.592586479 |
| GO:0051216 | cartilage development | 9 | 834 | 78 | 16650 | 0.023468224 | 0.49586926 | 2.303541782 | 5.41314753 |
| GO:0006941 | striated muscle contraction | 4 | 834 | 20 | 16650 | 0.025787344 | 0.49586926 | 3.992805755 | 5.277192979 |
| GO:0015909 | long-chain fatty acid transport | 3 | 834 | 11 | 16650 | 0.026736389 | 0.49586926 | 5.444735121 | 5.22505155 |
| GO:0001649 | osteoblast differentiation | 11 | 834 | 108 | 16650 | 0.027916742 | 0.49586926 | 2.033373301 | 5.162725628 |
| GO:0006766 | vitamin metabolic process | 10 | 834 | 95 | 16650 | 0.02925646 | 0.49586926 | 2.101476713 | 5.095100997 |
| GO:0032909 | regulation of transforming growth factor beta2 production | 2 | 834 | 4 | 16650 | 0.030105841 | 0.49586926 | 9.982014388 | 5.053812787 |
| GO:0045725 | positive regulation of glycogen biosynthetic process | 3 | 834 | 12 | 16650 | 0.03225411 | 0.49586926 | 4.991007194 | 4.954373161 |
| GO:0006096 | glycolytic process | 6 | 834 | 45 | 16650 | 0.034192448 | 0.49586926 | 2.661870504 | 4.870178479 |

| | | | | | | | | | |
|---|---|---|---|---|---|---|---|---|---|
| GO:0016055 | Wnt signaling pathway | 17 | 834 | 202 | 16650 | 0.035611376 | 0.49586926 | 1.680141036 | 4.811518026 |
| GO:0051726 | regulation of cell cycle | 14 | 834 | 157 | 16650 | 0.036290042 | 0.49586926 | 1.780231865 | 4.784282482 |
| GO:0090090 | negative regulation of canonical Wnt signaling pathway | 14 | 834 | 158 | 16650 | 0.037823342 | 0.49586926 | 1.768964575 | 4.724579357 |
| GO:0006664 | glycolipid metabolic process | 2 | 834 | 5 | 16650 | 0.040825846 | 0.49586926 | 7.985611511 | 4.614373397 |
| GO:0019626 | short-chain fatty acid catabolic process | 2 | 834 | 5 | 16650 | 0.040825846 | 0.49586926 | 7.985611511 | 4.614373397 |
| GO:0080009 | mRNA methylation | 2 | 834 | 5 | 16650 | 0.040825846 | 0.49586926 | 7.985611511 | 4.614373397 |
| GO:0038031 | non-canonical Wnt signaling pathway via JNK cascade | 2 | 834 | 5 | 16650 | 0.040825846 | 0.49586926 | 7.985611511 | 4.614373397 |
| GO:0044259 | multicellular organismal macromolecule metabolic process | 2 | 834 | 5 | 16650 | 0.040825846 | 0.49586926 | 7.985611511 | 4.614373397 |
| GO:0043568 | positive regulation of insulin-like growth factor receptor signaling pathway | 3 | 834 | 14 | 16650 | 0.044911818 | 0.49586926 | 4.278006166 | 4.476761068 |
| GO:0030239 | myofibril assembly | 3 | 834 | 14 | 16650 | 0.044911818 | 0.49586926 | 4.278006166 | 4.476761068 |
| GO:0001558 | regulation of cell growth | 8 | 834 | 75 | 16650 | 0.04513733 | 0.49586926 | 2.129496403 | 4.469535111 |
| GO:0008652 | cellular amino acid biosynthetic process | 8 | 834 | 35 | 16650 | 8.90E-04 | 0.21562757 | 4.563206578 | 10.13362475 |
| GO:0006631 | fatty acid metabolic process | 18 | 834 | 155 | 16650 | 0.001787843 | 0.27157107 | 2.318403342 | 9.127564089 |
| GO:0007519 | skeletal muscle tissue development | 10 | 834 | 60 | 16650 | 0.001811049 | 0.27157107 | 3.327338129 | 9.108958942 |
| GO:0045444 | fat cell differentiation | 11 | 834 | 73 | 16650 | 0.002241236 | 0.31037559 | 3.008278309 | 8.801489834 |
| GO:0051149 | positive regulation of muscle cell differentiation | 6 | 834 | 29 | 16650 | 0.005881487 | 0.4313915 | 4.130488712 | 7.409603254 |
| GO:0048741 | skeletal muscle fiber development | 6 | 834 | 29 | 16650 | 0.005881487 | 0.4313915 | 4.130488712 | 7.409603254 |
| GO:0040008 | regulation of growth | 10 | 834 | 74 | 16650 | 0.006874561 | 0.4313915 | 2.697841727 | 7.184516686 |
| GO:0035914 | skeletal muscle cell differentiation | 8 | 834 | 51 | 16650 | 0.006901635 | 0.4313915 | 3.131612357 | 7.178846058 |
| GO:0045663 | positive regulation of myoblast differentiation | 5 | 834 | 21 | 16650 | 0.007088716 | 0.4313915 | 4.753340185 | 7.140259904 |
| GO:0048743 | positive regulation of skeletal muscle fiber development | 3 | 834 | 6 | 16650 | 0.007380301 | 0.4313915 | 9.982014388 | 7.082104607 |
| GO:0006635 | fatty acid beta-oxidation | 7 | 834 | 42 | 16650 | 0.008434865 | 0.45795498 | 3.327338129 | 6.889419368 |
| GO:0042692 | muscle cell differentiation | 6 | 834 | 39 | 16650 | 0.019674276 | 0.47604647 | 3.071389043 | 5.667545632 |
| GO:0006629 | lipid metabolic process | 38 | 834 | 530 | 16650 | 0.026824273 | 0.49586926 | 1.431383195 | 5.220317107 |
| GO:0071398 | cellular response to fatty acid | 4 | 834 | 21 | 16650 | 0.029564442 | 0.49586926 | 3.802672148 | 5.079993145 |
| GO:0003009 | skeletal muscle contraction | 6 | 834 | 25 | 16650 | 0.003142168 | 0.34700159 | 4.791366906 | 8.31402415 |
| GO:0070542 | response to fatty acid | 5 | 834 | 21 | 16650 | 0.007088716 | 0.4313915 | 4.753340185 | 7.140259904 |
| GO:0008152 | metabolic process | 129 | 834 | 2068 | 16650 | 0.015372461 | 0.47604647 | 1.245338352 | 6.023508017 |
| GO:0045445 | myoblast differentiation | 4 | 834 | 20 | 16650 | 0.025787344 | 0.49586926 | 3.992805755 | 5.277192979 |
| GO:0045926 | negative regulation of growth | 3 | 834 | 14 | 16650 | 0.044911818 | 0.49586926 | 4.278006166 | 4.476761068 |
| GO:0007520 | myoblast fusion | 4 | 834 | 25 | 16650 | 0.047783458 | 0.49586926 | 3.194244604 | 4.387344914 |

## Table S6: Pathway-Analysis

| Significant pathways for all-DEGs | | | | | | | | | |
|---|---|---|---|---|---|---|---|---|---|
| **Pathway ID** | **Pathway Term** | **DifGene** | **AllDifGene** | **GeneInPathway** | **AllGene** | **P-Value** | **FDR** | **Enrichment** | **(-log2P)** |
| PATH:04068 | FoxO signaling pathway | 19 | 392 | 127 | 7262 | 0.00022203 | 0.0363803 | 2.771533 | 12.13695996 |
| PATH:01230 | Biosynthesis of amino acids | 13 | 392 | 72 | 7262 | 0.000429548 | 0.0363803 | 3.3448838 | 11.18489255 |
| PATH:01200 | Carbon metabolism | 16 | 392 | 107 | 7262 | 0.000657793 | 0.0363803 | 2.7701698 | 10.570078 |
| PATH:01100 | Metabolic pathways | 97 | 392 | 1210 | 7262 | 0.000691127 | 0.0363803 | 1.4851029 | 10.49876114 |
| PATH:00062 | Fatty acid elongation | 7 | 392 | 23 | 7262 | 0.000694281 | 0.0363803 | 5.6381988 | 10.49219236 |
| PATH:05414 | Dilated cardiomyopathy | 14 | 392 | 89 | 7262 | 0.000889975 | 0.0388622 | 2.9141252 | 10.13394775 |
| PATH:00620 | Pyruvate metabolism | 9 | 392 | 42 | 7262 | 0.001109029 | 0.0415094 | 3.9697522 | 9.816487371 |
| PATH:00330 | Arginine and proline metabolism | 11 | 392 | 62 | 7262 | 0.001293543 | 0.0423635 | 3.2867841 | 9.594456793 |
| PATH:04310 | Wnt signaling pathway | 19 | 392 | 152 | 7262 | 0.001540636 | 0.0448496 | 2.3156888 | 9.34225859 |
| PATH:00280 | Valine, leucine and isoleucine degradation | 9 | 392 | 48 | 7262 | 0.002476346 | 0.0608216 | 3.4735332 | 8.657571269 |
| PATH:00910 | Nitrogen metabolism | 6 | 392 | 22 | 7262 | 0.002650787 | 0.0608216 | 5.0524119 | 8.559363565 |
| PATH:00020 | Citrate cycle (TCA cycle) | 7 | 392 | 31 | 7262 | 0.002989627 | 0.0608216 | 4.1831797 | 8.385819004 |
| PATH:05410 | Hypertrophic cardiomyopathy (HCM) | 12 | 392 | 81 | 7262 | 0.003085958 | 0.0608216 | 2.74452 | 8.340065794 |
| PATH:04921 | Oxytocin signaling pathway | 18 | 392 | 152 | 7262 | 0.003366237 | 0.0608216 | 2.1938104 | 8.214647659 |
| PATH:00640 | Propanoate metabolism | 7 | 392 | 32 | 7262 | 0.003482155 | 0.0608216 | 4.0524554 | 8.165803714 |
| PATH:00270 | Cysteine and methionine metabolism | 8 | 392 | 42 | 7262 | 0.003874049 | 0.0634376 | 3.5286686 | 8.011942005 |
| PATH:04910 | Insulin signaling pathway | 17 | 392 | 143 | 7262 | 0.004120597 | 0.0635057 | 2.2023334 | 7.922930787 |
| PATH:05166 | HTLV-I infection | 26 | 392 | 264 | 7262 | 0.005276138 | 0.0767971 | 1.8244821 | 7.566301936 |
| PATH:00480 | Glutathione metabolism | 9 | 392 | 56 | 7262 | 0.006063778 | 0.0836163 | 2.9773141 | 7.365567442 |
| PATH:01212 | Fatty acid metabolism | 8 | 392 | 47 | 7262 | 0.006985755 | 0.0880247 | 3.1532783 | 7.16136823 |
| PATH:04261 | Adrenergic signaling in cardiomyocytes | 17 | 392 | 152 | 7262 | 0.007055418 | 0.0880247 | 2.0719321 | 7.1470528 |
| PATH:04962 | Vasopressin-regulated water reabsorption | 8 | 392 | 48 | 7262 | 0.007785867 | 0.0927226 | 3.087585 | 7.004926644 |
| PATH:05031 | Amphetamine addiction | 10 | 392 | 72 | 7262 | 0.009634109 | 0.1097451 | 2.5729875 | 6.697633028 |
| PATH:05205 | Proteoglycans in cancer | 22 | 392 | 225 | 7262 | 0.010135453 | 0.1106454 | 1.8113832 | 6.624445601 |

| | | | | | | | | | |
|---|---|---|---|---|---|---|---|---|---|
| PATH:04066 | HIF-1 signaling pathway | 13 | 392 | 109 | 7262 | 0.01070858 | 0.1122259 | 2.2094645 | 6.54508898 |
| PATH:04530 | Tight junction | 15 | 392 | 136 | 7262 | 0.012014162 | 0.1168326 | 2.0432548 | 6.379120129 |
| PATH:00630 | Glyoxylate and dicarboxylate metabolism | 6 | 392 | 32 | 7262 | 0.012485928 | 0.1168326 | 3.4735332 | 6.323553185 |
| PATH:00410 | beta-Alanine metabolism | 6 | 392 | 32 | 7262 | 0.012485928 | 0.1168326 | 3.4735332 | 6.323553185 |
| PATH:00650 | Butanoate metabolism | 6 | 392 | 33 | 7262 | 0.014122977 | 0.1275938 | 3.3682746 | 6.145811953 |
| PATH:00860 | Porphyrin and chlorophyll metabolism | 7 | 392 | 44 | 7262 | 0.015277712 | 0.1334254 | 2.9472403 | 6.032427676 |
| PATH:05416 | Viral myocarditis | 10 | 392 | 79 | 7262 | 0.016535257 | 0.1397496 | 2.3450013 | 5.918310694 |
| PATH:01210 | 2-Oxocarboxylic acid metabolism | 4 | 392 | 16 | 7262 | 0.017519893 | 0.1415804 | 4.6313776 | 5.834862244 |
| PATH:00250 | Alanine, aspartate and glutamate metabolism | 6 | 392 | 35 | 7262 | 0.017832641 | 0.1415804 | 3.1758017 | 5.809335838 |
| PATH:04930 | Type II diabetes mellitus | 7 | 392 | 49 | 7262 | 0.02449677 | 0.1887692 | 2.6465015 | 5.351264669 |
| PATH:05321 | Inflammatory bowel disease (IBD) | 8 | 392 | 61 | 7262 | 0.025407738 | 0.1901951 | 2.4295751 | 5.298588259 |
| PATH:05220 | Chronic myeloid leukemia | 9 | 392 | 74 | 7262 | 0.027248445 | 0.1983081 | 2.2531026 | 5.197682288 |
| PATH:00010 | Glycolysis / Gluconeogenesis | 8 | 392 | 63 | 7262 | 0.02954625 | 0.2092194 | 2.3524457 | 5.080881131 |
| PATH:04916 | Melanogenesis | 11 | 392 | 101 | 7262 | 0.030346366 | 0.2092302 | 2.0176298 | 5.042332444 |
| PATH:00512 | Mucin type O-Glycan biosynthesis | 5 | 392 | 31 | 7262 | 0.036049138 | 0.2421763 | 2.9879855 | 4.793891415 |
| PATH:04110 | Cell cycle | 13 | 392 | 132 | 7262 | 0.037930913 | 0.2484475 | 1.8244821 | 4.720482094 |
| PATH:04932 | Non-alcoholic fatty liver disease (NAFLD) | 16 | 392 | 176 | 7262 | 0.041630084 | 0.2610784 | 1.6841373 | 4.586229713 |
| PATH:04920 | Adipocytokine signaling pathway | 8 | 392 | 68 | 7262 | 0.041852263 | 0.2610784 | 2.1794718 | 4.578550556 |
| PATH:04144 | Endocytosis | 18 | 392 | 207 | 7262 | 0.045296254 | 0.2744738 | 1.6109139 | 4.464464459 |
| PATH:04913 | Ovarian steroidogenesis | 7 | 392 | 57 | 7262 | 0.046094837 | 0.2744738 | 2.2750627 | 4.439251034 |
| PATH:01040 | Biosynthesis of unsaturated fatty acids | 4 | 392 | 23 | 7262 | 0.047740739 | 0.2779572 | 3.2218279 | 4.388635285 |
| **Significant pathways for up-regulated DEGs** | | | | | | | | | |
| **Pathway ID** | **Pathway Term** | **DifGene** | **AllDifGene** | **GeneInPathway** | **AllGene** | **P-Value** | **FDR** | **Enrichment** | **(-log2P)** |
| PATH:04068 | FoxO signaling pathway | 11 | 163 | 127 | 7262 | 0.00031908 | 0.0644542 | 3.8588474 | 11.61379285 |
| PATH:00910 | Nitrogen metabolism | 4 | 163 | 22 | 7262 | 0.002475526 | 0.2500282 | 8.1003904 | 8.658048999 |
| PATH:00603 | Glycosphingolipid biosynthesis - globo series | 3 | 163 | 15 | 7262 | 0.006948786 | 0.3830132 | 8.9104294 | 7.169023372 |
| PATH:00630 | Glyoxylate and dicarboxylate metabolism | 4 | 163 | 32 | 7262 | 0.008162712 | 0.3830132 | 5.5690184 | 6.936735694 |
| PATH:04978 | Mineral absorption | 5 | 163 | 55 | 7262 | 0.010841303 | 0.3830132 | 4.0501952 | 6.527318013 |
| PATH:04115 | p53 signaling pathway | 6 | 163 | 78 | 7262 | 0.011376628 | 0.3830132 | 3.4270882 | 6.457783134 |
| PATH:04066 | HIF-1 signaling pathway | 7 | 163 | 109 | 7262 | 0.015614122 | 0.4254953 | 2.8611471 | 6.001004716 |

| | | | | | | | | | |
|---|---|---|---|---|---|---|---|---|---|
| PATH:00330 | Arginine and proline metabolism | 5 | 163 | 62 | 7262 | 0.01686245 | 0.4254953 | 3.5929151 | 5.890042036 |
| PATH:00270 | Cysteine and methionine metabolism | 4 | 163 | 42 | 7262 | 0.018957709 | 0.4254953 | 4.2430616 | 5.72107154 |
| PATH:00860 | Porphyrin and chlorophyll metabolism | 4 | 163 | 44 | 7262 | 0.021826415 | 0.4408936 | 4.0501952 | 5.517781003 |
| PATH:01230 | Biosynthesis of amino acids | 5 | 163 | 72 | 7262 | 0.028757276 | 0.5066966 | 3.0938991 | 5.11992915 |
| PATH:04930 | Type II diabetes mellitus | 4 | 163 | 49 | 7262 | 0.03010079 | 0.5066966 | 3.63691 | 5.054054851 |
| PATH:04110 | Cell cycle | 7 | 163 | 132 | 7262 | 0.036832264 | 0.5495287 | 2.3626139 | 4.762886121 |
| PATH:04140 | Regulation of autophagy | 3 | 163 | 31 | 7262 | 0.039130914 | 0.5495287 | 4.3114981 | 4.675547394 |
| PATH:05221 | Acute myeloid leukemia | 4 | 163 | 58 | 7262 | 0.049061965 | 0.5495287 | 3.0725619 | 4.349251185 |
| **Significant pathways for down-regulated DEGs** | | | | | | | | | |
| **Pathway ID** | **Pathway Term** | **DifGene** | **AllDifGene** | **GeneInPathway** | **AllGene** | **P-Value** | **FDR** | **Enrichment** | **(-log2P)** |
| PATH:05414 | Dilated cardiomyopathy | 14 | 229 | 89 | 7262 | 4.41109E-06 | 0.000838 | 4.9883715 | 17.79043263 |
| PATH:01200 | Carbon metabolism | 15 | 229 | 107 | 7262 | 7.25532E-06 | 0.000838 | 4.4455781 | 17.0725302 |
| PATH:00620 | Pyruvate metabolism | 9 | 229 | 42 | 7262 | 2.54989E-05 | 0.0019634 | 6.7953837 | 15.25920525 |
| PATH:05410 | Hypertrophic cardiomyopathy (HCM) | 12 | 229 | 81 | 7262 | 3.42601E-05 | 0.0019785 | 4.698043 | 14.83310944 |
| PATH:04921 | Oxytocin signaling pathway | 16 | 229 | 152 | 7262 | 8.80605E-05 | 0.0040684 | 3.3380832 | 13.47114494 |
| PATH:00020 | Citrate cycle (TCA cycle) | 7 | 229 | 31 | 7262 | 0.00014958 | 0.0057588 | 7.1607269 | 12.70679469 |
| PATH:00640 | Propanoate metabolism | 7 | 229 | 32 | 7262 | 0.000177353 | 0.0058526 | 6.9369541 | 12.46108891 |
| PATH:04261 | Adrenergic signaling in cardiomyocytes | 15 | 229 | 152 | 7262 | 0.000268216 | 0.0077447 | 3.129453 | 11.86431618 |
| PATH:01100 | Metabolic pathways | 63 | 229 | 1210 | 7262 | 0.000615145 | 0.0157887 | 1.6511097 | 10.66678583 |
| PATH:04310 | Wnt signaling pathway | 14 | 229 | 152 | 7262 | 0.000776147 | 0.017929 | 2.9208228 | 10.33138179 |
| PATH:05031 | Amphetamine addiction | 9 | 229 | 72 | 7262 | 0.000934366 | 0.0196217 | 3.9639738 | 10.06372453 |
| PATH:00650 | Butanoate metabolism | 6 | 229 | 33 | 7262 | 0.001191792 | 0.022942 | 5.7657801 | 9.712651515 |
| PATH:00280 | Valine, leucine and isoleucine degradation | 7 | 229 | 48 | 7262 | 0.001510353 | 0.0246552 | 4.6246361 | 9.370898519 |
| PATH:00062 | Fatty acid elongation | 5 | 229 | 23 | 7262 | 0.001529587 | 0.0246552 | 6.8938675 | 9.35264218 |
| PATH:00010 | Glycolysis / Gluconeogenesis | 8 | 229 | 63 | 7262 | 0.001600989 | 0.0246552 | 4.026894 | 9.28682079 |
| PATH:04916 | Melanogenesis | 10 | 229 | 101 | 7262 | 0.002491548 | 0.0359717 | 3.1397812 | 8.648741651 |
| PATH:01210 | 2-Oxocarboxylic acid metabolism | 4 | 229 | 16 | 7262 | 0.002955335 | 0.0391499 | 7.9279476 | 8.402462463 |
| PATH:04260 | Cardiac muscle contraction | 9 | 229 | 87 | 7262 | 0.003050641 | 0.0391499 | 3.28053 | 8.356671908 |
| PATH:01230 | Biosynthesis of amino acids | 8 | 229 | 72 | 7262 | 0.003403377 | 0.0413779 | 3.5235323 | 8.198817493 |
| PATH:05321 | Inflammatory bowel disease (IBD) | 7 | 229 | 61 | 7262 | 0.005073821 | 0.0586026 | 3.6390579 | 7.622711569 |
| PATH:05416 | Viral myocarditis | 8 | 229 | 79 | 7262 | 0.005661753 | 0.0607335 | 3.2113205 | 7.464535466 |
| PATH:01212 | Fatty acid metabolism | 6 | 229 | 47 | 7262 | 0.005784143 | 0.0607335 | 4.0483137 | 7.433681146 |

| | | | | | | | | | |
|---|---|---|---|---|---|---|---|---|---|
| PATH:04962 | Vasopressin-regulated water reabsorption | 6 | 229 | 48 | 7262 | 0.006337848 | 0.063654 | 3.9639738 | 7.301791209 |
| PATH:00980 | Metabolism of xenobiotics by cytochrome P450 | 9 | 229 | 100 | 7262 | 0.006989836 | 0.0672772 | 2.8540611 | 7.160525642 |
| PATH:05166 | HTLV-I infection | 17 | 229 | 264 | 7262 | 0.007729651 | 0.071422 | 2.0420471 | 7.01538092 |
| PATH:01040 | Biosynthesis of unsaturated fatty acids | 4 | 229 | 23 | 7262 | 0.009005706 | 0.0800122 | 5.515094 | 6.794944983 |
| PATH:00480 | Glutathione metabolism | 6 | 229 | 56 | 7262 | 0.012227101 | 0.1046096 | 3.3976918 | 6.353773749 |
| PATH:04971 | Gastric acid secretion | 7 | 229 | 74 | 7262 | 0.012840276 | 0.1059323 | 2.999764 | 6.283180005 |
| PATH:04932 | Non-alcoholic fatty liver disease (NAFLD) | 12 | 229 | 176 | 7262 | 0.015089253 | 0.1201937 | 2.1621675 | 6.050334844 |
| PATH:05205 | Proteoglycans in cancer | 14 | 229 | 225 | 7262 | 0.018317833 | 0.1387104 | 1.9731781 | 5.770607353 |
| PATH:00330 | Arginine and proline metabolism | 6 | 229 | 62 | 7262 | 0.018614814 | 0.1387104 | 3.0688829 | 5.747404981 |
| PATH:04020 | Calcium signaling pathway | 12 | 229 | 184 | 7262 | 0.020154082 | 0.1454873 | 2.0681602 | 5.632784137 |
| PATH:04910 | Insulin signaling pathway | 10 | 229 | 143 | 7262 | 0.021446507 | 0.1501255 | 2.2176077 | 5.543113494 |
| PATH:00410 | beta-Alanine metabolism | 4 | 229 | 32 | 7262 | 0.024261045 | 0.1648324 | 3.9639738 | 5.365214514 |
| PATH:05030 | Cocaine addiction | 5 | 229 | 50 | 7262 | 0.027158166 | 0.1792439 | 3.171179 | 5.20247015 |
| PATH:04720 | Long-term potentiation | 6 | 229 | 69 | 7262 | 0.028559244 | 0.1832551 | 2.757547 | 5.129898418 |
| PATH:04728 | Dopaminergic synapse | 9 | 229 | 132 | 7262 | 0.031894816 | 0.1991271 | 2.1621675 | 4.970534227 |
| PATH:04530 | Tight junction | 9 | 229 | 136 | 7262 | 0.037082021 | 0.2254197 | 2.0985744 | 4.753136296 |
| PATH:04913 | Ovarian steroidogenesis | 5 | 229 | 57 | 7262 | 0.042384731 | 0.247249 | 2.781736 | 4.560311556 |
| PATH:04514 | Cell adhesion molecules (CAMs) | 9 | 229 | 140 | 7262 | 0.042813678 | 0.247249 | 2.0386151 | 4.54578442 |
| PATH:04114 | Oocyte meiosis | 8 | 229 | 119 | 7262 | 0.044095242 | 0.248439 | 2.1318851 | 4.503233193 |
| PATH:04022 | cGMP-PKG signaling pathway | 10 | 229 | 164 | 7262 | 0.045254693 | 0.2489008 | 1.9336458 | 4.465788769 |
| PATH:05310 | Asthma | 3 | 229 | 24 | 7262 | 0.049215245 | 0.2604091 | 3.9639738 | 4.344750927 |
| PATH:05012 | Parkinson's disease | 10 | 229 | 167 | 7262 | 0.049702796 | 0.2604091 | 1.8989096 | 4.330529181 |

## Table S7: Selected significant enrichment pathways

| Pathway ID | Pathway Term | DifGene | AllDifGene | GeneInPathway | AllGene | P-Value | FDR | Enrichment | (-log2P) | First Grade | Second Grade |
|---|---|---|---|---|---|---|---|---|---|---|---|
| PATH:00910 | Nitrogen metabolism | 6 | 407 | 23 | 7932 | 0.002512232 | 0.040786826 | 5.084072215 | 8.636814561 | Metabolism | Energy metabolism |
| PATH:00650 | Butanoate metabolism | 6 | 407 | 34 | 7932 | 0.012783331 | 0.106915135 | 3.439225322 | 6.289592335 | Metabolism | Carbohydrate metabolism |
| PATH:04068 | FoxO signaling pathway | 20 | 407 | 130 | 7932 | 0.000058 | 0.00800911 | 2.998298998 | 14.07266688 | Environmental Information Processing | Signal transduction |
| PATH:01100 | Metabolic pathways | 106 | 407 | 1343 | 7932 | 0.000145 | 0.012953481 | 1.53821892 | 12.75043041 | Metabolism | Global and overview maps |
| PATH:04310 | Wnt signaling pathway | 21 | 407 | 155 | 7932 | 0.000191 | 0.012953481 | 2.640437505 | 12.35622307 | Environmental Information Processing | Signal transduction |
| PATH:00620 | Pyruvate metabolism | 10 | 407 | 43 | 7932 | 0.000235 | 0.012953481 | 4.532312439 | 12.05711269 | Metabolism | Carbohydrate metabolism |
| PATH:01230 | Biosynthesis of amino acids | 13 | 407 | 79 | 7932 | 0.000592 | 0.021193845 | 3.207041334 | 10.72275846 | Metabolism | Global and overview maps |
| PATH:00020 | Citrate cycle (TCA cycle) | 8 | 407 | 33 | 7932 | 0.000763 | 0.023411736 | 4.724592361 | 10.35522372 | Metabolism | Carbohydrate metabolism |
| PATH:00062 | Fatty acid elongation | 7 | 407 | 27 | 7932 | 0.001155711 | 0.028997828 | 5.052689053 | 9.757004195 | Metabolism | Lipid metabolism |
| PATH:04910 | Insulin signaling pathway | 18 | 407 | 146 | 7932 | 0.001356689 | 0.031203848 | 2.402746458 | 9.525694209 | Organismal Systems | Endocrine system |
| PATH:00330 | Arginine and proline metabolism | 11 | 407 | 67 | 7932 | 0.001525076 | 0.032378531 | 3.199677289 | 9.356903402 | Metabolism | Amino acid metabolism |
| PATH:00280 | Valine, leucine and isoleucine degradation | 9 | 407 | 53 | 7932 | 0.003222479 | 0.047561757 | 3.309443234 | 8.277613394 | Metabolism | Amino acid metabolism |
| PATH:00640 | Propanoate metabolism | 7 | 407 | 36 | 7932 | 0.004692965 | 0.061678969 | 3.78951679 | 7.735284564 | Metabolism | Carbohydrate metabolism |
| PATH:00410 | beta-Alanine metabolism | 7 | 407 | 37 | 7932 | 0.005346127 | 0.064259934 | 3.687097417 | 7.547290192 | Metabolism | Metabolism of other amino acids |
| PATH:00480 | Glutathione metabolism | 9 | 407 | 58 | 7932 | 0.00544401 | 0.064259934 | 3.024146403 | 7.521114639 | Metabolism | Metabolism of other amino acids |
| PATH:00270 | Cysteine and methionine metabolism | 8 | 407 | 50 | 7932 | 0.007268888 | 0.077162045 | 3.118230958 | 7.104049556 | Metabolism | Amino acid metabolism |
| PATH:01212 | Fatty acid metabolism | 8 | 407 | 51 | 7932 | 0.008051403 | 0.079363834 | 3.057089175 | 6.956544006 | Metabolism | Global and overview maps |
| PATH:04530 | Tight junction | 15 | 407 | 143 | 7932 | 0.011795263 | 0.105015892 | 2.044294772 | 6.405648573 | Cellular Processes | Cell communication |
| PATH:00010 | Glycolysis / Gluconeogenesis | 9 | 407 | 67 | 7932 | 0.01220979 | 0.105309436 | 2.617917782 | 6.355817844 | Metabolism | Carbohydrate metabolism |
| PATH:00630 | Glyoxylate and dicarboxylate metabolism | 6 | 407 | 36 | 7932 | 0.016073252 | 0.130476988 | 3.248157248 | 5.959194327 | Metabolism | Carbohydrate metabolism |
| PATH:04920 | Adipocytokine signaling pathway | 9 | 407 | 71 | 7932 | 0.016685171 | 0.131574487 | 2.470429456 | 5.905289761 | Organismal Systems | Endocrine system |
| PATH:00250 | Alanine, aspartate and glutamate metabolism | 6 | 407 | 39 | 7932 | 0.022032904 | 0.153532663 | 2.998298998 | 5.50419654 | Metabolism | Amino acid metabolism |
| PATH:04110 | Cell cycle | 13 | 407 | 132 | 7932 | 0.027289128 | 0.175158124 | 1.919365647 | 5.195529895 | Cellular Processes | Cell growth and death |

**Table S8: DEGs probably involved in muscle growth and development process**

| Gene ID | STH-normalized | QHMM-normalized | Log2FC | FDR | Style |
|---|---|---|---|---|---|
| AATF | 181.3141193 | 82.13125385 | 1.142488 | 6.89E-10 | up |
| ABAT | 22.09843496 | 100.0567623 | -2.178803 | 9.54E-04 | down |
| ABCB9 | 21.08561562 | 57.88460065 | -1.45692 | 6.37E-09 | down |
| ABCC5 | 299.4701362 | 684.2239398 | -1.192056 | 0 | down |
| ABCD1 | 163.4665462 | 379.4074562 | -1.214753 | 3.50E-04 | down |
| ABHD11 | 126.6597507 | 196.6984518 | -0.635027 | 0.013924 | down |
| ABHD6 | 16.62915353 | 41.04650966 | -1.303545 | 2.54E-04 | down |
| ACP5 | 388.3466223 | 233.8721929 | 0.7316246 | 0.0199459 | up |
| ACSM3 | 0.646964188 | 13.62967541 | -4.396922 | 0.0195692 | down |
| ACTN2 | 62892.35221 | 38891.04253 | 0.6934467 | 9.28E-04 | up |
| ADAMTS8 | 0.731710296 | 20.5048853 | -4.808551 | 2.85E-05 | down |
| ADCY1 | 3.065328725 | 15.98590951 | -2.382687 | 0.0050459 | down |
| ADHFE1 | 233.0735066 | 522.9754838 | -1.165958 | 9.22E-07 | down |
| ADIPOR2 | 3218.549745 | 997.4543171 | 1.6900881 | 1.09E-04 | up |
| AFMID | 30.6704503 | 7.416910928 | 2.047959 | 1.40E-05 | up |
| AGAP2 | 53.52194958 | 24.29085783 | 1.1397172 | 2.22E-04 | up |
| AGL | 1334.639865 | 4059.580292 | -1.60488 | 0.0113894 | down |
| AGPAT9 | 120.9915832 | 226.7338866 | -0.906093 | 6.76E-04 | down |
| AGXT2L1 | 419.726203 | 1471.602546 | -1.809868 | 3.94E-12 | down |
| AHCYL2 | 646.3219566 | 1451.630306 | -1.167349 | 1.69E-05 | down |
| AK4 | 144.6412354 | 529.2371395 | -1.871435 | 5.60E-04 | down |
| ALAD | 987.415589 | 440.0070738 | 1.1661307 | 0 | up |
| ALAS1 | 1180.381108 | 2440.355139 | -1.047838 | 1.24E-14 | down |
| ALDH18A1 | 603.3092514 | 342.2219516 | 0.8179654 | 5.40E-10 | up |
| ALDH2 | 7973.550842 | 15161.7522 | -0.927142 | 2.65E-05 | down |
| ALG3 | 104.7675859 | 60.60578791 | 0.7896649 | 0.009096 | up |
| ALKBH5 | 1301.584207 | 2247.439539 | -0.788014 | 8.04E-06 | down |
| AMOTL1 | 947.9953063 | 1647.020188 | -0.796906 | 0.0319473 | down |
| AMPD1 | 2881.739989 | 8247.763365 | -1.517063 | 1.80E-07 | down |
| AMPD3 | 1828.12717 | 115.0175341 | 3.9904407 | 1.21E-06 | up |
| AMY2B | 310.7383607 | 50.29568731 | 2.6271937 | 0.0089293 | up |
| ANGPTL4 | 810.5107478 | 187.0153882 | 2.1156743 | 0.0083303 | up |
| ANKRD23 | 3673.936454 | 8727.326014 | -1.248213 | 0.0059737 | down |
| APBB2 | 1310.031519 | 589.0851724 | 1.1530534 | 7.29E-08 | up |
| APIP | 399.2160341 | 178.3036018 | 1.1628338 | 0.029961 | up |
| AR | 45.00919237 | 86.08420073 | -0.935529 | 3.85E-04 | down |
| ARID5B | 4477.814012 | 449.4212476 | 3.3166544 | 0.0485792 | up |
| ASS1 | 207.4477519 | 897.0938098 | -2.112511 | 2.27E-04 | down |
| ASXL1 | 804.0872779 | 443.6637531 | 0.8578854 | 2.63E-04 | up |
| ATP13A1 | 388.7312941 | 220.7561814 | 0.8163194 | 3.49E-04 | up |
| ATP5A1 | 7191.29801 | 11921.13286 | -0.729197 | 0.0170226 | down |
| AZIN1 | 274.5965905 | 730.7821807 | -1.412128 | 7.94E-05 | down |
| B3GALNT1 | 795.6170577 | 247.9427724 | 1.682067 | 2.11E-05 | up |
| B3GALTL | 90.14854734 | 168.4049198 | -0.901558 | 1.65E-04 | down |
| BCL9L | 156.1731351 | 364.4586717 | -1.222609 | 0.005161 | down |
| BDH1 | 17.85688158 | 60.07323631 | -1.750242 | 0.0135222 | down |
| BLVRB | 449.2771351 | 718.4194143 | -0.677221 | 0.0378636 | down |
| BNIP3 | 5520.951614 | 2061.477173 | 1.4212385 | 7.94E-04 | up |
| BOP1 | 254.8108761 | 124.9422278 | 1.0281657 | 2.97E-05 | up |
| BTG1 | 3228.473692 | 670.5420525 | 2.2674526 | 9.08E-05 | up |
| BUB3 | 165.6427464 | 105.5623822 | 0.6499792 | 0.0012714 | up |
| C1QTNF2 | 9.20235817 | 36.91340005 | -2.004069 | 0.0016594 | down |
| C1QTNF3 | 41.52403898 | 142.106955 | -1.774958 | 0.0063664 | down |
| C2H9orf41 | 49.29730265 | 336.8750633 | -2.772633 | 7.10E-07 | down |
| C3H9orf114 | 116.6899164 | 76.28925955 | 0.613128 | 0.0281089 | up |

| | | | | | |
|---|---|---|---|---|---|
| CA12 | 123.5386991 | 8.762526325 | 3.8174724 | 0 | up |
| CA2 | 262.1757619 | 933.4641547 | -1.83206 | 1.56E-12 | down |
| CA3 | 15687.05914 | 54091.28904 | -1.785821 | 6.94E-06 | down |
| CA4 | 305.2311466 | 160.4150914 | 0.9280923 | 0.0049397 | up |
| CAMK2 | 1177.641464 | 2817.942056 | -1.258742 | 0.0012439 | down |
| CAMK2B | 979.0561731 | 622.7675129 | 0.6526979 | 2.26E-13 | up |
| CAMK2G | 486.8823402 | 1199.344128 | -1.300601 | 9.94E-12 | down |
| CARNS1 | 51.79898779 | 312.9982853 | -2.595159 | 0 | down |
| CASK | 73.65457419 | 123.8895829 | -0.750208 | 0.0041434 | down |
| CASQ1 | 17353.2746 | 45168.036 | -1.380094 | 5.41E-04 | down |
| CASQ2 | 248.0998682 | 620.41971 | -1.322324 | 0.019007 | down |
| CAST | 1951.309208 | 1068.649835 | 0.8686532 | 0.0077637 | up |
| CBFB | 125.1860522 | 58.97968388 | 1.0857838 | 4.56E-05 | up |
| CBLB | 151.1247617 | 84.71483447 | 0.8350535 | 0.0011867 | up |
| CBS | 273.2813509 | 111.9863728 | 1.2870638 | 0.0086567 | up |
| CBY1 | 296.9379738 | 497.8886011 | -0.745661 | 5.48E-04 | down |
| CCDC62 | 49.70610818 | 21.69918755 | 1.1957821 | 0.0084428 | up |
| CCND1 | 10.88901241 | 39.23665141 | -1.849329 | 0.0018275 | down |
| CCND3 | 227.7056069 | 125.6076995 | 0.8582449 | 8.35E-07 | up |
| CCNG2 | 124.1034688 | 74.15179952 | 0.7429898 | 0.0299953 | up |
| CDC26 | 44.18053366 | 77.66785768 | -0.813907 | 9.84E-04 | down |
| CDH15 | 263.6988255 | 122.5036723 | 1.1060661 | 0.0016623 | up |
| CDK8 | 99.05491423 | 53.44418995 | 0.8901954 | 0.0122289 | up |
| CDKN1A | 532.8482013 | 110.6358942 | 2.2679051 | 2.63E-05 | up |
| CDKN2C | 171.2298884 | 463.3919251 | -1.436298 | 1.92E-05 | down |
| CEBPB | 1512.302261 | 538.5327709 | 1.4896405 | 5.81E-06 | up |
| CEBPD | 2524.4366 | 291.3246856 | 3.1152616 | 0 | up |
| CGREF1 | 172.1998714 | 69.2266922 | 1.3146837 | 8.37E-10 | up |
| CHI3L1 | 402.5227716 | 149.7991028 | 1.4260414 | 0 | up |
| CHPT1 | 163.2434266 | 347.7041268 | -1.090835 | 1.05E-08 | down |
| CHRNE | 313.9368171 | 834.4670671 | -1.410381 | 0.0258351 | down |
| CISH | 746.8521088 | 330.8242686 | 1.1747575 | 9.07E-05 | up |
| CITED2 | 289.668839 | 155.8895215 | 0.8938805 | 0.0254268 | up |
| COL11A2 | 6.908069348 | 43.64821253 | -2.659568 | 0.0277273 | down |
| CREB3L1 | 86.70298015 | 189.2762538 | -1.12634 | 1.21E-10 | down |
| CRY2 | 214.9062504 | 460.1215124 | -1.098307 | 0.009818 | down |
| CRYL1 | 157.6592212 | 77.37599406 | 1.0268516 | 8.07E-10 | up |
| CSGALNACT1 | 169.9707708 | 76.10159593 | 1.1592881 | 0.0026819 | up |
| CSRP3 | 18425.39633 | 4896.283026 | 1.9119368 | 0.0460654 | up |
| CTTN | 609.3744498 | 394.7775271 | 0.6262891 | 3.29E-07 | up |
| CWF19L2 | 99.35844642 | 54.31138499 | 0.871388 | 0.0047258 | up |
| CXCR3 | 4.477245921 | 19.52207995 | -2.124423 | 0.0186567 | down |
| CYLD | 379.1613702 | 174.0174481 | 1.12358 | 0 | up |
| DAB2 | 182.173298 | 316.6880533 | -0.797751 | 0.0340774 | down |
| DAGLA | 18.86556696 | 40.94361672 | -1.117883 | 0.0218324 | down |
| DCTD | 115.5635843 | 46.25834655 | 1.3209013 | 0.0467838 | up |
| DDO | 1630.251523 | 371.9829266 | 2.1317863 | 0.0228098 | up |
| DDX27 | 491.4054445 | 217.4641727 | 1.1761361 | 1.40E-07 | up |
| DDX54 | 1150.593827 | 749.2953494 | 0.6187722 | 7.60E-04 | up |
| DDX56 | 416.4641407 | 180.0069638 | 1.2101396 | 0.0414319 | up |
| DECR1 | 115.8826402 | 192.9569547 | -0.735615 | 2.68E-07 | down |
| DGAT2 | 39.34193718 | 641.442781 | -4.027181 | 0.0149787 | down |
| DHDDS | 92.08587615 | 54.61049044 | 0.7538018 | 0.0047142 | up |
| DHDH | 381.7764968 | 941.3623591 | -1.302022 | 2.38E-05 | down |
| DHRS7B | 112.4449846 | 281.1290178 | -1.322013 | 7.56E-04 | down |
| DHTKD1 | 396.5721409 | 611.702189 | -0.625246 | 5.33E-08 | down |
| DHX34 | 72.87359854 | 42.29980638 | 0.7847452 | 0.0169741 | up |
| DKK2 | 47.34898322 | 440.4565033 | -3.217594 | 1.32E-05 | down |
| DLAT | 812.4394665 | 1468.41131 | -0.853924 | 1.97E-05 | down |

| | | | | | |
|---|---|---|---|---|---|
| DNMT3A | 378.97634 | 650.7281796 | -0.779947 | 0 | down |
| DTL | 33.76738241 | 8.248980123 | 2.0333427 | 2.41E-04 | up |
| DVL1 | 1425.181295 | 2286.054664 | -0.681714 | 0.0041237 | down |
| DYNC1LI1 | 374.7389491 | 230.6639186 | 0.7000936 | 0.0010106 | up |
| DYNLL1 | 283.2590825 | 468.9995077 | -0.727464 | 6.17E-04 | down |
| DYNLL2 | 960.5707306 | 1919.769166 | -0.998969 | 0.0459275 | down |
| DYRK1B | 651.4066945 | 1404.550559 | -1.108478 | 2.31E-10 | down |
| ECE2 | 143.7929761 | 45.21865453 | 1.6690032 | 0.0347232 | up |
| ECHDC3 | 949.3372455 | 246.5108556 | 1.9452695 | 4.33E-08 | up |
| EDEM1 | 308.2149794 | 134.3680003 | 1.1977474 | 2.89E-08 | up |
| EIF2C1 | 493.6139323 | 885.0824667 | -0.842429 | 3.42E-12 | down |
| EIF2C2 | 265.4286867 | 133.0511167 | 0.9963437 | 0.0014086 | up |
| EIF4EBP1 | 1794.296709 | 734.2069564 | 1.2891598 | 3.76E-06 | up |
| ELOVL6 | 8.689719058 | 370.9648541 | -5.415829 | 8.81E-04 | down |
| EPB41 | 805.8569676 | 1381.936698 | -0.778096 | 5.33E-15 | down |
| EXTL3 | 88.08196825 | 166.6563522 | -0.919958 | 8.61E-07 | down |
| FABP5 | 889.5614609 | 257.4553527 | 1.788772 | 0 | up |
| FAM107A | 173.3808572 | 63.37389992 | 1.4519839 | 3.99E-11 | up |
| FAM20B | 124.3273154 | 252.9230119 | -1.024555 | 0.0226189 | down |
| FHOD3 | 171.971848 | 502.6496308 | -1.547381 | 0 | down |
| FMO2 | 38.43651518 | 7.513751338 | 2.3548723 | 2.54E-05 | up |
| FOSL1 | 27.59045316 | 6.142548123 | 2.16726 | 0.0116079 | up |
| FOSL2 | 1233.941517 | 209.535894 | 2.5580047 | 3.89E-07 | up |
| FOXJ2 | 566.3529635 | 1094.048364 | -0.949903 | 0 | down |
| FOXO1 | 1696.6063 | 203.6181372 | 3.0587138 | 1.62E-05 | up |
| FOXO3 | 1304.648395 | 292.1399396 | 2.1589295 | 0.0169481 | up |
| FOXO6 | 9.287104278 | 65.40202589 | -2.816035 | 6.04E-04 | down |
| FOXP1 | 382.0938421 | 233.3081894 | 0.7116901 | 0.0245094 | up |
| FRG1 | 185.8667742 | 108.2866664 | 0.7794133 | 0.0200482 | up |
| FRZB | 84.55360789 | 636.2511152 | -2.911658 | 0 | down |
| FTH1 | 36433.11543 | 20327.73081 | 0.8418012 | 2.25E-14 | up |
| FTSJ3 | 287.3123412 | 129.7847076 | 1.1464996 | 0.0239949 | up |
| FZD2 | 69.04857691 | 159.6101762 | -1.208869 | 0.0024395 | down |
| FZD7 | 172.926849 | 567.105143 | -1.713454 | 0 | down |
| FZD9 | 17.0094205 | 62.77971617 | -1.883965 | 2.17E-11 | down |
| GABARAPL1 | 1598.550021 | 577.8455735 | 1.468008 | 0.0484231 | up |
| GADD45G | 1155.761914 | 49.02994405 | 4.5590373 | 2.23E-08 | up |
| GADL1 | 358.8395672 | 1945.97659 | -2.439083 | 0 | down |
| GAL-1 | 1542.340159 | 3771.58081 | -1.290048 | 7.17E-09 | down |
| GALNT12 | 42.19011915 | 104.3099686 | -1.3059 | 1.95E-07 | down |
| GALNTL2 | 65.11336396 | 8.378650186 | 2.9581639 | 9.43E-04 | up |
| GANC | 161.1101055 | 279.5570803 | -0.795096 | 9.63E-04 | down |
| GBGT1 | 92.9255825 | 49.6545435 | 0.9041501 | 4.68E-05 | up |
| GCAT | 260.287898 | 549.257811 | -1.077375 | 0 | down |
| GCNT1 | 27.09219736 | 64.34325782 | -1.247912 | 0.0105506 | down |
| GDF11 | 5.8620073 | 25.12173772 | -2.09947 | 3.47E-04 | down |
| GLO1 | 498.3501635 | 876.9826448 | -0.815388 | 0.0243074 | down |
| GLRX5 | 613.4051571 | 1634.164859 | -1.413641 | 4.55E-06 | down |
| GLT25D2 | 159.4510632 | 68.46755948 | 1.2196212 | 1.51E-06 | up |
| GPD1 | 6108.79247 | 18995.4779 | -1.636697 | 1.32E-09 | down |
| GPI | 8221.674045 | 23688.58373 | -1.526688 | 9.52E-06 | down |
| GPR116 | 1982.390659 | 1148.083004 | 0.7880143 | 1.96E-11 | up |
| GPT2 | 1993.4454 | 4285.902795 | -1.104335 | 0.0286905 | down |
| GPX1 | 761.868891 | 1529.089222 | -1.005058 | 1.22E-06 | down |
| GPX3 | 3812.124227 | 1709.056044 | 1.1573954 | 0 | up |
| HADH | 1967.372451 | 3165.430494 | -0.686132 | 3.81E-06 | down |
| HADHB | 4656.47436 | 2153.572991 | 1.1125058 | 0.0074222 | up |
| HCLS1 | 76.09340894 | 121.2520595 | -0.672166 | 0.0299891 | down |
| HDAC4 | 175.0001565 | 81.82322269 | 1.0967739 | 4.33E-06 | up |

| | | | | | |
|---|---|---|---|---|---|
| HEYL | 186.3627207 | 310.0585124 | -0.734427 | 7.27E-05 | down |
| HGS | 788.1457296 | 426.1031154 | 0.8872598 | 1.18E-14 | up |
| HLCS | 112.2425632 | 217.3646475 | -0.953497 | 0.0056306 | down |
| HLF | 31.70405346 | 76.33043792 | -1.267591 | 1.02E-04 | down |
| HMOX1 | 1141.803853 | 126.9970956 | 3.1684474 | 6.82E-09 | up |
| HNRNPU | 1481.220881 | 913.1363352 | 0.6978846 | 0.0041254 | up |
| HPDL | 12.27405889 | 43.69494886 | -1.831854 | 1.72E-09 | down |
| HR | 342.6766099 | 624.7869016 | -0.866516 | 2.22E-16 | down |
| HRC | 3870.385658 | 7034.373774 | -0.861945 | 6.12E-12 | down |
| HSD17B12 | 66.9054626 | 209.7965294 | -1.648795 | 0.0019327 | down |
| HSDL2 | 796.6074396 | 307.0053194 | 1.3756053 | 0.0155029 | up |
| HSF4 | 182.3378437 | 112.4849054 | 0.6968826 | 0.0130165 | up |
| HYAL2 | 218.549492 | 126.6512656 | 0.7870985 | 0.0017557 | up |
| IBA57 | 117.7212815 | 195.3305457 | -0.730542 | 2.66E-04 | down |
| IDH1 | 98.84113166 | 288.3936029 | -1.544856 | 9.51E-05 | down |
| IDH2 | 4927.240112 | 7686.788795 | -0.641601 | 0.0118348 | down |
| IDH3A | 1454.473484 | 2965.801513 | -1.027925 | 1.89E-15 | down |
| IGFBP5 | 195.9773056 | 1216.778217 | -2.634308 | 6.57E-08 | down |
| IGFR1 | 823.8780386 | 361.6607092 | 1.1877939 | 4.66E-15 | up |
| IKKBETA | 265.0470513 | 174.3848152 | 0.6039741 | 0.0087653 | up |
| IL4R | 369.1360838 | 221.7751018 | 0.7350554 | 0.041588 | up |
| IMPDH2 | 1949.012083 | 1275.794157 | 0.6113475 | 0.0033503 | up |
| INCA1 | 132.7081852 | 73.07954868 | 0.8607177 | 0.0038897 | up |
| ING2 | 59.03995468 | 138.1332482 | -1.226297 | 1.68E-05 | down |
| INSR | 764.8249693 | 444.1883564 | 0.783958 | 0.0390842 | up |
| IRS1 | 413.8593775 | 887.8892018 | -1.101239 | 0.0022419 | down |
| ISG20L2 | 362.487071 | 223.0604274 | 0.7004949 | 0.0080749 | up |
| ITGA7 | 1157.258948 | 4307.754627 | -1.896224 | 0 | down |
| ITGB1BP2 | 373.4335882 | 1183.376565 | -1.663986 | 1.42E-14 | down |
| IVD | 1198.27012 | 2141.552322 | -0.837704 | 1.58E-04 | down |
| JAM3 | 743.8513411 | 456.6192549 | 0.7040226 | 0.0050802 | up |
| JARID2 | 77.55883949 | 167.0263217 | -1.106712 | 2.91E-06 | down |
| KANK1 | 553.0456863 | 1253.544507 | -1.180543 | 1.22E-04 | down |
| KAT5 | 329.2406527 | 218.998488 | 0.5882216 | 0.0465332 | up |
| KCNIP2 | 68.77604939 | 173.2599992 | -1.33296 | 7.76E-05 | down |
| KCNJ12 | 343.5465939 | 1060.515947 | -1.626189 | 4.84E-13 | down |
| KCNS3 | 421.8590394 | 1395.093535 | -1.725529 | 0.0193937 | down |
| KCTD11 | 30.42674644 | 62.3531662 | -1.035123 | 2.90E-04 | down |
| KERA | 14.107212 | 51.96737369 | -1.881173 | 2.50E-06 | down |
| KHK | 56.42355937 | 101.2293862 | -0.843259 | 9.94E-04 | down |
| KIAA1161 | 214.7233015 | 661.5827602 | -1.623443 | 1.19E-06 | down |
| KIAA1456 | 5.415568511 | 31.10979336 | -2.522184 | 1.38E-09 | down |
| KLF11 | 1014.826315 | 361.1453378 | 1.4905814 | 0.0386192 | up |
| KLF15 | 124.2020566 | 45.6742425 | 1.4432364 | 3.81E-08 | up |
| KY | 233.7512189 | 1386.519932 | -2.568423 | 1.21E-04 | down |
| LDHB | 827.3723722 | 1608.181673 | -0.958822 | 1.25E-07 | down |
| LDHD | 150.6412313 | 280.1180929 | -0.894918 | 3.31E-04 | down |
| LEPREL1 | 58.51571198 | 124.3665078 | -1.087702 | 9.45E-12 | down |
| LGALS12 | 1.39529584 | 15.84670142 | -3.50554 | 0.0129061 | down |
| LIN52 | 77.275749 | 196.3380557 | -1.345252 | 9.50E-06 | down |
| LIN54 | 149.6212275 | 75.89630462 | 0.9792133 | 0.0015318 | up |
| LIPG | 188.1968859 | 48.02262244 | 1.9704567 | 0.013381 | up |
| LMOD2 | 12507.28684 | 5623.44244 | 1.1532434 | 1.18E-08 | up |
| LOC100037702 | 59.12082342 | 104.5929419 | -0.823047 | 0.0037649 | down |
| LOC100885761 | 27.75414358 | 50.17946468 | -0.854394 | 0.0386417 | down |
| LOC101102411 | 21.87106735 | 59.15762107 | -1.43554 | 2.22E-04 | down |
| LOC101102833 | 88.51489327 | 333.2993866 | -1.912827 | 0.0023676 | down |
| LOC101102999 | 12727.70563 | 2629.161886 | 2.2752975 | 2.66E-05 | up |
| LOC101104299 | 288.4837905 | 502.8181949 | -0.801547 | 0 | down |

| | | | | | |
|---|---|---|---|---|---|
| LOC101104304 | 97.23531765 | 177.5600596 | -0.868755 | 5.35E-07 | down |
| LOC101104557 | 859.0296977 | 413.5879643 | 1.0545138 | 5.11E-06 | up |
| LOC101105484 | 55594.73484 | 30118.56823 | 0.8842951 | 0 | up |
| LOC101105651 | 312.9768411 | 678.2624846 | -1.115788 | 0.0217991 | down |
| LOC101105659 | 73.77810822 | 170.0785256 | -1.204936 | 0.0129357 | down |
| LOC101106123 | 59.58520928 | 202.5474524 | -1.765234 | 2.96E-05 | down |
| LOC101106131 | 3147.297014 | 5807.240663 | -0.883739 | 1.52E-05 | down |
| LOC101106865 | 111.8739996 | 383.4421946 | -1.777134 | 0.0208315 | down |
| LOC101107037 | 12.13528625 | 35.4765022 | -1.547656 | 0.0394431 | down |
| LOC101107119 | 34.796538 | 100.8593343 | -1.535329 | 0.0060906 | down |
| LOC101107153 | 937.1214042 | 1656.476729 | -0.82181 | 8.43E-05 | down |
| LOC101107541 | 1066.297181 | 3365.518702 | -1.658219 | 8.53E-13 | down |
| LOC101107831 | 108.6542605 | 288.0198182 | -1.406423 | 1.28E-04 | down |
| LOC101107954 | 1360.910075 | 478.8783953 | 1.5068405 | 1.39E-08 | up |
| LOC101108705 | 907.6684326 | 1735.212775 | -0.934875 | 0.0231156 | down |
| LOC101108849 | 268.3145162 | 1336.155241 | -2.316091 | 0.0050406 | down |
| LOC101110740 | 106.5025077 | 482.0123672 | -2.178183 | 0 | down |
| LOC101112151 | 1724.713854 | 3674.738645 | -1.091285 | 1.11E-15 | down |
| LOC101112287 | 433.9551814 | 212.2366628 | 1.0318722 | 0.0058515 | up |
| LOC101112367 | 22.16879775 | 52.1251986 | -1.23345 | 0.0014646 | down |
| LOC101112411 | 2349.119198 | 634.6033799 | 1.8881928 | 0 | up |
| LOC101112867 | 1708.156773 | 2733.784914 | -0.678459 | 0 | down |
| LOC101113032 | 59.36153373 | 26.57388463 | 1.1595192 | 0.0044008 | up |
| LOC101114955 | 771.3872545 | 1537.491541 | -0.995051 | 0.0047173 | down |
| LOC101115438 | 256.3030918 | 140.5249496 | 0.8670246 | 0.0020594 | up |
| LOC101116336 | 174.6776366 | 1770.450862 | -3.34135 | 0 | down |
| LOC101117028 | 203.1255151 | 492.3352676 | -1.27727 | 2.25E-04 | down |
| LOC101117181 | 756.351114 | 1264.263201 | -0.741169 | 7.09E-07 | down |
| LOC101118433 | 1931.381984 | 4075.965162 | -1.077508 | 0.002245 | down |
| LOC101119498 | 87.44275879 | 263.6174479 | -1.592035 | 0.026075 | down |
| LOC101119560 | 129.553563 | 329.2842777 | -1.345785 | 0.009106 | down |
| LOC101119893 | 143.8046082 | 74.34597491 | 0.9517834 | 0.0385721 | up |
| LOC101120404 | 445.0648045 | 807.7077091 | -0.859818 | 0.0481813 | down |
| LOC101120590 | 254.5113638 | 525.3541617 | -1.04556 | 6.58E-08 | down |
| LOC101120670 | 1544.277872 | 373.8100475 | 2.0465551 | 0.0172066 | up |
| LOC101120934 | 9873.350111 | 1810.974005 | 2.4467738 | 0.0340509 | up |
| LOC101121539 | 571.7000366 | 99.05771878 | 2.5289171 | 0 | up |
| LOC101121811 | 157.1213222 | 247.3502019 | -0.654676 | 0.049508 | down |
| LOC101123148 | 89.92427308 | 145.045574 | -0.689724 | 0.0153349 | down |
| LOC101123553 | 239.1881557 | 397.8277763 | -0.733998 | 0.0087314 | down |
| LOC443090 | 1479.471576 | 515.5194552 | 1.5209832 | 0.0365898 | up |
| LOC443301 | 2228.663276 | 1254.810875 | 0.8287087 | 0.0367863 | up |
| LOC780509 | 27537.55444 | 77386.23824 | -1.490677 | 0.0023434 | down |
| LPIN3 | 113.4797281 | 63.08536872 | 0.8470573 | 0.0247466 | up |
| LRP4 | 1142.316748 | 410.207617 | 1.4775366 | 2.33E-09 | up |
| LRRC17 | 14.00199568 | 46.2634806 | -1.724241 | 0.0078096 | down |
| MAFF | 239.2610273 | 73.10877508 | 1.7104689 | 0.0018853 | up |
| MANEAL | 53.9889156 | 12.30083988 | 2.1339064 | 5.33E-09 | up |
| MCM7 | 747.8541656 | 480.1407237 | 0.6392997 | 1.18E-14 | up |
| ME2 | 84.20242116 | 168.5529712 | -1.001268 | 0.0307632 | down |
| MED6 | 115.9654619 | 73.34455275 | 0.6609335 | 0.0088671 | up |
| MEOX1 | 25.76562498 | 77.72098802 | -1.592857 | 0.004529 | down |
| METTL11B | 83.59778013 | 414.695235 | -2.310515 | 0 | down |
| METTL14 | 77.20822296 | 46.53492577 | 0.7304406 | 0.035555 | up |
| METTL3 | 379.3370063 | 170.0169641 | 1.1578014 | 0.0026464 | up |
| METTL7B | 42.97468707 | 643.6362916 | -3.904687 | 0 | down |
| MGST3 | 541.3641374 | 957.6110707 | -0.822841 | 4.54E-06 | down |
| MKL1 | 158.2914457 | 352.2580047 | -1.154049 | 0.0046415 | down |
| MLH3 | 124.9033324 | 69.45657043 | 0.8466289 | 2.95E-05 | up |

| | | | | | |
|---|---|---|---|---|---|
| MMAB | 154.505996 | 269.4487855 | -0.802348 | 0.0036635 | down |
| MOCS2 | 377.2894007 | 783.4246818 | -1.054123 | 0.0037639 | down |
| MPP5 | 83.22505406 | 138.541829 | -0.735232 | 0.0053398 | down |
| MRC2 | 228.0121041 | 764.6913378 | -1.745767 | 5.30E-09 | down |
| MT1A | 51.14509567 | 2.735588508 | 4.2246728 | 0.0033719 | up |
| MT2A | 137.9798492 | 27.50145185 | 2.3268779 | 0.0036939 | up |
| MX2 | 31.82292609 | 96.10986796 | -1.594618 | 0.0078806 | down |
| MYC | 1252.153492 | 233.7906604 | 2.4211222 | 1.48E-12 | up |
| MYF6 | 4474.066182 | 1688.584259 | 1.4057724 | 0 | up |
| MYH6 | 4502.737351 | 12787.65091 | -1.505877 | 1.17E-10 | down |
| MYH7B | 380.7357804 | 1533.55853 | -2.010021 | 9.47E-11 | down |
| MYL2 | 23309.2945 | 63933.45192 | -1.455666 | 7.13E-12 | down |
| MYL6B | 1654.633855 | 12018.29032 | -2.860648 | 0.0376922 | down |
| MYLK3 | 94.11932658 | 265.7931307 | -1.497741 | 6.03E-06 | down |
| MYOC | 212.1440262 | 576.9584545 | -1.443423 | 0 | down |
| MYOD1 | 37.50764368 | 232.4596229 | -2.631724 | 3.46E-06 | down |
| MYOG | 148.6274671 | 501.8397817 | -1.755526 | 2.41E-04 | down |
| N4BP2 | 125.6999173 | 29.83757367 | 2.0747816 | 0.0041176 | up |
| NAA40 | 43.73851393 | 93.83622611 | -1.101241 | 0.0267806 | down |
| NAT9 | 79.22165934 | 43.26185179 | 0.8727995 | 0.0358424 | up |
| NCEH1 | 239.9666937 | 70.45838117 | 1.7679909 | 9.50E-08 | up |
| NCOA5 | 310.5349843 | 182.0044504 | 0.7707821 | 0.0132316 | up |
| NDRG1 | 450.6306637 | 237.7062677 | 0.9227655 | 0.0055364 | up |
| NDUFA4 | 1781.124056 | 2812.772422 | -0.659205 | 2.49E-05 | down |
| NDUFA6 | 270.6188406 | 445.0870151 | -0.717825 | 2.20E-05 | down |
| NDUFS1 | 1905.335649 | 3552.03239 | -0.8986 | 0.0022512 | down |
| NEO1 | 178.1191839 | 390.0595363 | -1.130851 | 2.90E-06 | down |
| NEURL | 377.1765864 | 595.3395745 | -0.658473 | 0.0195525 | down |
| NFATC1 | 138.4233515 | 226.9638472 | -0.713375 | 0.041235 | down |
| NFIX | 2638.054759 | 5652.90902 | -1.099519 | 1.86E-08 | down |
| NMNAT2 | 24.64061862 | 74.52525449 | -1.596691 | 0.0044238 | down |
| NMNAT3 | 88.45141566 | 183.4607304 | -1.052514 | 0.0046446 | down |
| NRF1 | 183.1791608 | 117.6321546 | 0.6389729 | 4.28E-04 | up |
| NSUN2 | 679.7356434 | 417.9196415 | 0.7017482 | 3.58E-10 | up |
| NSUN3 | 41.68325332 | 91.57032066 | -1.135412 | 1.85E-04 | down |
| NT5C1A | 348.2609579 | 623.9887807 | -0.841351 | 8.75E-07 | down |
| NUDT1 | 71.16417903 | 27.56830047 | 1.3681409 | 0.0478331 | up |
| NUDT19 | 78.30932365 | 43.72881474 | 0.8405998 | 0.0029691 | up |
| NUFIP1 | 167.0873361 | 105.4569576 | 0.6639481 | 2.43E-04 | up |
| OAT | 70.8432272 | 167.1316639 | -1.238283 | 8.43E-11 | down |
| OGFR | 133.3547787 | 76.5296767 | 0.8011783 | 0.041873 | up |
| OLA1 | 260.3596863 | 168.8805377 | 0.624503 | 1.47E-07 | up |
| OMYHCS | 88659.65715 | 225229.999 | -1.345049 | 3.66E-14 | down |
| OSBPL11 | 344.4226363 | 160.2165827 | 1.1041565 | 6.03E-04 | up |
| OXNAD1 | 168.5967434 | 91.33036275 | 0.8844102 | 3.16E-04 | up |
| P2RY1 | 99.97576016 | 416.4010463 | -2.058323 | 7.22E-07 | down |
| PA2G4 | 1422.570445 | 728.7797367 | 0.9649453 | 0.0044804 | up |
| PAK1 | 286.9866995 | 482.127318 | -0.74843 | 0.0018002 | down |
| PAPSS2 | 45.73790911 | 10.539708 | 2.1175555 | 0.0050523 | up |
| PC | 140.2922277 | 463.209737 | -1.72323 | 0.0093536 | down |
| PCCA | 205.6498087 | 317.5831442 | -0.626945 | 0.0064756 | down |
| PCCB | 540.8106434 | 929.1569466 | -0.780799 | 0.0075993 | down |
| PCDH12 | 76.06435721 | 317.2170135 | -2.060178 | 0 | down |
| PCK1 | 7.494920107 | 97.48680577 | -3.701222 | 0.009503 | down |
| PCMT1 | 458.5493144 | 835.6270796 | -0.865782 | 0.0299133 | down |
| PCYT1A | 123.5074378 | 211.1827933 | -0.773894 | 6.89E-04 | down |
| PDE4B | 74.50132252 | 470.9403784 | -2.660206 | 0.0215371 | down |
| PDE8B | 35.5548054 | 95.97567617 | -1.432624 | 9.99E-16 | down |
| PDHB | 1409.303438 | 2302.230298 | -0.70805 | 0.0088872 | down |

| | | | | | |
|---|---|---|---|---|---|
| PDK4 | 37397.81011 | 1055.774358 | 5.1465804 | 0 | up |
| PDP1 | 263.4641878 | 725.01642 | -1.460407 | 9.88E-07 | down |
| PDXP | 60.5698892 | 185.6329748 | -1.61578 | 6.97E-08 | down |
| PES1 | 853.1920522 | 322.3392165 | 1.4042908 | 0.0016697 | up |
| PFKFB3 | 8379.261621 | 949.7301171 | 3.1412336 | 0.0017449 | up |
| PGK1 | 6945.387884 | 20207.27227 | -1.540747 | 1.25E-07 | down |
| PGM2L1 | 84.59072796 | 219.7124152 | -1.377045 | 0.0057313 | down |
| PHKG1 | 1553.971458 | 2342.423967 | -0.592042 | 0 | down |
| PIAS3 | 195.8198874 | 305.2021731 | -0.640238 | 1.00E-05 | down |
| PID1 | 641.3657562 | 265.4927888 | 1.2724747 | 0.0022621 | up |
| PIGZ | 32.75172632 | 102.1158457 | -1.640564 | 0.0072824 | down |
| PIK3R2 | 91.5192105 | 147.4821394 | -0.688394 | 0.0080294 | down |
| PLA2G15 | 105.4887903 | 51.96641989 | 1.0214381 | 4.04E-06 | up |
| PLCL1 | 87.90888377 | 209.3696733 | -1.251972 | 0.0251537 | down |
| PLIN2 | 724.6671744 | 237.154381 | 1.611492 | 0.0143632 | up |
| PLTP | 136.5850096 | 325.7274415 | -1.253866 | 1.06E-04 | down |
| PNPLA2 | 2151.002912 | 1018.810065 | 1.0781244 | 4.10E-05 | up |
| POLR2D | 215.1294698 | 98.56416711 | 1.12607 | 0 | up |
| PPARGC1B | 111.0494892 | 311.8478816 | -1.48964 | 2.96E-04 | down |
| PPM1J | 141.6196554 | 360.8402623 | -1.349339 | 0.0074631 | down |
| PPM1K | 118.3936054 | 879.010029 | -2.892288 | 4.55E-11 | down |
| PPP1R3B | 181.5400756 | 657.3736407 | -1.856426 | 0.0270473 | down |
| PPP1R3C | 434.6504844 | 7755.408986 | -4.157275 | 0 | down |
| PPP2R2D | 67.58639651 | 1.797140875 | 5.2329575 | 0.0139147 | up |
| PPP2R3A | 364.1065841 | 740.6440765 | -1.02442 | 0.0135694 | down |
| PPP3CB | 1122.042201 | 1977.985914 | -0.817905 | 0.0384972 | down |
| PPP3R1 | 1009.912295 | 1793.588443 | -0.828619 | 0.0359071 | down |
| PPT2 | 957.0734034 | 573.9544236 | 0.7376934 | 0.0152682 | up |
| PPTC7 | 429.4324477 | 761.0685362 | -0.825595 | 0.0384603 | down |
| PRKACA | 1622.440084 | 2453.779664 | -0.596841 | 0.0221068 | down |
| PRKAG3 | 519.9357598 | 1091.878498 | -1.070407 | 0.0235431 | down |
| PRKCQ | 1528.81324 | 756.1949813 | 1.015582 | 0.028832 | up |
| PRUNE | 160.9679687 | 297.3121289 | -0.885205 | 5.77E-05 | down |
| PSMA7 | 1560.053121 | 909.5383913 | 0.7783887 | 3.82E-07 | up |
| PSMC1 | 5260.111006 | 3139.922455 | 0.7443643 | 0.0198785 | up |
| PTPLA | 241.1555198 | 459.766792 | -0.930938 | 1.43E-06 | down |
| PTPN1 | 200.8366147 | 130.476344 | 0.622234 | 0.0019005 | up |
| PTPN14 | 413.8985788 | 642.9691596 | -0.635472 | 0.0036889 | down |
| PTPN21 | 723.6985456 | 1130.673209 | -0.643721 | 0.0481714 | down |
| PTPN23 | 276.0397404 | 154.3996463 | 0.8382065 | 0.0016073 | up |
| PTPN3 | 454.2008626 | 155.5045384 | 1.5463738 | 0.0171404 | up |
| PTPRT | 3.065328725 | 23.11835173 | -2.914925 | 1.41E-04 | down |
| QPCT | 21.22855073 | 66.06535444 | -1.637888 | 1.02E-12 | down |
| QSOX2 | 388.7050649 | 192.348146 | 1.014956 | 0.0150683 | up |
| RAB21 | 117.9725548 | 66.18563956 | 0.8338611 | 0.0017914 | up |
| RAPSN | 269.0737244 | 139.8930024 | 0.9436777 | 1.45E-10 | up |
| RASD1 | 5337.657515 | 544.6662402 | 3.2927624 | 0 | up |
| RBKS | 50.96010824 | 18.5700318 | 1.4563921 | 0.0126481 | up |
| REV3L | 843.0103598 | 290.0467078 | 1.5392651 | 0.029951 | up |
| RGCC | 935.938779 | 306.8053741 | 1.6090904 | 4.67E-08 | up |
| RGMA | 101.3522251 | 252.9777908 | -1.319633 | 4.60E-14 | down |
| RHOBTB3 | 940.6464431 | 1771.08699 | -0.912911 | 0.0258507 | down |
| RNF146 | 607.912583 | 250.6624642 | 1.2781179 | 2.44E-15 | up |
| RRAS2 | 147.4988257 | 63.47636894 | 1.216412 | 2.77E-06 | up |
| RTN4 | 1304.10679 | 2785.308239 | -1.094775 | 0.0055559 | down |
| RTN4IP1 | 99.05481444 | 204.9477381 | -1.048957 | 0.0138536 | down |
| RUVBL1 | 1308.020392 | 588.2095698 | 1.1529829 | 0.0234151 | up |
| RXRG | 273.7361288 | 1104.109533 | -2.012026 | 2.89E-06 | down |
| SCARB1 | 165.6742785 | 351.3544356 | -1.084577 | 1.87E-07 | down |

| | | | | | |
|---|---|---|---|---|---|
| SCD | 83.91646541 | 2310.114784 | -4.782867 | 0.0010575 | down |
| SCN4A | 2682.021695 | 4051.300213 | -0.595064 | 0.0013001 | down |
| SDHC | 1779.835217 | 3580.428756 | -1.008389 | 7.90E-05 | down |
| SERPINF1 | 553.7479315 | 998.3276588 | -0.850284 | 0.0160024 | down |
| SESN1 | 4907.210295 | 623.2063446 | 2.9771213 | 1.19E-12 | up |
| SETD3 | 719.0181229 | 1297.087802 | -0.851176 | 1.02E-04 | down |
| SETD8 | 355.3638221 | 777.9235013 | -1.130331 | 2.45E-05 | down |
| SFRP2 | 8.685556591 | 51.06010716 | -2.555506 | 3.58E-12 | down |
| SFRP5 | 54.48017218 | 128.5185404 | -1.238173 | 1.82E-07 | down |
| SGCG | 431.4356062 | 1052.93683 | -1.287202 | 0.0014202 | down |
| SHMT2 | 787.4430426 | 516.171291 | 0.6093257 | 1.45E-04 | up |
| SLC25A42 | 237.1965437 | 110.3878584 | 1.1035015 | 0.0066219 | up |
| SLC27A1 | 197.0205452 | 396.6039886 | -1.009353 | 0.0142201 | down |
| SLC27A6 | 15.61857223 | 1.658238945 | 3.2355388 | 0.0038668 | up |
| SLC2A4 | 975.3060901 | 2020.969338 | -1.05112 | 0.0011416 | down |
| SLC33A1 | 77.18356178 | 46.85494424 | 0.7200923 | 0.0497333 | up |
| SLC37A4 | 206.2106155 | 601.112271 | -1.543516 | 6.98E-07 | down |
| SLMAP | 696.1450272 | 449.3423881 | 0.6315727 | 0.0316724 | up |
| SMAD3 | 159.9461116 | 312.761389 | -0.967477 | 0.0053151 | down |
| SMPDL3A | 96.04858704 | 25.04834202 | 1.9390493 | 8.37E-06 | up |
| SMS | 42.23358272 | 19.16149544 | 1.1401805 | 0.0028173 | up |
| SOCS2 | 191.2094853 | 66.11010074 | 1.5322115 | 0 | up |
| SOX6 | 258.4552866 | 448.8171255 | -0.796213 | 0 | down |
| SPP1 | 242.2902196 | 2.935474933 | 6.3669984 | 3.79E-06 | up |
| SREBF1 | 210.2108314 | 730.9851994 | -1.798005 | 6.85E-13 | down |
| SREBF2 | 520.8913309 | 877.6665685 | -0.752691 | 2.94E-05 | down |
| ST3GAL1 | 328.8051902 | 163.2516182 | 1.0101358 | 1.50E-07 | up |
| ST3GAL6 | 329.3967309 | 173.3094203 | 0.9264762 | 0.0021089 | up |
| ST6GALNAC4 | 107.0321958 | 191.6966517 | -0.84078 | 0.0210698 | down |
| STAC3 | 2569.446591 | 5060.113731 | -0.977712 | 0.0046488 | down |
| STBD1 | 469.3845426 | 1862.748251 | -1.98859 | 0 | down |
| TBC1D7 | 50.86315983 | 97.91275521 | -0.944876 | 2.79E-04 | down |
| TEF | 253.365987 | 422.3297778 | -0.737147 | 0.015005 | down |
| TFAP4 | 77.47353744 | 18.34788998 | 2.0780895 | 0.0053925 | up |
| TFRC | 471.3845222 | 2297.305391 | -2.284966 | 0 | down |
| TGFB2 | 55.01778609 | 126.0595664 | -1.196136 | 4.73E-07 | down |
| TH | 3.89231959 | 14.724111 | -1.919478 | 0.0361993 | down |
| THRSP | 20.26496824 | 354.2948679 | -4.127891 | 0.0037285 | down |
| THSD4 | 17.31131336 | 89.39424929 | -2.368467 | 0.0019832 | down |
| TMEM120A | 541.9802395 | 279.5817496 | 0.9549701 | 1.58E-07 | up |
| TNNC1 | 20984.0368 | 46777.41999 | -1.15652 | 0.0011457 | down |
| TNNT1 | 17000.23345 | 44366.56216 | -1.383918 | 4.84E-14 | down |
| TP53INP1 | 77.99364618 | 40.59981207 | 0.9418835 | 3.82E-04 | up |
| TPI1 | 8725.494231 | 19951.72263 | -1.193205 | 7.99E-07 | down |
| TRMT1 | 426.4312666 | 192.8552158 | 1.1447951 | 0.008658 | up |
| TUB | 20.71146405 | 58.16589001 | -1.489744 | 1.04E-07 | down |
| UCK2 | 356.5408736 | 59.18910833 | 2.5906638 | 1.70E-04 | up |
| UGP2 | 3640.223483 | 1805.096661 | 1.0119509 | 0 | up |
| USP2 | 793.4655614 | 1294.397348 | -0.706041 | 2.26E-04 | down |
| WBSCR17 | 13.49346201 | 66.21141578 | -2.294819 | 0.0192255 | down |
| WNK2 | 1479.645644 | 2703.294233 | -0.869467 | 3.55E-06 | down |
| XIRP1 | 37631.66032 | 7518.661738 | 2.3233991 | 1.11E-06 | up |
| ZBTB16 | 1768.331257 | 489.8326424 | 1.8520277 | 1.29E-14 | up |
| ZIC1 | 114.717677 | 213.9152849 | -0.898952 | 6.25E-06 | down |

## Table S9: The DEGs with highest k-core in the QHMM group and the STH group

**The DEGs with highest k-core in the QHMM group**

| Gene ID | K-core | STH normalized | QHMM normalized | Log2FC | FDR | Style | A1 | A2 | A3 | B1 | B2 | B3 | Symbol | Description | E-Value |
|---|---|---|---|---|---|---|---|---|---|---|---|---|---|---|---|
| CA2 | 8 | 262.1758 | 933.4642 | -1.83206 | 1.56E-12 | down | 1176 | 901 | 700 | 239 | 226 | 321 | CA2 | Carbonic anhydrase 2 | 8.00E-119 |
| CAMK2B | 8 | 979.0562 | 622.7675 | 0.652698 | 2.26E-13 | up | 595 | 600 | 631 | 1176 | 975 | 858 | CAMK2B | Uncharacterized protein | 0 |
| CCND3 | 8 | 227.7056 | 125.6077 | 0.858245 | 8.35E-07 | up | 125 | 121 | 123 | 286 | 183 | 231 | CCND3 | Ccnd3 | 3.00E-167 |
| CD4 | 8 | 10.81893 | 28.0584 | -1.37487 | 0.011593 | down | 39 | 27 | 18 | 7 | 13 | 12 | CD4 | T-cell surface glycoprotein CD4 | 1.00E-145 |
| CDKN1A | 8 | 532.8482 | 110.6359 | 2.267905 | 2.63E-05 | up | 181 | 107 | 47 | 438 | 501 | 650 | CDKN1A | CDKN1A | 6.00E-71 |
| CDKN2C | 8 | 171.2299 | 463.3919 | -1.4363 | 1.92E-05 | down | 387 | 445 | 519 | 196 | 122 | 203 | CDKN2C | Uncharacterized protein | 4.00E-80 |
| LOC101106131 | 8 | 3147.297 | 5807.241 | -0.88374 | 1.52E-05 | down | 5558 | 5577 | 5896 | 3173 | 2907 | 3438 | LOC101106131 | Cytochrome c oxidase subunit 6A, mitochondrial | 5.00E-34 |
| LOC101120934 | 8 | 9873.3501 | 1810.9740 | 2.44677 | 0.034050 | up | 2248 | 1754 | 1380 | 6648 | 14017 | 8732 | LOC101120934 | glutamine synthetase-like | 6E-159 |
| PC | 8 | 140.2922 | 463.2097 | -1.72323 | 0.009353 | down | 677 | 449 | 265 | 214 | 80 | 144 | PC | Pyruvate carboxylase | 0 |

**The DEGs with highest k-core in the STH group**

| Gene ID | K-core | STH normalized | QHMM normalized | Log2FC | FDR | Style | A1 | A2 | A3 | B1 | B2 | B3 | Symbol | Description | E-Value |
|---|---|---|---|---|---|---|---|---|---|---|---|---|---|---|---|
| CD4 | 9 | 10.81893 | 28.0584 | -1.37487 | 0.011593 | down | 39 | 27 | 18 | 7 | 13 | 12 | CD4 | T-cell surface glycoprotein CD4 | 1.00E-145 |
| SLC2A4 | 9 | 975.3061 | 2020.969 | -1.05112 | 0.001142 | down | 1967 | 2051 | 1899 | 1556 | 737 | 779 | SLC2A4 | Uncharacterized protein | 0 |
| ME2 | 9 | 84.20242 | 168.553 | -1.00127 | 0.030763 | down | 218 | 104 | 188 | 136 | 70 | 60 | ME2 | Malic enzyme | 0 |
| MYL2 | 9 | 23309.29 | 63933.45 | -1.45567 | 7.13E-12 | down | 76275 | 46219 | 69149 | 21705 | 31231 | 17742 | MYL2 | Myosin light chain 2 | 4.00E-89 |
| AR | 9 | 45.00919 | 86.0842 | -0.93553 | 3.85E-04 | down | 105 | 65 | 88 | 62 | 26 | 51 | AR | Androgen receptor | 0 |
| LOC101121811 | 9 | 157.1213 | 247.3502 | -0.65468 | 0.049508 | down | 342 | 187 | 218 | 158 | 144 | 173 | LOC101121811 | Uncharacterized protein | 2.00E-24 |
| MMAB | 9 | 154.506 | 269.4488 | -0.80235 | 0.003664 | down | 268 | 241 | 285 | 147 | 123 | 194 | MMAB | Uncharacterized protein | 1.00E-113 |
| DVL1 | 9 | 1425.181 | 2286.055 | -0.68171 | 0.004124 | down | 2359 | 2230 | 2135 | 1541 | 1153 | 1630 | DVL1 | Uncharacterized protein | 0 |
| PHKG1 | 9 | 1553.971 | 2342.424 | -0.59204 | 0 | down | 2584 | 2137 | 2212 | 1688 | 1619 | 1432 | PHKG1 | Uncharacterized protein | 0 |
| ABAT | 9 | 22.09843 | 100.0568 | -2.1788 | 9.54E-04 | down | 90 | 63 | 144 | 25 | 13 | 29 | ABAT | Uncharacterized protein | 0 |
| CDKN1A | 9 | 532.8482 | 110.6358 | 2.2679 | 2.63E-05 | up | 181 | 107 | 47 | 438 | 501 | 650 | CDKN1A | CDKN1A | 6.00E-71 |

## Table S 10: The DEGs identified from the pathways

| PathwayID | PathwayTerm | QueryID | Symbol | Description | P-Value | FDR | Enrichment |
|---|---|---|---|---|---|---|---|
| PATH:01100 | Metabolic pathways | AGL | AGL | Uncharacterized protein | 0.0006911 | 0.0363803 | 1.4851029 |
| PATH:01100 | Metabolic pathways | AMY2B | AMY2B | Alpha-amylase | 0.0006911 | 0.0363803 | 1.4851029 |
| PATH:01100 | Metabolic pathways | GANC | GANC | Uncharacterized protein | 0.0006911 | 0.0363803 | 1.4851029 |
| PATH:01100 | Metabolic pathways | EXTL3 | EXTL3 | Uncharacterized protein | 0.0006911 | 0.0363803 | 1.4851029 |
| PATH:01100 | Metabolic pathways | NDUFA4 | NDUFA4 | NADH dehydrogenase (ubiquinone) 1 alpha subcomplex, 4, 9kDa | 0.0006911 | 0.0363803 | 1.4851029 |
| PATH:01100 | Metabolic pathways | AFMID | AFMID | Kynurenine formamidase | 0.0006911 | 0.0363803 | 1.4851029 |
| PATH:01100 | Metabolic pathways | UCK2 | UCK2 | Uncharacterized protein | 0.0006911 | 0.0363803 | 1.4851029 |
| PATH:01100 | Metabolic pathways | GALNTL2 | GALNTL2 | Polypeptide N-acetylgalactosaminyltransferase | 0.0006911 | 0.0363803 | 1.4851029 |
| PATH:01100 | Metabolic pathways | LOC101107153 | LOC101107153 | Uncharacterized protein | 0.0006911 | 0.0363803 | 1.4851029 |
| PATH:01100 | Metabolic pathways | MOCS2 | MOCS2 | Molybdopterin synthase sulfur carrier subunit | 0.0006911 | 0.0363803 | 1.4851029 |
| PATH:01100 | Metabolic pathways | IDH2 | IDH2 | Uncharacterized protein | 0.0006911 | 0.0363803 | 1.4851029 |
| PATH:01100 | Metabolic pathways | AMPD1 | AMPD1 | Uncharacterized protein | 0.0006911 | 0.0363803 | 1.4851029 |
| PATH:01100 | Metabolic pathways | HYAL2 | HYAL2 | Hyaluronidase-2 | 0.0006911 | 0.0363803 | 1.4851029 |
| PATH:01100 | Metabolic pathways | PC | PC | Pyruvate carboxylase | 0.0006911 | 0.0363803 | 1.4851029 |
| PATH:01100 | Metabolic pathways | PPT2 | PPT2 | Uncharacterized protein | 0.0006911 | 0.0363803 | 1.4851029 |
| PATH:01100 | Metabolic pathways | LOC101104299 | LOC101104299 | NADH dehydrogenase [ubiquinone] 1 alpha subcomplex subunit 4-like | 0.0006911 | 0.0363803 | 1.4851029 |
| PATH:01100 | Metabolic pathways | NMNAT2 | NMNAT2 | Uncharacterized protein | 0.0006911 | 0.0363803 | 1.4851029 |
| PATH:01100 | Metabolic pathways | PAPSS2 | PAPSS2 | Adenylyl-sulfate kinase | 0.0006911 | 0.0363803 | 1.4851029 |
| PATH:01100 | Metabolic pathways | ALDH2 | ALDH2 | Uncharacterized protein | 0.0006911 | 0.0363803 | 1.4851029 |
| PATH:01100 | Metabolic pathways | BDH1 | BDH1 | Uncharacterized protein | 0.0006911 | 0.0363803 | 1.4851029 |
| PATH:01100 | Metabolic pathways | LOC101102999 | LOC101102999 | Glutamine synthetase | 0.0006911 | 0.0363803 | 1.4851029 |
| PATH:01100 | Metabolic pathways | TPI1 | TPI1 | Triosephosphate isomerase | 0.0006911 | 0.0363803 | 1.4851029 |
| PATH:01100 | Metabolic pathways | GPI | GPI | Glucose-6-phosphate | 0.0006911 | 0.0363803 | 1.4851029 |

| | | | | | | | |
|---|---|---|---|---|---|---|---|
| | | | | isomerase | | | |
| PATH:01100 | Metabolic pathways | REV3L | REV3L | DNA polymerase | 0.0006911 | 0.0363803 | 1.4851029 |
| PATH:01100 | Metabolic pathways | PDHB | PDHB | Uncharacterized protein | 0.0006911 | 0.0363803 | 1.4851029 |
| PATH:01100 | Metabolic pathways | PCCA | PCCA | Uncharacterized protein | 0.0006911 | 0.0363803 | 1.4851029 |
| PATH:01100 | Metabolic pathways | LOC101106131 | LOC101106131 | Cytochrome c oxidase subunit 6A, mitochondrial | 0.0006911 | 0.0363803 | 1.4851029 |
| PATH:01100 | Metabolic pathways | LOC100037702 | LOC100037702 | Transmembrane 7 superfamily member 2 | 0.0006911 | 0.0363803 | 1.4851029 |
| PATH:01100 | Metabolic pathways | NDUFA6 | NDUFA6 | Uncharacterized protein | 0.0006911 | 0.0363803 | 1.4851029 |
| PATH:01100 | Metabolic pathways | HLCS | HLCS | Uncharacterized protein | 0.0006911 | 0.0363803 | 1.4851029 |
| PATH:01100 | Metabolic pathways | ABAT | ABAT | Uncharacterized protein | 0.0006911 | 0.0363803 | 1.4851029 |
| PATH:01100 | Metabolic pathways | PDXP | PDXP | Uncharacterized protein | 0.0006911 | 0.0363803 | 1.4851029 |
| PATH:01100 | Metabolic pathways | APIP | APIP | Methylthioribulose-1-phosphate dehydratase | 0.0006911 | 0.0363803 | 1.4851029 |
| PATH:01100 | Metabolic pathways | ST3GAL6 | ST3GAL6 | Uncharacterized protein | 0.0006911 | 0.0363803 | 1.4851029 |
| PATH:01100 | Metabolic pathways | DCTD | DCTD | Uncharacterized protein | 0.0006911 | 0.0363803 | 1.4851029 |
| PATH:01100 | Metabolic pathways | ST6GALNAC4 | ST6GALNAC4 | Uncharacterized protein | 0.0006911 | 0.0363803 | 1.4851029 |
| PATH:01100 | Metabolic pathways | LOC101123148 | LOC101123148 | L-lactate dehydrogenase B chain-like | 0.0006911 | 0.0363803 | 1.4851029 |
| PATH:01100 | Metabolic pathways | HADHB | HADHB | Uncharacterized protein | 0.0006911 | 0.0363803 | 1.4851029 |
| PATH:01100 | Metabolic pathways | SLC33A1 | SLC33A1 | Uncharacterized protein | 0.0006911 | 0.0363803 | 1.4851029 |
| PATH:01100 | Metabolic pathways | B3GALNT1 | B3GALNT1 | Uncharacterized protein | 0.0006911 | 0.0363803 | 1.4851029 |
| PATH:01100 | Metabolic pathways | CSGALNACT1 | CSGALNACT1 | Uncharacterized protein | 0.0006911 | 0.0363803 | 1.4851029 |
| PATH:01100 | Metabolic pathways | CRYL1 | CRYL1 | Uncharacterized protein | 0.0006911 | 0.0363803 | 1.4851029 |
| PATH:01100 | Metabolic pathways | OAT | OAT | Uncharacterized protein | 0.0006911 | 0.0363803 | 1.4851029 |
| PATH:01100 | Metabolic pathways | PCCB | PCCB | Propionyl coenzyme A carboxylase beta polypeptide | 0.0006911 | 0.0363803 | 1.4851029 |
| PATH:01100 | Metabolic pathways | LOC101104557 | LOC101104557 | Uncharacterized protein | 0.0006911 | 0.0363803 | 1.4851029 |
| PATH:01100 | Metabolic pathways | ALAS1 | ALAS1 | 5-aminolevulinate synthase | 0.0006911 | 0.0363803 | 1.4851029 |
| PATH:01100 | Metabolic pathways | ACSM3 | ACSM3 | Uncharacterized protein | 0.0006911 | 0.0363803 | 1.4851029 |
| PATH:01100 | Metabolic pathways | LOC101116336 | LOC101116336 | Uncharacterized protein | 0.0006911 | 0.0363803 | 1.4851029 |
| PATH:01100 | Metabolic pathways | AK4 | AK4 | Uncharacterized protein | 0.0006911 | 0.0363803 | 1.4851029 |

| | | | | | | | |
|---|---|---|---|---|---|---|---|
| PATH:01100 | Metabolic pathways | ST3GAL1 | ST3GAL1 | Uncharacterized protein | 0.0006911 | 0.0363803 | 1.4851029 |
| PATH:01100 | Metabolic pathways | GBGT1 | GBGT1 | Uncharacterized protein | 0.0006911 | 0.0363803 | 1.4851029 |
| PATH:01100 | Metabolic pathways | LIPG | LIPG | Endothelial lipase | 0.0006911 | 0.0363803 | 1.4851029 |
| PATH:01100 | Metabolic pathways | ASS1 | ASS1 | Uncharacterized protein | 0.0006911 | 0.0363803 | 1.4851029 |
| PATH:01100 | Metabolic pathways | PGK1 | PGK1 | Phosphoglycerate kinase 1 | 0.0006911 | 0.0363803 | 1.4851029 |
| PATH:01100 | Metabolic pathways | DNMT3A | DNMT3A | DNA methyl transferase alpha | 0.0006911 | 0.0363803 | 1.4851029 |
| PATH:01100 | Metabolic pathways | GPT2 | GPT2 | Uncharacterized protein | 0.0006911 | 0.0363803 | 1.4851029 |
| PATH:01100 | Metabolic pathways | ALG3 | ALG3 | Uncharacterized protein | 0.0006911 | 0.0363803 | 1.4851029 |
| PATH:01100 | Metabolic pathways | LOC101123553 | LOC101123553 | Uncharacterized protein | 0.0006911 | 0.0363803 | 1.4851029 |
| PATH:01100 | Metabolic pathways | SDHC | SDHC | Uncharacterized protein | 0.0006911 | 0.0363803 | 1.4851029 |
| PATH:01100 | Metabolic pathways | SMS | SMS | spermine synthase | 0.0006911 | 0.0363803 | 1.4851029 |
| PATH:01100 | Metabolic pathways | PCYT1A | PCYT1A | Uncharacterized protein | 0.0006911 | 0.0363803 | 1.4851029 |
| PATH:01100 | Metabolic pathways | LOC101121811 | LOC101121811 | Uncharacterized protein | 0.0006911 | 0.0363803 | 1.4851029 |
| PATH:01100 | Metabolic pathways | MMAB | MMAB | Uncharacterized protein | 0.0006911 | 0.0363803 | 1.4851029 |
| PATH:01100 | Metabolic pathways | NDUFS1 | NDUFS1 | Uncharacterized protein | 0.0006911 | 0.0363803 | 1.4851029 |
| PATH:01100 | Metabolic pathways | AGPAT9 | AGPAT9 | Uncharacterized protein | 0.0006911 | 0.0363803 | 1.4851029 |
| PATH:01100 | Metabolic pathways | LOC101108849 | LOC101108849 | Uncharacterized protein | 0.0006911 | 0.0363803 | 1.4851029 |
| PATH:01100 | Metabolic pathways | LOC101112287 | LOC101112287 | Uncharacterized protein | 0.0006911 | 0.0363803 | 1.4851029 |
| PATH:01100 | Metabolic pathways | ALAD | ALAD | Delta-aminolevulinic acid dehydratase | 0.0006911 | 0.0363803 | 1.4851029 |
| PATH:01100 | Metabolic pathways | GALNT12 | GALNT12 | Polypeptide N-acetylgalactosaminyltransferase | 0.0006911 | 0.0363803 | 1.4851029 |
| PATH:01100 | Metabolic pathways | IMPDH2 | IMPDH2 | Inosine-5'-monophosphate dehydrogenase | 0.0006911 | 0.0363803 | 1.4851029 |
| PATH:01100 | Metabolic pathways | LDHB | LDHB | L-lactate dehydrogenase | 0.0006911 | 0.0363803 | 1.4851029 |
| PATH:01100 | Metabolic pathways | LPIN3 | LPIN3 | Uncharacterized protein | 0.0006911 | 0.0363803 | 1.4851029 |
| PATH:01100 | Metabolic pathways | CHPT1 | CHPT1 | Uncharacterized protein | 0.0006911 | 0.0363803 | 1.4851029 |
| PATH:01100 | Metabolic pathways | DGAT2 | DGAT2 | Uncharacterized protein | 0.0006911 | 0.0363803 | 1.4851029 |
| PATH:01100 | Metabolic pathways | AMPD3 | AMPD3 | Uncharacterized protein | 0.0006911 | 0.0363803 | 1.4851029 |
| PATH:01100 | Metabolic pathways | GCNT1 | GCNT1 | Uncharacterized protein | 0.0006911 | 0.0363803 | 1.4851029 |

| | | | | | | | |
|---|---|---|---|---|---|---|---|
| PATH:01100 | Metabolic pathways | NMNAT3 | NMNAT3 | Uncharacterized protein | 0.0006911 | 0.0363803 | 1.4851029 |
| PATH:01100 | Metabolic pathways | LOC101114955 | LOC101114955 | uncharacterized LOC101114955 | 0.0006911 | 0.0363803 | 1.4851029 |
| PATH:01100 | Metabolic pathways | SHMT2 | SHMT2 | Serine hydroxymethyltransferase | 0.0006911 | 0.0363803 | 1.4851029 |
| PATH:01100 | Metabolic pathways | IVD | IVD | Uncharacterized protein | 0.0006911 | 0.0363803 | 1.4851029 |
| PATH:01100 | Metabolic pathways | TH | TH | Uncharacterized protein | 0.0006911 | 0.0363803 | 1.4851029 |
| PATH:01100 | Metabolic pathways | UGP2 | UGP2 | Uncharacterized protein | 0.0006911 | 0.0363803 | 1.4851029 |
| PATH:01100 | Metabolic pathways | LOC101112867 | LOC101112867 | enoyl-CoA hydratase, mitochondrial-like | 0.0006911 | 0.0363803 | 1.4851029 |
| PATH:01100 | Metabolic pathways | DLAT | DLAT | Acetyltransferase component of pyruvate dehydrogenase complex | 0.0006911 | 0.0363803 | 1.4851029 |
| PATH:01100 | Metabolic pathways | KHK | KHK | Uncharacterized protein | 0.0006911 | 0.0363803 | 1.4851029 |
| PATH:01100 | Metabolic pathways | HADH | HADH | Uncharacterized protein | 0.0006911 | 0.0363803 | 1.4851029 |
| PATH:01100 | Metabolic pathways | LOC101120934 | LOC101120934 | glutamine synthetase-like | 0.0006911 | 0.0363803 | 1.4851029 |
| PATH:01100 | Metabolic pathways | POLR2D | POLR2D | Uncharacterized protein | 0.0006911 | 0.0363803 | 1.4851029 |
| PATH:01100 | Metabolic pathways | ATP5A1 | ATP5A1 | ATP synthase subunit alpha | 0.0006911 | 0.0363803 | 1.4851029 |
| PATH:01100 | Metabolic pathways | WBSCR17 | WBSCR17 | Williams-Beuren syndrome chromosome region 17 | 0.0006911 | 0.0363803 | 1.4851029 |
| PATH:01100 | Metabolic pathways | HSD17B12 | HSD17B12 | Uncharacterized protein | 0.0006911 | 0.0363803 | 1.4851029 |
| PATH:01100 | Metabolic pathways | CBS | CBS | Cystathionine beta-synthase | 0.0006911 | 0.0363803 | 1.4851029 |
| PATH:01100 | Metabolic pathways | AHCYL2 | AHCYL2 | Adenosylhomocysteinase | 0.0006911 | 0.0363803 | 1.4851029 |
| PATH:01100 | Metabolic pathways | IDH3A | IDH3A | Isocitrate dehydrogenase [NAD] subunit, mitochondrial | 0.0006911 | 0.0363803 | 1.4851029 |
| PATH:01100 | Metabolic pathways | NT5C1A | NT5C1A | Uncharacterized protein | 0.0006911 | 0.0363803 | 1.4851029 |
| PATH:01100 | Metabolic pathways | ALDH18A1 | ALDH18A1 | Uncharacterized protein | 0.0006911 | 0.0363803 | 1.4851029 |
| PATH:01100 | Metabolic pathways | IDH1 | IDH1 | Cytosolic NADP-isocitrate dehydrongenase | 0.0006911 | 0.0363803 | 1.4851029 |
| PATH:00020 | Citrate cycle (TCA cycle) | IDH2 | IDH2 | Uncharacterized protein | 0.0029896 | 0.0608216 | 4.1831797 |
| PATH:00020 | Citrate cycle (TCA cycle) | PC | PC | Pyruvate carboxylase | 0.0029896 | 0.0608216 | 4.1831797 |
| PATH:00020 | Citrate cycle (TCA cycle) | PDHB | PDHB | Uncharacterized protein | 0.0029896 | 0.0608216 | 4.1831797 |
| PATH:00020 | Citrate cycle (TCA cycle) | SDHC | SDHC | Uncharacterized protein | 0.0029896 | 0.0608216 | 4.1831797 |
| PATH:00020 | Citrate cycle (TCA cycle) | DLAT | DLAT | Acetyltransferase | 0.0029896 | 0.0608216 | 4.1831797 |

| | | | | | | | |
|---|---|---|---|---|---|---|---|
| | | | | component of pyruvate dehydrogenase complex | | | |
| PATH:00020 | Citrate cycle (TCA cycle) | IDH3A | IDH3A | Isocitrate dehydrogenase [NAD] subunit, mitochondrial | 0.0029896 | 0.0608216 | 4.1831797 |
| PATH:00020 | Citrate cycle (TCA cycle) | IDH1 | IDH1 | Cytosolic NADP-isocitrate dehydrongenase | 0.0029896 | 0.0608216 | 4.1831797 |
| PATH:00010 | Glycolysis / Gluconeogenesis | ALDH2 | ALDH2 | Uncharacterized protein | 0.0295463 | 0.2092194 | 2.3524457 |
| PATH:00010 | Glycolysis / Gluconeogenesis | TPI1 | TPI1 | Triosephosphate isomerase | 0.0295463 | 0.2092194 | 2.3524457 |
| PATH:00010 | Glycolysis / Gluconeogenesis | GPI | GPI | Glucose-6-phosphate isomerase | 0.0295463 | 0.2092194 | 2.3524457 |
| PATH:00010 | Glycolysis / Gluconeogenesis | PDHB | PDHB | Uncharacterized protein | 0.0295463 | 0.2092194 | 2.3524457 |
| PATH:00010 | Glycolysis / Gluconeogenesis | LOC101123148 | LOC101123148 | L-lactate dehydrogenase B chain-like | 0.0295463 | 0.2092194 | 2.3524457 |
| PATH:00010 | Glycolysis / Gluconeogenesis | PGK1 | PGK1 | Phosphoglycerate kinase 1 | 0.0295463 | 0.2092194 | 2.3524457 |
| PATH:00010 | Glycolysis / Gluconeogenesis | LDHB | LDHB | L-lactate dehydrogenase | 0.0295463 | 0.2092194 | 2.3524457 |
| PATH:00010 | Glycolysis / Gluconeogenesis | DLAT | DLAT | Acetyltransferase component of pyruvate dehydrogenase complex | 0.0295463 | 0.2092194 | 2.3524457 |
| PATH:04310 | Wnt signaling pathway | DVL1 | DVL1 | Uncharacterized protein | 0.0015406 | 0.0448496 | 2.3156888 |
| PATH:04310 | Wnt signaling pathway | PPP3CB | PPP3CB | Serine/threonine-protein phosphatase | 0.0015406 | 0.0448496 | 2.3156888 |
| PATH:04310 | Wnt signaling pathway | FOSL1 | FOSL1 | Uncharacterized protein | 0.0015406 | 0.0448496 | 2.3156888 |
| PATH:04310 | Wnt signaling pathway | CAMK2G | CAMK2G | Uncharacterized protein | 0.0015406 | 0.0448496 | 2.3156888 |
| PATH:04310 | Wnt signaling pathway | CCND3 | CCND3 | Ccnd3 | 0.0015406 | 0.0448496 | 2.3156888 |
| PATH:04310 | Wnt signaling pathway | FZD9 | FZD9 | Uncharacterized protein | 0.0015406 | 0.0448496 | 2.3156888 |
| PATH:04310 | Wnt signaling pathway | SFRP5 | SFRP5 | Uncharacterized protein | 0.0015406 | 0.0448496 | 2.3156888 |
| PATH:04310 | Wnt signaling pathway | CAMK2 | CAMK2 | Ca2+/calmodulin-dependent protein kinase II | 0.0015406 | 0.0448496 | 2.3156888 |
| PATH:04310 | Wnt signaling pathway | NFATC1 | NFATC1 | Uncharacterized protein | 0.0015406 | 0.0448496 | 2.3156888 |
| PATH:04310 | Wnt signaling pathway | RUVBL1 | RUVBL1 | Uncharacterized protein | 0.0015406 | 0.0448496 | 2.3156888 |
| PATH:04310 | Wnt signaling pathway | MYC | MYC | Myc proto-oncogene protein | 0.0015406 | 0.0448496 | 2.3156888 |
| PATH:04310 | Wnt signaling pathway | PPP3R1 | PPP3R1 | Uncharacterized protein | 0.0015406 | 0.0448496 | 2.3156888 |
| PATH:04310 | Wnt signaling pathway | FZD7 | FZD7 | Uncharacterized protein | 0.0015406 | 0.0448496 | 2.3156888 |
| PATH:04310 | Wnt signaling pathway | PRKACA | PRKACA | cAMP-dependent protein kinase catalytic subunit alpha | 0.0015406 | 0.0448496 | 2.3156888 |

| | | | | | | | |
|---|---|---|---|---|---|---|---|
| PATH:04310 | Wnt signaling pathway | SMAD3 | SMAD3 | Smad3 | 0.0015406 | 0.0448496 | 2.3156888 |
| PATH:04310 | Wnt signaling pathway | CCND1 | CCND1 | Cyclin D1 | 0.0015406 | 0.0448496 | 2.3156888 |
| PATH:04310 | Wnt signaling pathway | CAMK2B | CAMK2B | Uncharacterized protein | 0.0015406 | 0.0448496 | 2.3156888 |
| PATH:04310 | Wnt signaling pathway | SFRP2 | SFRP2 | Secreted frizzled-related protein 2 | 0.0015406 | 0.0448496 | 2.3156888 |
| PATH:04310 | Wnt signaling pathway | DKK2 | DKK2 | Uncharacterized protein | 0.0015406 | 0.0448496 | 2.3156888 |
| PATH:04068 | FoxO signaling pathway | AGAP2 | AGAP2 | Uncharacterized protein | 0.000222 | 0.0363803 | 2.771533 |
| PATH:04068 | FoxO signaling pathway | PIK3R2 | PIK3R2 | Uncharacterized protein | 0.000222 | 0.0363803 | 2.771533 |
| PATH:04068 | FoxO signaling pathway | CCNG2 | CCNG2 | Uncharacterized protein | 0.000222 | 0.0363803 | 2.771533 |
| PATH:04068 | FoxO signaling pathway | PRKAG3 | PRKAG3 | 5'-AMP-activated protein kinase subunit gamma-3 | 0.000222 | 0.0363803 | 2.771533 |
| PATH:04068 | FoxO signaling pathway | FOXO6 | FOXO6 | Uncharacterized protein | 0.000222 | 0.0363803 | 2.771533 |
| PATH:04068 | FoxO signaling pathway | IKKBETA | IKKBETA | IKB kinase beta | 0.000222 | 0.0363803 | 2.771533 |
| PATH:04068 | FoxO signaling pathway | CDKN1A | CDKN1A | CDKN1A | 0.000222 | 0.0363803 | 2.771533 |
| PATH:04068 | FoxO signaling pathway | IGFR1 | IGFR1 | Tyrosine-protein kinase receptor | 0.000222 | 0.0363803 | 2.771533 |
| PATH:04068 | FoxO signaling pathway | FOXO3 | FOXO3 | Forkhead box O3 | 0.000222 | 0.0363803 | 2.771533 |
| PATH:04068 | FoxO signaling pathway | GADD45G | GADD45G | Growth arrest and DNA-damage-inducible gamma | 0.000222 | 0.0363803 | 2.771533 |
| PATH:04068 | FoxO signaling pathway | BNIP3 | BNIP3 | Uncharacterized protein | 0.000222 | 0.0363803 | 2.771533 |
| PATH:04068 | FoxO signaling pathway | IRS1 | IRS1 | Uncharacterized protein | 0.000222 | 0.0363803 | 2.771533 |
| PATH:04068 | FoxO signaling pathway | TGFB2 | TGFB2 | Transforming growth factor-beta 2 | 0.000222 | 0.0363803 | 2.771533 |
| PATH:04068 | FoxO signaling pathway | SLC2A4 | SLC2A4 | Uncharacterized protein | 0.000222 | 0.0363803 | 2.771533 |
| PATH:04068 | FoxO signaling pathway | GABARAPL1 | GABARAPL1 | Uncharacterized protein | 0.000222 | 0.0363803 | 2.771533 |
| PATH:04068 | FoxO signaling pathway | SMAD3 | SMAD3 | Smad3 | 0.000222 | 0.0363803 | 2.771533 |
| PATH:04068 | FoxO signaling pathway | CCND1 | CCND1 | Cyclin D1 | 0.000222 | 0.0363803 | 2.771533 |
| PATH:04068 | FoxO signaling pathway | FOXO1 | FOXO1 | Uncharacterized protein | 0.000222 | 0.0363803 | 2.771533 |
| PATH:04068 | FoxO signaling pathway | INSR | INSR | Insulin receptor | 0.000222 | 0.0363803 | 2.771533 |

## Table S11. The description of gene symbol

| Gene symbol | Description |
|---|---|
| ACTN2 | cDNA FLJ51840, highly similar to Alpha-actinin-2 |
| ALDH2 | ALDH2 |
| BCL9L | B-cell CLL/lymphoma 9-like protein |
| BDH1 | 3-hydroxybutyrate dehydrogenase |
| BTG1 | Protein BTG1 |
| CACNB1 | cDNA FLJ45350 fis, clone BRHIP3011567, highly similar to Voltage-dependent L-type calcium |
| CASQ1 | Calsequestrin |
| CASQ2 | Calsequestrin |
| CBS | Cystathionine beta-synthase |
| CCND3 | Cyclin D3 |
| CDC26 | Cell division cycle 26, isoform CRA_a |
| CDH15 | Cadherin-15 |
| CITED2 | Cbp/p300-interacting transactivator with Glu/Asp-rich carboxy-terminal domain 2 |
| COLL11A2 | Collagen alpha-2(XI) chain |
| CSRP3 | cDNA, FLJ93801, Homo sapiens cysteine andglycine-rich protein 3 (cardiac LIMprotein) |
| FHOD3 | FH1/FH2 domain-containing protein 3 |
| FOXO1 | Forkhead box protein O1 |
| FOXP1 | cDNA FLJ58267, highly similar to Forkhead box protein P1 |
| GPX1 | Glutathione peroxidase |
| HEYL | cDNA FLJ52278, highly similar to Homo sapienshairy/enhancer-of-split related with YRPW motif-like (HEYL), mRNA |
| HLF | Hepatic leukemia factor |
| HMOX1 | Heme oxygenase 1 |
| INSR | Insulin receptor |
| ITGA7 | cDNA FLJ12486 fis, clone NT2RM2000566, highly similar to Integrin alpha-7 |
| KIAA1161 | KIAA1161 ortholog |
| LMOD2 | cDNA FLJ50049, highly similar to Mus musculus leiomodin 2 (cardiac) (Lmod2), mRNA |
| LOC100037702 | Transmembrane 7 superfamily member 2 |
| LOC101107037 | Protein tyrosine phosphatase-2 |
| LOC101121811 | Cytochrome c oxidase subunit 8A, mitochondrial |
| LOC443301 | Tyrosine 3-monooxygenase/tryptophan 5-monooxygenase activation protein, eta polypeptide, [illegible] |
| MAFF | Transcription factor MafF |
| MGST3 | Microsomal glutathione S-transferase 3, isoform CRA_a |
| MYH7B | Myosin-7B |
| MYF6 | Myogenic factor 6 |
| MYH6 | myosin, heavy chain 6, cardiac muscle, alpha |
| MYL2 | Myosin light chain 2 |
| MYL6B | myosin, light chain 6B, alkali, smooth muscle and non-muscle |
| MYLK3 | Myosin light chain kinase 3 |
| MYOD1 | Myoblast determination protein 1 |
| MYOG | Myogenin |
| NEO1 | Neogenin homolog 1 (Chicken), isoform CRA_a |
| NFATC1 | cDNA FLJ76687, highly similar to Homo sapiens nuclear factor of activated T-cells, |
| NMNAT3 | Nicotinamide nucleotide adenylyltransferase 3, isoform CRA_g |

| | |
|---|---|
| OMYHCS | Myosin heavy chain slow |
| PAK1 | Non-specific serine/threonine protein kinase |
| PIK3R2 | Phosphoinositide-3-kinase, regulatory subunit 2 (P85 beta), isoform CRA_a |
| PPP3CB | Serine/threonine-protein phosphatase |
| PRKACA | cAMP-dependent protein kinase catalytic subunit alpha |
| PRKAG3 | 5'-AMP-activated protein kinase subunit gamma-3 |
| PTPLA | Very-long-chain (3R)-3-hydroxyacyl-CoA dehydratase 1 |
| RXRG | Retinoic acid receptor RXR-gamma |
| SCARB1 | Scavenger receptor class B, member 1, isoform CRA_b |
| SDHC | cDNA FLJ57874, highly similar to Homo sapiens succinate dehydrogenase complex, subunit C, transcript variant 3, mRNA |
| SETD3 | SET domain containing 3, isoform CRA_a |
| SMAD3 | cDNA FLJ56254, highly similar to Mothers against decapentaplegic homolog 3 (SMAD3) |
| SOCS2 | Suppressor of cytokine signaling 2, isoform CRA_a |
| SPP1 | Secreted phosphoprotein 1 (Osteopontin, bone sialoprotein I, early T-lymphocyte activation 1), |
| STAC3 | SH3 and cysteine rich domain 3, isoform CRA_a |
| XIRP1 | xin actin-binding repeat containing 1 |
| ZBTB16 | zinc finger and BTB domain containing 16 |
